# Supplementary material for: Cobalt catalyst with exclusive metal-centered chirality for asymmetric photocatalysis
Source: Nat Commun. 2025 Jul 18;16:6635. doi: 10.1038/s41467-025-61727-9 (PMC12274477; doi:10.1038/s41467-025-61727-9)
Supplement: Supplementary file 1 — Supplementary Information [file 41467_2025_61727_MOESM1_ESM.pdf]

Supplementary Information

for

Cobalt catalyst with exclusive metal-centered chirality for asymmetric photocatalysis

Suyang Yao,[1,2] Marco Villa,[3] Yuan Zheng,[1] Antonio Fiorentino,[3], Barbara Ventura,[4] Sergei I. Ivlev,[1] Paola Ceroni,[3]* and Eric Meggers[1]*

[1]Fachbereich Chemie, Philipps-Universität Marburg, Hans-Meerwein-Strasse 4, 35043 Marburg, Germany

[2]School of Chemistry and Materials Science, Guangdong University of Education, Guangzhou 510303, China

[3]Department of Chemistry "Giacomo Ciamician", University of Bologna, Via Selmi 2, 40126 Bologna, Italy

[4]Institute for Organic Synthesis and Photoreactivity (ISOF), National Research Council (CNR), Via P. Gobetti 101, 40129 Bologna, Italy

*Email: paola.ceroni@unibo.it; meggers@chemie.uni-marburg.de

Contents

1. General Information........3

2. Synthesis and Characterization of Cobalt Catalysts........5

2.1 Synthesis of the Tridentate Ligands........5

2.2 Synthesis of Bidentate Ligands........9

2.3 Synthesis of Cobalt Complexes **CoL1** and **CoL2**........11

2.4 Synthesis of Racemic Cobalt Complexes *rac*-**CoBr1-4**........13

2.5 Synthesis of Non-Racemic Cobalt Complexes Δ-(*S*)- and Λ-(*S*)-**CoAux1-3**........17

2.6 Synthesis of the Non-Racemic Cobalt Complex Δ-(*S*)-**CoAux4**........23

2.7 Synthesis of Non-Racemic Cobalt Complexes Δ- and Λ-**CoCat1-3**........25

2.9 Synthesis of Complex *rac*-**[CoCat1](NTf_2)$_3$**........29

2.10 Synthesis of Complex Λ-**[CoCat3](BArF)$_3$**........31

3. Determination of the Absolute Configuration of Cobalt Complexes........32

4. Determination of the Enantiomeric Purities of Cobalt Complexes........33

4.1 Enantiomeric Purity of **CoCat1**........33

4.2 Enantiomeric Purity of **CoCat2**........34

4.3 Enantiomeric Purity of **CoCat3**........36

5. Stability Studies of Cobalt Complexes........38

5.1 Stability Studies of Cobalt Complexes in CH_3CN at Elevated Temperature........38

5.2 Stability Studies of Cobalt Complexes in CH_3CN under Irradiation........40

6. Determination of Ligand Dissociation Rate Constants........42

6.1 Rate Constant under Light Irradiation........42

6.2 Rates Constant in the Dark........44

7. Light-Induced Cobalt-Catalyzed Ring Contraction Reaction........46

7.1 Initial Experiments and Optimization of Reaction Conditions........46

7.2 Substrate Scope of Ring Contraction to 2*H*-Azirines........48

8. Mechanistic Experiments........53

8.1 Procedure Using Zinc as Reducing Agent........53

8.2 Reaction in the Dark Using Pre-Irradiated **Δ-CoCat3**........53

8.3 Identification of Biphenyl Byproduct after Irradiation of Cobalt Catalyst........54

8.4 Detection and Identification of Boron Fragments........57

8.5 Catalysis Reaction with **[Co1a](NTf_2)$_3$** as Catalyst........59

9. Photophysical Experiments........60

9.1 Photophysical Measurements........60

9.2 Irradiation Experiment........60

9.3 Transient Absorption Spectroscopy........60

9.4 Absorption Spectra of Cobalt Complexes after Irradiation........61

10. Single Crystal X-Ray Diffraction........63

10.1 Crystal Structure of Complex **Δ-CoCat4**........63

10.2 Crystal Structure of Complex **Co-bpq**........66

11. Enantioselectivities as Determined by Chiral HPLC........69

12. CD Spectra of Chiral Cobalt Complexes........80

13. NMR Spectra........85

14. References........131

1. General Information

All reactions were carried out under an atmosphere of nitrogen with magnetic stirring unless otherwise noted. Catalytic reactions were performed in Schlenk tubes (5 mL). A blue LEDs lamp (24 W) served as light source (λ_{max} = 455 nm; Hongchangzhaoming from Chinese Taobao, https://hongchang-led.taobao.com). **S1**,[1] **S2**,[2] **S4**[3] were prepared according to a reported literature procedure. Ligand **BL1** and **BL2** were prepared according to a reported literature procedure.[4] Isoxazole substrates were synthesized according to a published procedure.[5] Solvents were distilled under nitrogen from calcium hydride (CH_3CN and CH_2Cl_2) or sodium/benzophenone (THF and Et_2O). Reagents that were purchased from commercial suppliers were used without further purification. Flash column chromatography was performed with silica gel 60 M from Macherey-Nagel (irreg. shaped, 230-400 mesh, pH 6.8, pore volume: 0.81 mL × g^{-1}, mean pore size: 66 Å, specific surface: 492 m^2 × g^{-1}, particle size distribution: 0.5% < 25 μm and 1.7% > 71 μm, water content: 1.6%). Preparative layer chromatography (PLC) was performed, using Yantai Huanghai silica gel 60 F_{254} plates purchased from Taobao. The preparative plates were 200 mm of width × 200 mm height × 1 mm phase thickness. ^{1}H NMR, proton decoupled ^{13}C NMR spectra, ^{19}F NMR spectra and ^{11}B spectra were recorded on Bruker AVII 300 (300 MHz), Bruker AVIII 500 (500 MHz) or Bruker AVIII-NEO-600 (600 MHz) spectrometers at ambient temperature. NMR standards were used as follows: ^{1}H NMR spectroscopy: δ = 7.26 ppm ($CDCl_3$), δ = 5.32 ppm (CD_2Cl_2), δ = 1.94 ppm (CD_3CN), δ = 3.31 ppm (CD_3OD). ^{13}C NMR spectroscopy: δ = 77.16 ppm ($CDCl_3$), δ = 54.0 ppm (CD_2Cl_2), δ = 1.32 ppm (CD_3CN), δ = 49.00 ppm (CD_3OD). The multiplicity abbreviations used (or combinations thereof) are: s = singlet, d = doublet, t = triplet, q = quartet, hept = heptet, m = multiplet. All ^{13}C and ^{19}F NMR signals are singlets and account for one carbon or one fluorine atom respectively if not stated otherwise. IR spectra were recorded on a Bruker Alpha FT-IR spectrophotometer. The resulting absorption bands are listed as wavenumbers (cm^{-1}) and the correlating intensities are specified as w (weak), s (strong), or m (medium). CD spectra were recorded on a JASCO J-810 CD

spectropolarimeter (250-600 or 250-700 nm, 2 nm bandwidth, 50 nm/min scanning speed, accumulation of 3 scans). GC-MS analyses were performed using an Agilent 7890A gas chromatograph (Capillary Column: HP-5 Trace Analysis 5%, 30 m, Ø 0.25 mm, film 0.25 μm; injector: 250 °C; oven: 40 °C (2 min), 40 °C to 280 °C (20 °C·min^{-1}); carrier gas: He (1.0 mL min^{-1}) equipped with an Agilent 5973N inert MSD with triple-axis detector operating in EI mode and an Agilent 6890N series auto sampler/injector. High-resolution mass spectrometry was measured via electrospray-ionization-technique (ESI) or atmospheric pressure chemical ionization-technique (APCI) on a Finnigan LTQ-FT Ultra mass spectrometer (Thermo Fischer Scientific). Chiral HPLC chromatography was performed with an Agilent 1260 HPLC system. Optical rotations were measured on a Perkin-Elmer 241 polarimeter with $[\alpha]_D^{22}$ values reported in degrees with concentrations reported in g/100 mL.

2. Synthesis and Characterization of Cobalt Catalysts

2.1 Synthesis of the Tridentate Ligands

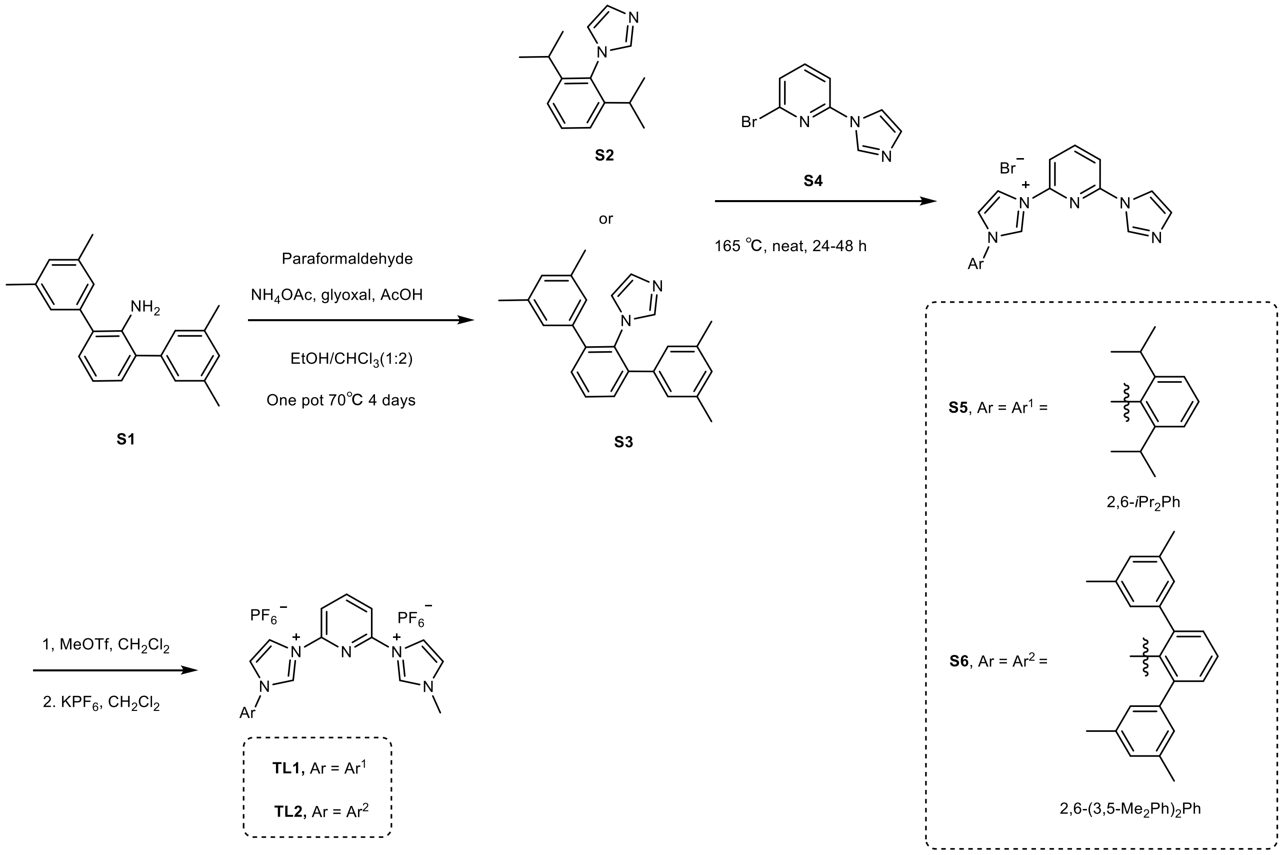

Synthesis of 1-(2,6-di-(3,5-dimethylphenyl)-phenyl)-imidazole (S3): A mixture of substituted aniline **S1** (3.01 g, 10 mmol), ammonium acetate (1.23 g, 16 mmol), glyoxal (40 % wt. in water, 2.90 g, 20 mmol), *p*-formaldehyde (0.60 g, 20 mmol), glacial acetic acid (4.80 g, 80 mmol) and $MgSO_4$ (1.50 g) was dissolved in a mixture of solvents ($CHCl_3$/EtOH: 45mL/20mL) in a round bottomed flask and stirred at 70 °C for 4 days under nitrogen. After cooling to room temperature, the resulting brown solution was concentrated to dryness. After removal of the solvent, the dark residue was neutralized with aqueous 10% KOH solution until the pH = 9. The resulting mixture was extracted with Et_2O (5 × 100 mL). Combined organic layers were then washed with water, dried with Na_2SO_4, and concentrated to give a brown residue. This residue was purified by chromatography on silica gel eluting with *n*-hexane/EtOAc (10:1 to 3:1) to obtain the desired product **S3**. (1.55 g, 44% yield).

^{1}H NMR (300 MHz, CD_3OD): δ 7.57 (dd, *J* = 8.5, 6.7 Hz, 1H), 7.47 (s, 1H), 7.45 (d, *J* = 1.2 Hz, 1H), 7.23 (t, *J* = 1.1 Hz, 1H), 6.90 (dt, *J* = 1.7, 0.9 Hz, 2H), 6.73 (q, *J* = 1.3 Hz, 6H), 2.20 (s, 12H). **^{13}C NMR** (75 MHz, CD_3OD): δ 141.56, 139.90, 139.52, 138.98, 133.90, 131.08, 130.19, 130.03, 128.02, 127.14, 123.70, 21.27. **HRMS** (ESI, *m/z*): calcd. for $C_{25}H_{24}N_2H$ $[M+H]^+$: 353.2012, found: 353.2002. **IR** (film): *v* (cm^{-1}) 3409 (w), 3022 (w), 2920 (m), 2862 (w), 2798 (w), 1607 (m), 1534 (m), 1485 (s), 1459 (w), 1393 (w), 1378 (w), 1326 (w), 1306 (w), 1239 (m), 1220 (w), 1178 (w), 1130 (w), 1104 (w), 1082 (w), 1060 (w), 1004 (w), 903 (w), 854 (w), 804 (m), 763 (w), 732 (w), 707 (w), 670 (w), 654 (w), 622 (w), 607 (w), 544 (w), 532 (w), 517 (w), 494 (w), 420 (w).

Synthesis of S5: 2-Bromo-6-(1*H*-imidazol-1-yl)pyridine (**S4**) (224 mg, 1.0 mmol) and 1-(2,6-diisopropylphenyl)-1H-imidazole (**S2**) (342 mg, 1.5 mmol). were stirred in a sealed tube at 165 ºC under N_2. The mixture was reacted in neat for 40 hours. After cooling to room temperature, the solid residue was dissolved in methanol and concentrated to dryness. The residue was subjected to a flash silica gel chromatography (CH_2Cl_2 /MeOH= 10:1) to give a brown solid (245 mg, 54% yield).

^{1}H NMR (300 MHz, CD_3OD): δ 8.89 (d, *J* = 2.2 Hz, 1H), 8.79 (t, *J* = 1.1 Hz, 1H), 8.39 (t, *J* = 8.1 Hz, 1H), 8.19 (d, *J* = 2.2 Hz, 1H), 8.14 – 8.06 (m, 2H), 8.02 (d, *J* = 8.1 Hz, 1H), 7.69 (dd, *J* = 8.4, 7.3 Hz, 1H), 7.51 (d, *J* = 7.8 Hz, 2H), 7.20 (t, *J* = 1.3 Hz, 1H), 2.57 (p, *J* = 6.8 Hz, 2H), 1.28 (dd, *J* = 8.1, 6.8 Hz, 12H). **^{13}C NMR** (76 MHz, CD_3OD): δ 149.71, 146.92, 146.77, 145.26, 137.06, 133.26, 131.92, 131.03, 127.86, 125.90, 121.77, 118.24, 115.13, 113.37, 29.91, 24.57, 24.29. **HRMS** (ESI, *m/z*): calcd. for $C_{23}H_{26}N_5$ $[M-Br]^+$: 372.2183, found: 372.2181. **IR** (film): *v* (cm^{-1}): 3406 (w), 3085 (w), 2965 (m), 2930 (w), 2870 (w), 1609 (m), 1586 (w), 1534 (m), 1486 (s), 1460 (w), 1388 (w), 1367 (w), 1307 (w), 1253 (w), 1222 (m), 1181 (w), 1105 (w), 1061 (m), 1006 (w), 993 (w), 959 (w), 908 (w), 805 (m), 763 (m), 671 (w), 654 (w), 624 (w), 567 (w), 532 (w), 462 (w), 450 (w), 419 (w).

Synthesis of S6: 2-Bromo-6-(1H-imidazol-1-yl)pyridine (**S4**) (160 mg, 0.71 mmol) and 1-(2,6-di-(3,5-dimethylphenyl)-phenyl)-imidazole (**S3**) (166 mg, 0.47 mmol) were stirred in a sealed tube at 165 ºC under N_2. The mixture was reacted in neat for 24 hours. After cooling to room temperature,

the solid residue was dissolved in methanol and concentrated to dryness. The residue was subjected to a flash silica gel chromatography (CH_2Cl_2 /MeOH= 10:1) to give **S6** as a brown solid (100 mg, 37% yield).

^{1}H NMR (300 MHz, CD_3OD): δ 8.61 (s, 1H), 8.34 (d, *J* = 2.2 Hz, 1H), 8.27 (t, *J* = 8.1 Hz, 1H), 7.98 – 7.90 (m, 2H), 7.86 – 7.76 (m, 2H), 7.70 (d, *J* = 8.0 Hz, 1H), 7.64 (d, *J* = 7.6 Hz, 2H), 7.21 (s, 1H), 6.98 (s, 2H), 6.95 (s, 4H), 2.23 (s, 12H). **^{13}C NMR** (76 MHz, CD_3OD): δ 149.78, 146.29, 145.38, 141.25, 139.87, 137.99, 136.90, 132.52, 131.94, 131.59, 131.14, 131.02, 128.61, 127.42, 120.03, 118.07, 115.23, 112.84, 21.24. **HRMS** (ESI, *m/z*): calcd. for C_{33} H_{30} N_5 $[M-Br]^+$: 496.2496, found: 496.2495. **IR** (film): v (cm^{-1}) 3400 (m), 3083 (w), 2964 (w), 2917 (w), 1606 (m), 1534 (w), 1485 (s), 1459 (w), 1393 (w), 1306 (w), 1238 (m), 1220 (w), 1177 (w), 1104 (w), 1082 (w), 1061 (w), 1004 (w), 993 (w), 908 (w), 854 (w), 804 (m), 763 (m), 707 (w), 670 (w), 653 (w), 623 (w), 604 (w), 540 (w), 520 (w), 490 (w), 440 (w).

Synthesis of TL1: MeOTf (410 mg, 2.5 mmol) was added to a solution of **S5** (452 mg, 1.0 mmol) in 10 mL CH_2Cl_2. The mixture was stirred at room temperature under air overnight. The solution was concentrated to dryness then KPF_6 (3680 mg, 20 mmol) and 10 mL CH_3CN were added into the flask. The solution was stirred overnight and then concentrated to dryness. The residue material was dissolved in CH_2Cl_2 and the organic layer was washed by H_2O. The organic layer was dried over $MgSO_4$, filtered and evaporated under reduced pressure. The residue was subjected to flash silica gel chromatography (CH_2Cl_2 /MeOH= 10:1) to give **TL1** as a brown solid (616 mg, 91% yield).

^{1}H NMR (600 MHz, CD_3CN): δ 9.65 (s, 1H), 9.40 (s, 1H), 8.52 – 8.45 (m, 2H), 8.19 (s, 1H), 8.01 (s, 1H), 7.95 (d, *J* = 8.1 Hz, 1H), 7.82 (s, 1H), 7.67 (t, *J* = 7.9 Hz, 1H), 7.61 (s, 1H), 7.50 (d, *J* = 7.9 Hz, 2H), 3.99 (d, *J* = 2.0 Hz, 3H), 2.52 – 2.48 (m, *J* = 6.9 Hz, 2H), 1.21 (dddd, *J* = 15.0, 6.9, 1.9 Hz, 12H). **^{13}C NMR** (151 MHz, CD_3CN): δ 146.55, 146.50, 146.41, 146.01, 136.58, 136.35, 133.28, 131.02, 127.42, 126.18, 125.82, 121.38, 120.42, 116.23, 116.8, 37.76, 29.39, 24.38, 24.15. **^{19}F NMR** (282 MHz, CD_3CN): δ -71.65, -74.15. **HRMS** (ESI, *m/z*): calcd. for $C_{24}H_{29}N_5F_6P$ $[M-PF_6]^+$: 532.2059,

found: 532.2052. **IR** (film): *v* (cm^{-1}) 3154 (w), 2966 (w), 2927 (w), 1614 (w), 1596 (w), 1535 (w), 1465 (m), 1390 (w), 1367 (w), 1316 (w), 1222 (w), 1111 (w), 1064 (w), 1005 (w), 834 (s), 803 (w), 766 (w), 739 (w), 671 (w), 620 (w), 557 (m).

Synthesis of TL2: MeOTf (356 mg, 2.18 mmol) was added to a solution of **S6** (500 mg, 0.87 mmol) in 10 mL CH_2Cl_2. The mixture was stirred at room temperature under air overnight. The solution was concentrated to dryness then KPF_6 (3680 mg, 20 mmol) and 10 mL CH_3CN were added into the flask. The solution was stirred overnight and then concentrated to dryness. The residue material was dissolved in CH_2Cl_2 and the organic layer was washed by H_2O. The organic layer was dried over $MgSO_4$, filtered and evaporated under reduced pressure. The residue was subjected to a flash silica gel chromatography (CH_2Cl_2 /MeOH= 10:1) to give a **TL2** as brown solid (655 mg, 94% yield).
^{1}H NMR (300 MHz, CD_3CN): δ 9.36 (s, 1H), 9.26 (s, 1H), 8.40 (t, *J* = 8.2 Hz, 1H), 8.09 (t, *J* = 2.0 Hz, 1H), 8.03 (t, *J* = 2.0 Hz, 1H), 7.91 (d, *J* = 8.2 Hz, 1H), 7.84 (t, *J* = 7.7 Hz, 1H), 7.71 (d, *J* = 8.2 Hz, 1H), 7.68 – 7.62 (m, 3H), 7.49 (t, *J* = 2.0 Hz, 1H), 7.02 (s, 2H), 6.94 (s, 4H), 4.03 (s, 3H), 2.25 (s, 12H). **^{13}C NMR** (75 MHz, CD_3CN): δ 146.45, 146.06, 145.91, 140.66, 139.68, 137.40, 137.05, 136.25, 132.56, 131.51, 131.07, 130.86, 128.04, 127.24, 126.17, 120.30, 120.12, 116.44, 116.28, 37.79, 21.24. **^{19}F NMR** (282 MHz, CD_3CN): δ -71.61, -74.11. **HRMS** (ESI, *m/z*): calcd. for $C_{34}H_{33}N_5$ [M-2PF$_6$] $^{2+}$/2: 255.6362, found: 255.6353. **IR** (film): *v* (cm^{-1}) 3423 (w), 3158 (w), 2922 (w), 1612 (w), 1541 (w), 1466 (w), 1228 (w), 1103 (w), 999 (w), 839 (s), 805 (w), 740 (w), 710 (w), 671 (w), 627 (w), 558 (m), 424 (w).

2.2 Synthesis of Bidentate Ligands

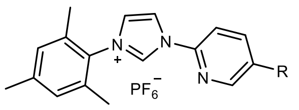

BL1: R= H

BL2: R= CF_3

BL3: R= 2,6-Me_2Ph

Ligand **BL1** and **BL2** were prepared according to reported literature procedures.[4]

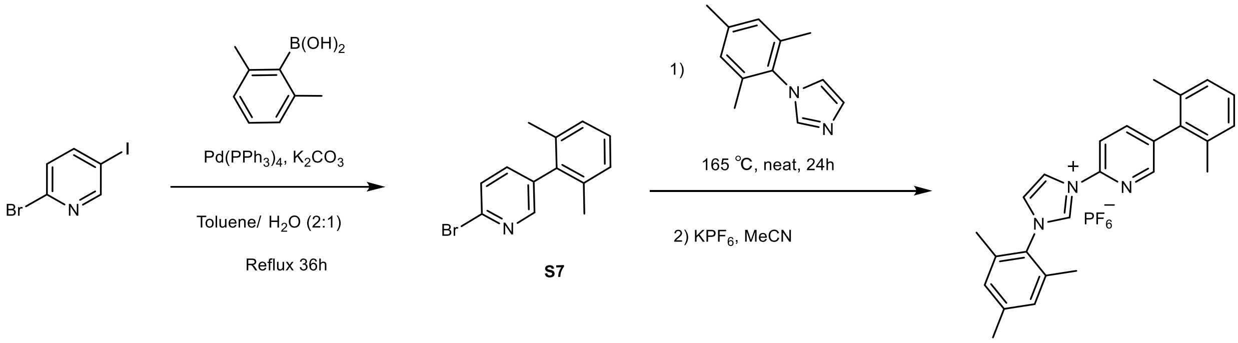

Synthesis of S7: A mixture of 2-bromo-5-iodopyridine (849 mg, 3.0 mmol), (2,6- bis(methyl)phenyl) boronic acid (450 mg, 3.0 mmol), $Pd(PPh_3)_4$ (208 mg, 0.18 mmol, 6 mol%) and K_2CO_3 (1251 mg, 9.0 mmol) in toluene (8 mL) and water (4 mL) in a round bottomed flask was allowed to heat at 110 °C for 36 h. After cooling to room temperature, the layers were separated and the aqueous layer was extracted with CH_2Cl_2.The combined organic layers were washed with water, dried with $MgSO_4$ and concentrated under reduced pressure. The residue was purified by flash column chromatography on silica gel (*n*-hexane/ CH_2Cl_2 = 4:1) to afford compound **S7** (410 mg, 52% yield) as a white solid.

^{1}H NMR (300 MHz, CD_3CN): δ 8.16 (dd, *J* = 2.5, 0.8 Hz, 1H), 7.64 (dd, *J* = 8.1, 0.8 Hz, 1H), 7.47 (dd, *J* = 8.1, 2.5 Hz, 1H), 7.24 – 7.06 (m, 3H), 2.01 (s, 6H). **^{13}C NMR** (75 MHz, CD_3CN): δ 151.38, 141.09, 141.01, 137.56, 137.18, 137.00, 129.20, 128.92, 128.58, 20.97. **HRMS** (ESI, *m/z*): calcd. for $C_{13}H_{12}$BrNH $[M+H]^+$: 262.0226, found: 262.0222. **IR** (film): ν (cm^{-1}) 3038 (w), 2952 (w), 2921 (w), 2857 (w), 1943 (w), 1577 (w), 1547 (w), 1451 (s), 1379 (w), 1354 (w), 1282 (w), 1166 (w), 1128 (w), 1085 (s), 1035 (w), 996 (w), 834 (w), 786 (w), 772 (m), 744 (w), 715 (w), 628 (w), 552 (w), 518 (w), 408 (w).

Synthesis of BL3: Ligand **BL3** was prepared according to reported literature procedures with slight modifications.[6] 2-Bromo-6-(1H-imidazol-1-yl)pyridine (**S7**) (197 mg, 0.75 mmol) and mesitylimidazole (155 mg, 0.83 mmol) were stirred in a sealed tube at 165 ºC under N_2. The mixture was reacted in neat for 24 hours. After cooling to room temperature, KPF_6 (1380 mg, 7.5 mmol) and 10 mL CH_3CN were added into the flask. The solution was stirred overnight and then concentrated to dryness. The residue was subjected to flash silica gel chromatography (CH_2Cl_2/MeOH= 15:1) to give **BL3** as a brown solid (328 mg, 85% yield).

^{1}H NMR (300 MHz, CD_3CN) δ 9.47 (t, *J* = 1.7 Hz, 1H), 8.44 (dd, *J* = 2.2, 0.9 Hz, 1H), 8.40 (t, *J* = 1.9 Hz, 1H), 7.95 (qd, *J* = 8.4, 1.5 Hz, 2H), 7.68 (t, *J* = 1.9 Hz, 1H), 7.31 – 7.08 (m, 5H), 2.39 (s, 3H), 2.14 (s, 6H), 2.06 (s, 6H). **^{13}C NMR** (76 MHz, CD_3CN): δ 150.48, 146.34, 142.61, 142.20, 139.59, 137.22, 135.75, 131.93, 130.55, 129.57, 128.73, 126.12, 121.24, 115.18, 21.16, 20.99, 17.60. **^{19}F NMR** (282 MHz, CD_3CN): δ -71.70, -74.20. **HRMS** (ESI, *m/z*): calcd. for $C_{25}H_{26}N_3$ $[M-PF_6]^+$: 368.2121, found: 368.2113. **IR** (film): *v* (cm^{-1}) 3154 (w), 2924 (w), 2156 (w), 1592 (w), 1542 (w), 1487 (w), 1467 (w), 1384 (w), 1331 (w), 1279 (w), 1243 (w), 1169 (w), 1104 (w), 1059 (w), 1039 (w), 1003 (w), 967 (w), 934 (w), 839 (s), 778 (w), 761 (w), 741 (w), 669 (w), 557 (m), 524 (w).

2.3 Synthesis of Cobalt Complexes CoL1 and CoL2

The complexes **CoL1** and **CoL2** were prepared according to a reported literature procedure with modifications as described below.[7,8]

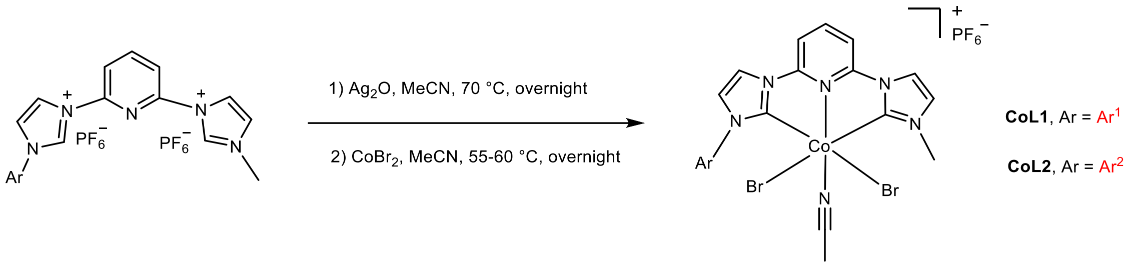

Synthesis of Cobalt Complex CoL1: A mixture of **TL1** (406mg ,0.60 mmol) and Ag_2O (154 mg, 0.66 mmol) was stirred in CH_3CN (15 mL) in the dark at 70 °C overnight under air. Then, the solution was filtered to remove unreacted Ag_2O through a plug of Celite. The filtrate was concentrated to dryness and then dissolved in 8 mL CH_3CN. After that, the **Ag-TL1** solution was added into a flask charged with dried $CoBr_2$ (327 mg, 1.50 mmol) and CH_3CN (2 mL). The mixture was stirred at 60 °C under air for 16 h with reduced light exposure. After cooling to room temperature, the solution was filtered through a plug of Celite and concentrated to dryness. The residue was subjected to flash silica gel chromatography (CH_2Cl_2/CH_3CN = 8:1) to give a green solid **CoL1** (379 mg, 80% yield).

^{1}H NMR (600 MHz, CD_3CN): δ 8.48 (s, 1H), 8.33 (t, J = 8.2 Hz, 1H), 8.19 (s, 1H), 7.80 (d, J = 8.2 Hz, 1H), 7.73 (s, 1H), 7.70 (d, J = 8.2 Hz, 1H), 7.59 (d, J = 7.5 Hz, 2H), 7.52 (d, J = 7.8 Hz, 2H), 4.22 (s, 3H), 3.17 (p, J = 6.7 Hz, 2H), 1.96 (s, 3H), 1.23 (d, J = 6.6 Hz, 6H), 1.18 (d, J = 6.8 Hz, 6H). **^{13}C NMR** (151 MHz, CD_3CN): δ 185.11, 182.38, 153.53, 153.32, 147.98, 146.46, 134.60, 133.77, 132.39, 130.11, 128.59, 125.65, 119.73, 119.25, 118.31, 110.04, 37.89, 29.30, 25.99, 23.58, 1.77. **^{19}F NMR** (282 MHz, CD_3CN): δ -71.48, -73.98. **HRMS** (ESI, *m/z*): calcd. for $C_{26}H_{30}Br_2CoN_6$ $[M-PF_6]^+$: 645.0207, found: 645.0187. **IR** (film): v (cm^{-1}) 3650 (w), 3147 (w), 2965 (w), 2929 (w), 2870 (w), 1698 (w), 1633 (w), 1589 (w), 1540 (w), 1501 (w), 1452 (m), 1406 (w), 1365 (w), 1319 (w), 1300

(w), 1280 (w), 1251 (w), 1229 (w), 1157 (w), 1118 (w), 1060 (w), 978 (w), 926 (w), 841 (s), 806 (w), 781 (w), 763 (w), 738 (w), 700 (w), 680 (w), 558 (m), 454 (w), 431 (w).

Synthesis of Cobalt Complex CoL2: A mixture of **TL2** (600mg ,0.75 mmol) and Ag_2O (191 mg, 0.83 mmol) was stirred in CH_3CN (15 mL) in the dark at 70 °C overnight under air. Then, the solution was filtered to remove unreacted Ag_2O through a plug of Celite. The filtrate was concentrated to dryness and then redissolved in 8 mL dry CH_3CN. The solution was added into a flask charged with dried $CoBr_2$ (410 mg, 1.88 mmol) and CH_3CN (2 mL). The mixture was stirred at 55 °C under air for 16 h with reduced light exposure. After cooling to room temperature, the solution was filtered through a plug of Celite and concentrated to dryness. The residue was subjected to flash silica gel chromatography (CH_2Cl_2/CH_3CN = 8:1) to give a green solid **CoL2** (368 mg, 54% yield).

^{1}H NMR (300 MHz, CD_3CN): δ 8.18 – 8.09 (m, 2H), 8.05 (d, *J* = 2.2 Hz, 1H), 7.97 (d, *J* = 2.2 Hz, 1H), 7.70 (dd, *J* = 8.4, 6.8 Hz, 1H), 7.59 (s, 1H), 7.57 – 7.48 (m, 4H), 7.12 (s, 4H), 6.84 (s, 2H), 4.19 (s, 3H), 2.19 (s, 12H), 1.96 (s, 3H). **^{13}C NMR** (75 MHz, CD_3CN): δ 186.22, 181.19, 153.29, 152.81, 146.07, 143.23, 138.42, 138.38, 134.23, 133.38, 132.73, 131.89, 131.63, 130.20, 128.50, 118.75, 118.31, 117.58, 109.56, 109.29, 37.80, 21.30, 1.77. **^{19}F NMR** (282 MHz, CD_3CN): δ -71.29, -73.80. **HRMS** (ESI, *m/z*) calcd. for $C_{36}H_{34}Br_2CoN_6$ $[M–PF_6]^+$: 767.0538, found: 767.0518. **IR** (film): *v* (cm^{-1}) 3655 (w), 3144 (w), 2919 (w), 1703 (w), 1635 (w), 1605 (w), 1591 (w), 1538 (w), 1501 (m), 1462 (w), 1427 (w), 1329 (w), 1298 (w), 1280 (w), 1237 (w), 1156 (w), 1116 (w), 1041 (w), 1000 (w), 946 (w), 841 (s), 806 (w), 763 (w), 738 (w), 709 (w), 695 (w), 558 (m), 455 (w), 428 (w).

2.4 Synthesis of Racemic Cobalt Complexes *rac*-CoBr1-4

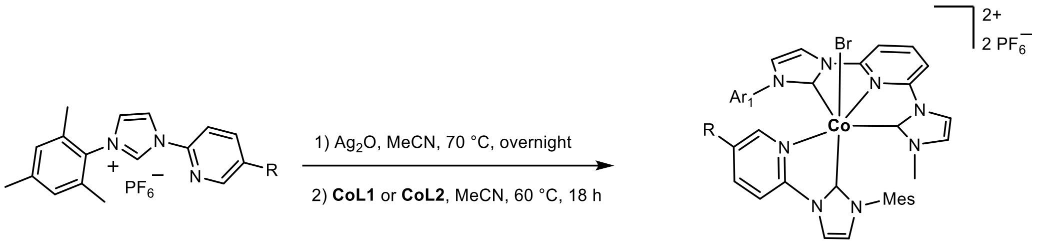

BL1: R= H

BL2: R= CF_3

BL3: R= 2,6-Me_2Ph

rac-**CoBr1:** Ar = Ar^1, R= H, **82% yield**

rac-**CoBr2:** Ar = Ar^2, R= H, **78% yield**

rac-**CoBr3:** Ar = Ar^2, R= CF_3, **80% yield**

rac-**CoBr4:** Ar = Ar^2, R= 2,6-Me_2Ph, **61 % yield**

General Procedures for the Synthesis of Complexes *rac*-CoBr1-4

The racemic complexes *rac*-**CoBr1-4** were prepared according to reported literature procedures with modifications as described in the following.[7,8] A mixture of bidentate ligand **BL1-3** (0.40 mmol) and Ag_2O (92 mg, 0.40 mmol) was stirred in CH_3CN (10 mL) in the dark at 70 °C overnight under air. Then, the solution was filtered to remove unreacted Ag_2O through a plug of Celite. The filtrate was removed to a round-bottom flask and concentrated to dryness. After that, the fresh prepared Ag complex of **BL1-3** was dissolved in 10 mL MeCN and added to the round-bottom flask containing **CoL1** or **CoL2** (0.31 mmol) in 2 mL CH_3CN. The mixture was stirred at 60 °C under air for 18 h without intensive light irradiation. After cooling to room temperature, the solution was filtered through a plug of Celite and concentrated to dryness. The residue was subjected to a flash silica gel chromatography (CH_2Cl_2 / CH_3CN = 8:1).

Synthesis of *rac*-CoBr1: Following the general procedures, **BL1** (164 mg, 0.40 mmol) and **CoL1** (245 mg, 0.31 mmol) were used to obtain the complex *rac*-**CoBr1** as a yellow solid (274 mg, 82% yield).

^{1}H NMR (300 MHz, CD_3CN): δ 8.96 (d, J = 5.8 Hz, 1H), 8.39 (dd, J = 8.9, 2.1 Hz, 2H), 8.20 (t, J = 8.2 Hz, 1H), 8.13 – 8.02 (m, 2H), 7.97 – 7.87 (m, 1H), 7.69 – 7.56 (m, 2H), 7.43 – 7.22 (m, 5H), 7.05 (t, J = 6.6 Hz, 1H), 6.89 (dd, J = 6.6, 2.3 Hz, 1H), 6.78 (d, J = 4.1 Hz, 2H), 2.88 (d, J = 4.9 Hz, 4H),

2.27 (s, 3H), 1.83 – 1.70 (m, 1H), 1.57 (s, 3H), 1.39 (d, *J* = 6.6 Hz, 3H), 1.28 (s, 3H), 1.07 (d, *J* = 6.8 Hz, 3H), 1.00 (d, *J* = 6.7 Hz, 3H), 0.83 (d, *J* = 6.7 Hz, 3H). **^{13}C NMR** (151 MHz, CD_3CN): δ 178.66, 174.23, 164.23, 156.33, 152.62, 152.24, 152.18, 147.69, 146.96, 144.18, 143.95, 142.14, 135.63, 134.91, 133.44, 132.40, 132.14, 131.76, 130.91, 130.84, 130.50, 129.91, 126.02, 125.39, 124.65, 121.30, 119.63, 119.60, 114.31, 110.85, 36.49, 29.48, 29.10, 26.57, 25.84, 23.06, 22.33, 20.99, 16.96, 16.34. **^{19}F NMR** (282 MHz, CD_3CN): δ -71.43, -73.94. **HRMS** (ESI, *m/z*): calcd. for $C_{41}H_{44}BrCoN_8F_6P$ $[M–PF_6]^+$: 933.1820, found: 933.1794 **IR** (film): *ν* (cm^{-1}) 3669 (w), 3174 (w), 3145 (w), 2967 (w), 2927 (w), 1633 (w), 1622 (w), 1592 (w), 1500 (m), 1490 (w), 1461 (w), 1426 (w), 1388 (w), 1353 (w), 1314 (w), 1303 (w), 1282 (w), 1158 (w), 1145 (w), 1118 (w), 1096 (w), 1060 (w), 1042 (w), 1002 (w), 961 (w), 933 (w), 836 (s), 779 (w), 737 (w), 695 (w), 678 (w), 557 (m), 520 (w), 455 (w).

Synthesis of *rac*-CoBr2: Following the general procedures, **BL1** (164 mg, 0.40 mmol) and **CoL2** (283 mg, 0.31 mmol) were used to obtain complex *rac*-**CoBr2** as a yellow solid. (291 mg, 78% yield).

^{1}H NMR (300 MHz, CD_3CN): δ 9.15 (d, *J* = 5.8 Hz, 1H), 8.38 (d, *J* = 2.0 Hz, 1H), 8.26 (d, *J* = 9.6 Hz, 2H), 8.22 – 8.13 (m, 2H), 8.05 (t, *J* = 8.2 Hz, 1H), 7.91 (d, *J* = 1.9 Hz, 1H), 7.48 – 7.30 (m, 4H), 7.28 – 7.24 (m, 1H), 7.20 (s, 2H), 7.11 (d, *J* = 8.2 Hz, 1H), 7.02 (d, *J* = 6.1 Hz, 2H), 6.91 (s, 1H), 6.83 (s, 1H), 6.77 (s, 2H), 6.68 (s, 1H), 6.50 (s, 1H), 2.78 (s, 3H), 2.26 (s, 12H), 2.17 (s, 3H), 1.12 (s, 3H), 0.67 (s, 3H). **^{13}C NMR** (151 MHz, CD_3CN): δ 178.95, 173.27, 164.08, 156.60, 152.54, 152.38, 151.69, 146.54, 144.35, 142.90, 141.74, 140.67, 139.54, 138.65, 137.70, 137.54, 135.82, 135.44, 135.00, 132.42, 132.33, 132.28, 132.02, 131.40, 131.22, 130.52, 130.50, 130.29, 130.20, 129.80, 129.15, 127.60, 125.81, 120.90, 119.35, 117.17, 114.23, 110.31, 110.28, 36.29, 21.54, 21.27, 20.89, 16.28, 15.83. **^{19}F NMR** (282 MHz, CD_3CN): δ -71.58, -74.09. **HRMS** (ESI, *m/z*): calcd. for $C_{51}H_{48}BrCoN_8F_6P$ $[M-PF_6]^+$: 1055.2154, found: 1055.2146. **IR** (film): *ν* (cm^{-1}) 3654 (w), 3147 (w), 2923 (w), 2854 (w), 2231 (w), 2191 (w), 2127 (w), 2051 (w), 1973 (w), 1943 (w), 1622 (w), 1593 (w), 1501 (w), 1460 (w), 1427 (w), 1381 (w), 1354 (w), 1301 (w), 1282 (w), 1158 (w), 1119 (w),

1041 (w), 1000 (w), 946 (w), 840 (s), 778 (w), 741 (w), 708 (w), 694 (w), 678 (w), 558 (m), 520 (w), 484 (w), 428 (w), 416 (w).

Synthesis of *rac*-CoBr3: Following the general procedures, **BL2** (191 mg, 0.40 mmol) and **CoL2** (283 mg, 0.31 mmol) were used to obtain the complex *rac*-**CoBr3** as a yellow solid (315 mg, 80% yield).

^{1}**H NMR** (300 MHz, CD_3CN): δ 9.54 (s, 1H), 8.66 (dd, *J* = 8.7, 1.9 Hz, 1H), 8.52 – 8.43 (m, 2H), 8.32 (d, *J* = 2.2 Hz, 1H), 8.24 (d, *J* = 2.2 Hz, 1H), 8.07 (t, *J* = 8.3 Hz, 1H), 7.95 (d, *J* = 2.2 Hz, 1H), 7.48 (d, *J* = 8.2 Hz, 1H), 7.38 (t, *J* = 7.7 Hz, 1H), 7.33 – 7.26 (m, 2H), 7.18 – 7.06 (m, 5H), 6.92 (s, 1H), 6.86 (s, 1H), 6.81 (s, 2H), 6.70 (s, 1H), 6.53 (s, 1H), 2.78 (s, 3H), 2.26 (d, *J* = 8.8 Hz, 12H), 2.17 (s, 3H), 1.13 (s, 3H), 0.70 (s, 3H). ^{13}**C NMR** (75 MHz, CD_3CN): δ 177.51, 171.78, 165.54, 155.48, 153.07 (q, J = 4.5 Hz), 152.32, 151.62, 146.89, 142.96, 142.90 (q, J = 3.0 Hz), 142.04, 140.66, 139.83, 138.70, 137.48, 137.25, 135.93, 135.33, 134.98, 132.52, 132.35, 132.28, 132.08, 131.50, 131.17, 130.89, 130.63, 130.44, 130.04, 128.96, 127.56, 126.93 (q, J = 35.1 Hz), 121.76, 122.96 (q, J = 272.8 Hz), 119.53, 117.45, 115.49, 110.63, 110.61, 36.69, 21.53, 21.31, 20.91, 16.22, 15.88. ^{19}**F NMR** (282 MHz, CD_3CN): δ -61.96, -71.17, -73.67. **HRMS** (ESI, *m/z*): calcd. for $C_{52}H_{47}BrCoF_9N_8P$ $[M-PF_6]^+$: 1123.2027, found: 1123.1985. **IR** (film): *v* (cm^{-1}) 3145 (w), 3114 (w), 2920 (w), 2851 (w), 1627 (w), 1594 (w), 1502 (m), 1429 (w), 1381 (w), 1330 (w), 1310 (w), 1283 (w), 1265 (w), 1181 (w), 1152 (w), 1121 (w), 1077 (w), 1043 (w), 1001 (w), 947 (w), 839 (s), 737 (m), 704 (w), 694 (w), 677 (w), 628 (w), 557 (m).

Synthesis of *rac*-CoBr4: Following the general procedures, **BL3** (205 mg, 0.40 mmol) and **CoL2** (283 mg, 0.31 mmol) was used to obtain the complex *rac*-**CoBr4** as a yellow solid (244 mg, 61% yield).

^{1}**H NMR** (300 MHz, CD_3CN): δ 9.12 (d, *J* = 1.8 Hz, 1H), 8.46 (d, *J* = 2.2 Hz, 1H), 8.33 (s, 2H), 8.30 (dd, *J* = 8.5, 2.0 Hz, 1H), 8.18 (d, *J* = 8.5 Hz, 1H), 8.07 (t, *J* = 8.3 Hz, 1H), 7.92 (d, *J* = 2.2 Hz, 1H),

7.54 – 7.30 (m, 5H), 7.26 (d, *J* = 2.2 Hz, 1H), 7.16 – 7.00 (m, 4H), 6.87 (s, 1H), 6.76 (d, *J* = 13.2 Hz, 3H), 6.65 (s, 1H), 6.53 (d, *J* = 13.1 Hz, 3H), 2.79 (s, 3H), 2.45 (s, 3H), 2.28 (s, 9H), 2.16 (s, 3H), 1.07 (s, 6H), 0.66 (s, 3H). **^{13}C NMR** (126 MHz, CD_3CN): δ 178.07, 172.38, 164.34, 158.18, 152.06, 151.56, 151.36, 146.54, 146.38, 141.74, 141.57, 139.39, 139.02, 138.17, 137.95, 137.52, 137.14, 136.49, 135.81, 135.32, 134.88, 134.39, 132.62, 132.28, 132.26, 132.11, 131.29, 130.58, 130.53, 130.50, 130.44, 130.40, 130.35, 130.18, 129.64, 128.00, 127.63, 120.93, 119.46, 117.25, 113.17, 110.30, 110.27, 36.36, 22.78, 21.59, 21.10, 20.86, 16.20, 15.58. **^{19}F NMR** (282 MHz, CD_3CN): δ -71.44, -73.94. **HRMS**: (ESI, *m/z*): calcd. for $C_{59}H_{56}BrCoN_8F_6P$ $[M{-}PF_6]^+$: 1159.2780, found: 1159.2759. **IR** (film): *ν* (cm^{-1}) 3660 (w), 3141 (w), 2961 (w), 2924 (w), 2855 (w), 1635 (w), 1610 (w), 1503 (m), 1465 (w), 1379 (w), 1342 (w), 1302 (w), 1282 (w), 1260 (w), 1157 (w), 1091 (w), 1017 (w), 960 (w), 837 (s), 740 (w), 703 (w), 694 (w), 678 (w), 557 (m), 470 (w).

2.5 Synthesis of Non-Racemic Cobalt Complexes Δ-(*S*)- and Λ-(*S*)-CoAux1-3

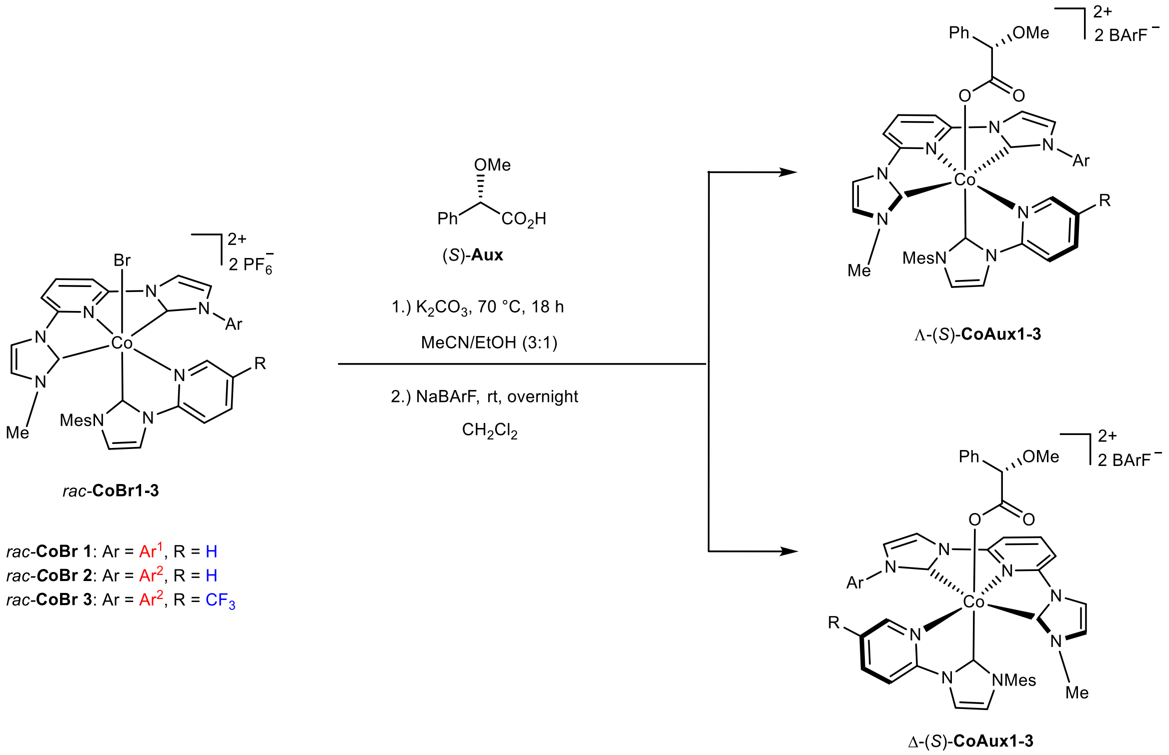

General Procedure for the Synthesis of Δ-(*S*)- and Λ-(*S*)-CoAux1-3

A mixture of *rac*-**CoBr1-3** (0.15 mmol), (*S*)-methoxyphenylacetic acid (200 mg, 1.20 mmol) and K_2CO_3 (84 mg, 0.60 mmol) in CH_3CN/EtOH (3:1, 16 mL) was heated at 70 °C for 18 h under air. The reaction mixture was concentrated to dryness. The residue material was dissolved in CH_2Cl_2 and washed with H_2O. The organic layer was dried over $MgSO_4$, filtered and evaporated under reduced pressure to dryness, and washed with Et_2O to give yellow solid. The precipitate was dissolved in 10 mL CH_2Cl_2 and NaBArF (337 mg ,0.38 mmol) was added to the solution. The mixture was stirred at room temperature overnight under air. Then, CH_2Cl_2 (10 mL) was added and the solution was filtered through a plug of Celite. The filtrate was concentrated to dryness. The residue was subjected to flash silica gel chromatography (CH_2Cl_2/CH_3OH/sat. solution of KPF_6 in MeCN = 200:10:1) or purified by preparative layer chromatography (CH_2Cl_2/CH_3OH = 20:1).

Remark to the flash silica gel chromatography: The addition of KPF_6 to the acetonitrile solvent served to increase the polarity of the eluent. KPF_6 did not induce anion exchange with BArF in these complexes, presumably because the large cation prefers the bulky BArF anion.

Remark to the preparative layer chromatography (PLC): About 100 mg of fresh crude product, previously dissolved in 0.5 mL of CH_2Cl_2, were applied to the plate, subsequently eluted with the mobile phase (CH_2Cl_2 / CH_3OH = 20:1). Finally, the dry eluted PLC plate was exposed to 254 nm UV light, and the two yellow band, located at Rf from 0.15 to 0.40 was scratched from the glass surface. The recovered stationary phase was packed inside a small glass column separately and eluted with CH_3CN contained KPF_6. After solvent evaporation at reduced pressure, the residue material was dissolved in CH_2Cl_2 (20 mL) and washed by H_2O (3 × 8 mL). The organic layer was dried over $MgSO_4$, filtered and evaporated under reduced pressure.

Synthesis of Δ-(*S*)-CoAux1 and Λ-(*S*)-CoAux1: Following the general procedure, *rac*-**CoBr1** (162 mg, 0.15 mmol) was used to obtain complexes Δ-(*S*)-**CoAux1** (140 mg, 36% yield) and Λ-(*S*)-**CoAux1** (135 mg, 35% yield) as yellow solids.

Δ-*(S)*-**CoAux1: PLC** (CH_2Cl_2/CH_3OH = 20:1): R_f= 0.28-0.35. **^{1}H NMR** (300 MHz, CD_3CN): δ 8.42 – 8.33 (m, 2H), 8.29 (d, *J* = 2.1 Hz, 1H), 8.15 (t, *J* = 8.2 Hz, 1H), 8.06 (t, *J* = 7.5 Hz, 1H), 7.84 (d, *J* = 8.2 Hz, 1H), 7.71 (s, 16H), 7.67 (s, 8H), 7.62 (d, *J* = 1.9 Hz, 2H), 7.58 (d, *J* = 1.9 Hz, 1H), 7.53 (d, *J* = 8.2 Hz, 1H), 7.32 – 7.16 (m, 5H), 7.12 (dd, *J* = 7.9, 4.9 Hz, 3H), 7.02 (d, *J* = 2.0 Hz, 1H), 6.95 (d, *J* = 7.3 Hz, 1H), 6.79 (d, *J* = 7.1 Hz, 2H), 6.71 (d, *J* = 12.8 Hz, 2H), 4.44 (s, 1H), 3.05 (s, 3H), 2.60 – 2.60 (m, 1H), 2.59 (s, 3H), 2.23 (s, 3H), 1.79 – 1.69 (m, 1H), 1.53 (s, 3H), 1.38 (d, *J* = 6.7 Hz, 3H), 1.08 (s, 3H), 1.01 (dd, *J* = 6.6, 4.4 Hz, 6H), 0.85 (d, *J* = 6.7 Hz, 3H). **^{13}C NMR** (151 MHz, CD_3CN): δ δ 176.86, 176.81, 171.47, 162.64 (dd, J = 99.7, 49.9 Hz), 161.51, 154.34, 153.11, 152.28, 150.87, 147.17, 147.13, 144.36, 144.24, 142.08, 140.16, 135.69, 135.48, 134.84, 133.46, 132.28, 131.92, 131.84, 130.81, 130.62, 130.18, 129.96 (q, *J* = 28.8 Hz), 129.43, 129.00, 128.70, 127.33, 125.84, 124.85, 124.79, 123.70 (q, *J* = 271.6 Hz), 121.20, 119.37, 118.84, 118.70 (p, *J* = 4.1 Hz), 114.15,

110.25, 110.03, 85.33, 56.98, 36.12, 29.42, 28.61, 26.50, 25.94, 22.87, 22.73, 20.95, 16.98, 16.00. **^{19}F NMR** (282 MHz, CD_3CN): δ -63.25. **HRMS** (ESI, *m/z*): calcd. for $C_{82}H_{65}CoN_8O_3F_{24}B$ [M−BArF]$^+$: 1735.4229, found: 1735.4175 **IR** (film): *v* (cm^{-1}) 3084 (w), 2923 (w), 2853 (w), 1649 (w), 1611 (w), 1503 (w), 1491 (w), 1463 (w), 1354 (m), 1275 (s), 1118 (s), 1001 (w), 932 (w), 887 (w), 839 (w), 793 (w), 776 (w), 745 (w), 713 (w), 682 (m), 671 (w), 584 (w), 450 (w). **CD** (CH_2Cl_2) for Δ-*(S)*-**CoAux1**: λ, nm (Δε, M^{-1} cm^{-1}), 416(-8.8), 343(-9.6), 300(+60.8), 265(+35.4).

Λ-*(S)*-**CoAux1: PLC** (CH_2Cl_2/CH_3OH = 20:1): R*f* = 0.18-0.25. **^{1}H NMR** (300 MHz, CD_3CN): δ 8.31 (d, *J* = 2.2 Hz, 1H), 8.28 (d, *J* = 6.0 Hz, 1H), 8.21 (d, *J* = 2.1 Hz, 1H), 8.05 (t, *J* = 7.3 Hz, 1H), 7.97 (t, *J* = 8.2 Hz, 1H), 7.88 – 7.79 (m, 2H), 7.70 (s, 16H), 7.67 (s, 8H), 7.56 (s, 1H), 7.33 – 7.15 (m, 7H), 7.04 (t, *J* = 7.6 Hz, 2H), 6.95 (dd, *J* = 12.6, 8.2 Hz, 2H), 6.71 (s, 1H), 6.64 – 6.54 (m, 3H), 4.41 (s, 1H), 3.11 (s, 3H), 2.90 (s, 3H), 2.83 – 2.67 (m, 1H), 2.19 (s, 3H), 1.82 – 1.65 (m, 1H), 1.44 (d, *J* = 5.3 Hz, 6H), 1.13 (s, 3H), 1.10 (d, *J* = 6.8 Hz, 3H), 1.01 (d, *J* = 6.8 Hz, 3H), 0.83 (d, *J* = 6.7 Hz, 3H). **^{13}C NMR** (75 MHz, CD_3CN): δ 177.38, 176.88, 173.77, δ 162.62 (dd, J = 99.7, 49.9 Hz), 161.75, 152.66, 152.32, 152.19, 151.76, 147.11, 146.89, 144.36, 144.18, 142.05, 139.66, 135.67, 135.46, 134.65, 133.45, 132.33, 131.83, 131.81, 130.69, 130.48, 129.94 (q, *J* = 28.6 Hz), 129.39, 129.35, 128.69, 127.23, 125.91, 124.97, 124.89, 123.67 (q, *J* = 271.7 Hz), 121.21, 119.14, 118.92, 118.78 – 118.62 (m), 114.11, 110.19, 110.03, 85.38, 57.07, 36.58, 29.43, 28.64, 26.61, 25.74, 22.76, 22.73, 20.90, 16.78, 16.03. **^{19}F NMR** (282 MHz, CD_3CN): δ -63.25. **HRMS** (ESI, *m/z*): calcd. for $C_{82}H_{65}CoN_8O_3F_{24}B$ [M−BArF]$^+$: 1735.4229, found: 1735.4169 **IR** (film): *v* (cm^{-1}): 2923 (w), 2853 (w), 1636 (w), 1611 (w), 1502 (w), 1491 (w), 1463 (w), 1427 (w), 1354 (m), 1276 (s), 1121 (s), 1001 (w), 932 (w), 887 (w), 839 (w), 776 (w), 743 (w), 712 (w), 682 (m), 670 (w), 448 (w). **CD** (CH_2Cl_2) for Λ-*(S)*-**CoAux1**: λ, nm (Δε, M^{-1} cm^{-1}), 460(-5.0), 348(+36.1), 294(-64.6), 266(-44.2).

Synthesis of Δ-(*S*)-CoAux2 and Λ-(*S*)-CoAux2: Following the general procedure, *rac*-**CoBr2** (180 mg, 0.15 mmol) was used to obtain the complexes Δ-(*S*)-**CoAux2** (142 mg, 35% yield) and Λ-(*S*)-**CoAux2** (130 mg, 32% yield) as yellow solids.

Δ-(*S*)-CoAux2: PLC (CH_2Cl_2/CH_3OH = 20:1): R_f = 0.27-0.33. **^{1}H NMR** (300 MHz, CD_3CN): δ 8.27 (dd, *J* = 9.6, 2.0 Hz, 2H), 8.21 (t, *J* = 7.4 Hz, 1H), 8.15 – 8.02 (m, 3H), 7.93 (d, *J* = 2.0 Hz, 1H), 7.80 (d, *J* = 2.0 Hz, 1H), 7.72 (s, 16H), 7.67 (s, 8H), 7.56 – 7.47 (m, 2H), 7.41 (t, *J* = 7.8 Hz, 1H), 7.28 (t, *J* = 6.6 Hz, 1H), 7.17 (q, *J* = 5.4, 5.0 Hz, 3H), 7.12 – 6.97 (m, 6H), 6.93 (d, *J* = 2.1 Hz, 1H), 6.81 (d, *J* = 8.1 Hz, 3H), 6.68 (d, *J* = 10.8 Hz, 3H), 6.51 (s, 1H), 3.94 (s, 1H), 2.80 (s, 3H), 2.27 (s, 6H), 2.21 (s, 6H), 2.17 (s, 3H), 1.04 (s, 3H), 0.64 (s, 3H). **^{13}C NMR** (126 MHz, CD_3CN): δ 176.73, 176.71, 169.95, δ 162.61 (dd, *J* = 99.7, 49.8 Hz), 160.09, 154.61, 153.22, 152.35, 150.38, 147.11, 144.32, 141.69, 141.54, 140.44, 140.35, 139.46, 139.20, 137.76, 137.31, 135.66, 135.37, 134.96, 134.34, 132.29, 132.21, 131.97, 131.90, 131.57, 131.14, 131.08, 130.48, 130.23, 130.04, 129.92 (q, *J* = 29.2 Hz), 129.50, 129.15, 129.04, 128.05, 128.01, 127.60, 124.84, 123.30 (q, *J* = 271.8 Hz), 120.69, 119.02, 118.77 – 118.57 (m), 117.78, 114.03, 110.00, 109.86, 84.51, 56.07, 35.47, 21.55, 21.46, 20.87, 16.30, 15.79. **^{19}F NMR** (282 MHz, CD_3CN): δ -63.24. **HRMS** (ESI, *m/z*): calcd. for $C_{92}H_{69}BCoF_{24}N_8O_3$ $[M{-}BArF]^+$: 1859.4543, found: 1859.4491. **IR** (film): *ν* (cm^{-1}) 2922 (m), 2852 (w), 1655 (w), 1609 (w), 1503 (w), 1492 (w), 1460 (w), 1354 (m), 1274 (s), 1117 (s), 1001 (w), 932 (w), 887 (w), 855 (w), 839 (w), 805 (w), 791 (w), 776 (w), 744 (w), 711 (m), 682 (m), 670 (w), 620 (w), 581 (w), 449 (w). **CD** (CH_2Cl_2) for **Δ-(*S*)-CoAux2:** λ, nm (Δε, M^{-1} cm^{-1}), 472(+3.6), 422(-5.5), 345(-17.0), 319(+59.8), 291(-45.7), 273(+110.9).

Λ-(*S*)-CoAux2: PLC (CH_2Cl_2/CH_3OH = 20:1): R_f = 0.18-0.26. **^{1}H NMR** (300 MHz, CD_3CN): δ 8.47 (d, *J* = 1.9 Hz, 1H), 8.43 – 8.36 (m, 2H), 8.27 (t, *J* = 7.6 Hz, 1H), 8.17 – 8.05 (m, 2H), 7.85 – 7.57 (m, 27H), 7.38 (t, *J* = 7.7 Hz, 1H), 7.30 (d, *J* = 6.7 Hz, 3H), 7.18 – 6.99 (m, 5H), 6.98 – 6.85 (m, 3H), 6.82 (s, 3H), 6.58 (d, *J* = 8.2 Hz, 2H), 6.30 (s, 1H), 6.05 (d, *J* = 7.5 Hz, 2H), 3.61 (s, 1H), 2.98 (s, 3H), 2.78 (s, 3H), 2.41 (s, 6H), 2.23 (s, 6H), 2.06 (s, 3H), 0.99 (s, 3H), 0.47 (s, 3H). **^{13}C NMR** (151 MHz, CD_3CN): δ 178.07, 176.43, 173.06, 162.63 (dd, *J* = 99.7, 49.8 Hz), 161.87, 152.80, 152.50, 151.93, 150.76, 146.44, 144.59, 142.18, 141.58, 141.31, 139.45, 139.40, 139.24, 138.92, 137.64, 135.69, 135.11, 134.84, 133.19, 132.63, 132.12, 131.94, 131.40, 131.35, 131.13, 130.17, 129.94, 129.86, 129.82, 129.74 (d, *J* = 23.0 Hz), 129.27, 128.99, 128.72, 128.26, 127.78, 126.85, 125.35,

123.69 (d, *J* = 271.8 Hz), 120.91, 118.83, 118.75 – 118.62 (m), 116.72, 114.10, 109.70, 109.36, 84.00, 56.36, 36.27, 21.62, 21.39, 20.77, 16.13, 15.35. **^{19}F NMR** (282 MHz, CD_3CN): δ -63.23. **HRMS** (ESI, *m/z*): calcd. for $C_{92}H_{69}BCoF_{24}N_8O_3$ [M–BArF]$^+$: 1859.4543, found: 1859.4512. **IR** (film): *v* (cm^{-1}) 2922 (w), 2852 (w), 1655 (w), 1609 (w), 1503 (w), 1492 (w), 1460 (w), 1354 (m), 1275 (s), 1120 (s), 1001 (w), 932 (w), 887 (w), 855 (w), 839 (w), 805 (w), 791 (w), 776 (w), 745 (w), 712 (w), 682 (m), 670 (w), 450 (w), 581 (w), 449 (w). **CD** (CH_2Cl_2) for Λ-(*S*)-**CoAux2:** λ, nm (Δε, M^{-1} cm^{-1}), 457(-7.7), 397(+1.7), 348(+38.1), 314(-49.9), 289(+21.3), 264(-99.8).

Synthesis of Δ-(*S*)-CoAux3 and Λ-(*S*)-CoAux3: Following the general procedure, ***rac*-CoBr3** (190 mg, 0.15 mmol) was used to obtain the complexes Δ-(*S*)-**CoAux3** (125 mg, 30% yield) and Λ-(*S*)-**CoAux3** (118 mg, 28% yield) as yellow solids. Notice: Separation of the two diastereomers was achieved by preparative layer chromatography.

Δ-(*S*)-CoAux3: PLC (CH_2Cl_2/CH_3OH = 20:1): R_f = 0.29-0.40. **^{1}H NMR** (300 MHz, CD_3CN): δ 8.73 (s, 1H), 8.63 (d, *J* = 10.6 Hz, 1H), 8.45 – 8.34 (m, 2H), 8.29 (d, *J* = 2.1 Hz, 1H), 8.18 (s, 1H), 8.00 (d, *J* = 2.1 Hz, 1H), 7.86 (d, *J* = 2.0 Hz, 1H), 7.73 (s, 16H), 7.67 (s, 8H), 7.57 – 7.48 (m, 2H), 7.43 (t, *J* = 7.7 Hz, 1H), 7.22 – 7.07 (m, 5H), 7.04 (s, 4H), 6.99 (d, *J* = 2.2 Hz, 1H), 6.84 – 6.71 (m, 5H), 6.68 (s, 1H), 6.54 (s, 1H), 4.02 (s, 1H), 2.79 (s, 3H), 2.24 (s, 12H), 2.18 (s, 3H), 1.97 (s, 3H), 1.02 (s, 3H), 0.68 (s, 3H). **^{13}C NMR** (126 MHz, CD_3CN): 176.98, 174.99, 168.09, 162.63 (dd, *J* = 99.7, 49.8 Hz), 161.34, 155.15, 154.58, 153.35, 147.42, 146.97 (q, *J* = 4.5 Hz), 143.03 (q, *J* = 3.3 Hz), 141.94, 141.76, 140.35, 140.15, 139.71, 139.39, 137.58, 137.05, 135.68, 135.28, 134.88, 134.52, 132.54, 132.28, 131.95, 131.86, 131.39, 131.36, 131.23, 130.72, 130.60, 130.33, 129.94 (q, J = 28.5 Hz), 129.58, 129.48, 129.20, 127.84, 127.56, 127.54, 126.51 (q, *J* = 35.2 Hz), 123.32 (q, *J* = 271.8 Hz), 123.09 (q, *J* = 272.8 Hz), 121.61, 119.17, 118.75 – 118.62 (m), 117.99, 115.27, 110.39, 110.21, 84.72, 55.76, 35.73, 21.46, 20.89, 16.21, 15.88. **^{19}F NMR** (282 MHz, CD_3CN): δ -61.73, -63.25. **HRMS** (ESI, *m/z*) calcd for $C_{93}H_{68}BCoF_{27}N_8O_3$ [M–BArF]$^+$: 1927.4417, found: 1927.4377. **IR** (film): *v* (cm^{-1}) 3089 (w), 2926 (w), 1660 (w), 1626 (w), 1609 (w), 1504 (w), 1430 (w), 1353 (m), 1330 (w), 1307 (w),

1274 (s), 1116 (s), 1045 (w), 1001 (w), 947 (w), 932 (w), 887 (w), 854 (w), 839 (m), 806 (w), 790 (w), 744 (w), 711 (m), 682 (m), 670 (w), 622 (w), 583 (w), 559 (w), 508 (w), 449 (w), 421 (w). **CD** (CH_2Cl_2) for Δ-(*S*)-**CoAux3**: λ, nm (Δε, M^{-1} cm^{-1}), 484(+3.2), 421(-10.4), 330(+32.1), 295(-55.2).

Λ-(*S*)-CoAux3: PLC (CH_2Cl_2/CH_3OH = 20:1): R_f = 0.17-0.29. **^{1}H NMR** (300 MHz, CD_3CN): δ 8.98 (s, 1H), 8.67 (d, *J* = 8.7 Hz, 1H), 8.44 (d, *J* = 2.3 Hz, 1H), 8.41 (d, *J* = 8.7 Hz, 1H), 8.16 (s, 2H), 7.86 (t, *J* = 8.2 Hz, 1H), 7.74 – 7.69 (m, 16H), 7.67 (s, 8H), 7.64 (d, *J* = 2.2 Hz, 1H), 7.45 – 7.37 (m, , 2H), 7.21 – 7.06 (m, 6H), 7.04 (d, *J* = 2.2 Hz, 2H), 6.95 (t, *J* = 7.6 Hz, 2H), 6.82 (d, *J* = 4.9 Hz, 3H), 6.73 (d, *J* = 8.2 Hz, 1H), 6.59 (s, 1H), 6.39 (s, 1H), 6.29 (d, *J* = 7.3 Hz, 2H), 3.88 (s, 1H), 2.95 (s, 3H), 2.69 (s, 3H), 2.31 (s, 6H), 2.24 (s, 6H), 2.09 (s, 3H), 1.00 (s, 3H), 0.57 (s, 3H). **^{13}C NMR** (126 MHz, CD_3CN): δ 177.26, 176.11, 170.21, 162.58 (dd, *J* = 99.7, 49.9 Hz), 162.45, 155.21, 152.58, 151.32, 149.43 (q, *J* = 4.3 Hz), 147.02, 142.90 (q, *J* = 3.1 Hz), 142.31, 141.83, 140.48, 139.66, 139.26, 139.15, 138.09, 137.08, 135.63, 135.05, 134.78, 134.72, 132.49, 132.29, 132.22, 131.88, 131.41, 131.17, 131.02, 130.50, 130.28, 130.05, 129.64 (q, *J* = 28.6 Hz), 129.38, 129.10, 128.68, 128.07, 127.61, 126.73, 126.37 (q, *J* = 35.3 Hz), 125.44 (q, *J* = 271.7 Hz), 123.08(q, *J* = 272.2.3 Hz), 121.63, 118.96, 118.76 – 118.58 (m), 117.53, 115.15, 109.99, 109.90, 84.50, 56.36, 36.55, 21.49, 21.41, 20.79, 16.09, 15.55. **^{19}F NMR** (282 MHz, CD_3CN): δ -62.03, -63.25. **HRMS** (ESI, *m/z*) calcd for $C_{93}H_{68}BCoF_{27}N_8O_3$ [M–BArF]$^+$: 1927.4417, found: 1927.4380. **IR** (film): *v* (cm^{-1}) 2923 (w), 2852 (w), 1656 (w), 1609 (w), 1503 (w), 1460 (w), 1354 (m), 1276 (s), 1122 (s), 1001 (w), 932 (w), 887 (w), 855 (w), 839 (w), 805 (w), 790 (w), 776 (w), 745 (w), 712 (w), 682 (w), 670 (w). **CD** (CH_2Cl_2) for Λ-(*S*)-**CoAux3**: λ, nm (Δε, M^{-1} cm^{-1}), 475(-3.9), 414(+4.2), 364(-11.5), 327(-25.2), 296(+51.9).

2.6 Synthesis of the Non-Racemic Cobalt Complex Δ-(*S*)-CoAux4

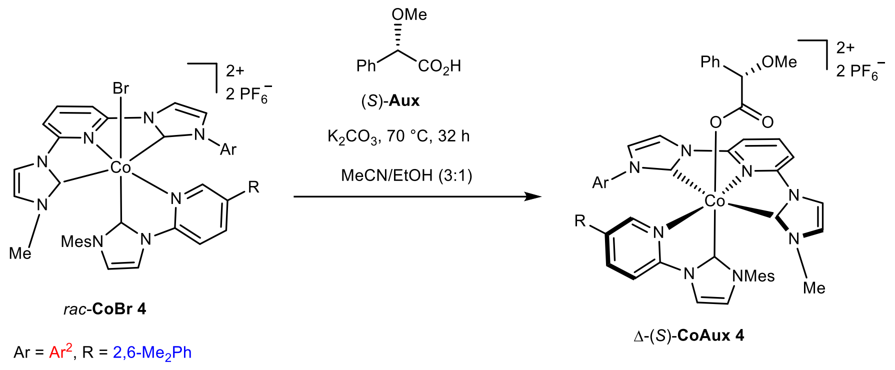

A mixture of *rac*-**CoBr4** (200 mg, 0.15 mmol), (*S*)-methoxyphenylacetic acid (256 mg, 1.50 mmol) and K_2CO_3 (100 mg, 0.70 mmol) in CH_3CN/EtOH (3:1, 16 mL) was heated at 70 °C for 32 h under air. The reaction mixture was concentrated to dryness. The residue material was dissolved in CH_2Cl_2 (30 mL) and washed by H_2O (3 × 15 mL). The organic layer was dried over $MgSO_4$, filtered, evaporated under reduced pressure to dryness, and washed with Et_2O to afford a yellow solid. The solid was subjected to flash silica gel chromatography (CH_2Cl_2/CH_3OH/sat. solution of KPF_6 in MeCN = 200:10:1) or purified by preparative layer chromatography (CH_2Cl_2/CH_3OH = 20:1). Δ-(*S*)-**CoAux4** was obtain as a yellow solid (56 mg, 27% yield).

Δ-(*S*)-CoAux4: TLC (CH_2Cl_2/CH_3OH = 20:1): R_f = 0.25-0.29. **^{1}H NMR** (500 MHz, CD_3CN): δ 8.39 (d, *J* = 2.2 Hz, 1H), 8.37 (d, *J* = 1.9 Hz, 1H), 8.33 (d, *J* = 2.2 Hz, 1H), 8.27 (dd, *J* = 8.5, 2.1 Hz, 1H), 8.20 (d, *J* = 2.2 Hz, 1H), 8.13 (d, *J* = 8.5 Hz, 1H), 8.08 (t, *J* = 8.2 Hz, 1H), 7.55 – 7.50 (m, 2H), 7.45 (d, *J* = 4.1 Hz, 2H), 7.40 (d, *J* = 2.2 Hz, 1H), 7.39 – 7.34 (m, 1H), 7.22 (dd, *J* = 7.8, 1.5 Hz, 1H), 7.16 – 7.08 (m, 2H), 7.02 (d, *J* = 2.2 Hz, 1H), 6.98 (t, *J* = 7.7 Hz, 2H), 6.92 – 6.88 (m, 3H), 6.85 (s, 1H), 6.80 (s, 2H), 6.55 (d, *J* = 7.9 Hz, 3H), 6.49 – 6.41 (m, 3H), 3.61 (s, 1H), 2.72 (s, 3H), 2.48 (s, 3H), 2.45 (s, 3H), 2.26 (s, 6H), 2.18 (s, 9H), 2.13(s, 3H), 0.84 (s, 3H), 0.63 (s, 3H). **^{13}C NMR** (126 MHz, CD_3CN): δ 175.18, 168.76, 160.69, 154.59, 152.32, 152.08, 151.38, 146.97, 146.77, 141.68, 141.47, 140.95, 139.36, 139.28, 138.94, 138.05, 137.56, 137.39, 136.99, 135.80, 135.65, 135.31, 134.77, 134.68, 133.01, 132.74, 132.28, 132.10, 131.43, 131.32, 131.27, 130.43, 130.40, 130.26, 129.83,

129.37, 128.77, 128.61, 127.78, 127.51, 127.43, 121.00, 117.44, 113.39, 109.89, 109.67, 84.41, 55.51, 36.07, 22.84, 22.62, 21.48, 21.28, 20.83, 15.74, 15.65. **^{19}F NMR** (282 MHz, CD_3CN): δ -71.54, -74.04. **HRMS** (ESI, *m/z*): calcd. for $C_{68}H_{65}CoF_6N_8O_3$ P [M–PF6]$^+$: 1245.4148, found: 1245.4130. **IR** (film): *v* (cm^{-1}): 3650 (w), 3144 (w), 2922 (m), 2853 (w), 1747 (w), 1639 (w), 1610 (w), 1504 (m), 1463 (w), 1377 (w), 1338 (w), 1304 (w), 1281 (w), 1194 (w), 1158 (w), 1105 (w), 1030 (w), 1000 (w), 961 (w), 841 (s), 741 (w), 703 (w), 558 (m). **CD** (CH_2Cl_2) for Δ-(*S*)-**CoAux 4**: λ, nm (Δε, M^{-1} cm^{-1}), 425(-10.8), 353(-5.4), 330(+16.4), 304(+35.1).

2.7 Synthesis of Non-Racemic Cobalt Complexes Δ- and Λ-CoCat1-3

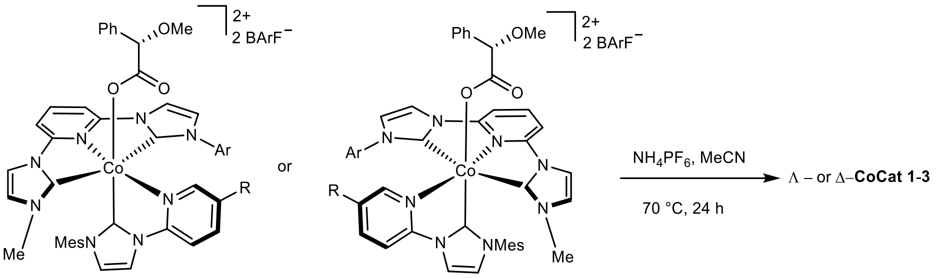

Λ-(*S*)-**CoAux1-3** Δ-(*S*)-**CoAux1-3**

Λ or Δ-(*S*)-**CoAux 1**: Ar = Ar^1, R = H
Λ or Δ-(*S*)-**CoAux 2**: Ar = Ar^2, R = H
Λ or Δ-(*S*)-**CoAux 3**: Ar = Ar^2, R = CF_3

To a solution of Δ-(*S*)-**CoAux1-3** or Λ-(*S*)-**CoAux1-3** (0.05 mmol) in CH_3CN (5 mL) was added NH_4PF_6 (0.75 mmol, 122 mg) and the tube sealed. The resulting mixture was stirred at 70 °C for 24 h. The reaction mixture was evaporated to dryness, dissolved in CH_2Cl_2 (30 mL) and washed by H_2O (3 × 15 mL). The organic layer was dried over $MgSO_4$, filtered and evaporated under reduced pressure to dryness. The obtained solid was washed with cold Et_2O and dried under vacuo to provide a yellow solid.

Synthesis of Δ- and Λ-CoCat1: Following the general procedure, Δ-(*S*)-**CoAux 1** or Λ-(*S*)-**CoAux** (130 mg, 0.05 mmol) was used to provide Δ-**CoCat1** (113 mg, 86% yield) or Λ-**CoCat1** (113 mg, 86% yield) as a yellow solid. Note: *rac*-**CoCat1** was synthesized following the same procedure using a 1:1 mixture of Δ-(*S*)-**CoAux1** and Λ-(*S*)-**CoAux1** as the starting material.

^{1}H NMR (300 MHz, CD_3CN): δ 8.58 (d, *J* = 1.8 Hz, 1H), 8.49 (d, *J* = 2.0 Hz, 1H), 8.40 (t, *J* = 8.3 Hz, 1H), 8.25 (q, *J* = 8.1, 6.5 Hz, 3H), 8.08 (d, *J* = 8.1 Hz, 1H), 7.77 (d, *J* = 11.2 Hz, 18H), 7.68 (s, 8H), 7.59 (d, *J* = 8.3 Hz, 1H), 7.51 (d, *J* = 1.8 Hz, 1H), 7.38 – 7.26 (m, 4H), 6.99 (d, *J* = 5.5 Hz, 1H), 6.85 (d, *J* = 3.8 Hz, 2H), 3.00 (s, 3H), 2.51 (p, *J* = 6.7 Hz, 1H), 2.30 (s, 3H), 2.25 (s, 3H), 1.79 – 1.64 (m, 1H), 1.57 (s, 3H), 1.41 (d, *J* = 6.7 Hz, 3H), 1.28 (s, 3H), 1.09 (t, *J* = 5.8 Hz, 6H), 0.89 (d, *J* = 6.6 Hz, 3H). **^{13}C NMR** (126 MHz, CD_3CN): δ 170.87, 166.44, δ 162.60 (dd, *J* = 99.7, 49.8 Hz), 154.40, 153.09, 152.36, 152.28, 151.61, 148.89, 146.71, 145.84, 143.82, 142.62, 135.66, 134.83, 132.86,

132.78, 132.65, 131.42, 131.03, 130.96, 130.80, 129.90 (q, J = 31.6 Hz), 126.38, 125.93, 125.45 (d, J = 271.8 Hz), 125.16, 122.00, 121.14, 120.94, 118.80 – 118.59 (m), 115.47, 112.58, 112.29, 37.03, 29.57, 29.05, 26.84, 25.91, 22.46, 20.98, 16.96, 16.26, 6.10. **^{19}F NMR** (282 MHz, CD_3CN): δ -63.25, -71.11, -73.61. **HRMS** (ESI, *m/z*): calcd. for C_{107} $H_{71}CoN_9F_{48}B_2$ [M–PF6]$^+$: 2474.4607, found: 2474.4575. **IR** (film): v (cm^{-1}) 2978 (w), 1610 (w), 1499 (w), 1464 (w), 1428 (w), 1354 (m), 1276 (s), 1117 (s), 1001 (w), 932 (w), 887 (w), 838 (w), 793 (w), 773 (w), 744 (w), 712 (m), 682 (m), 670 (w), 580 (w), 517 (w), 449 (w).

CD(CH_2Cl_2) for Δ-**CoCat1**: λ, nm (Δε, M^{-1} cm^{-1}), 444(+5.9), 393(-2.4), 337(-16.8), 296(+65.8).

CD(CH_2Cl_2) for Λ-**CoCat1**: λ, nm (Δε, M^{-1} cm^{-1}), 444(-6.0), 393(+1.8), 337(+14.4), 296(-64.6).

Synthesis of Δ- and Λ-CoCat2: Following the general procedure, Δ-(*S*)-**CoAux2** or Λ-(*S*)-**CoAux2** (136 mg, 0.05 mmol) was used to obtain complex Δ-**CoCat2** (114 mg, 83% yield) or Λ-**CoCat2** (111 mg, 81% yield) as a yellow solid. Note: *rac*-**CoCat2** was synthesized following the same procedure by using a 1:1 mixture of Δ-(*S*)-**CoAux2** and Λ-(*S*)-**CoAux2** as starting material.

^{1}H NMR (300 MHz, CD_3CN): δ 8.62 (d, J = 2.2 Hz, 1H), 8.48 – 8.41 (m, 3H), 8.37 (t, J = 7.9 Hz, 1H), 8.24 – 8.15 (m, 2H), 8.04 (d, J = 2.2 Hz, 1H), 7.75 (d, J = 8.7 Hz, 1H), 7.73 – 7.65 (m, 26H), 7.64 (s, 1H), 7.47 – 7.35 (m, 3H), 7.24 (d, J = 8.2 Hz, 1H), 7.20 (s, 2H), 7.15 (dd, J = 7.2, 2.1 Hz, 1H), 7.07 (d, J = 2.3 Hz, 1H), 7.03 (s, 1H), 6.88 (s, 1H), 6.83 (s, 2H), 6.70 (d, J = 1.9 Hz, 1H), 6.53 (d, J = 1.7 Hz, 1H), 2.87 (s, 3H), 2.35 (s, 6H), 2.27 (s, 6H), 2.19 (s, 3H), 1.66 (s, 3H), 1.05 (s, 3H), 0.56 (s, 3H). **^{13}C NMR** (126 MHz, CD_3CN): δ 170.79, 165.75, 162.59 (dd, J = 99.7, 49.9 Hz), 153.93, 153.54, 151.86, 151.57, 148.42, 146.14, 142.23, 141.41, 140.85, 139.95, 139.49, 137.88, 137.42, 137.26, 135.65, 135.34, 134.98, 133.19, 132.99, 132.60, 131.58, 131.55, 131.23, 131.02, 130.76, 130.63, 130.59, 130.31, 129.91 (q, J = 28.7 Hz), 128.13, 127.88, 126.58, 125.45 (d, J = 271.7 Hz), 121.99, 120.91, 118.76 – 118.59 (m), 115.30, 112.55, 112.03, 36.92, 21.46, 21.37, 20.88, 16.08, 15.34, 5.22. **^{19}F NMR** (282 MHz, CD_3CN): δ -63.25, -71.42, -73.93. **HRMS** (ESI, *m/z*): calcd. for

$C_{117}H_{75}B_2CoF_{48}N_9$ [M–PF6]$^+$: 2599.4942, found: 2599.4902. **IR** (film): *v* (cm^{-1}) 2925 (w), 1609 (w), 1501 (w), 1463 (w), 1355 (m), 1277 (s), 1123 (s), 1001 (w), 933 (w), 887 (w), 839 (w), 807 (w), 791 (w), 775 (w), 744 (w), 712 (w), 682 (w), 670 (w), 586 (w), 559 (w), 449 (w), 420 (w).

CD (CH_2Cl_2) for Δ-**CoCat2**: λ, nm (Δε, M^{-1} cm^{-1}), 446(+5.4), 395(-5.0), 349(-8.9), 313(+61.9), 288(-56.6).

CD (CH_2Cl_2): for Λ-**CoCat2**: λ, nm (Δε, M^{-1} cm^{-1}), 444(-5.5), 393(+4.6), 349(+10.1), 313(-56.4), 288(+58.6).

Synthesis of Δ- and Λ-CoCat3: Following the general procedure, Δ-(*S*)-**CoAux3** or Λ-(*S*)-**CoAux3** (140 mg, 0.05 mmol) was used to obtain complex Δ-**CoCat3** (115 mg, 82% yield) or Λ-**CoCat3** (117 mg, 83% yield) as a yellow solid. Note: *rac*-**CoCat3** was synthesized following the same procedure by using a 1:1 mixture of Δ-(*S*)-**CoAux3** and Λ-(*S*)-**CoAux3** as starting material.

^{1}**H NMR** (300 MHz, CD_3CN): δ 8.78 (dd, *J* = 8.7, 1.6 Hz, 1H), 8.69 (d, *J* = 2.2 Hz, 1H), 8.60 (s, 1H), 8.53 – 8.42 (m, 3H), 8.22 (t, *J* = 8.3 Hz, 1H), 8.08 (d, *J* = 2.2 Hz, 1H), 7.74 – 7.64 (m, 24H), 7.61 (d, *J* = 8.3 Hz, 1H), 7.49 – 7.36 (m, 3H), 7.30 (d, *J* = 8.2 Hz, 1H), 7.22 – 7.15 (m, 3H), 7.10 (d, *J* = 2.3 Hz, 1H), 7.04 (s, 1H), 6.91 (s, 1H), 6.87 (s, 2H), 6.69 (s, 1H), 6.58 (s, 1H), 2.86 (s, 3H), 2.31 (d, *J* = 7.8 Hz, 12H), 2.19 (s, 3H), 1.68 (s, 3H), 1.05 (s, 3H), 0.58 (s, 3H). ^{13}**C NMR** (151 MHz, CD_3CN): δ 169.66, 164.60, δ 162.63 (dd, J = 99.7, 49.8 Hz), 154.90, 154.73, 151.78, 151.59, 149.79 (q, *J* = 4.1 Hz), 148.73, 144.94 – 144.62 (m), 142.52, 142.52, 141.42, 141.23, 140.09, 139.77, 137.81, 137.56, 136.97, 135.68, 135.35, 134.97, 133.12, 132.89, 132.82, 131.82, 131.70, 131.29, 131.23, 130.83, 130.76, 130.53, 129.95 (q, J = 31.7 Hz), 127.91, 127.90 (q, *J* = 35.8 Hz), 127.65, 125.49 (q, *J* = 271.4 Hz), 122.82, 122.79, 122.77 (d, *J* = 273.3 Hz), 121.21, 118.80, 118.76 – 118.59 (m), 116.57, 112.95, 112.48, 37.39, 21.43, 21.40, 20.91, 16.06, 15.38, 5.66. ^{19}**F NMR** (282 MHz, CD_3CN): δ -61.50, -63.25, -71.41, -73.91. **HRMS** (ESI, *m/z*) calcd. for $C_{118}H_{74}B_2CoF_{51}N_9$ [M–PF_6]$^+$: 2667.4816, found: 2667.4736. **IR** (film): *v* (cm^{-1}) 3146 (w), 1629 (w), 1609 (w), 1503 (w), 1450 (w), 1354 (m), 1329

(w), 1313 (w), 1276 (s), 1121 (s), 1001 (w), 932 (w), 887 (w), 838 (m), 807 (w), 792 (w), 744 (w), 712 (w), 694 (w), 682 (m), 670 (w), 559 (w), 449 (w).

CD (CH_2Cl_2) for Δ-**CoCat3**: λ, nm (Δε, M^{-1} cm^{-1}), 451(+5.9), 395(-4.9), 325(+38.7), 294(-43.8).

CD (CH_2Cl_2) for Λ-**CoCat3**: λ, nm (Δε, M^{-1} cm^{-1}), 451(-5.7), 395(+5.2), 325(-36.6), 294(+40.7).

2.8 Synthesis of the Non-Racemic Cobalt Complex Δ-CoCat4

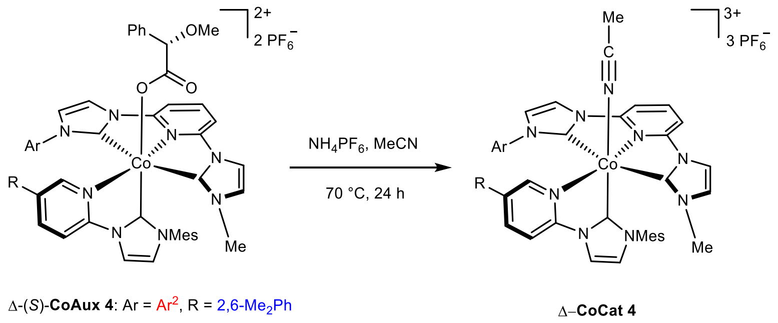

To a solution of Δ-(*S*)-**CoAux4** (56 mg, 0.04 mmol) in CH_3CN (3 mL) was added NH_4PF_6 (0.60 mmol, 98 mg) and the tube was sealed. The resulting mixture was stirred at 70 ºC for 24 h. The reaction mixture was evaporated to dryness, and washed first with CH_2Cl_2 and then H_2O for several times to give a yellow solid (35 mg, 63 % yield). Note that Δ-**CoCat4** contains three PF_6 counteranions for single crystal X-ray diffraction analysis.

^{1}H NMR (300 MHz, CD_3CN): δ 8.55 – 8.50 (m, 3H), 8.45 (dd, *J* = 8.6, 1.9 Hz, 1H), 8.30 – 8.19 (m, 2H), 8.12 (d, *J* = 1.7 Hz, 1H), 8.04 (d, *J* = 2.2 Hz, 1H), 7.69 (d, *J* = 8.2 Hz, 1H), 7.55 (t, *J* = 7.8 Hz, 1H), 7.52 – 7.45 (m, 2H), 7.39 (d, *J* = 2.2 Hz, 2H), 7.26 (d, *J* = 8.2 Hz, 1H), 7.21 – 7.12 (m, 3H), 6.91 (d, *J* = 4.1 Hz, 2H), 6.85 – 6.63 (m, 3H), 6.56 (d, *J* = 7.7 Hz, 3H), 2.84 (s, 3H), 2.50 (s, 3H), 2.28 (s, 6H), 2.23 (s, 3H), 2.19 (s, 1H), 1.97 (s, 4H), 1.68 (s, 3H), 1.02 (s, 3H), 0.56 (s, 3H). **^{13}C NMR** (126 MHz, CD_3CN): δ 169.85, 163.89, 153.07, 152.96, 151.60, 151.38, 150.65, 148.73, 148.57, 142.29, 141.55, 140.02, 139.80, 137.99, 137.83, 137.59, 137.31, 136.65, 135.21, 134.85, 133.56, 133.15,

133.09, 132.94, 131.65, 131.64, 131.32, 131.10, 130.93, 130.89, 130.78, 130.73, 130.30, 127.77, 127.34, 121.92, 120.91, 118.93, 114.78, 112.67, 112.24, 37.09, 22.97, 22.61, 21.47, 21.09, 20.86, 16.16, 15.24, 5.14. **^{19}F NMR** (282 MHz, CD_3CN): δ -71.50, -74.00. **HRMS** (ESI, *m/z*): calcd. for $C_{61}H_{59}CoF_{12}N_9P_2$ [M–PF_6]$^+$: 1266.3478, found: 1266.3486. **IR** (film): *v* (cm^{-1}) 3641 (w), 3408 (w), 3166 (w), 2925 (w), 1625 (w), 1603 (w), 1542 (w), 1501 (w), 1452 (w), 1355 (w), 1279 (w), 1127 (w), 841 (s), 779 (w), 708 (w), 670 (w), 558 (w).

CD (CH_2Cl_2) for Δ-**CoCat4**: λ, nm (Δε, M^{-1} cm^{-1}), 462(+1.9), 413(-5.7), 351(-5.5), 306(+50.5), 271(+99.1).

2.9 Synthesis of Complex *rac*-[CoCat1](NTf_2)$_3$

A mixture of *rac*-**CoBr1** (162 mg, 0.15 mmol), (*S*)-methoxyphenylacetic acid (200 mg, 1.20 mmol) and K_2CO_3 (83 mg, 0.60 mmol) in 16 mL mixed solvent (CH_3CN/EtOH, v/v = 3/1) was heated at 65 °C for 18 h under air. The reaction mixture was concentrated to dryness, dissolved in CH_2Cl_2 and washed by H_2O. The organic layer was dried over $MgSO_4$, filtered and evaporated under reduced pressure. The residue was subjected to flash silica gel chromatography (CH_2Cl_2/CH_3OH/sat. solution of KPF_6 in MeCN = 100:10:1) and collected the yellow fraction. The obtained yellow fraction was dried and dissolved in 5 mL CH_3CN in a flask. After that, NH_4PF_6 (1.50 mmol, 245 mg) was added to the solution and the resulting mixture was stirred at 70 °C for 24 h. The reaction mixture was evaporated to dryness. The residue was washed with H_2O and CH_2Cl_2 for several times. The precipitate was dissolved in 10 mL of CH_3CN and filtered. The filtrate was then concentrated to dryness, yielding a yellow solid of *rac*-**[CoCat1](PF_6)$_3$** (yield over two steps: 99 mg, 55%).

^{1}H NMR (300 MHz, CD_3CN): δ 8.52 (d, *J* = 2.1 Hz, 1H), 8.42 (d, *J* = 2.2 Hz, 1H), 8.35 (t, *J* = 8.3 Hz, 1H), 8.20 (p, *J* = 3.6, 3.2 Hz, 3H), 8.01 (d, *J* = 8.6 Hz, 1H), 7.79 – 7.65 (m, 2H), 7.53 (d, *J* = 8.3 Hz, 1H), 7.46 (d, *J* = 2.1 Hz, 1H), 7.38 – 7.23 (m, 4H), 6.97 (d, *J* = 8.7 Hz, 1H), 6.81 (d, *J* = 5.6 Hz, 2H), 2.93 (s, 3H), 2.52 – 2.38 (m, 1H), 2.29 (s, 3H), 2.20 (s, 3H), 1.74 – 1.58 (m, 1H), 1.52 (s, 3H),

1.37 (d, $J = 6.8$ Hz, 3H), 1.22 (s, 3H), 1.06 (t, $J = 7.2$ Hz, 6H), 0.87 (d, $J = 6.7$ Hz, 3H). **^{13}C NMR** (126 MHz, CD_3CN): δ 170.89, 166.40, 154.44, 153.16, 152.30, 152.24, 151.55, 148.77, 146.78, 145.78, 143.83, 142.61, 135.63, 134.80, 132.86, 132.80, 132.73, 132.70, 131.42, 131.03, 130.99, 130.92, 130.69, 126.42, 125.93, 125.14, 121.92, 121.15, 120.93, 115.40, 112.59, 112.36, 36.94, 29.55, 29.02, 26.88, 25.94, 22.45, 22.42, 20.97, 16.94, 16.24, 5.87. **^{19}F NMR** (282 MHz, CD_3CN): δ -71.24, -73.75. **HRMS** (ESI, *m/z*) calcd. for $C_{43}H_{47}CoF_{12}N_9P_2$ [M–PF_6]$^+$: 1038.2565, found: 1038.2548. **IR** (film): *v* (cm^{-1}) 3665 (w), 3146 (w), 2974 (w), 2327 (w), 1635 (w), 1593 (w), 1501 (w), 1463 (w), 1429 (w), 1390 (w), 1354 (w), 1306 (w), 1288 (w), 1198 (w), 1146 (w), 1060 (w), 837 (s), 797 (w), 779 (w), 748 (w), 696 (w), 679 (w), 558 (m), 438 (w).

rac-**[CoCat1](PF_6)$_3$** (35.5 mg, 0.03 mmol) and $NaNTf_2$ (182.0 mg, 0.60 mmol) were dissolved in CH_2Cl_2/MeCN (2:1, 3 mL) and stirred at 40 °C in a sealed tube for 20 h under air. After the reaction, the reaction mixture was concentrated under reduced pressure, and the residue was treated with 10 ml water and 5 ml CH_2Cl_2 and stirred at room temperature for 30 min. Then, the organic phase was collected and the aqueous phase was washed with CH_2Cl_2. The combined organic layers were filtered, and concentrated under reduced pressure to give *rac*-**[CoCat1](NTf_2)$_3$** (40 mg, 83% yield) as a yellow solid.

^{1}H NMR (300 MHz, CD_3CN): δ 8.53 (d, $J = 2.2$ Hz, 1H), 8.43 (d, $J = 2.3$ Hz, 1H), 8.35 (t, $J = 8.3$ Hz, 1H), 8.24 – 8.17 (m, 3H), 8.02 (d, $J = 8.6$ Hz, 1H), 7.75 (d, $J = 2.2$ Hz, 1H), 7.72 (d, $J = 8.2$ Hz, 1H), 7.54 (d, $J = 8.3$ Hz, 1H), 7.46 (d, $J = 2.2$ Hz, 1H), 7.40 – 7.21 (m, 4H), 6.97 (d, $J = 7.4$ Hz, 1H), 6.81 (d, $J = 5.7$ Hz, 2H), 2.93 (s, 3H), 2.50 – 2.41 (m, 1H), 2.29 (s, 3H), 2.20 (s, 3H), 1.72 – 1.58 (m, 1H), 1.51 (s, 3H), 1.37 (d, $J = 6.8$ Hz, 3H), 1.22 (s, 3H), 1.06 (dd, $J = 8.8, 6.7$ Hz, 6H), 0.87 (d, $J = 6.7$ Hz, 3H). **^{13}C NMR** (126 MHz, CD_3CN): δ 170.85, 166.40, 154.34, 153.05, 152.33, 152.24, 151.58, 148.88, 146.70, 145.83, 143.80, 142.60, 135.61, 134.81, 132.86, 132.81, 132.76, 132.62, 131.40, 131.01, 130.95, 130.78, 126.37, 125.92, 125.15, 123.43 (q, J = 320.7 Hz), 121.97, 121.13, 120.92, 115.46, 112.56, 112.29, 37.00, 29.55, 29.04, 26.84, 25.91, 22.45, 20.97, 16.94, 16.24, 6.11. **^{19}F NMR**

(282 MHz, CD_3CN): δ -80.12. **HRMS** (ESI, *m/z*): calcd. $C_{47}H_{47}CoF_{12}N_{11}O_8S_4$ for $[M–NTf_2]^+$: 1308.1627, found: 1308.1611. **IR** (film): *v* (cm^{-1}) 3567 (w), 3140 (w), 2973 (w), 1635 (w), 1593 (w), 1500 (w), 1463 (w), 1346 (m), 1281 (w), 1190 (s), 1135 (m), 1059 (m), 842 (w), 794 (w), 779 (w), 764 (w), 743 (w), 696 (w), 654 (w), 615 (m), 574 (w), 515 (m), 410 (w).

2.10 Synthesis of Complex Λ-[CoCat3](BArF)$_3$

Λ-CoCat3 (28.1 mg, 0.01 mmol) and NaBArF (8.9 mg, 0.01 mmol) were dissolved in 4 mL solvent (CH_2Cl_2/CH_3CN, v/v = 3/1) and stirred at room temperature overnight under air. After that, the reaction mixture was evaporated to dryness, then dissolved in 15 mL CH_2Cl_2 and washed with H_2O (3 x 8 mL). The organic layer was dried over $MgSO_4$, filtered and evaporated under reduced pressure. The obtained residue was washed with a small amount of Et_2O. The supernatant solution was decanted and all the volatiles of the remaining solid were removed in vacuo to give Λ-**[CoCat3]**(BArF)$_3$ (27.6 mg, 79% yield**)** as a yellow solid.

^{1}H NMR (300 MHz, CD_3CN): δ 8.77 (dd, *J* = 8.8, 1.6 Hz, 1H), 8.69 (d, *J* = 2.2 Hz, 1H), 8.59 (s, 1H), 8.50 (d, *J* = 2.3 Hz, 1H), 8.46 (d, *J* = 8.8 Hz, 1H), 8.43 (d, *J* = 2.2 Hz, 1H), 8.20 (t, *J* = 8.3 Hz, 1H), 8.07 (d, *J* = 2.2 Hz, 1H), 7.74 – 7.63 (m, 36H), 7.60 (d, *J* = 8.1 Hz, 1H), 7.50 – 7.36 (m, 3H), 7.28 (d, *J* = 8.2 Hz, 1H), 7.19 (dd, *J* = 7.5, 1.7 Hz, 1H), 7.15 (s, 2H), 7.09 (d, *J* = 2.3 Hz, 1H), 7.03 (s, 1H), 6.91 (s, 1H), 6.86 (s, 2H), 6.68 (s, 1H), 6.57 (s, 1H), 2.85 (s, 3H), 2.31 (d, *J* = 7.9 Hz, 12H), 2.19 (s, 3H), 1.67 (s, 3H), 1.04 (s, 3H), 0.57 (s, 3H). **^{19}F NMR** (282 MHz, CD_3CN): δ -61.50, -63.25. **HRMS** (ESI, *m/z*): calcd. for $C_{118}H_{74}B_2CoF_{51}N_9$ [M–BArF]$^+$: 2667.4816, found: 2666.4771. **IR** (film): *v* (cm^{-1}) 2927 (w), 1628 (w), 1609 (w), 1502 (w), 1450 (w), 1354 (m), 1328 (w), 1311 (w), 1276 (s), 1120 (s), 1001 (w), 931 (w), 887 (w), 856 (w), 838 (m), 808 (w), 793 (w), 742 (w), 711 (m), 682 (m), 670 (w), 625 (w), 581 (w), 507 (w), 449 (w).

CD (CH_2Cl_2) for Λ-**[CoCat3]**(BArF)$_3$: λ, nm (Δε, M^{-1} cm^{-1}), 454(-4.5), 401(+4.2), 326(-21.2), 299(-35.7).

3. Determination of the Absolute Configuration of Cobalt Complexes

The absolute configurations of cobalt complexes were determined by comparing their CD spectra with Δ-**CoCat4**, whose configuration was assigned by X-ray diffraction.

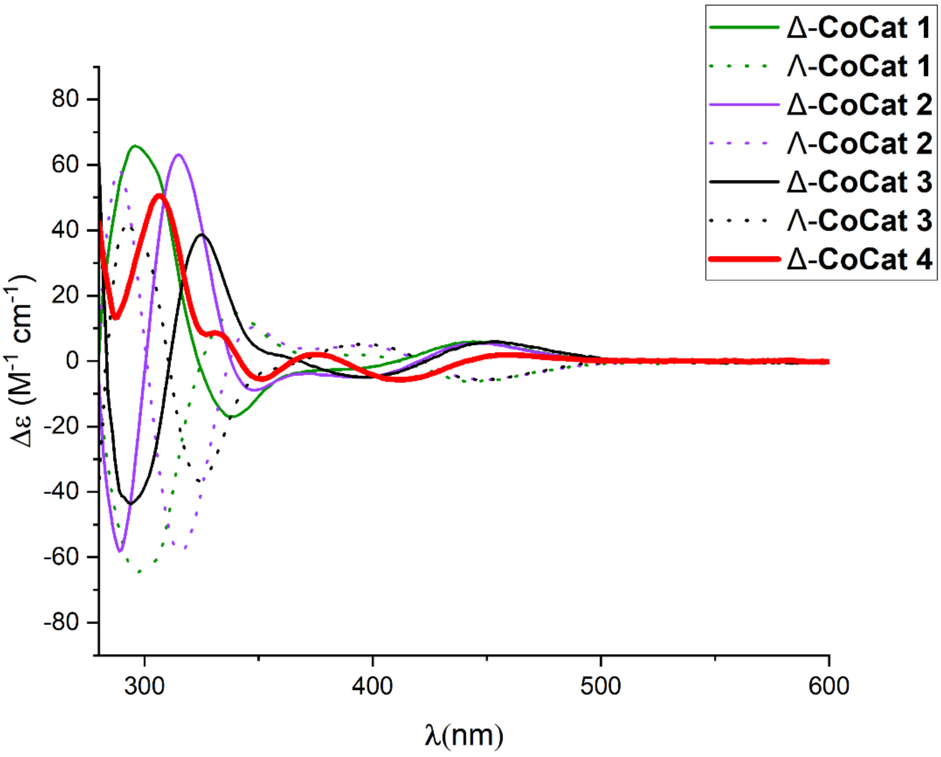

Supplementary Fig. 1. Comparison the CD spectra of cobalt complexes (recorded in CH_2Cl_2, 1.0 mM).

4. Determination of the Enantiomeric Purities of Cobalt Complexes

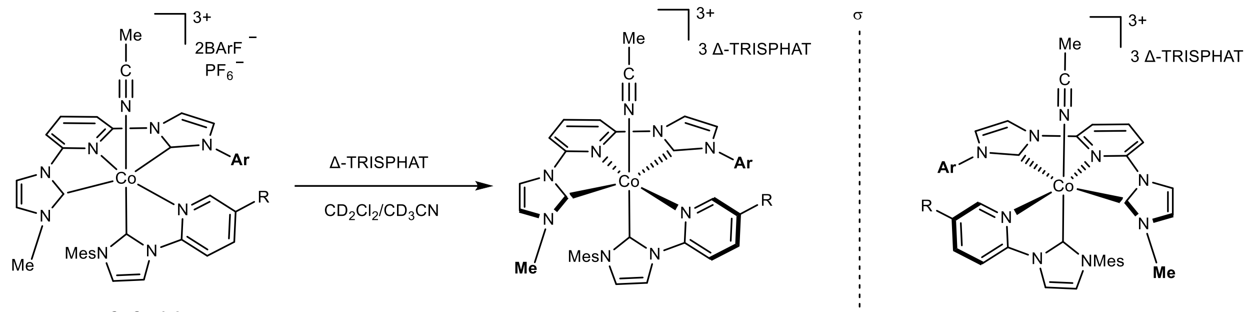

rac-**CoCat1-3** ΛΔ- **CoTRIP1-3** ΔΔ-**CoTRIP1-3**

Supplementary Fig. 2. Interaction of *rac*-**CoCat1-3** with Δ-TRISPHAT in CD_2Cl_2/CD_3CN.

Method: The chiral counterion Δ-TRISPHAT ([tetrabutylammonium] [Δ-tris(tetrachlor-1,2-benzoldiolato)-phosphate(V)], ee>98.5%) interacts with chiral cobalt cations to form two diastereomers: Λ/Δ-**CoTRIP1-3** and Δ/Δ-**CoTRIP1-3** (**Supplementary Fig. 2**). These two diastereomers can be distinguishable by their ^{1}H NMR spectra, enabling the calculation of the enantiomeric purities of **CoCat1-3** through integration of their respective signals.

4.1 Enantiomeric Purity of CoCat1

General Procedure: *rac*-**CoCat1,** Λ-**CoCat1** or Δ-**CoCat1** (0.0019 mmol, 5 mg) were dissolved in a mixture of CD_2Cl_2/CD_3CN (15:1; 640 μL). The chiral shift reagent Δ-TRISPHAT (0.0076 mmol, 7.7 mg; 4 equiv.) was then added. The enantiomeric excess (ee) of the catalyst was analysed by ^{1}H NMR spectroscopy.

Results: As shown in **Supplementary Fig. 3**, the mixture of 4 equiv. of Δ-TRISPHAT and *rac*-**CoCat1** showed two sets of peaks at 8.53 and 8.74 ppm. These peaks appeared in a 1:1 ratio (**Supplementary Fig. 3**, b), corresponding to the signals of Δ/Δ-**CoTRIP1** and Λ/Δ-**CoTRIP1**. In contrast, the ^{1}H-NMR spectra of Δ-**CoCat1** or Λ-**CoCat1** with 4 equivalents of Δ-TRISPHAT showed only a single peak at 8.53 or 8.74 ppm, respectively (**Supplementary Fig. 3**, c and d). These results allowed us to determine the enantiomeric ratios of Δ-**CoCat1** and Λ-**CoCat1**, which were calculated to be ≥ 98:2 e.r.

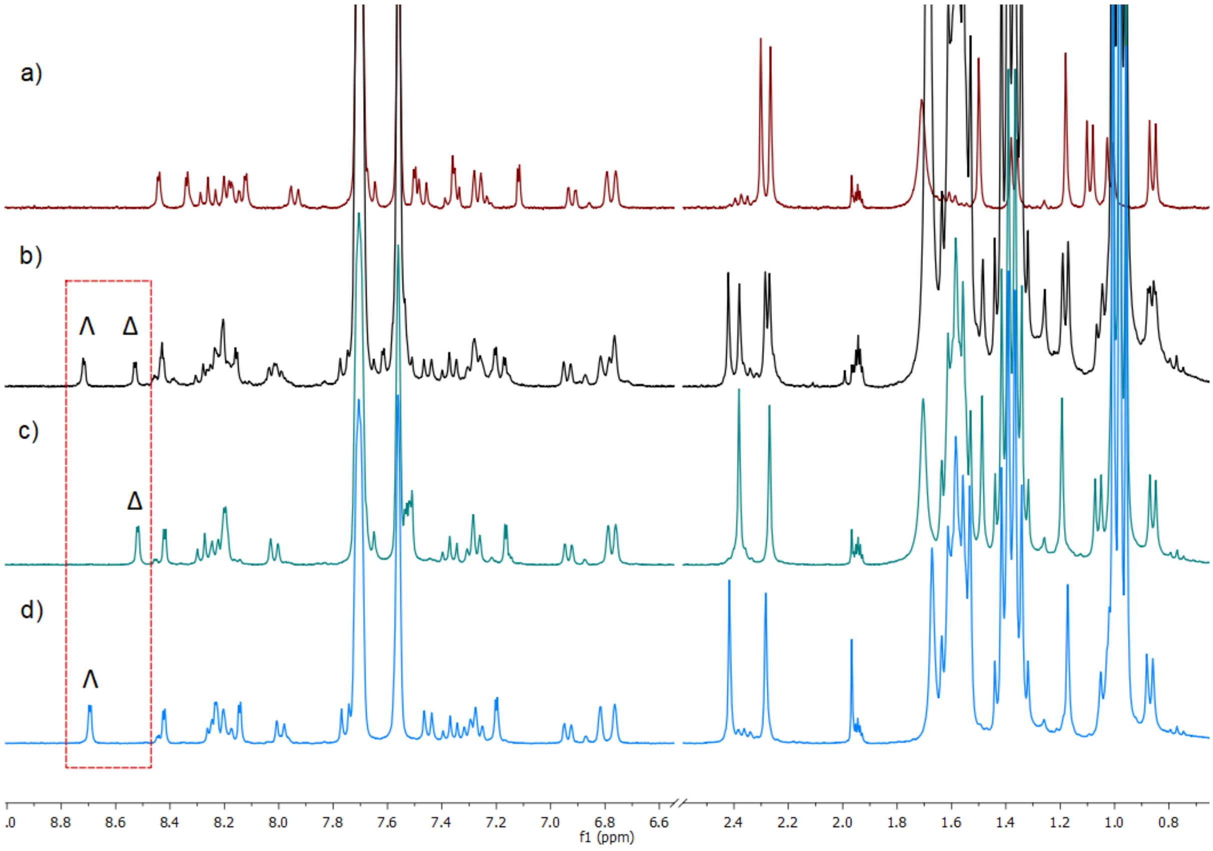

Supplementary Fig. 3. ^{1}H NMR (300 MHz, 298 K) spectra recorded in CD_2Cl_2/CD_3CN. a) *rac*-**CoCat1**. b) *rac*-**CoCat1** with 4 equivalents of Δ-TRISPHAT. c) Δ-**CoCat1** with 4 equivalents of Δ-TRISPHAT. d) Λ- **CoCat1** with 4 equivalents of Δ-TRISPHAT.

4.2 Enantiomeric Purity of CoCat2

General Procedure: *rac*-**CoCat2**, Λ-**CoCat2** or Δ-**CoCat2** (0.0022 mmol, 6 mg) were dissolved in a mixture of CD_2Cl_2/CD_3CN (15:1; 640 μL). The chiral shift reagent Δ-TRISPHAT (0.0088 mmol, 8.9 mg; 4 equiv.) was then added. The enantiomeric excess (ee) of the catalyst was analysed by ^{1}H NMR spectroscopy.

Result: As shown in **Supplementary Fig. 4**, the ^{1}H NMR spectrum of *rac*-**CoCat2**, with the addition of 4 equiv. of Δ-TRISPHAT showed two set of peaks at 8.57 and 8.74 ppm. These two sets of peaks were observed in a 1:1 ratio (**Supplementary Fig. 4**, b), corresponding to the signals of ΔΔ-**CoTRIP2** and ΛΔ-**CoTRIP2**. In contrast, the ^{1}H NMR spectra of Δ-**CoCat2** or Λ-**CoCat2** with addition of 4

equivalents of Δ-TRISPHAT showed only a single set of peaks at 8.57 or 8.74 ppm (**Supplementary Fig. 4**, c or d). These data were used to calculate the enantiomeric ratios of Δ-**CoCat2** or Λ-**CoCat2** complexes, which were determined to be ≥ 98:2 e.r.

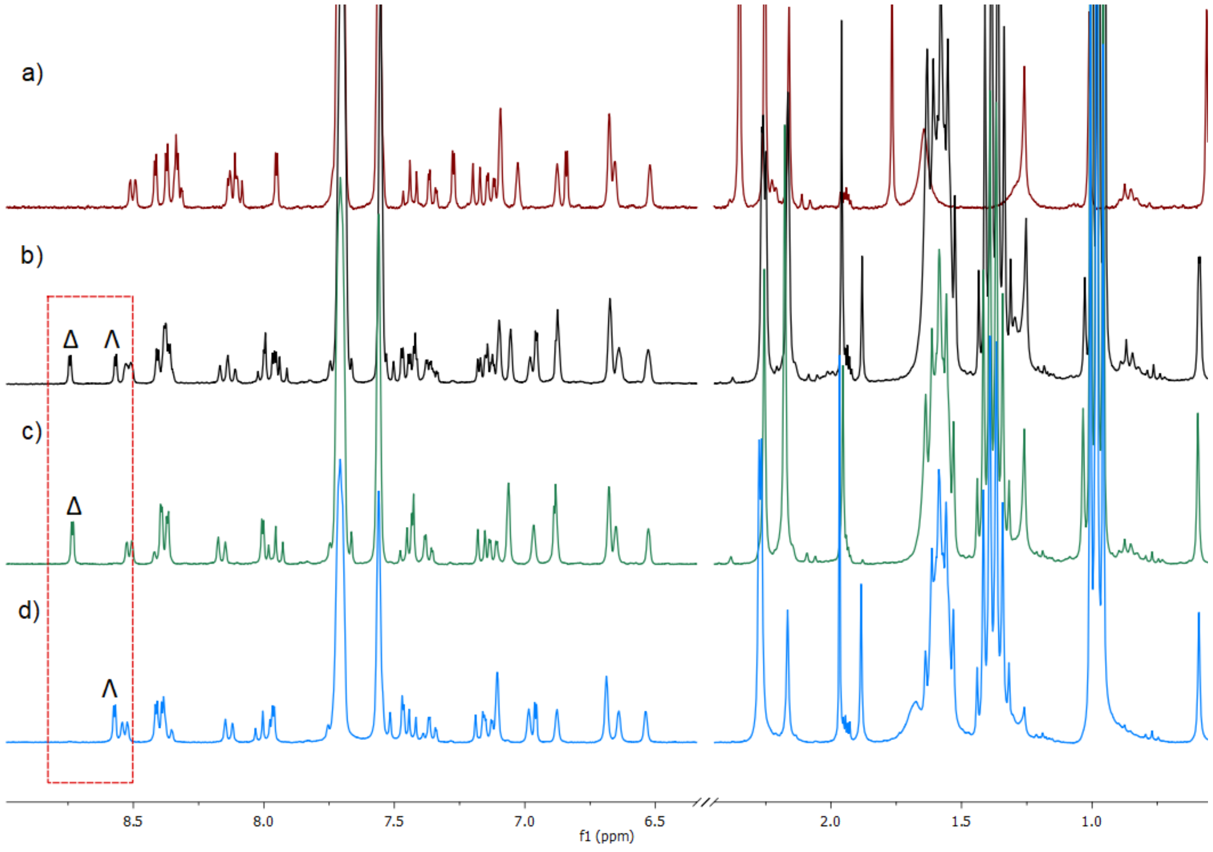

Supplementary Fig. 4. ^{1}H NMR (300 MHz, 298 K) spectra recorded in CD_2Cl_2/CD_3CN. a) *rac*-**CoCat2**. b) *rac*-**CoCat2** with 4 equivalents of Δ-TRISPHAT. c) Δ-**CoCat2** with 4 equivalents of Δ-TRISPHAT. d) Λ-**CoCat2** with 4 equivalents of Δ-TRISPHAT.

4.3 Enantiomeric Purity of CoCat3

General Procedure: *rac*-**CoCat3**, Λ-**CoCat3** or Δ-**CoCat3** (0.0021 mmol, 6 mg) were dissolved in a mixture of CD_2Cl_2/CD_3CN (15:1; 640 μL). The chiral shift reagent Δ-TRISPHAT (0.0084 mmol, 8.6 mg; 4 equiv.) was then added. The enantiomeric excess (ee) of the catalyst was analysed by ^{1}H NMR spectroscopy.

Result: As shown in **Supplementary Fig. 5**, the ^{1}H NMR spectrum of *rac*-**CoCat3**, with the addition of 4 equiv. of Δ-TRISPHAT, showed two set of peaks show at 7.06 and 7.10 ppm. These two sets of peaks were observed in a 1:1 ratio (**Supplementary Fig. 5**, b), corresponding to the signals of ΔΔ-**CoTRIP3** and ΛΔ-**CoTRIP3**. In contrast, the ^{1}H-NMR spectra of Δ-**CoCat3** with addition of 4 equivalents of Δ-TRISPHAT showed only a single set of peaks at 7.06 ppm (**Supplementary Fig. 5**, c). These data were used to calculate the enantiomeric ratios of Δ-**CoCat3** or Λ-**CoCat3** complexes, which were determined to be ≥ 98:2 e.r. The ^{1}H-NMR spectra of Λ-**CoCat3** with addition of 4 equivalents of Δ-TRISPHAT displayed two distinct peaks at 7.06 ppm (minor) and 7.10 ppm (major) (**Supplementary Fig. 5**, d), with an integration ratio of 2.7:100. These data were used to calculate the enantiomeric ratios of Δ-**CoCat3** or Λ-**CoCat3** complexes, which were determined to be = 97.4:2.6 e.r.

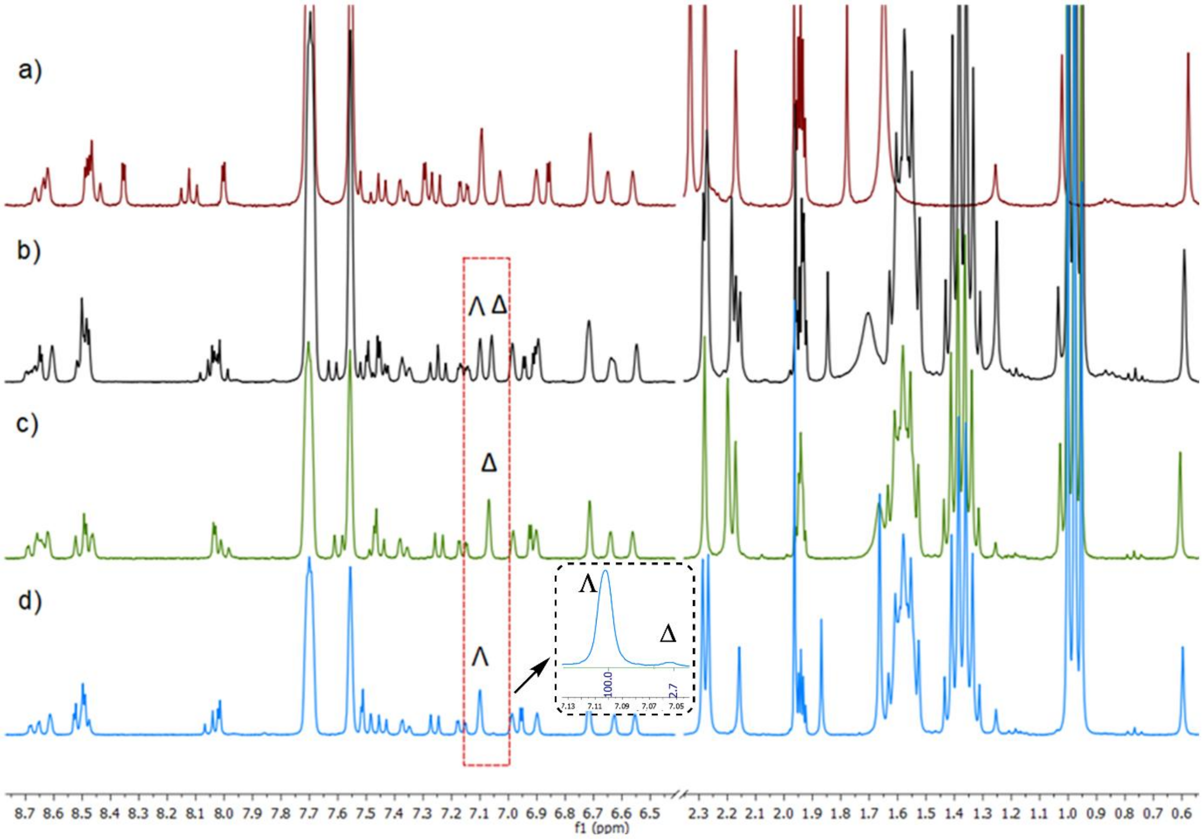

Supplementary Fig. 5. ^{1}H NMR (300 MHz, 298 K) spectra recorded in CD_2Cl_2/CD_3CN. a) *rac*-**CoCat3**. b) *rac*-**CoCat3** with 4 equivalents of Δ-TRISPHAT. c) Δ-**CoCat3** with 4 equivalents of Δ-TRISPHAT. d) Λ- **CoCat3** with 4 equivalents of Δ-TRISPHAT.

5. Stability Studies of Cobalt Complexes

5.1 Stability Studies of CoCat1 in CH_3CN at Elevated Temperature

5.1.1 Constitutional Stability

General Procedure: *rac*-**CoCat1** (0.0038 mmol, 10 mg) was dissolved in 2 mL CH_3CN and stirred at 70 °C under air for 20 h. After that, the solution was directly concentrated to dryness under reduced pressure. The residual materials were dissolved in CD_3CN and analyzed by ^1H NMR.

Result: As shown in **Supplementary Fig. 6**, the ^1H NMR signal of *rac*-**CoCat1** did not change after stirred at 70 °C in CH_3CN for 20 hours. These experiments indicate that *rac*-**CoCat1** is stable in CH_3CN at 70 °C.

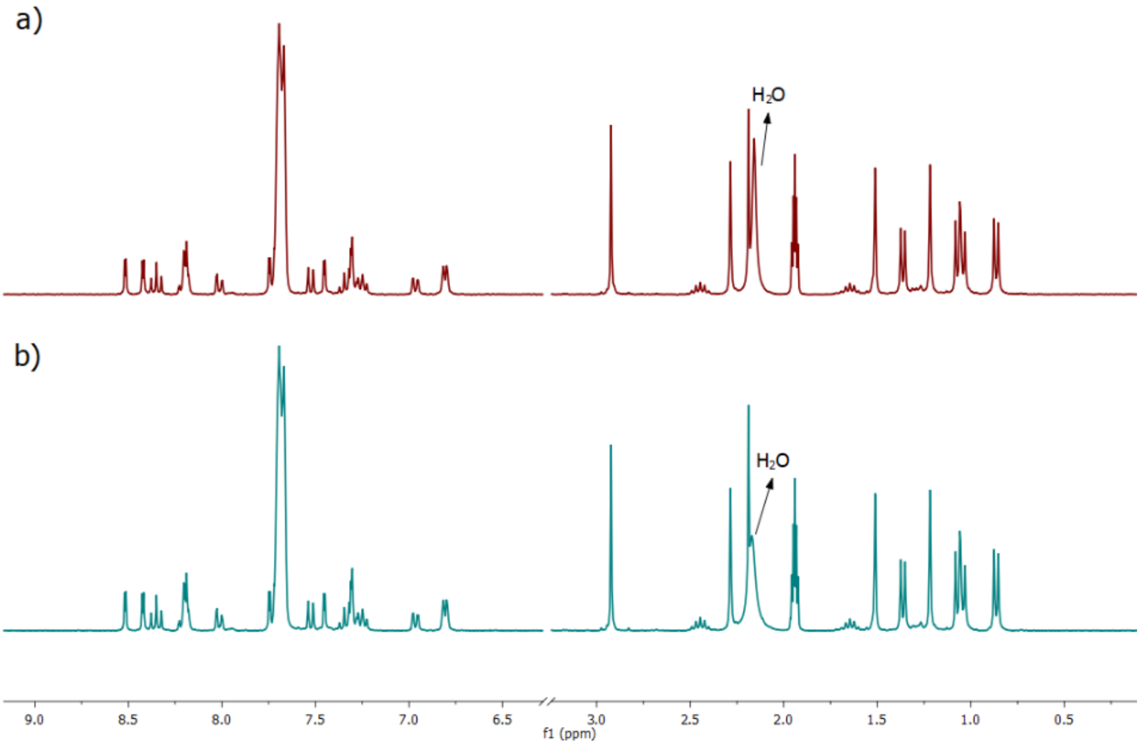

Supplementary Fig. 6. ^1H NMR (300 MHz, 298 K) spectra of *rac*-**CoCat1** recorded in CD_3CN. a) Right after dissolution. b) After stirring at 70°C for 20 h.

5.1.2 Configurational Stability

General Procedure: **Δ-CoCat1** (0.0019 mmol, 5 mg) was dissolved in 1 mL CH_3CN and stirred at 70 °C under air for 20 h. After that, the solution was directly concentrated to dryness under reduced pressure. The residue was dissolved in a mixture of CD_2Cl_2/CD3CN (15:1; 640 μL), and 4 equivalents of Δ-TRISPHAT (0.0076 mmol, 7.7 mg) were added as a chiral shift reagent. The ee value of catalyst was analysed by ^{1}H-NMR.

Results: As shown in **Supplementary Fig. 7**, the ^{1}H NMR spectrum of **Δ-CoCat1** (after stirring at 70 °C for 20 h) with addition of Δ-TRISPHAT only showed the peak at 8.53 ppm (**Figure S7**, c). This observation indicates that only the Δ/Δ-**CoTRIP1** complex was present and no racemization occurred during the heating process.

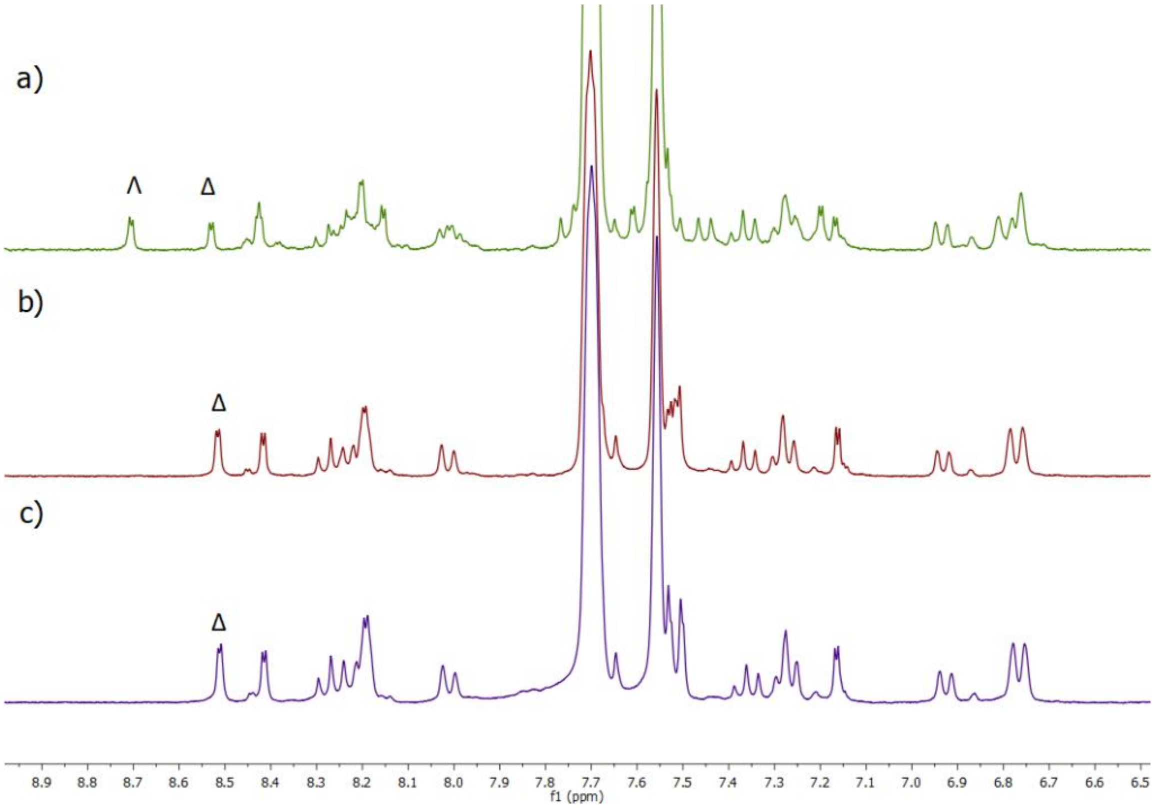

Supplementary Fig. 7. ^{1}H NMR (300 MHz, 298 K) spectra of **CoCat1 with** Δ-TRISPHAT recorded in CD_2Cl_2/CD_3CN. a) Complex *rac*-**CoCat1** with addition of Δ-TRISPHAT. b) Complex **Δ-CoCat1** with addition of Δ-TRISPHAT. c) **Δ-CoCat1** (after heating) with addition of Δ-TRISPHAT.

5.2 Stability Studies of Cobalt Complexes in CH_3CN under Irradiation

5.2.1 Constitutional Stability

General Procedure: *rac*-**CoCat1** (0.0038 mmol, 10 mg) was dissolved in 0.6 mL CD_3CN in an NMR tube and irradiated under 24W blue LEDs (positioned approximately 15 cm from the light source) at room temperature for 72 h. After that, ^1H NMR of the solutions were recorded.

Result: As shown in **Supplementary Fig. 8**, the ^1H NMR spectrum of *rac*-**CoCat1** after irradiation reveals a decrease in the coordinated CH_3CN peaks at 2.19 ppm, accompanied by an increase in the dissociated CH_3CN peak at 1.97 ppm. Notably, no significant changes were observed in other parts of the spectrum. This indicates that ligand exchange between CH_3CN and CD_3CN occurs during irradiation, while the complex remains intact without decomposition or electron transfer with BArF anions during this process.

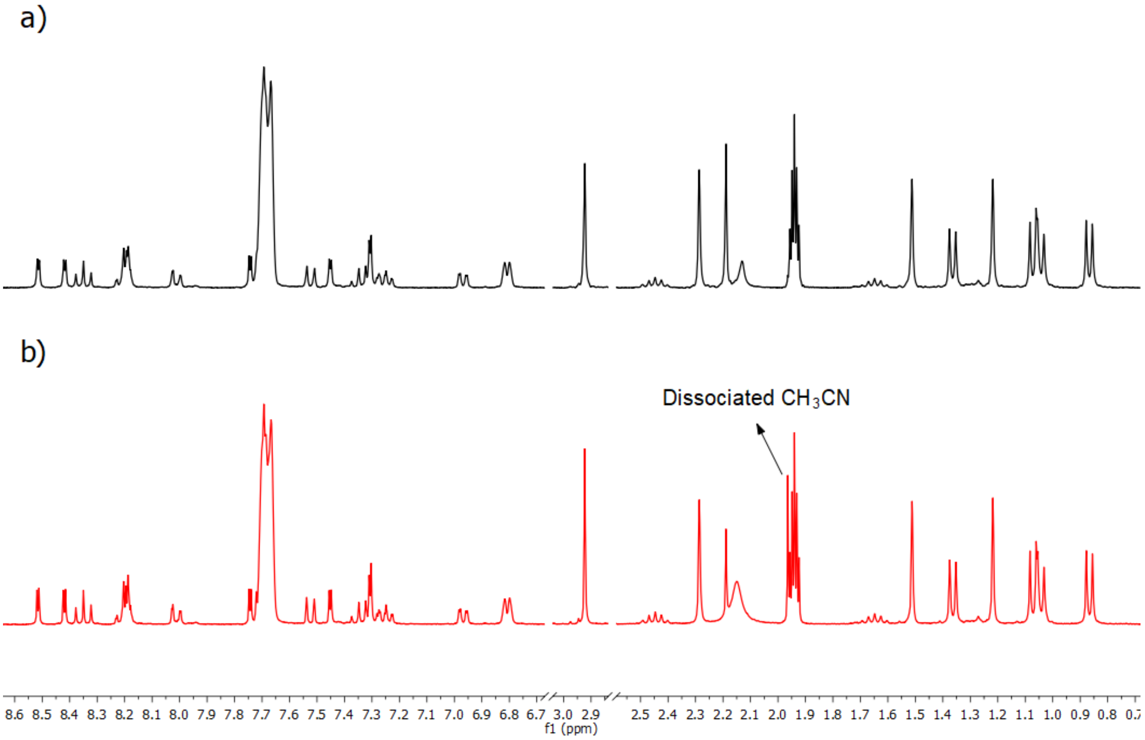

Supplementary Fig. 8. ^1H NMR (300 MHz, 298 K) spectra of Δ-**CoCat1** recorded in CD_3CN. a) Before irradiation. b) After irradiation for 72 h.

5.2.2 Configurational Stability

Methods: Λ-**CoCat3** (0.0021 mmol, 6 mg) was dissolved in 1 mL CH_3CN and exposed to 24 W blue LEDs irradiation (positioned approximately 15 cm from the light source) under air at room temperature for 8 h. After that, the solution was directly concentrated to dryness under reduced pressure. The residue was dissolved in a mixture of CD_2Cl_2/CD_3CN (15:1; 640 μL). A chiral shift reagent, Δ-TRISPHAT (0.0084 mmol, 8.6 mg; 4 equiv.), was then added. The enantiomeric excess (ee) of the catalyst was analyzed by ^1H NMR spectroscopy.

Results: As shown in **Supplementary Fig. 9**, after 8 hours irradiation, the ^1H NMR signal of Λ-**CoCat3** with addition of Δ-TRISPHAT showed two set of peaks at 7.10 and 7.05 ppm with the same e.r. value (97.4:2.6) compared to the sample before irradiation. This observation indicates that no racemization occurred during the visible light induced ligand exchange process.

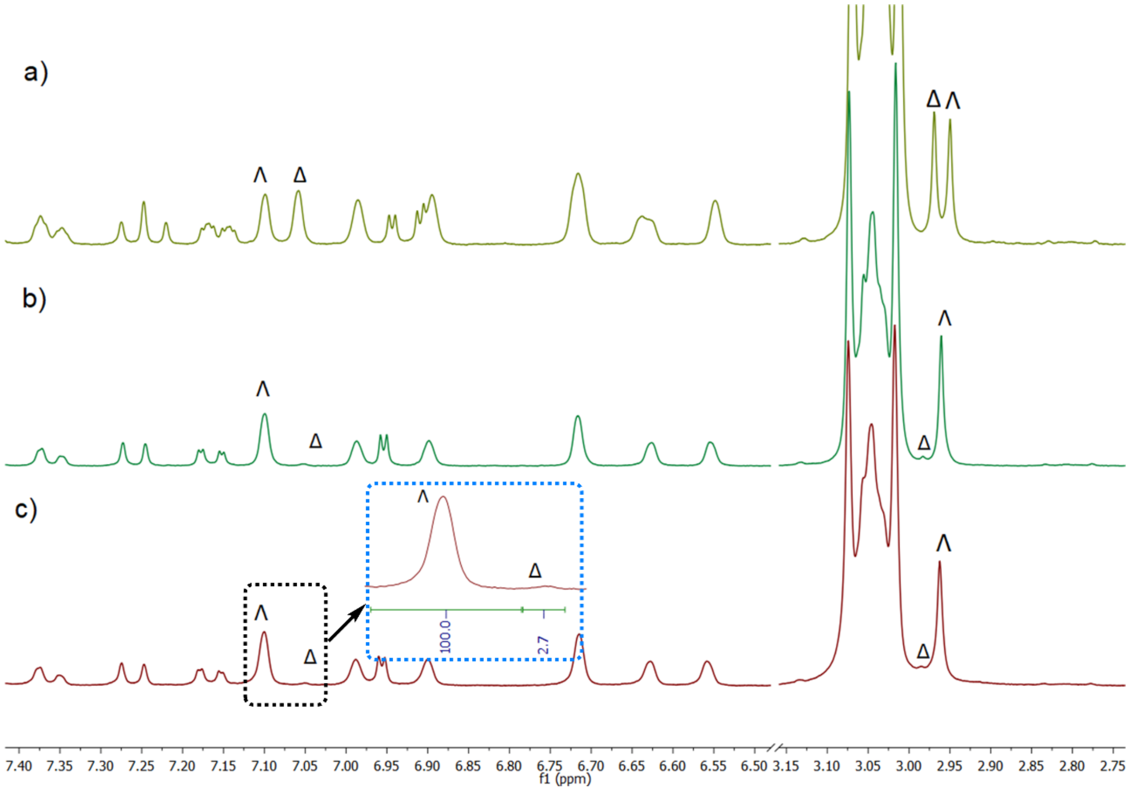

Supplementary Fig. 9. ^1H NMR (300 MHz, 298 K) spectra of **CoCat3** with Δ-TRISPHAT recorded in CD_2Cl_2/CD_3CN. a) *rac*-**CoCat3** with addition of Δ-TRISPHAT. b) Λ-**CoCat3** with addition of Δ-TRISPHAT. c) Λ-**CoCat3** (after irradiation) with addition of Δ-TRISPHAT.

6. Determination of Ligand Dissociation Rate Constants

6.1 Rate Constant under Light Irradiation

Method: *rac*-**CoCat1** (0.0076 mmol, 20 mg) and 40 μL CD_3CN (0.76 mmol) were dissolved in CD_2Cl_2 (resulting in CD_2Cl_2/CD_3CN = 30:1, 1.2 mL) in an NMR tube with addition of 1,3,5-trimethoxybenzene (1.3 mg, 0.0076 mmol) as an internal standard. The mixture was divided into two aliquots. One aliquot was stored at room temperature under an atmosphere of air and irradiated with 24W blue LEDs (positioned approximately 15 cm from the light source). ^1H NMR spectra were recorded at 20 min, 40 min, 60 min, 80 min, 100 min, 120 min, 140 min, 160 min, 180 min to monitor the concentration of *rac*-**CoCat1** at indicated times (**Supplementary Fig. 10**).

Results: As shown in **Supplementary Fig. 10**, ^1H NMR spectroscopy revealed the release of the coordinated CH_3CN ligand upon light irradiation. The resulting coordination-unsaturated complexes appeared to re-coordinate with the excess CD_3CN present in the system. Notably, the ^1H NMR peaks of *rac*-**CoCat1** remained unchanged except for the coordinated CH_3CN signal, indicating that the complex still remained stable after 3 h of irradiation (**Supplementary Fig. 10**, i). To further investigate the ligand dissociation rate under light irradiation, we monitored the concentration of *rac*-**CoCat1** at the specified time intervals. The decay of the concentration of *rac*-**CoCat1** followed first-order kinetics, and the rate constants were determined through regression analysis (**Supplementary Fig. 11**). The ligand dissociation rate constant of *rac*-**CoCat1** under irradiation was calculated to be 3.32×10^{-3} min^{-1} in CD_2Cl_2.

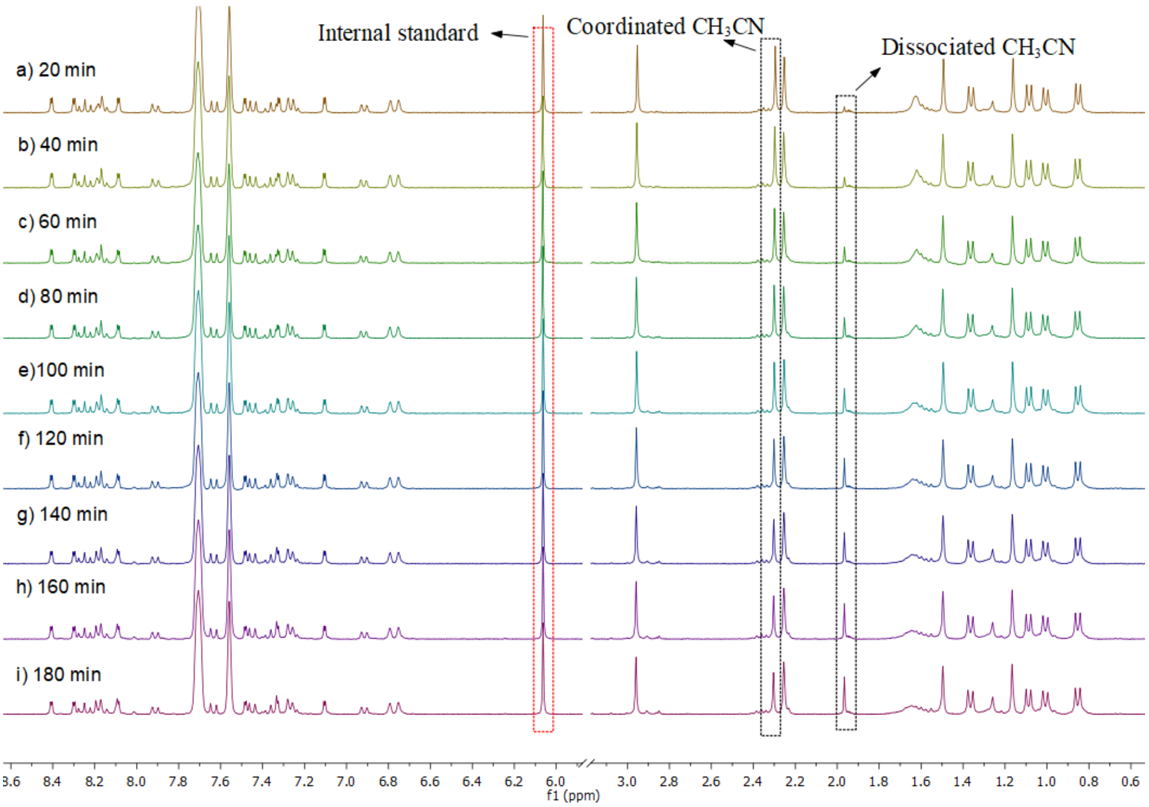

Supplementary Fig. 10. ^{1}H NMR (300 MHz, 298 K) spectra of *rac*-**CoCat1** recorded in CD_2Cl_2/CD_3CN (*v/v* = 30:1) after irradiation at specified time intervals.

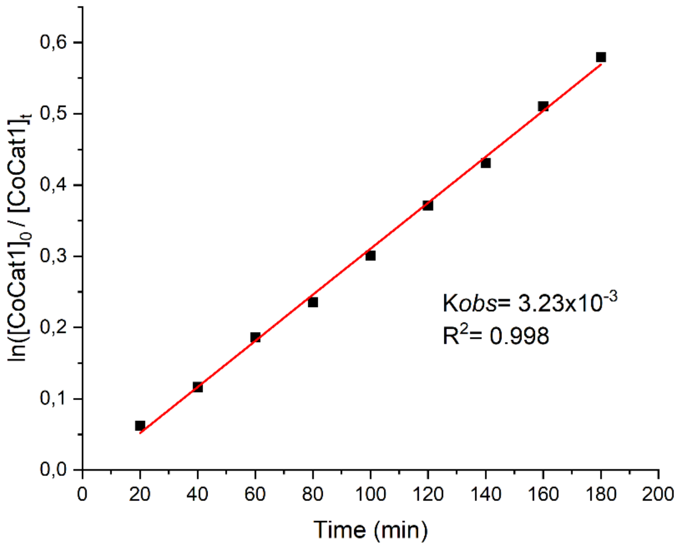

Supplementary Fig. 11. Dissociation rate constant of *rac*-**CoCat1** under blue LEDs irradiation.

6.2 Rates Constant in the Dark

Method: *rac*-**CoCat1** (0.0076 mmol, 20 mg) and CD_3CN (40 µL, 0.76 mmol) was dissolved in CD_2Cl_2 (resulting in CD_2Cl_2/CD_3CN = 30:1, 1.2 mL) in an NMR tube with addition of 1,3,5-trimethoxybenzene (1.3 mg, 0.0076 mmol) as an internal standard. The mixture was divided into two aliquots. The other aliquot was stored at room temperature in the dark under an atmosphere of air. ^1H NMR spectra were recorded at 24 h, 44 h, 68 h, 92 h, 116 h, 144 h, 168 h to monitor the concentration of *rac*-**CoCat1** at indicated times (**Supplementary Fig. 12**).

Results: As shown in **Supplementary Fig. 12**, ^1H NMR spectroscopy revealed a sluggish release of the coordinated CH_3CN ligand under dark conditions. Notably, except for the coordinated CH_3CN signal, the ^1H NMR peaks of *rac*-**CoCat1** remained unchanged after 168 h in the dark (**Supplementary Fig. 12**, g). To further investigate the ligand dissociation rate under dark condition, we monitored the concentration of *rac*-**CoCat1** at the specified time intervals. The decay of the concentration of *rac*-**CoCat1** followed first-order kinetics, and regression analysis (**Supplementary Fig. 13**) yielded a ligand dissociation rate constant of 1.67×10^{-5} min^{-1} in CD_2Cl_2.

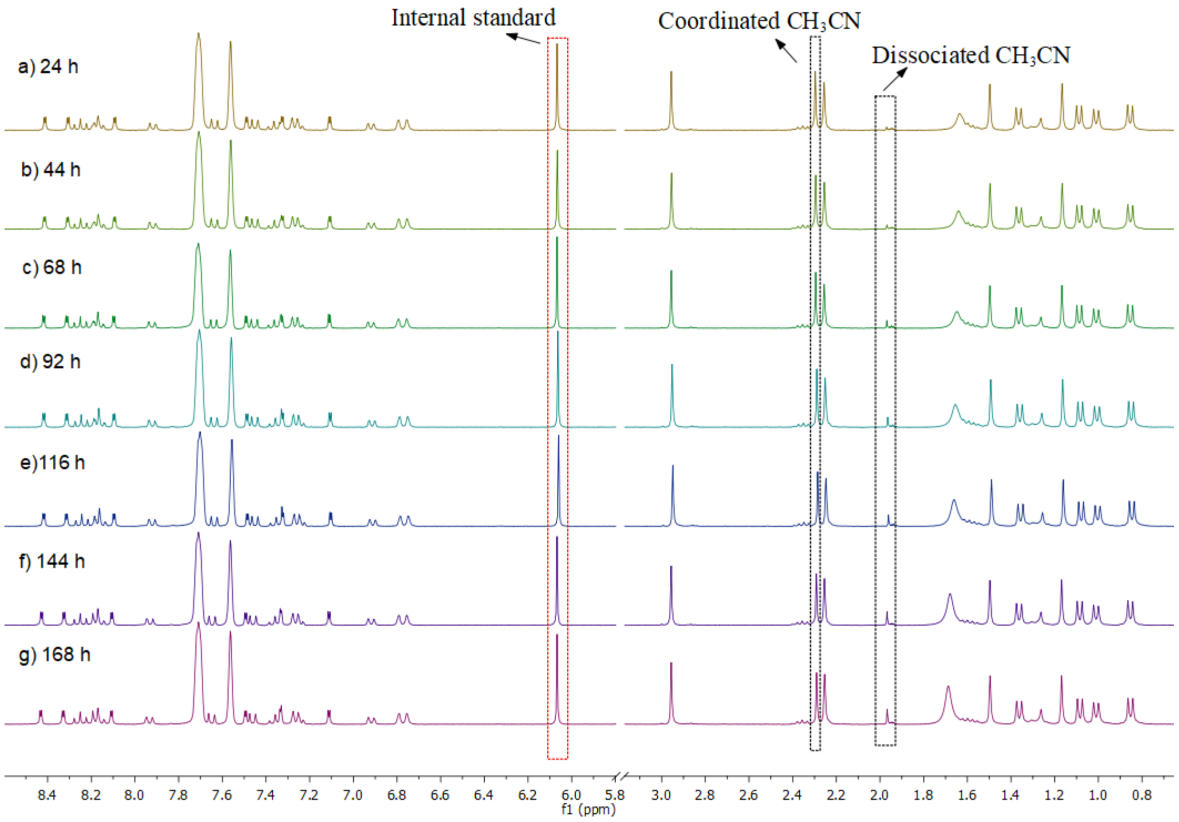

Supplementary Fig. 12. ^{1}H NMR (300 MHz, 298 K) spectra of *rac*-**CoCat1** recorded in CD_2Cl_2 /CD_3CN (*v/v* = 30/1) in dark at specified time intervals.

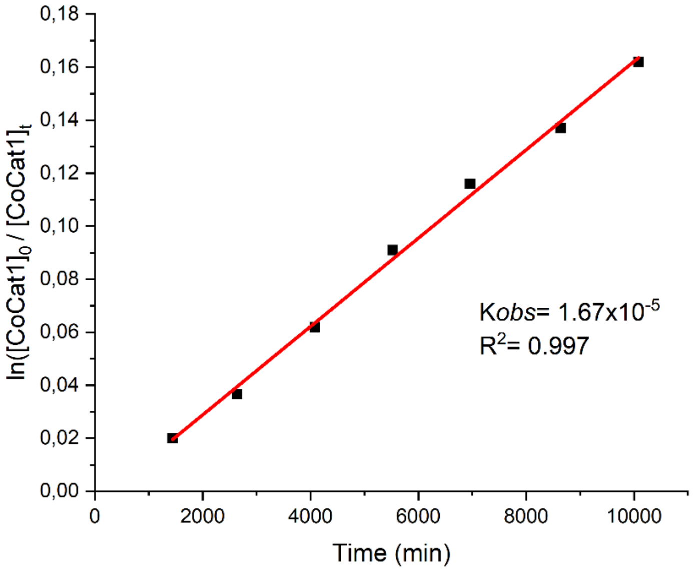

Supplementary Fig. 13. Dissociation rate constant of *rac*-**CoCat1** in dark.

7. Light-Induced Cobalt-Catalyzed Ring Contraction Reaction

7.1 Initial Experiments and Optimization of Reaction Conditions

7.1.1 General Procedure

A dried 5 mL Schlenk tube was charged with the cobalt catalyst (3 − 6 mol %), substrate **1** (0.04 mmol, 1.00 equiv.) and NaBArF (0 − 0.35 equiv.). The tube was purged with nitrogen, and CH_2Cl_2 (0.4 mL, 0.1 M) was added via a syringe. The reaction mixture was degassed using three freeze-pump-thaw cycles. The vial was then positioned approximately 15 cm from a 24 W blue LEDs lamp and stirred under irradiation at the specified temperature for 24 hours. For reactions involving NaBArF, the mixture was stirred at −50 °C for 30 minutes then changed to the specified temperature. Upon completion of the irradiation, the reaction mixture was diluted with cold *n*-hexane, and 1,3,5-trimethoxybenzene was added as an internal standard. The solution was passed through a short silica gel column and eluted with CH_2Cl_2. The combined eluents were concentrated under reduced pressure, and the crude residue was analyzed by ^{1}H NMR spectroscopy to determine the yield. Then, the entire mixture was collected and purified by flash chromatography on silica gel (*n*-hexane/EtOAc=5:1) to afford the product **2**. Racemic samples were prepared using *rac*-**CoCat1**. The enantiomeric excess (ee) of product **2** was determined by HPLC analysis using a chiral stationary phase.

7.1.2 Procedure for Λ-[CoCat3](BArF)$_3$ as Catalyst

A dried 5 mL Schlenk tube was charged with the **Λ-[CoCat3]**(BArF)$_3$ (3 mol %), substrate **1** (0.04 mmol). The tube was purged with nitrogen, and CH_2Cl_2 (0.4 mL, 0.1 M) was added via a syringe. The reaction mixture was degassed using three freeze-pump-thaw cycles. The vial was then positioned approximately 15 cm from a 24 W blue LEDs lamp and stirred under irradiation at −50 °C for 24 hours. Upon completion of the irradiation, the reaction mixture was diluted with cold *n*-hexane, and 1,3,5-trimethoxybenzene was added as an internal standard. The solution was passed through a short silica gel column and eluted with CH_2Cl_2. The combined eluents were concentrated under

reduced pressure, and the crude residue was analyzed by ^{1}H NMR spectroscopy to determine the yield. Then, the entire mixture was collected and purified by flash chromatography on silica gel (*n*-hexane/EtOAc=5:1) to afford the product **2**. The enantiomeric excess (ee) of product **2** was determined by HPLC analysis using a chiral stationary phase.

7.1.3 Additional Optimization and Control experiments

Supplementary Table 1. Control experiments and optimization for cobalt-catalyzed enantioselective ring contraction of isoxazoles under light condition.

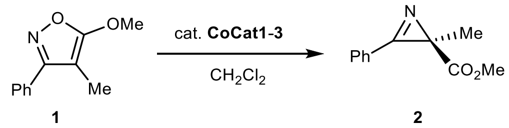

entry	catalyst (mol%)	conditions[a]	T/[°C]	yield[b]	ee(%)[c]
1	Δ-**CoCat1**(3.0)	under air	r.t.	trace	-
2	none	standard	r.t.	0	-
3	Δ-**CoCat3**(3.0)	$CHCl_3$(0.1 M)	-45	trace	-
4	Δ-**CoCat3**(3.0)	MeCN (0.1 M)	-40	0	-
5	Δ-**CoCat3**(3.0)	NaBArF (0.1 equiv.)	-60	57	91
6	Δ-**CoCat3**(4.5)	NaBArF (0.1 equiv.)	-60	66	91
7	Δ-**CoCat3**(4.5)	NaBArF (0.1 equiv.), 32 h	-60	74	88
8	Δ-**CoCat3**(4.5)	NaBArF (0.05 equiv.)	-60	58	90
9	Δ-**CoCat3**(4.5)	NaBArF (0.2 equiv.)	-60	65	90
10	Δ-**CoCat3**(4.5)	NaBArF (0.35 equiv.)	-60	51	91

[a]Standard condition: Substrate **1** (0.04 mmol, 1equiv.) in CH_2Cl_2 (0.1 M) with cobalt catalyst (3 − 4.5 mol%) was stirred at the indicated temperature under nitrogen and irradiation with blue LEDs (24 W) for 24 h. Deviations from these standard conditions are shown. [b]Determined by ^{1}H NMR using 1,3,5-trimethoxybenzene as internal standard. [c]Determined with the purified products by HPLC on chiral stationary phase.

7.1.4 Characterization of Azirine Product 2

Methyl (*R*)-2-methyl-3-phenyl-2*H*-azirine-2-carboxylate (2)

Following the general procedure by using substrate **1** under optimized condition, product (*R*)–**2** was obtained in 78% yield with 91% ee value as a colorless oil. HPLC conditions: Daicel Chiralpak OD-H column, 250 × 4.6 mm, absorbance at 254 nm, mobile phase n-hexane/isopropanol = 98:2, isocratic flow, flow rate 1 mL/min, 25 °C, t_r (major) = 8.7 min, t_r (minor) = 9.7 min. The absolute configuration of (*R*)–**2** was assigned by comparison with published optical rotation and chiral HPLC retention time data. The analytical data match those reported in the literature.[9] **^{1}H NMR** (300 MHz, $CDCl_3$): δ 7.84 (d, *J* = 7.7 Hz, 3H), 7.71 – 7.55 (m, 2H), 3.69 (s, 3H), 1.63 (s, 3H). **HRMS** (ESI, *m/z*): calcd. for $C_{11}H_{11}NO_2H$ $[M+H]^+$: 190.0863, found: 190.0858. **$[\alpha]D^{22}$** = −116.7° (*c* = 1.0, $CHCl_3$).

7.2 Substrate Scope of Ring Contraction to 2*H*-Azirines

7.2.1 Synthesis of Substrates

Chlorinated isoxazole and isoxazole substrates **1b, 1d-1j** were synthesized according to a published procedure. The experimental data are in accordance with the literature.[5,9] Substrates **1c** was synthesized according to a slightly modified published procedure.[5]

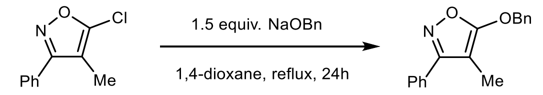

1c

5- Benzyl -4-methyl-3-phenylisoxazole (1c)

To a flame dried Schlenk flask were added chlorinated isoxazole (100 mg, 0.52 mmol), Sodium benzyloxide (101 mg, 0.78 mmol), and 1,4-dioxane (0.5 mL). Subsequently the mixture was refluxed under inert gas atmosphere for 24 h. After cooling to rt, removal of the solvent, the crude product was purified by column chromatography on silica gel (*n*- hexane /EtOAc= 10:1) to afford pure isoxazole

substrates **1c** as colorless oil with 85% yield. **^{1}H NMR** (300 MHz, $CDCl_3$) δ 7.65 (dd, J = 6.5, 3.2 Hz, 2H), 7.52 – 7.33 (m, 8H), 5.40 (s, 2H), 1.94 (s, 3H). **^{13}C NMR** (75 MHz, $CDCl_3$) δ 169.20, 164.72, 135.36, 130.41, 129.58, 128.96, 128.83, 128.79, 128.53, 127.85, 88.06, 73.17, 6.77. **HRMS** (ESI, *m/z*): calcd. for $C_{17}H_{15}NO_2Na$ $[M+Na]^+$: 288.0995, found: 288.0991.

7.2.2 General Procedure for Catalytic Reactions

A dried 5 mL Schlenk tube was charged with the Δ-**CoCat3** (5.1 mg, 6 mol %), substrate (0.03 mmol, 1.00 equiv.) and NaBArF (2.7 mg, 0.003 mmol, 0.1 equiv.). The tube was purged with nitrogen, and CH_2Cl_2 (0.3 mL, 0.1 M) was added via a syringe. The reaction mixture was degassed using three freeze-pump-thaw cycles. The mixture was stirred at −50 °C for 30 minutes. The vial was then positioned approximately 15 cm from a 24 W blue LEDs lamp and stirred under irradiation at -60 °C for 24 hours. Upon completion of the irradiation, the reaction mixture was diluted with cold *n*-hexane, and 1,3,5-trimethoxybenzene was added as an internal standard. The solution was passed through a short silica gel column and eluted with CH_2Cl_2. The combined eluents were concentrated under reduced pressure, and the crude residue was analyzed by ^{1}H NMR spectroscopy to determine the yield. Then, the entire mixture was collected and purified by flash chromatography on silica gel (*n*-hexane/EtOAc=5:1) to afford the product. Racemic samples were prepared using *rac*-**CoCat1**. The enantiomeric excess (ee) of product was determined by HPLC analysis using a chiral stationary phase. The absolute configuration of the products was determined by comparison of the HPLC traces with the literature. [5,9]

Ethyl (*R*)-2-methyl-3-phenyl-2*H*-azirine-2-carboxylate (2b)

Following the general procedure by using **1b** as substrate, product **2b** was obtained in 65% yield with 89% ee value as a colorless oil. HPLC conditions: Daicel Chiralcel OJ-H column, 250 × 4.6 mm, absorbance at 254 nm, *n*hexane/*i*PrOH 80:20, isocratic flow, flow rate 1.0 mL/min, 25 °C, tr (minor) = 6.8 min, tr (major) = 9.7 min. The analytical data are in accordance with the literature. [5, 9]

^{1}**H NMR** (300 MHz, $CDCl_3$): δ 7.82 (d, *J* = 8.2 Hz, 2H), 7.66 – 7.40 (m, 3H), 4.14 (q, *J* = 7.1 Hz, 2H), 1.61 (s, 3H), 1.19 (t, *J* = 7.1 Hz, 3H).

Benzyl (*R*)-2-methyl-3-phenyl-2*H*-azirine-2-carboxylate (2c)

Following the general procedure by using **1c** as substrate, product **2c** was obtained in 62% yield with 93% ee value as a colorless solid. HPLC conditions: Daicel Chiralcel OJ-H column, 250 × 4.6 mm, absorbance at 254 nm, *n*hexane/*i*PrOH 80:20, isocratic flow, flow rate 1.0 mL/min, 25 °C, tr (minor) = 13.3 min, tr (major) = 17.5 min.

^{1}**H NMR** (300 MHz, $CDCl_3$): δ 7.85 (d, *J* = 8.3 Hz, 2H), 7.62 (dq, *J* = 14.3, 7.2 Hz, 3H), 7.46 – 7.14 (m, 5H), 5.29 – 5.00 (m, 2H), 1.67 (s, 3H). ^{13}**C NMR** (75 MHz, $CDCl_3$) δ 173.08, 163.66, 136.04, 133.75, 130.27, 129.46, 128.58, 128.17, 127.84, 122.66, 66.98, 35.75, 17.86. **HRMS** (ESI, *m/z*): calcd. for $C_{17}H_{15}NO_2Na$ $[M+Na]^+$: 288.0995, found: 288.0992. **[α]D^{22}** = −85.6° (*c* = 0.4, $CHCl_3$).

Methyl (*R*)-2-methyl-3-(*p*-tolyl)-2*H*-azirine-2-carboxylate (2d)

Following the general procedure by using **1d** as substrate, product **2d** was obtained in 74% yield with 95% ee value as a colorless solid. HPLC conditions: Daicel Chiralcel OJ-H column, 250 × 4.6 mm, absorbance at 254 nm, *n*hexane/*i*PrOH 80:20, isocratic flow, flow rate 1.0 mL/min, 25 °C, tr (minor) = 8.1 min, tr (major) = 12.4 min. The analytical data are in accordance with the literature. [5, 9]

^{1}**H NMR** (300 MHz, $CDCl_3$): δ 7.73 (d, *J* = 8.1 Hz, 2H), 7.37 (d, *J* = 7.9 Hz, 2H), 3.67 (s, 3H), 2.45 (s, 3H), 1.61 (s, 3H).

Methyl (*R*)- 2-methyl-3-(4-methoxyphenyl)- 2*H*-azirine-2-carboxylate (2e)

Following the general procedure by using **1e** as substrate, product **2e** was obtained in 77% yield with 94% ee value as a colorless solid. HPLC conditions: Daicel Chiralcel OJ-H column, 250 × 4.6 mm,

absorbance at 254 nm, *n*hexane/*i*PrOH 80:20, isocratic flow, flow rate 1.0 mL/min, 25 °C, tr (minor) = 13.8 min, tr (major) = 18.6 min. The analytical data are in accordance with the literature.[5, 9]

^{1}H NMR (300 MHz, $CDCl_3$): δ 7.78 (d, *J* = 8.9 Hz, 2H), 7.06 (d, *J* = 8.8 Hz, 2H), 3.89 (s, 3H), 3.67 (s, 3H), 1.60 (s, 3H).

Methyl (*R*)- 2-methyl-3-(4-chlorophenyl)- 2*H*-azirine-2-carboxylate (2f)

Following the general procedure by using **1f** as substrate, product **2f** was obtained in 62% yield with 90% ee value as a colorless oil. HPLC conditions: Daicel Chiralcel OJ-H column, 250 × 4.6 mm, absorbance at 254 nm, *n*hexane/*i*PrOH 80:20, isocratic flow, flow rate 1.0 mL/min, 25 °C, tr (minor) = 7.6 min, tr (major) = 10.5 min. The analytical data are in accordance with the literature.[5, 9]

^{1}H NMR (300 MHz, $CDCl_3$): δ 7.78 (d, *J* = 8.6 Hz, 2H), 7.56 (d, *J* = 8.6 Hz, 2H), 3.69 (s, 3H), 1.62 (s, 3H).

Methyl (*R*)-2-methyl-3-(4-(trifluoromethyl)phenyl)-2*H*-azirine-2-carboxylate (2g)

Following the general procedure by using **1g** as substrate, product **2g** was obtained in 48% yield with 90% ee value as a colorless solid. HPLC conditions: Daicel Chiralcel OJ-H column, 250 × 4.6 mm, absorbance at 254 nm, *n*hexane/*i*PrOH 97:3, isocratic flow, flow rate 1.0 mL/min, 25 °C, tr (minor) = 7.6 min, tr (major) = 9.5 min. The analytical data are in accordance with the literature.[5,9]

^{1}H NMR (300 MHz, $CDCl_3$): δ 7.98 (d, *J* = 7.9 Hz, 2H), 7.84 (d, *J* = 8.1 Hz, 2H), 3.70 (s, 3H), 1.65 (s, 3H).

Methyl (*R*)-2-methyl-3-(naphthalen-2-yl)-2*H*-azirine-2-carboxylate (2h)

Following the general procedure by using **1h** as substrate, product **2h** was obtained in 81% yield with 79% ee value as a colorless solid. HPLC conditions: Daicel Chiralcel OD-H column, 250 × 4.6 mm,

absorbance at 254 nm, *n*hexane/*i*PrOH 97:3, isocratic flow, flow rate 1.0 mL/min, 25 °C, tr (minor) = 10.1 min, tr (major) = 11.6 min. The analytical data are in accordance with the literature. [5,9]

^{1}H NMR (300 MHz, $CDCl_3$): δ 8.25 (d, *J* = 1.4 Hz, 1H), 8.04 – 7.84 (m, 4H), 7.74 – 7.53 (m, 2H), 3.70 (s, 3H), 1.70 (s, 3H).

Methyl (*R*)- 2-propyl-3-phenyl-2*H*-azirine-2-carboxylate (2i)

Following the general procedure by using **1i** as substrate, product **2i** was obtained in 68% yield with 95% ee value as a colorless solid. HPLC conditions: Daicel Chiralcel OJ-H column, 250 × 4.6 mm, absorbance at 254 nm, *n*hexane/*i*PrOH 90:10, isocratic flow, flow rate 1.0 mL/min, 25 °C, tr (minor) = 7.3 min, tr (major) = 9.3 min. The analytical data are in accordance with the literature. [5,9]

^{1}H NMR (300 MHz, $CDCl_3$): δ 7.91 – 7.78 (m, 2H), 7.69 – 7.48 (m, 3H), 3.67 (s, 3H), 2.05 (dt, *J* = 8.5, 7.4 Hz, 2H), 1.35 – 1.17 (m, 2H), 0.90 (t, *J* = 7.3 Hz, 3H).

Methyl (*R*)-2-benzyl-3-phenyl-2*H*-azirine-2-carboxylate (2j)

Following the general procedure by using **1j** as substrate, product **2j** was obtained in 76% yield with 97% ee value as a colorless solid. HPLC conditions: Daicel Chiralcel OJ-H column, 250 × 4.6 mm, absorbance at 254 nm, *n*hexane/*i*PrOH 80:20, isocratic flow, flow rate 1.0 mL/min, 25 °C, tr (minor) = 16.2 min, tr (major) = 24.8 min. The analytical data are in accordance with the literature. [5,9]

^{1}H NMR (300 MHz, $CDCl_3$): δ 7.64 – 7.51 (m, 3H), 7.45 (t, *J* = 7.4 Hz, 2H), δ 7.23 – 7.10 (m, 2H), δ 3.74 – 3.58 (m, 4H), 3.13 (d, *J* = 14.7 Hz, 1H).

8. Mechanistic Experiments

8.1 Procedure Using Zinc as Reducing Agent

A dried 5 mL Schlenk tube was charged with the Δ-**CoCat3** (4.5 mol %), substrate **1** (0.04 mmol, 1.00 equiv.) and zinc (0.6 equiv.). The tube was purged with nitrogen, and CH_2Cl_2 (0.4 mL, 0.1 M) was added via syringe. (Note: the reaction mixture was immediately frozen by immersion in liquid nitrogen after addition of solvent). The reaction mixture was degassed using three freeze-pump-thaw cycles and stirred in the dark at −50 °C for 24 hours. After that, the reaction mixture was diluted with cold *n*-hexane, and 1,3,5-trimethoxybenzene was added as an internal standard. The solution was passed through a short silica gel column and eluted with CH_2Cl_2. The combined eluents were concentrated under reduced pressure, and the crude residue was analyzed by ^{1}H NMR spectroscopy to determine the yield. Then, the entire mixture was collected and purified by flash chromatography on silica gel (*n*-hexane/EtOAc=5:1) to afford the product **2**. The enantiomeric excess (ee) of product **2** was determined by HPLC analysis using a chiral stationary phase.

8.2 Reaction in the Dark Using Pre-Irradiated Δ-CoCat3

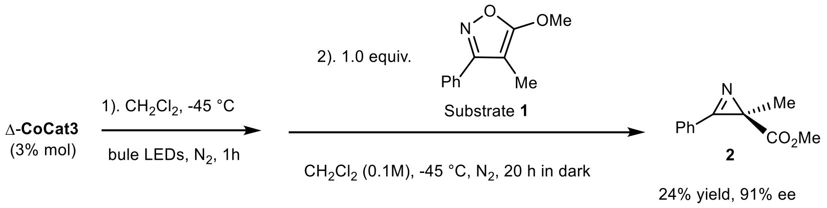

A dried 5 mL Schlenk tube was charged with Δ-**CoCat3** (3 mol%) and CH_2Cl_2 (0.4 mL). The reaction mixture was degassed under nitrogen through three freeze-pump-thaw cycles. The vial was then placed approximately 15 cm from a 24 W blue LEDs lamp and stirred under irradiation at –45 °C for 1 h. Substrate **1** (0.04 mmol, 1.00 equiv.) was subsequently added under a nitrogen atmosphere via Schlenk line at –45 °C, and the mixture was degassed again using three freeze-pump-thaw cycles. The reaction was conducted in the dark at –45 °C for 20 h. Afterwards, the mixture was diluted with

cold n-hexane and 1,3,5-trimethoxybenzene was added as internal standard. The solution was passed through a short silica gel column and eluted with CH_2Cl_2. The combined eluents were concentrated under reduced pressure, and the crude residue was analyzed by ^1H NMR spectroscopy to determine the yield. Then, the entire mixture was collected and purified by flash chromatography on silica gel (*n*-hexane/EtOAc=5:1) to afford the product **2**. The enantiomeric excess (ee) of product **2** was determined by HPLC analysis using a chiral stationary phase.

8.3 Identification of Biphenyl Byproduct after Irradiation of Cobalt Catalyst

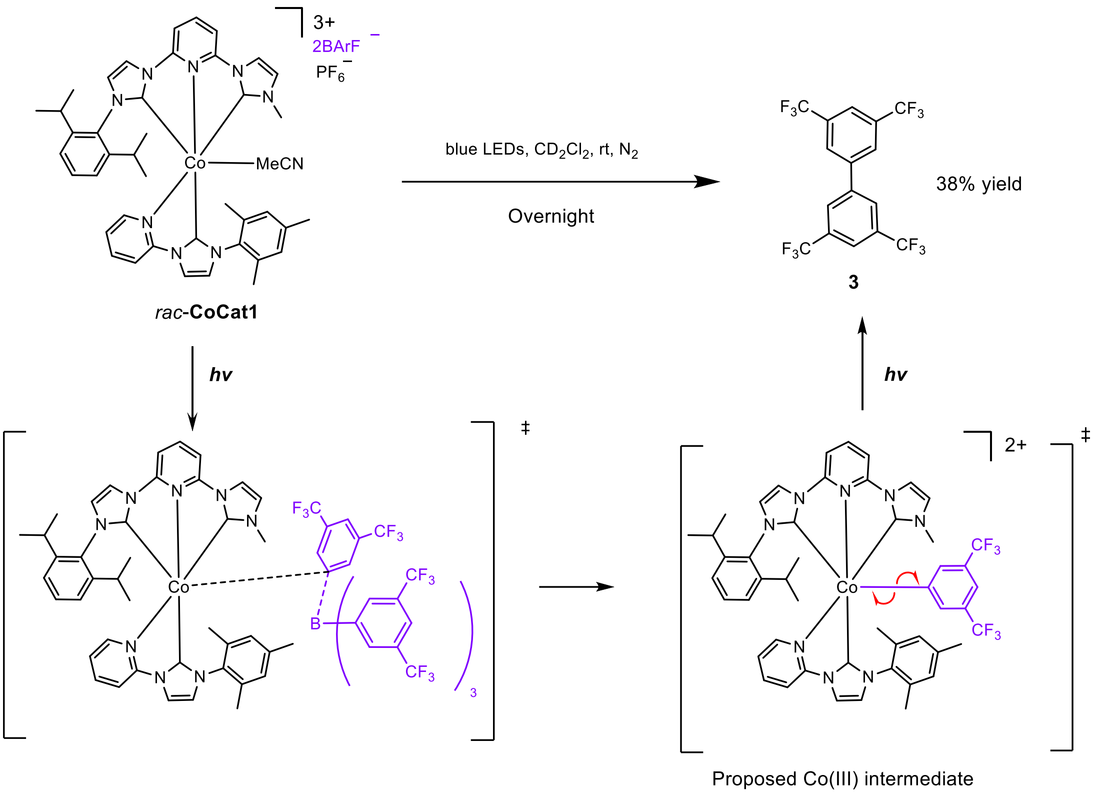

Supplementary Fig. 14. Proposed mechanism for the formation of product **3**.

8.3.1 Detection and Identification of the Biphenyl Byproduct 3

General Procedure: *rac*-**CoCat1**(0.0038 mmol, 10 mg) and CD_2Cl_2 (0.5 mL) was added to an NMR tube and the solution was stirred at room temperature under irradiation with a 24W blue LEDs for 4 hours. After that, ^{19}F NMR, gas chromatography-mass spectrometry and TLC of the solutions were recorded.

Results: The ^{19}F NMR spectra of irradiated *rac*-**CoCat1** revealed the appearance of additional fluorine-containing species. Since the cobalt cation in *rac*-**CoCat1** does not contain fluorinated groups, we initially speculated that these species originate from the BArF anions. To further investigate, the irradiated *rac*-**CoCat1** sample was analyzed using thin-layer chromatography (TLC) with hexane as the eluent. A highly non-polar spot was observed, indicating the formation of a new organic compound (**Supplementary Fig. 15**). Subsequently, gas chromatography-mass spectrometry (GC-MS) analysis (**Supplementary Fig. 16**) further suggested the unknown fluorine-containing should be 3,3',5,5'-tetrakis(trifluoromethyl)biphenyl through its molecular mass.

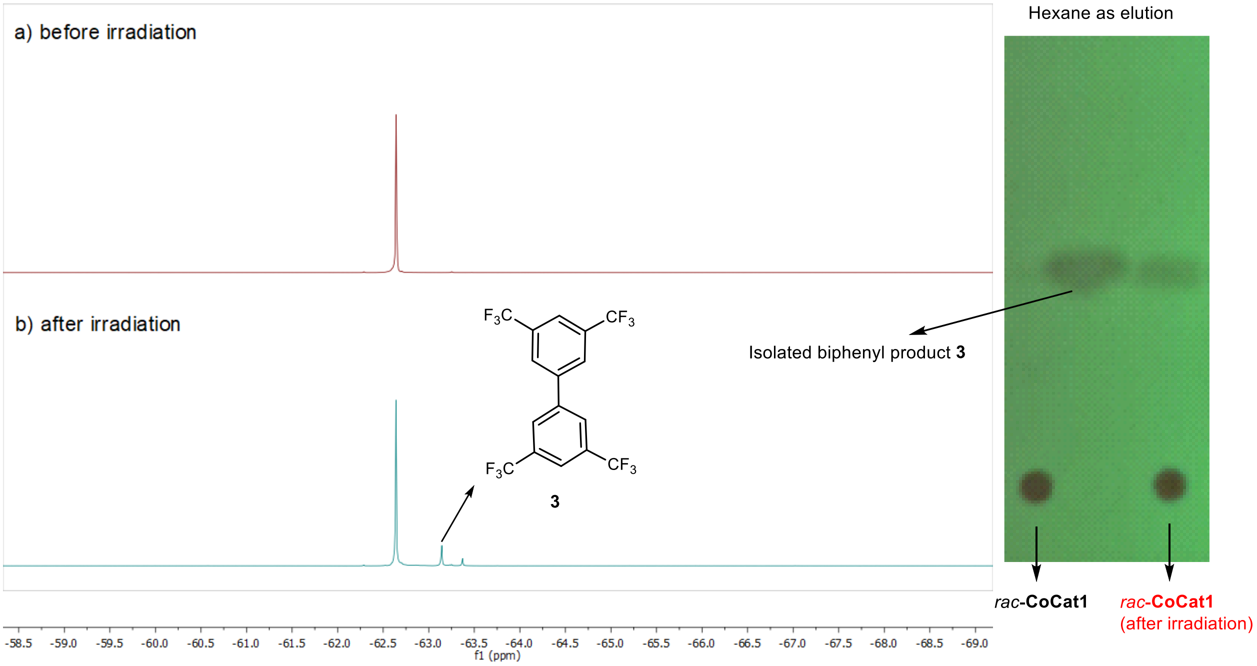

Supplementary Fig. 15. ^{19}F NMR spectra (282 MHz, 298 K, CD_2Cl_2) of *rac*-**CoCat1**(left). a) Before irradiation. b) After irradiation. TLC monitoring of *rac*-**CoCat1** after irradiation(right).

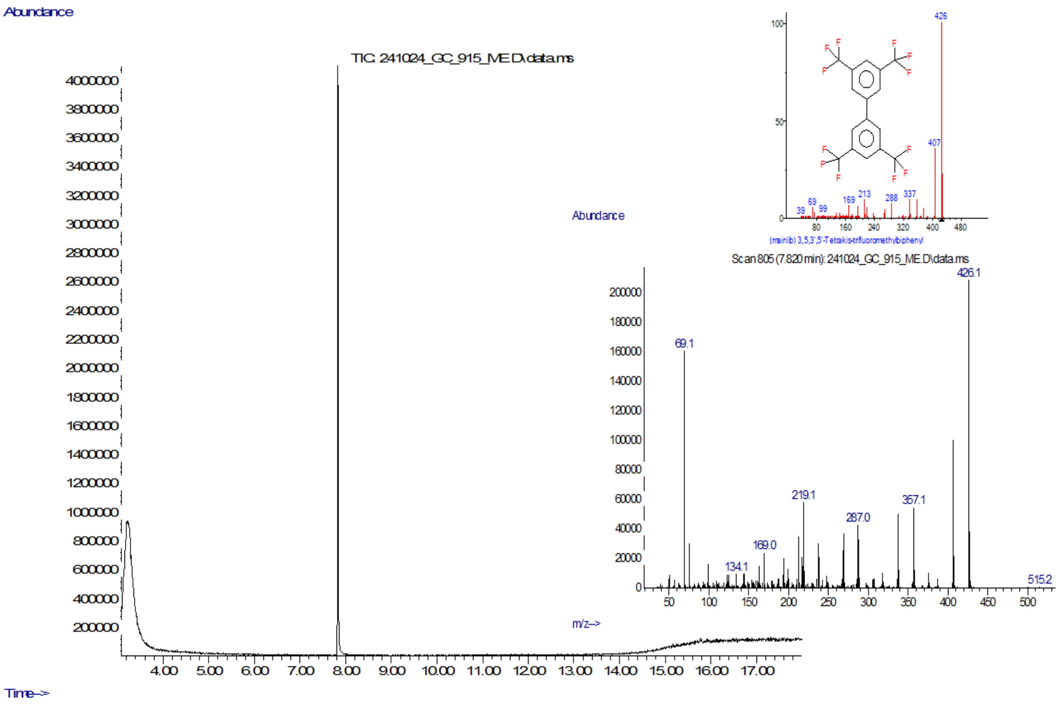

Supplementary Fig. 16. GC-MS of *rac*-**CoCat1** after irradiation.

The pure product **3** was isolated by flash chromatography on silica gel using *n*-hexane as the eluent and spectroscopic data of the isolated product further corroborated its identity as 3,3',5,5'-tetrakis(trifluoromethyl)biphenyl, matching previously reported literature values.[10]

^{1}H NMR (300 MHz, $CDCl_3$): δ 8.07 (s, 4H), 8.03 (s, 2H). **^{13}C NMR** (75 MHz, $CDCl_3$): δ 140.61, 133.11 (q, J = 33.7 Hz), 127.67 (q, J = 3.8 Hz), 123.10 (q, J = 272.9 Hz), 123.10 – 122.51 (m). **^{19}F NMR** (282 MHz, $CDCl_3$): δ -68.28. **HRMS (APCI)**: calcd for $C_{16}H_6F_{12}$ $[M]^-$: 426.0272, found: 426.0281. **GC-MS**: [t = 7.820 min], *m/z*: 426 $[M]^+$.

8.3.2 Determination of the Yield of Biphenyl Byproduct 3

A dried 5 mL Schlenk tube was charged with *rac*-**CoCat1**(0.0019 mmol, 5.0 mg) was purged with nitrogen, and CD_2Cl_2 (0.5 mL) was added via syringe. The reaction mixture was degassed under nitrogen using three freeze-pump-thaw cycles. The vial was then placed approximately 15 cm from a 24 W blue LEDs lamp and stirred under irradiation at room temperature 20 h. Afterwards, 2,6-

bis(trifluoromethyl)bromobenzene (0.0076 mmol, 2.2 mg) was added as an internal standard, and the crude residue was analyzed by ^{19}F NMR spectroscopy to determine the yield.

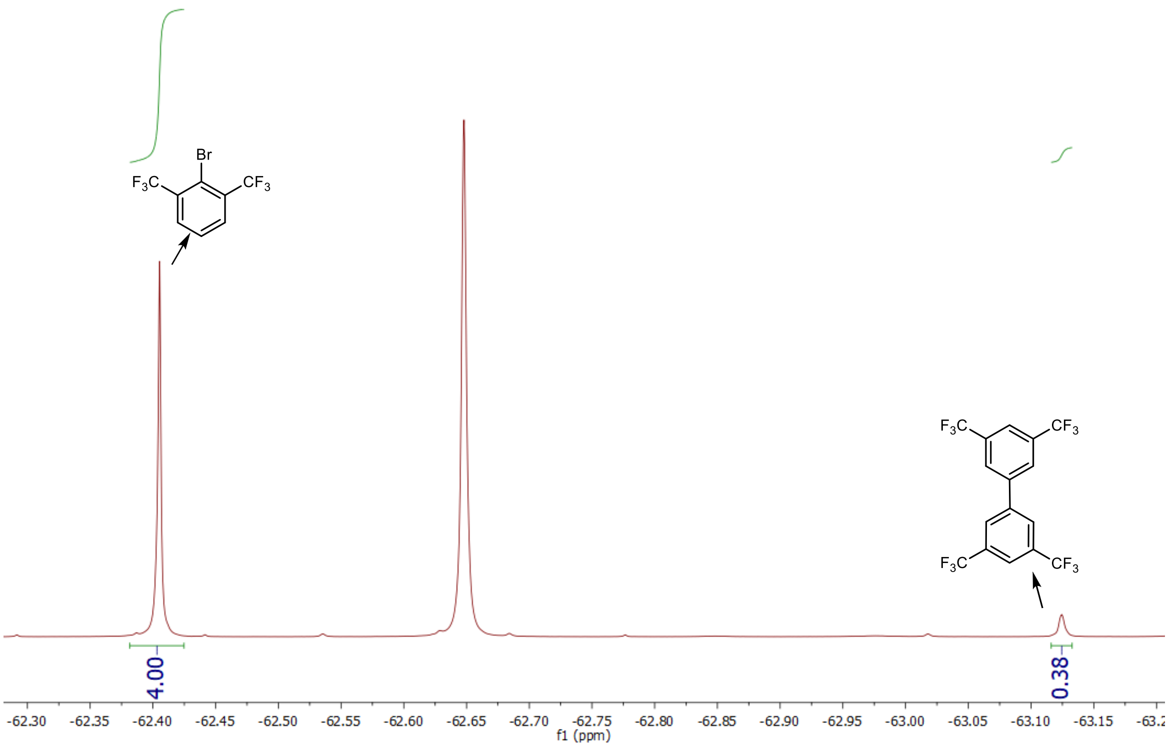

Supplementary Fig. 17. ^{19}F NMR (565 MHz, 298 K, CD_2Cl_2) spectrum of a reaction of *rac*-**CoCat1** exposure to a 24 W blue LEDs overnight.

8.4 Detection and Identification of Boron Fragments

To investigated the remaining borane species, we conducted the following control experiments.

Method: A dried 5 mL Schlenk tube was charged with *rac*-**CoCat1** (13 mg, 0.005 mmol) and NaBArF (8.9 mg, 0.01 mmol) and CH_2Cl_2 (1 mL). The reaction mixture was degassed via three freeze-pump-thaw cycles under nitrogen. The vial was placed approximately 15 cm from a 24 W blue LED lamp and stirred under irradiation at room temperature for 24 h. The crude residue was concentrated to dryness and analyzed by ^{11}B- and ^{19}F-NMR spectroscopy in CD_2Cl_2.

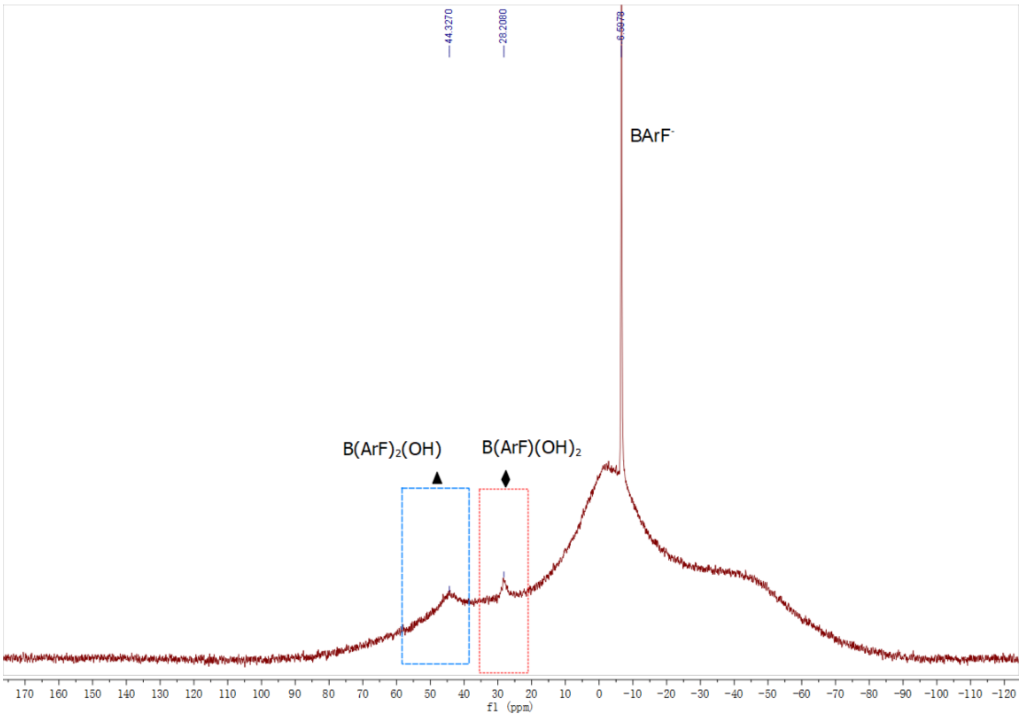

Supplementary Fig. 18. ^{11}B-NMR (96 MHz, 298 K, CD_2Cl_2) spectrum of the crude product after irradiation. Triangle: $B(ArF)_2(OH)$, Diamond: $B(ArF)(OH)_2$.

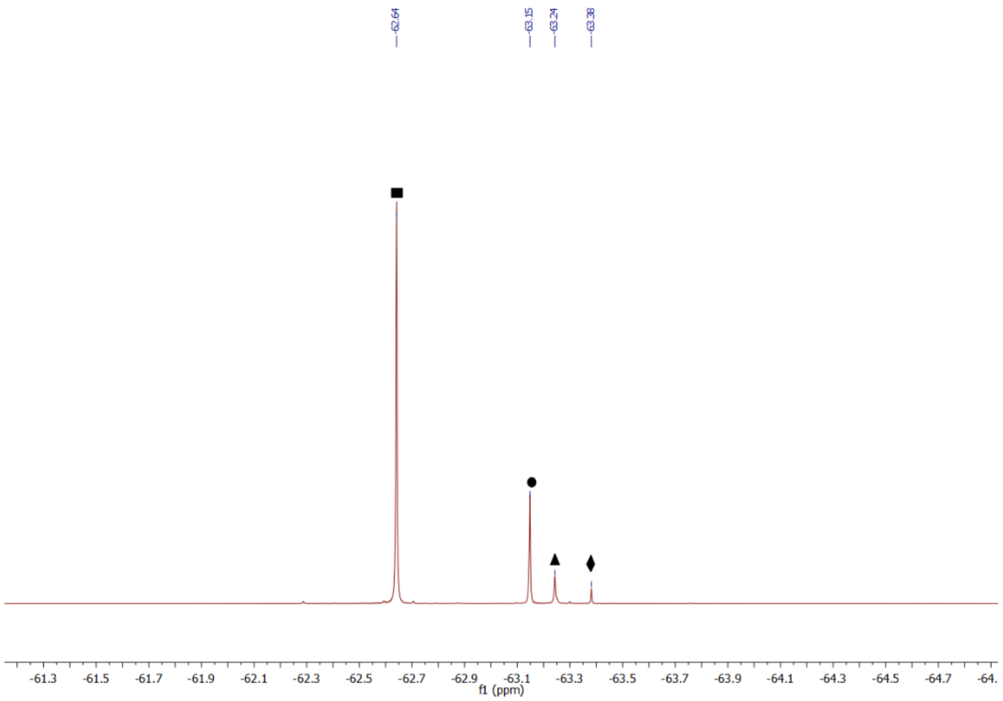

Supplementary Fig. 19. ^{19}F-NMR (282 MHz, 298 K, CD_2Cl_2) spectrum of the crude product after irradiation. Square: $Na[BArF_4]$, circle: 3,3',5,5'-tetrakis(trifluoromethyl)biphenyl, triangle: $B(ArF)_2(OH)$, and Diamond: $B(ArF)(OH)_2$.

Results: **Supplementary Fig. 18** displays the ^{11}B NMR spectra of *rac*-**CoCat1** after irradiation under nitrogen for 24 h in CH_2Cl_2. Instead of the signal corresponding to $B(ArF)_3$, two borane signals at 44.3 ppm and 28.2 ppm were detected. These signals can be assigned to $B(ArF)_2(OH)$ and $B(ArF)(OH)_2$, respectively, based on literature precedents.[11,12] This is supposed to be caused by C-B bond cleavage events occurred in the presence of adventitious water. Related reaction of $B(ArF)_3$ was also observed in previous published work.[10-13] Complementary ^{19}F NMR analysis of the crude product **(Supplementary Fig. 19**) reveals two dominant signals at -63.24 and -63.38 ppm alongside the peaks of BArF (-62.64 ppm) and 3,3',5,5'-tetrakis(trifluoromethyl)biphenyl (-63.15 ppm). These are tentatively assigned to $B(ArF)_2(OH)$, and $B(ArF)(OH)_2$, respectively, further supporting the proposed hydrolytic degradation pathway.

8.5 Catalysis Reaction with [Co1a](NTf_2)$_3$ as Catalyst

A dried 5 mL Schlenk tube was charged with **[Co1a](NTf_2)$_3$** (3 mol %), substrate **1** (0.04 mmol, 1.00 equiv.) and NaBArF (0 or 0.1 equiv.). The tube was purged with nitrogen, and CH_2Cl_2 (0.4 mL, 0.1 M) was added via a syringe. The reaction mixture was degassed using three freeze-pump-thaw cycles. The vial was then positioned approximately 15 cm from a 24 W blue LEDs lamp and stirred under irradiation at room temperature for 24 hours. Upon completion of the irradiation, the reaction mixture was diluted with cold *n*-hexane, and 1,3,5-trimethoxybenzene was added as an internal standard. The solution was passed through a short silica gel column and eluted with CH_2Cl_2. The combined eluents were concentrated under reduced pressure, and the crude residue was analyzed by ^1H NMR spectroscopy to determine the yield.

9. Photophysical Experiments

9.1 Photophysical Measurements

Optically diluted solutions with concentrations in the order of 10^{-3} or 10^{-6} M were prepared in spectroscopic or HPLC grade solvents for steady-state and time-resolved absorption analysis. Absorption spectra were recorded at room temperature on a Varian Cary 300 spectrophotometer with 1 cm or 0.2 cm quartz cuvettes. Degassed solutions were prepared via 4 consecutive freeze-pump-thaw cycles and spectra were taken using home-made Schlenk quartz cuvette; alternatively, N_2-saturated solutions were prepared in a glove box using the same glassware. The estimated experimental errors are 2 nm on the band maximum and 5% on the molar absorption coefficient.

9.2 Irradiation Experiment

Irradiation of samples in CH_3CN or CH_2Cl_2 were performed at room temperature on thoroughly stirred N_2-saturated solutions by using a Kessil lamp at 467 nm (40 W) or blue LED (LED Engin LuxiGenTM LZ1-10DB00, $\lambda = 450 - 480$ nm) operating at 10 V and 500 mA. The sample was placed at 15 cm from the source.

9.3 Transient Absorption Spectroscopy

Pump-probe transient absorption measurements were performed with an Ultrafast Systems HELIOS (HE-VIS-NIR) femtosecond transient absorption spectrometer by using, as excitation source, a Newport Spectra Physics Solstice-F-1K-230 V laser system, combined with a TOPAS Prime (TPR-TOPAS-F) optical parametric amplifier (pulse width: 100 fs, 1 kHz repetition rate) tuned at 350 nm. The pump energy on the sample was 8 μJ/pulse. The probe continuum generation was in the visible range (450-800 nm). The overall time resolution of the system is 300 fs. Air-equilibrated solutions of the sample in 0.2 cm optical path cells were analyzed under continuous stirring. Surface Xplorer software from Ultrafast Systems was used for data acquisition and analysis. The raw 3D surfaces

were corrected for the chirp of the probe pulse prior to analysis. The error on the lifetime derives from the fitting procedure.

9.4 Absorption Spectra of Cobalt Complexes after Irradiation

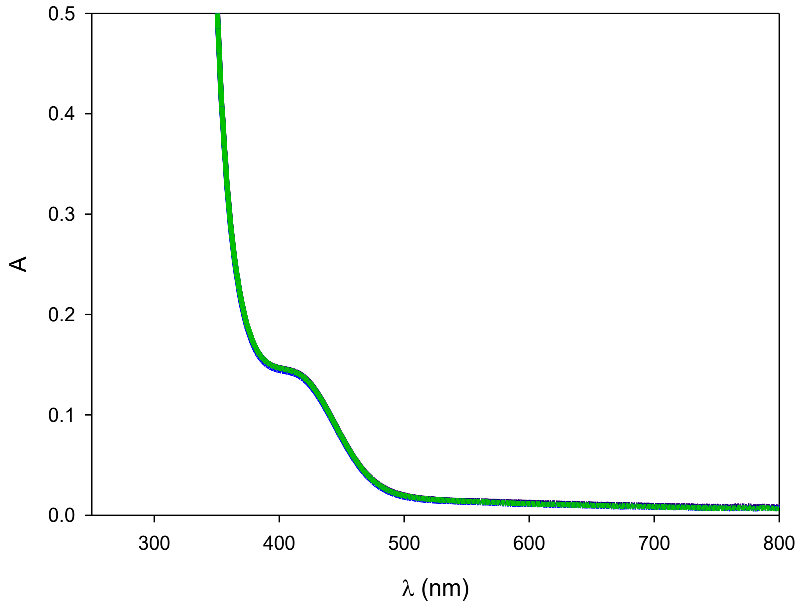

Supplementary Fig. 20. Absorption spectra of a 0.3 mM solution of *rac*-**CoCat3** (black line) in CH_3CN under inert atmosphere and evolution upon 30 min irradiation at 467 nm.

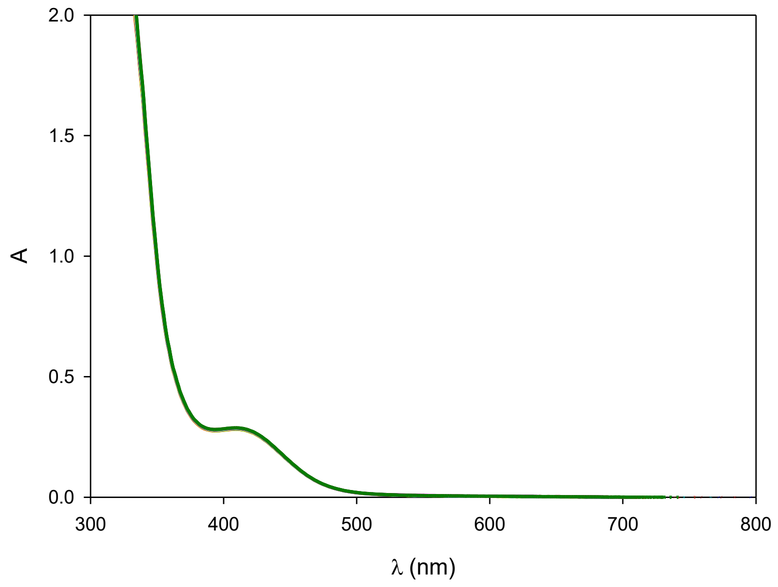

Supplementary Fig. 21. Absorption spectra of a 0.7 mM solution of *rac*-**CoCat1(NTf$_2$)$_3$** (black line) in CH_2Cl_2 under inert atmosphere and evolution upon 30 min irradiation at 467 nm.

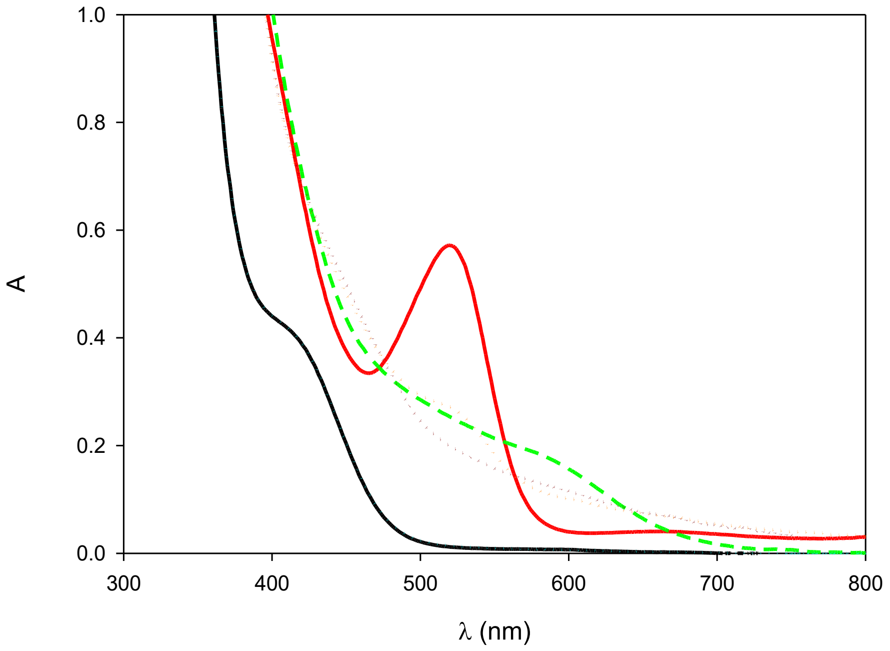

Supplementary Fig. 22. Absorption spectra of a 0.8 mM *rac*-**CoCat3** in dichloromethane (black line) and in presence of substrate **1** (0.1 M) (dotted cyan line) under inert atmosphere. After irradiation with 467 nm light for 30 min in presence (dotted orange line) and without substrate **1** (red line). Absorption spectra recorded immediately after air equilibration in presence (dotted brown line) and without substrate **1** (dash green line)

10. Single Crystal X-Ray Diffraction

10.1 Crystal Structure of Complex Δ-CoCat4

Attempts to crystallize Δ- or Λ-**CoCat1-3** with BArF or PF_6 counterions were not successful in our hands. As an alternative, Δ-**CoCat4**, which is an analogue of Δ-**CoCat1-3** containing three PF_6 anions, was synthesized and employed to obtain a crystal structure. It is interesting to note that Δ-**CoCat4** (with and without BArF counterions) is catalytically not active (only trace product formation for conversion **1** → **2** using 3 mol% of Δ-**CoCat4** at −30 °C). Single crystals of Δ-**CoCat4** suitable for X-ray diffraction were obtained by slow evaporation of a CH_3OH solution of Δ-**CoCat4** at room temperature.

A suitable crystal of $C_{61}H_{59}CoN_9(PF_6)_3 \cdot C_2H_3N$ was selected under inert oil and mounted using a MiTeGen loop. Intensity data of the crystal were recorded with a D8 Quest diffractometer (Bruker AXS). The instrument was operated with Mo-Kα radiation (0.71073 Å, microfocus source) and equipped with a PHOTON III C14 detector. Evaluation, integration and reduction of the diffraction data was carried out using the Bruker APEX 3 software suite.[14] Multi-scan and numerical absorption corrections were applied using the SADABS program.[15,16] The structure was solved using dual-space methods (SHELXT-2018/2) and refined against F^2 (SHELXL-2019/1 using ShelXle interface).[17-19] All non-hydrogen atoms were refined with anisotropic displacement parameters. The hydrogen atoms were refined using the "riding model" approach with isotropic displacement parameters 1.2 times (1.5 times for terminal methyl groups) of that of the preceding carbon atom. Four out of six $[PF_6]^-$ anions were found disordered and were refined accordingly using the DSR plugin[20] implemented in ShelXle. The diffuse residual electron density in the solvent accessible voids was eliminated using the SQUEEZE algorithm implemented in the PLATON software.[21,22] CCDC 2425622 contains the supplementary crystallographic data for this paper. These data can be obtained free of charge from The Cambridge Crystallographic Data Centre via www.ccdc.cam.ac.uk/structures. The absolute configuration of complex was assigned to Δ-configuration according to the crystal structure.

Supplementary Table 2. Selected crystallographic data and details of the structure determination for $C_{61}H_{59}CoN_9(PF_6)_3 \cdot C_2H_3N$.

Identification code	**Δ-CoCat4**
Empirical formula	$C_{63}H_{62}CoF_{18}N_{10}P_3$
Molar mass / g·mol^{-1}	1453.06
Space group (No.)	*I*2 (5)
a / Å	31.4015(13)
b / Å	12.6775(6)
c / Å	33.416(3)
β / °	97.2590(10)
V / Å^3	13195.9(13)
Z	8
$\rho_{calc.}$ / g·cm^{-3}	1.463
μ / mm^{-1}	0.432
Color	yellow
Crystal habitus	plate
Crystal size / mm^3	0.204 x 0.196 x 0.066
T / K	100
λ / Å	0.71073 (Mo-K$_{\alpha}$)
θ range / °	2.077 to 25.727
Range of Miller indices	$-38 \le h \le 38$
	$-15 \le k \le 15$
	$-40 \le l \le 40$
Absorption correction	multi-scan and numerical
$T_{\min}$, $T_{\max}$	0.9064, 1.0000
R_{int}, R_{σ}	0.0584, 0.0465
Completeness of the data set	0.999
No. of measured reflections	117561
No. of independent reflections	25105
No. of parameters	1994
No. of restraints	4435
S (all data)	1.024
$R(F)$ ($I \ge 2\sigma(I)$, all data)	0.0478, 0.0548
$wR(F^2)$ ($I \ge 2\sigma(I)$, all data)	0.1079, 0.1111
Extinction coefficient	0.00016(4)
Flack parameter *x*	0.012(4)
$\Delta\rho_{\max}$, $\Delta\rho_{\min}$ / e·Å^{-3}	0.827, –0.666

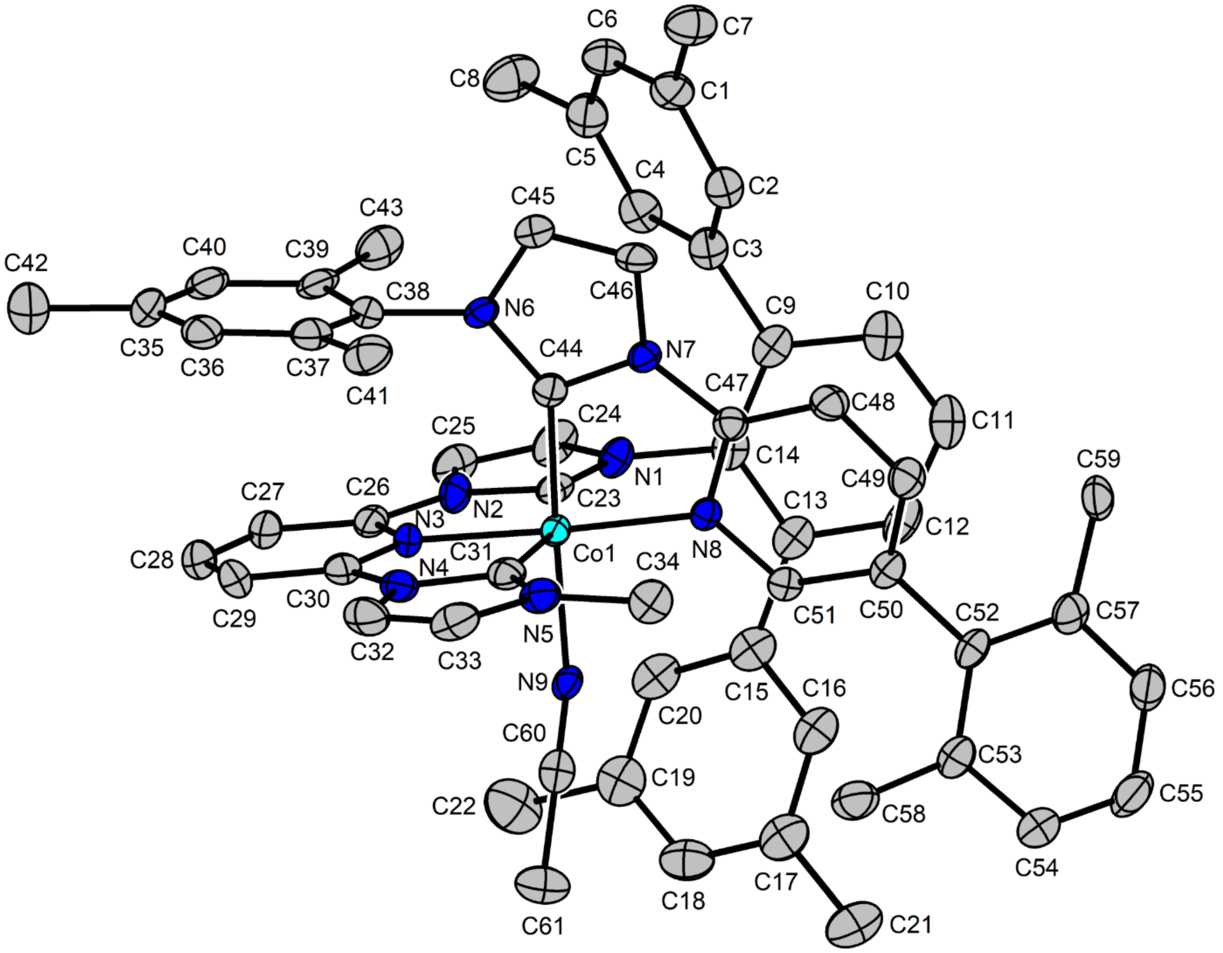

Supplementary Fig. 23. Crystal structure of Δ-**CoCat4**. One out of the two symmetry independent molecules is shown. Three $[PF_6]^-$ anions, the acetonitrile solvent molecules and the hydrogen atoms are not shown. Displacement ellipsoids are shown at 50 % probability level at 100 K.

Alerts of CheckCif for Δ-CoCat4:

Alert level B: PLAT910_ALERT_3_B Missing # of FCF Reflection(s) Below Theta (Min). 11 Note

Author Response: A few reflections could not be collected using the selected measurement strategy and the instrument.

10.2 Crystal Structure of Complex Co-bpq

Attempts to obtain a pure Cobalt complex with **TL1** as tridentate ligand and 2,2'-biquinoline as bidentate ligand (**Co-bpq**) were not successful. The crude **Co-bpq** product was synthesized by reacting **CoL1** with an equimolar amount of 2,2'-biquinoline in 1,2-dichloroethane at 50 °C under aerobic conditions for 24 h. Crystal of this complex could be obtained by slow evaporation of a CH_2Cl_2 solution of **Co-bpq** crude sample at room temperature.

A suitable crystal of $C_{39}H_{33}CoN_7(PF_6)_2 \cdot 2CH_2Cl_2$ was selected under inert oil and mounted using a MiTeGen loop. Intensity data of the crystal were recorded with a STADIVARI diffractometer (Stoe & Cie). The diffractometer was operated with Cu-Kα radiation (1.54186 Å, microfocus source) and equipped with a Dectris PILATUS 300K detector. Evaluation, integration and reduction of the diffraction data was carried out using the X-Area software suite.[23] Multi-scan and numerical absorption corrections were applied with the LANA and X-RED32 modules of the X-Area software suite.[24, 25] The structure was solved using dual-space methods (SHELXT-2018/2) and refined against F^2 (SHELXL-2019/1 using ShelXle interface).[17-19] All non-hydrogen atoms were refined with anisotropic displacement parameters. The hydrogen atoms were refined using the "riding model" approach with isotropic displacement parameters 1.2 times (1.5 times for the methyl groups) of that of the preceding carbon atom. CCDC 2452612 contains the supplementary crystallographic data for this paper. These data can be obtained free of charge from The Cambridge Crystallographic Data Centre via www.ccdc.cam.ac.uk/structures.

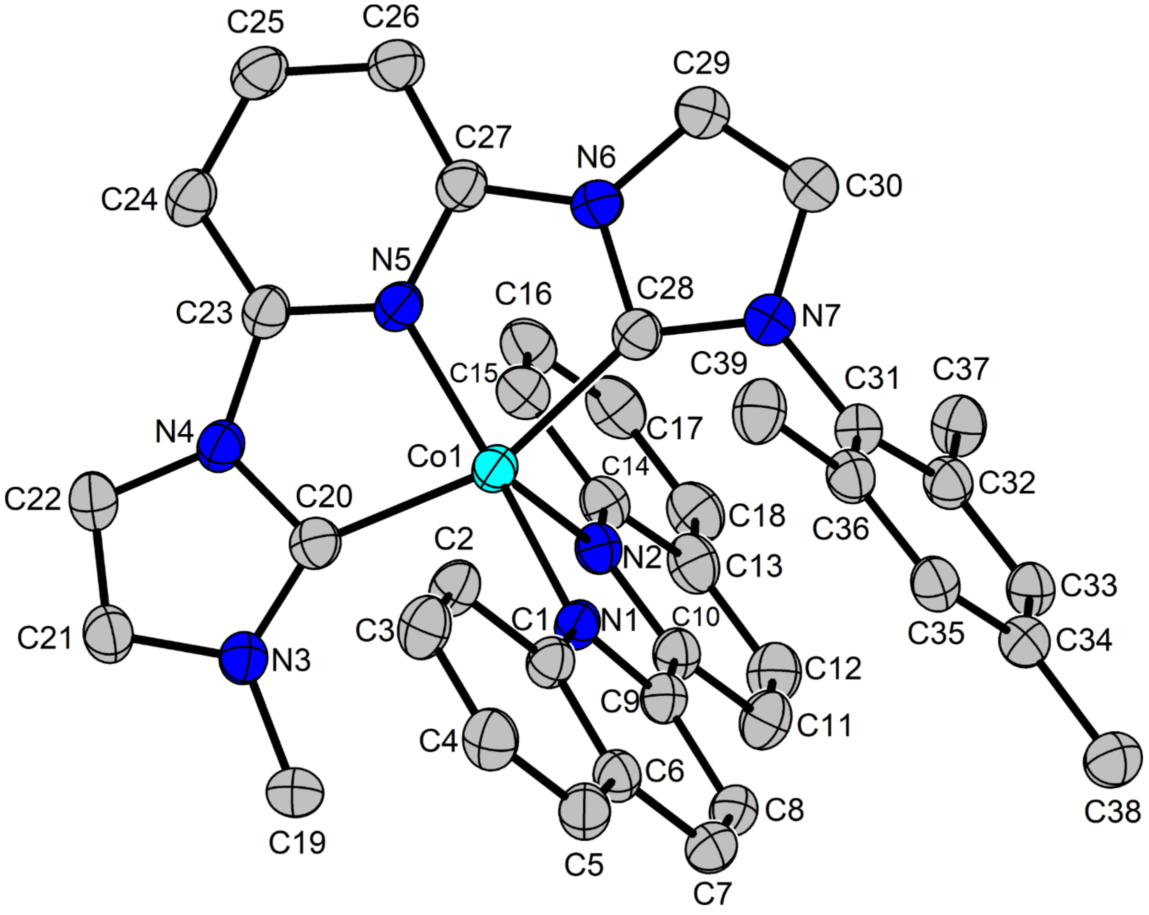

Supplementary Fig. 24. Crystal structure of **Co-bpq**. Three $[PF_6]^-$ anions, the CH_2Cl_2 solvent molecules and the hydrogen atoms are not shown. Displacement ellipsoids are shown at 50 % probability level at 100 K.

Supplementary Table 3. Selected crystallographic data and details of the structure determination for $C_{39}H_{33}CoN_7(PF_6)_2 \cdot 2CH_2Cl_2$.

Identification code	**Co-bpq**
Empirical formula	$C_{41}H_{37}Cl_4CoF_{12}N_7P_2$
Molar mass / g·mol^{-1}	1118.44
Space group (No.)	$P\bar{1}$ (2)
a / Å	9.6699(2)
b / Å	12.1699(2)
c / Å	19.7138(3)
α / °	78.2490(10)
β / °	84.8120(10)
γ / °	79.7910(10)
V / Å^3	2231.82(7)
Z	2
$\rho_{calc.}$ / g·cm^{-3}	1.664
μ / mm^{-1}	6.723
Color	red
Crystal habitus	block
Crystal size / mm^3	0.173 x 0.094 x 0.070
T / K	100
λ / Å	1.54186 (Cu-K$_\alpha$)
θ range / °	3.760 to 76.316
Range of Miller indices	$-11 \leq h \leq 12$
	$-15 \leq k \leq 15$
	$-24 \leq l \leq 13$
Absorption correction	multi-scan and numerical
T_{min}, T_{max}	0.3842, 0.6790
R_{int}, R_σ	0.0440, 0.0326
Completeness of the data set	0.991
No. of measured reflections	51040
No. of independent reflections	9097
No. of parameters	612
No. of restraints	0
S (all data)	1.068
$R(F)$ ($I \geq 2\sigma(I)$, all data)	0.0426, 0.0496
$wR(F^2)$ ($I \geq 2\sigma(I)$, all data)	0.1144, 0.1182
Extinction coefficient	0.00087(13)
$\Delta\rho_{\mathrm{max}}$, $\Delta\rho_{\mathrm{min}}$ / e·Å^{-3}	0.352, –0.352

11. Enantioselectivities as Determined by Chiral HPLC

Enantiomeric excess values of the products were determined with a Daicel Chiralpak OD-H or OJ-H (250 × 4.6 mm) HPLC columnon an Agilent 1200 Series HPLC System using *n*-hexane/isopropanol as mobile phase, the column temperature was 25 °C, and UV-absorption was measured at 254 nm.

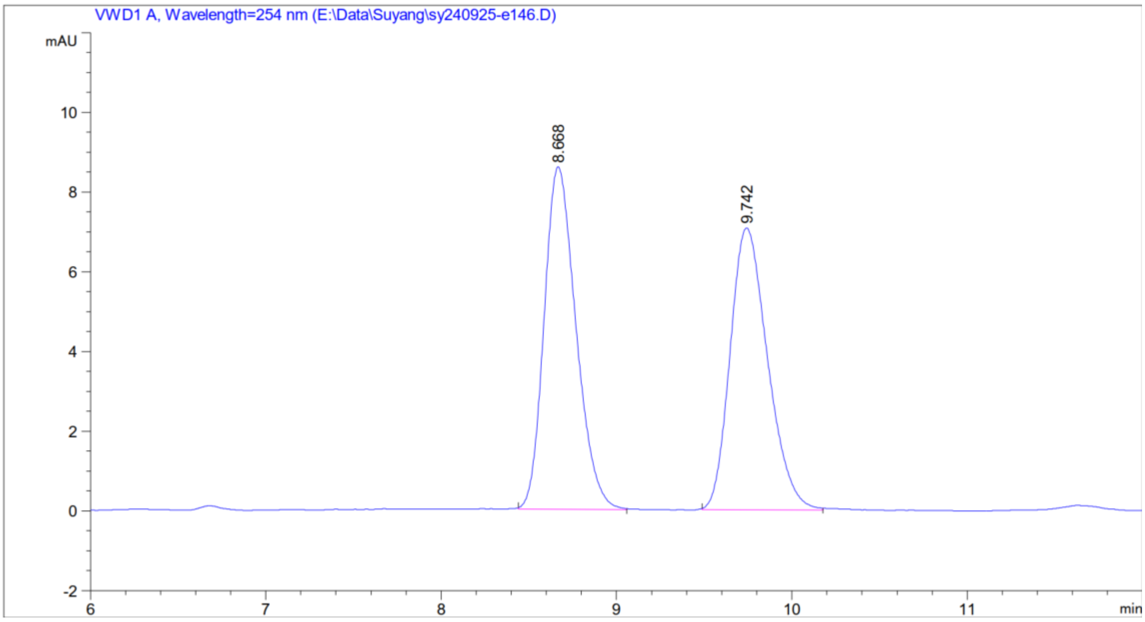

Peak #	RetTime [min]	Type	Width [min]	Area [mAU*s]	Height [mAU]	Area %
1	8.668	BB	0.1953	108.28378	8.57797	50.7673
2	9.742	BB	0.2294	105.01037	7.06723	49.2327

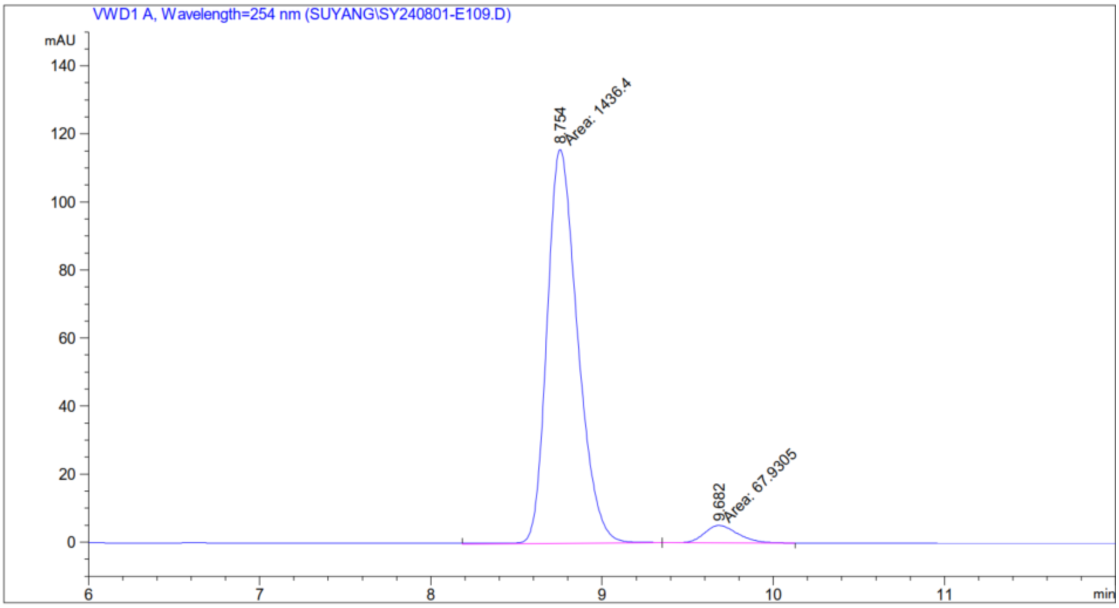

Peak #	RetTime [min]	Type	Width [min]	Area [mAU*s]	Height [mAU]	Area %
1	8.754	MM	0.2061	1436.40161	116.16174	95.4843
2	9.682	MM	0.2223	67.93049	5.09227	4.5157

Supplementary Fig. 25. HPLC traces of racemic **2** (reference) and enantioenriched **2**. Area integration = 95.5: 4.5 (91.0% ee)

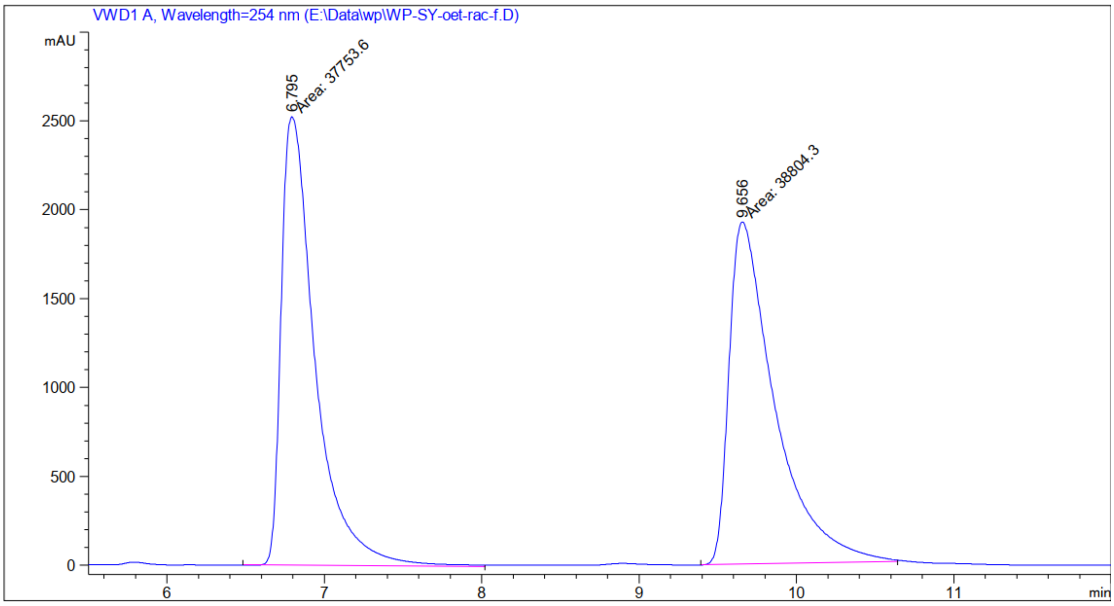

Peak #	RetTime [min]	Type	Width [min]	Area [mAU*s]	Height [mAU]	Area %
1	6.795	MM	0.2494	3.77536e4	2523.31323	49.3138
2	9.656	MM	0.3361	3.88043e4	1924.46985	50.6862

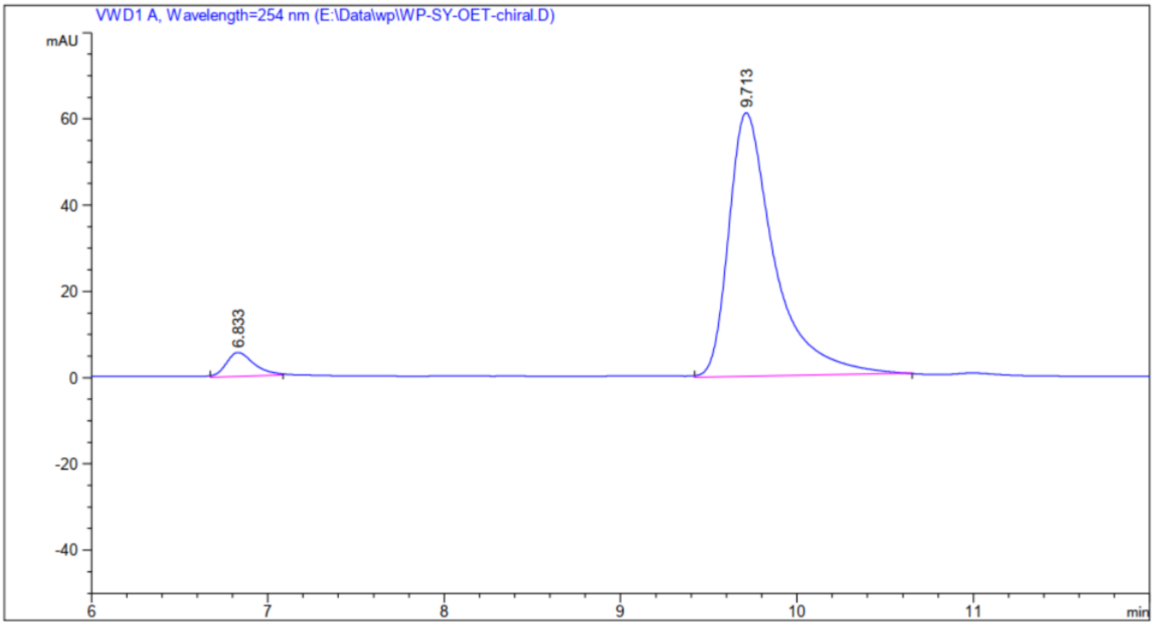

Peak #	RetTime [min]	Type	Width [min]	Area [mAU*s]	Height [mAU]	Area %
1	6.833	MM R	0.1839	61.21185	5.54895	5.3010
2	9.713	MM R	0.2982	1093.50122	61.11850	94.6990

Supplementary Fig. 26. HPLC traces of racemic **2b** (reference) and enantioenriched **2b**. Area integration = 94.7: 5.3 (89.4% ee)

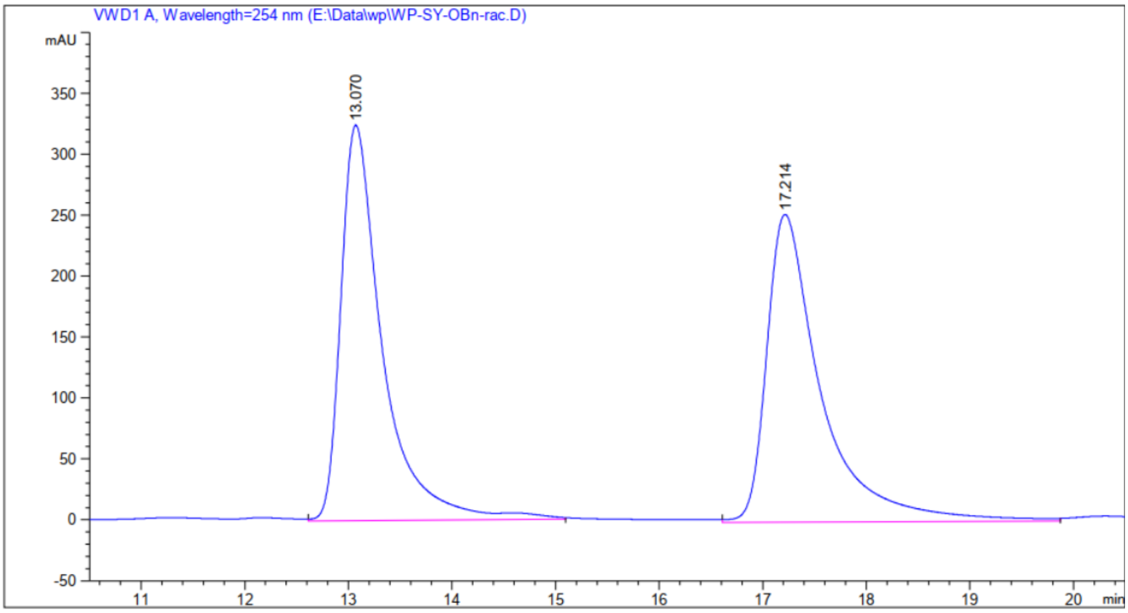

Peak #	RetTime [min]	Type	Width [min]	Area [mAU*s]	Height [mAU]	Area %
1	13.070	MM R	0.4673	9096.76660	324.45779	49.6692
2	17.214	MM R	0.6087	9217.94434	252.38989	50.3308

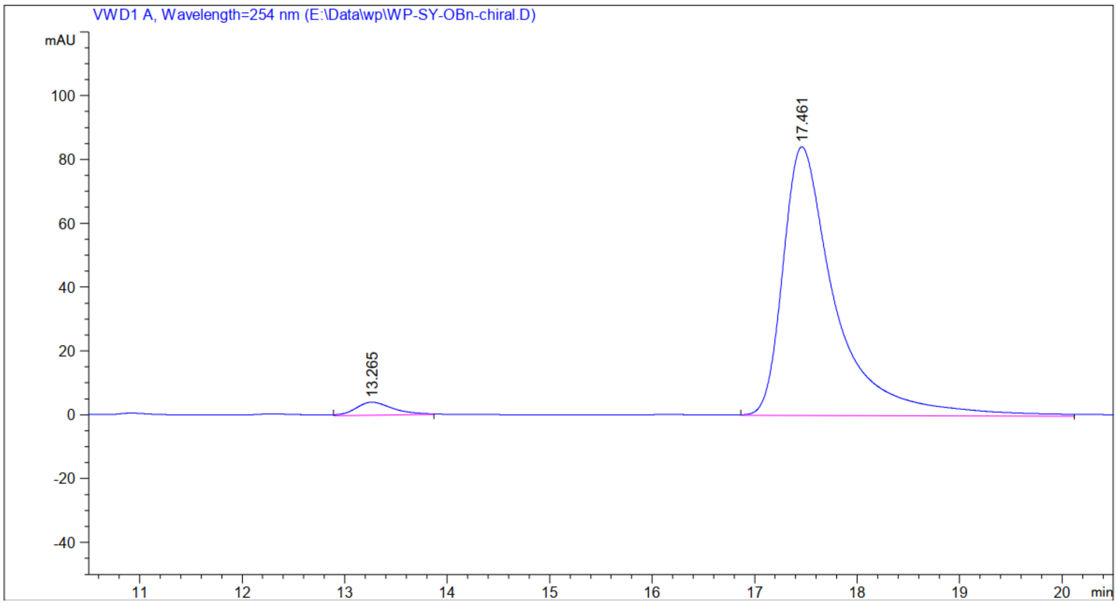

Peak #	RetTime [min]	Type	Width [min]	Area [mAU*s]	Height [mAU]	Area %
1	13.265	MM R	0.4233	103.94934	4.09310	3.3342
2	17.461	MM R	0.5965	3013.67847	84.20273	96.6658

Supplementary Fig. 27. HPLC traces of racemic **2c** (reference) and enantioenriched **2c**. Area integration = 96.7: 3.3 (93.4% ee)

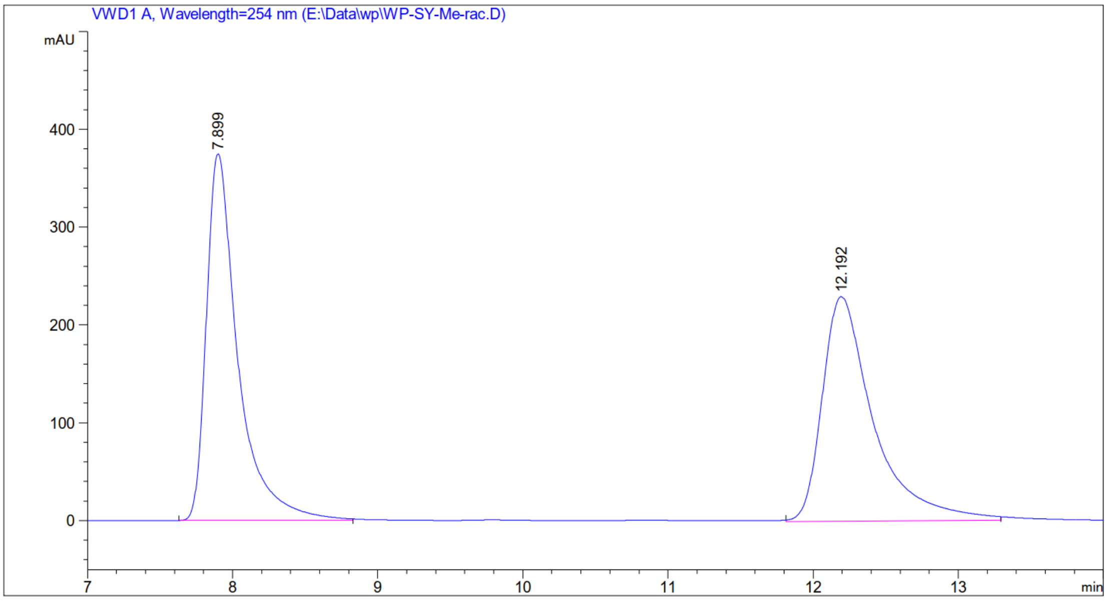

Peak #	RetTime [min]	Type	Width [min]	Area [mAU*s]	Height [mAU]	Area %
1	7.899	MM R	0.2508	5630.86475	374.19238	50.0434
2	12.192	MM R	0.4084	5621.09521	229.37410	49.9566

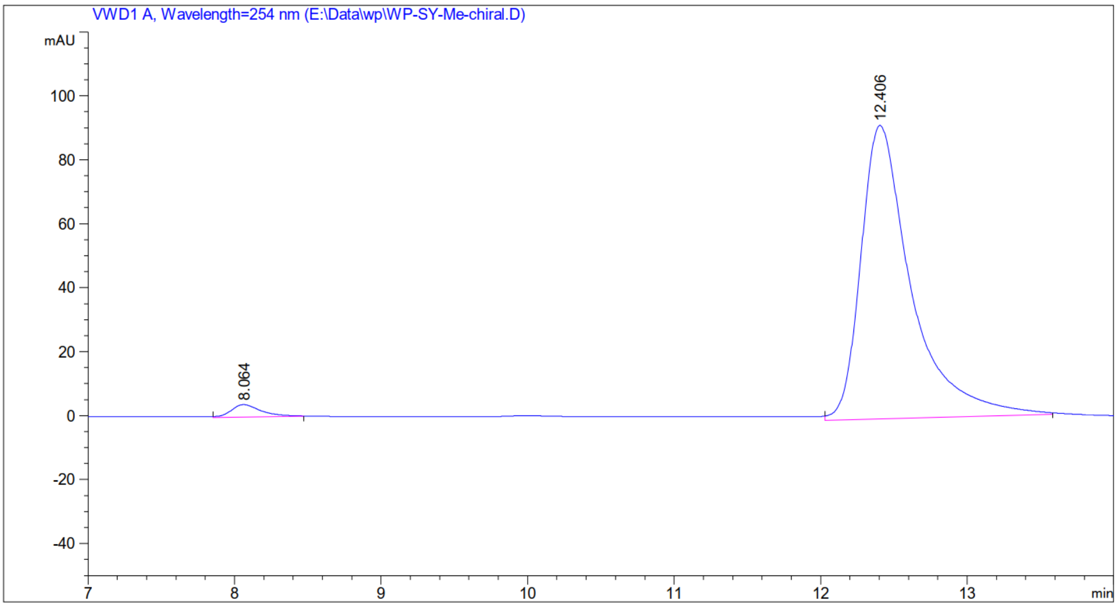

Peak #	RetTime [min]	Type	Width [min]	Area [mAU*s]	Height [mAU]	Area %
1	8.064	MM R	0.2392	55.74791	3.88502	2.4717
2	12.406	MM R	0.3992	2199.70386	91.84200	97.5283

Supplementary Fig. 28. HPLC traces of racemic **2d** (reference) and enantioenriched **2d**. Area integration = 97.5: 2.5 (95.0% ee)

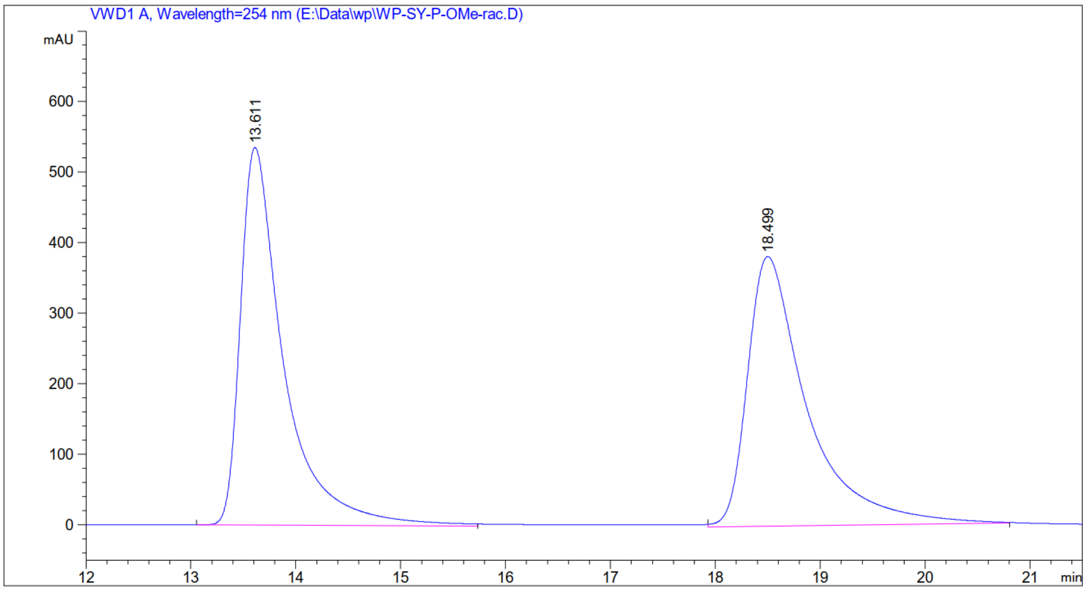

Peak #	RetTime [min]	Type	Width [min]	Area [mAU*s]	Height [mAU]	Area %
1	13.611	MM R	0.4779	1.53565e4	535.51605	50.4069
2	18.499	MM R	0.6586	1.51086e4	382.33432	49.5931

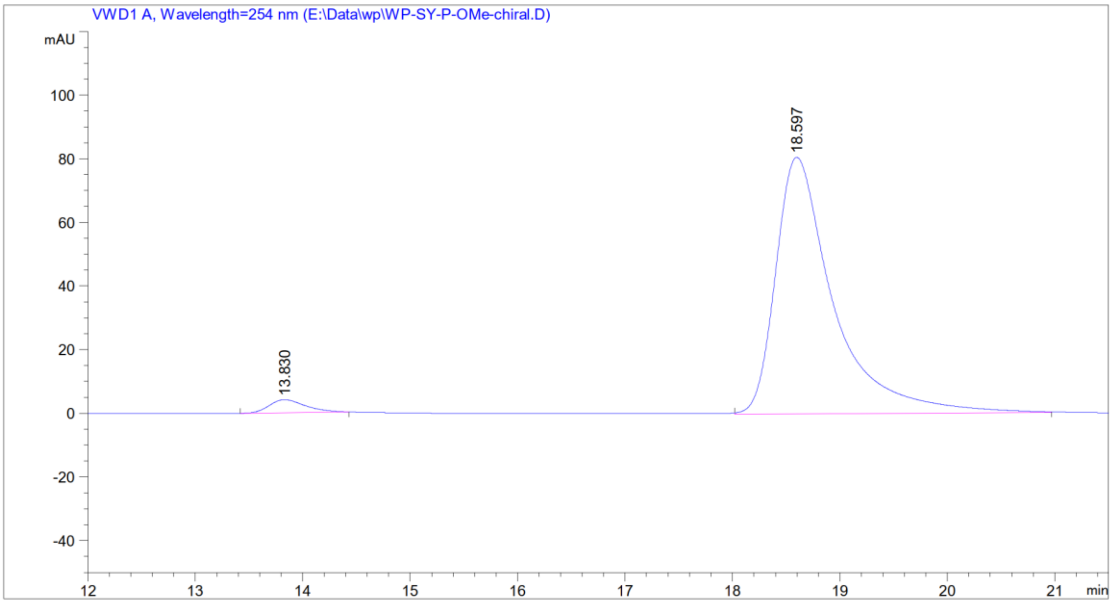

Peak #	RetTime [min]	Type	Width [min]	Area [mAU*s]	Height [mAU]	Area %
1	13.830	MM R	0.3934	97.02509	4.11067	3.1198
2	18.597	MM R	0.6231	3012.94629	80.59406	96.8802

Supplementary Fig. 29. HPLC traces of racemic **2e** (reference) and enantioenriched **2e**. Area integration = 96.9: 3.1 (93.8% ee)

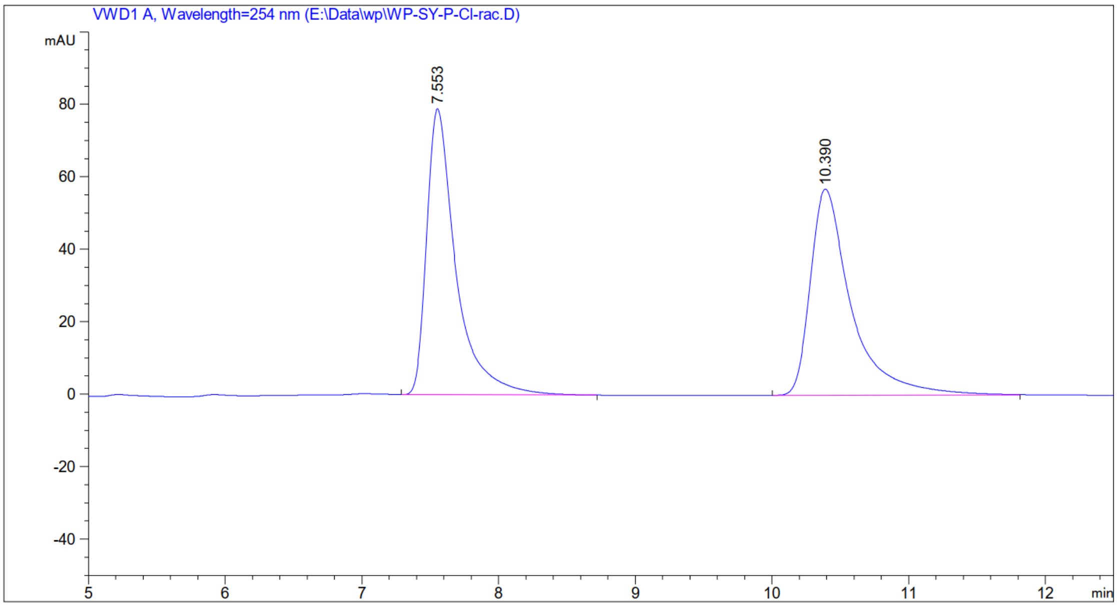

Peak #	RetTime [min]	Type	Width [min]	Area [mAU*s]	Height [mAU]	Area %
1	7.553	MM R	0.2473	1172.49695	79.00941	49.7929
2	10.390	MM R	0.3459	1182.25232	56.96378	50.2071

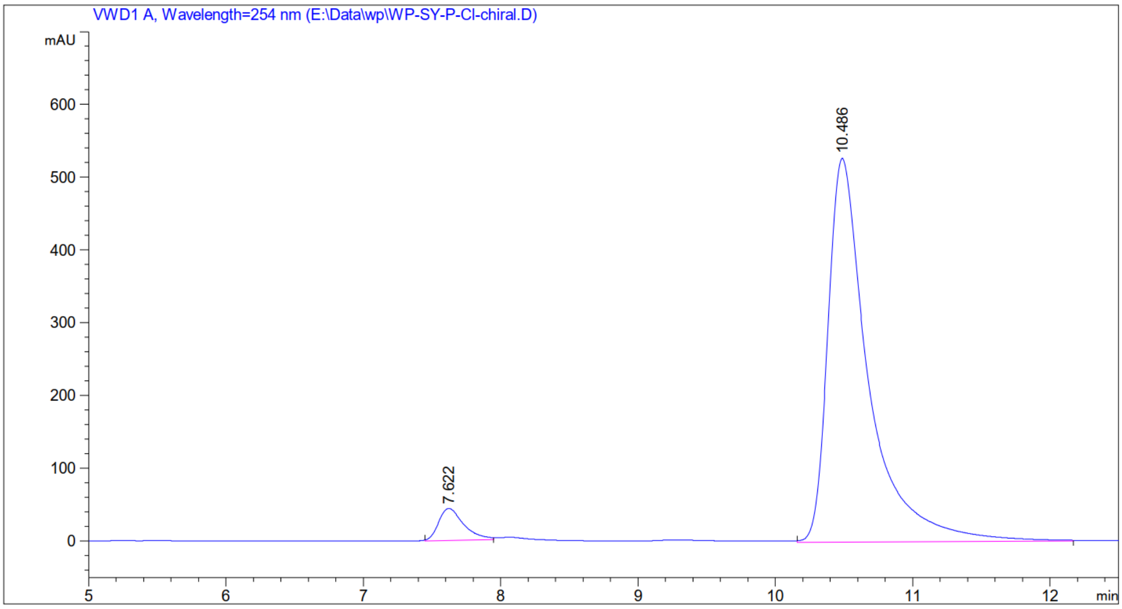

Peak #	RetTime [min]	Type	Width [min]	Area [mAU*s]	Height [mAU]	Area %
1	7.622	MM R	0.2114	559.11560	44.07424	4.9007
2	10.486	MM R	0.3425	1.08498e4	527.95245	95.0993

Supplementary Fig. 30. HPLC traces of racemic **2f** (reference) and enantioenriched **2f**. Area integration = 95.1: 4.9 (90.2% ee)

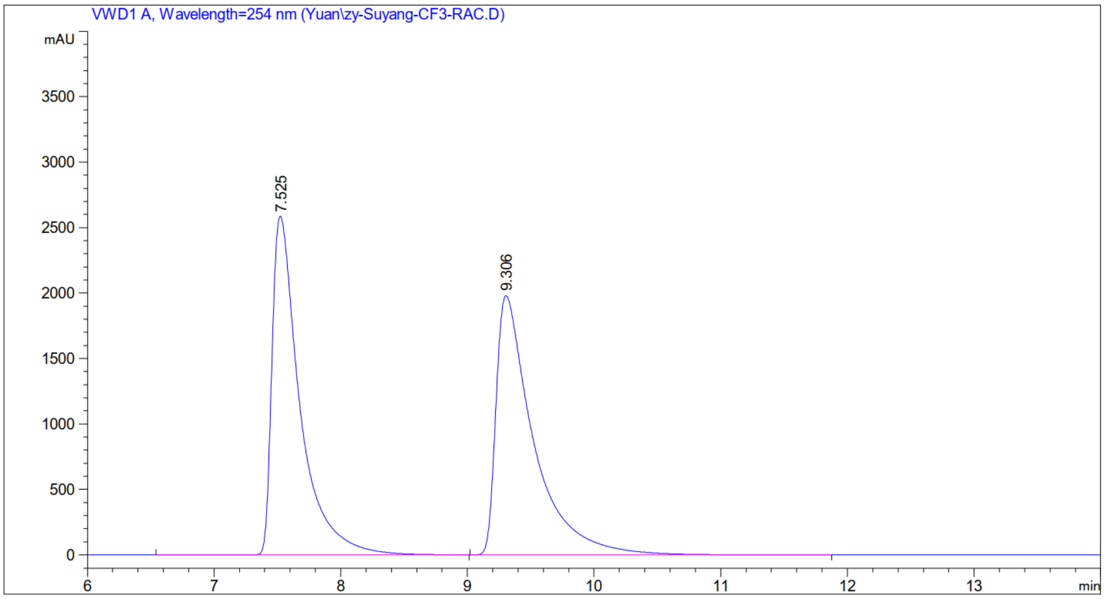

Peak #	RetTime [min]	Type	Width [min]	Area [mAU*s]	Height [mAU]	Area %
1	7.525	VB R	0.2245	3.94117e4	2584.05200	49.2393
2	9.306	BB	0.2946	4.06294e4	1981.21667	50.7607

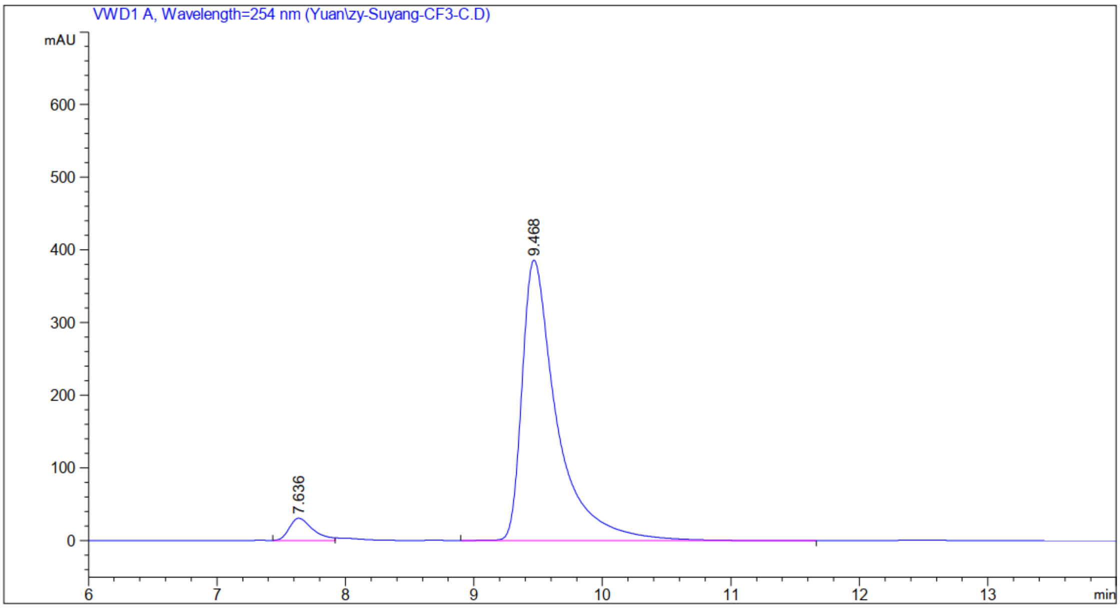

Peak #	RetTime [min]	Type	Width [min]	Area [mAU*s]	Height [mAU]	Area %
1	7.636	MF R	0.2155	400.36813	30.96995	5.2015
2	9.468	BB	0.2759	7296.87158	385.90768	94.7985

Supplementary Fig. 31. HPLC traces of racemic **2g** (reference) and enantioenriched **2g**. Area integration = 94.8: 5.2 (89.6% ee)

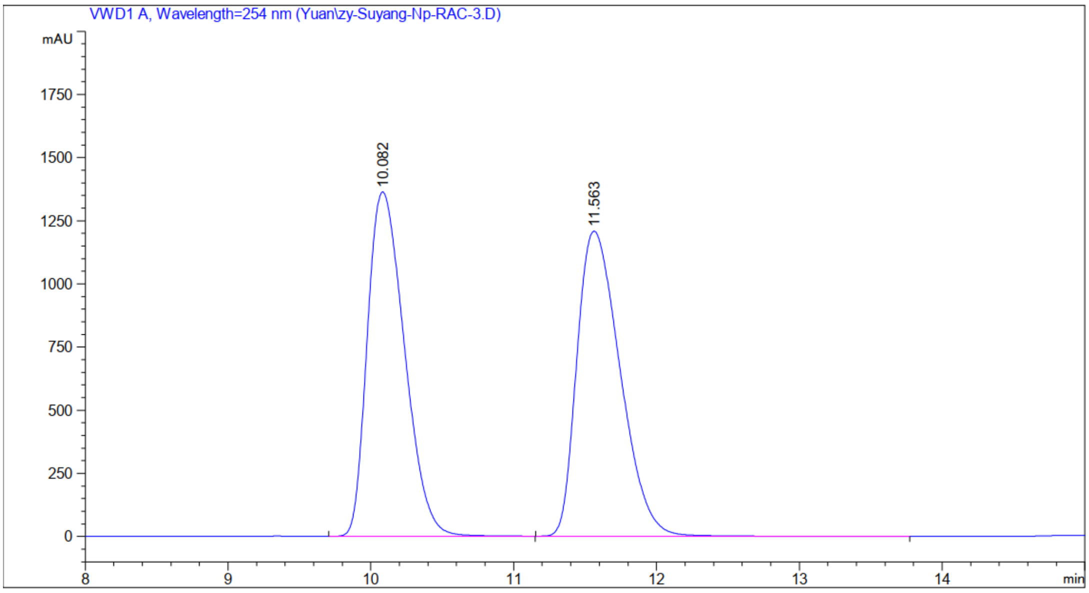

Peak #	RetTime [min]	Type	Width [min]	Area [mAU*s]	Height [mAU]	Area %
1	10.082	BB	0.2861	2.45964e4	1363.84497	49.2567
2	11.563	BB	0.3322	2.53387e4	1207.54028	50.7433

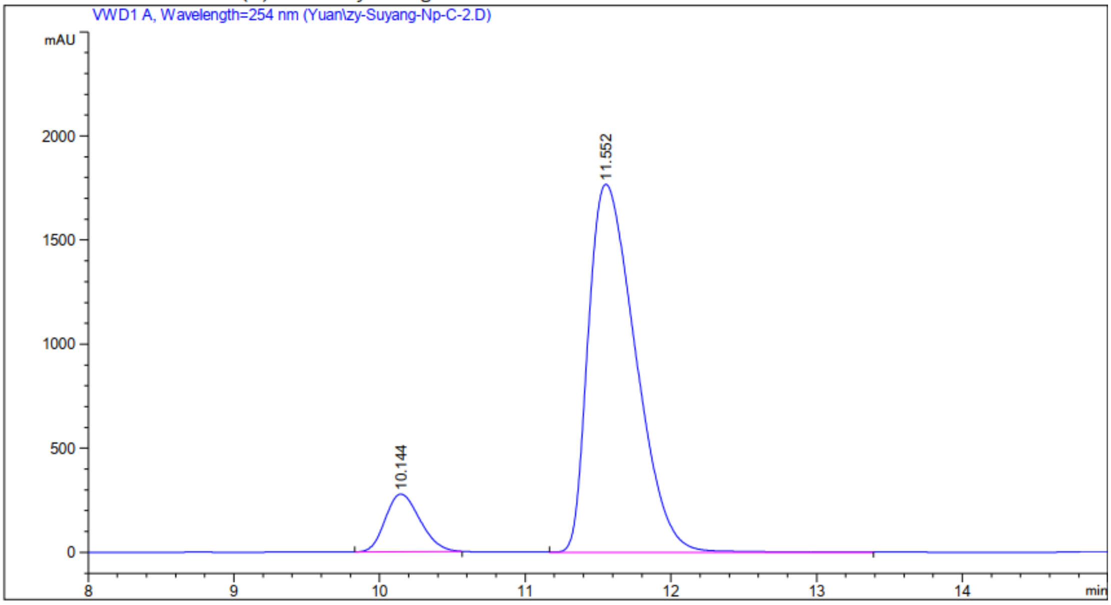

Peak #	RetTime [min]	Type	Width [min]	Area [mAU*s]	Height [mAU]	Area %
1	10.144	MM R	0.2761	4598.77832	277.62076	10.3911
2	11.552	MM R	0.3739	3.96582e4	1767.91321	89.6089

Supplementary Fig. 32. HPLC traces of racemic **2h** (reference) and enantioenriched **2h**. Area integration = 89.6: 10.4 (79.2% ee)

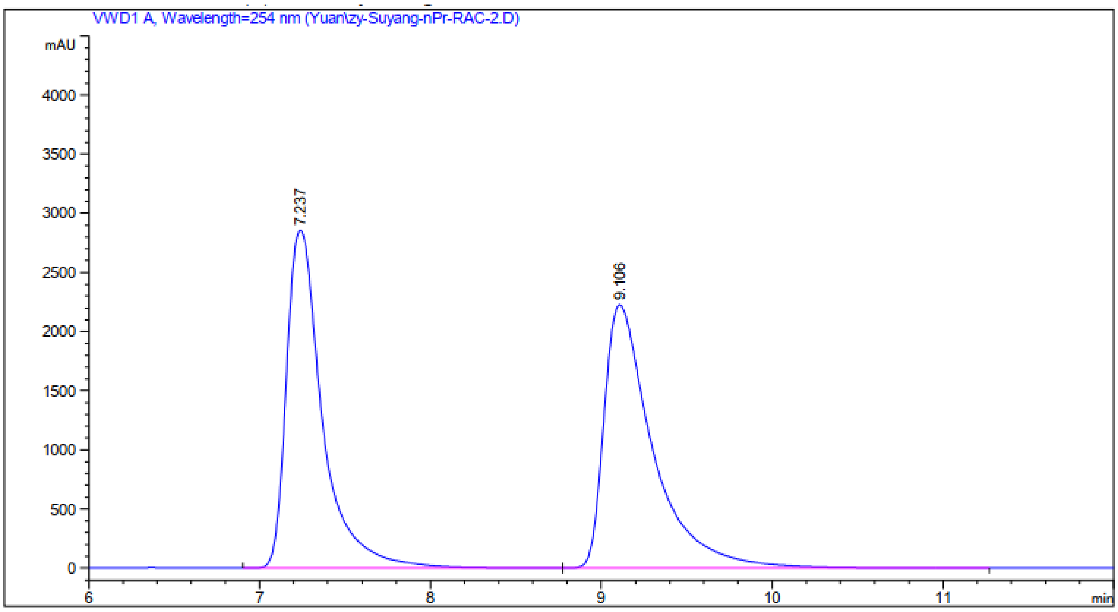

Peak #	RetTime [min]	Type	Width [min]	Area [mAU*s]	Height [mAU]	Area %
1	7.237	MF R	0.2445	4.19166e4	2857.07080	48.6307
2	9.106	FM R	0.3310	4.42770e4	2229.12427	51.3693

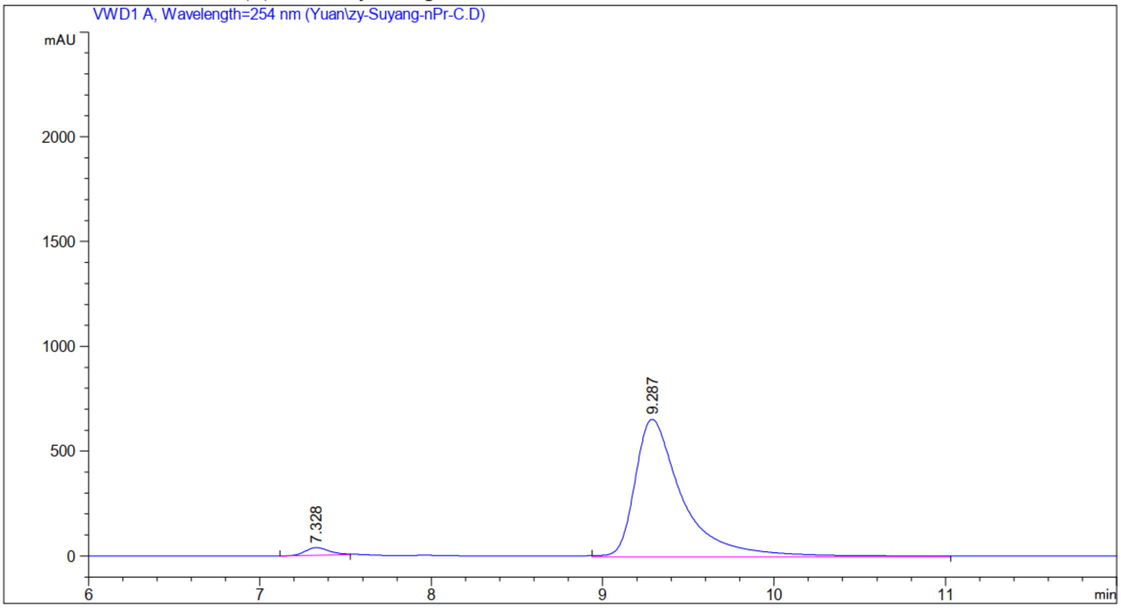

Peak #	RetTime [min]	Type	Width [min]	Area [mAU*s]	Height [mAU]	Area %
1	7.328	MM R	0.1634	355.84570	36.30398	2.6925
2	9.287	MM R	0.3263	1.28604e4	656.88342	97.3075

Supplementary Fig. 33. HPLC traces of racemic **2i** (reference) and enantioenriched **2i**. Area integration = 97.3: 2.7 (94.6% ee)

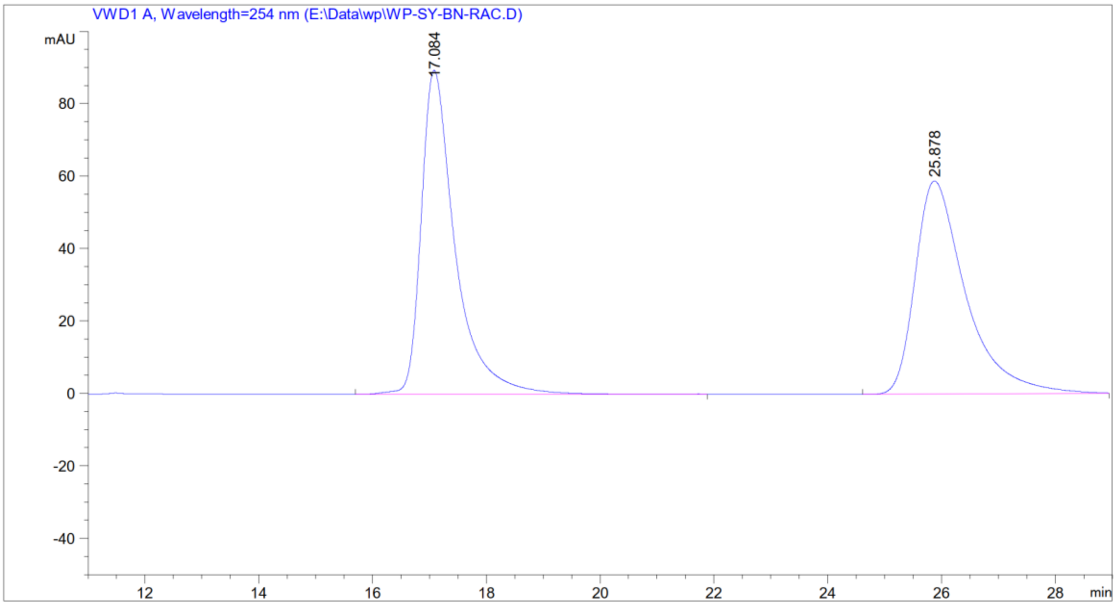

Peak #	RetTime [min]	Type	Width [min]	Area [mAU*s]	Height [mAU]	Area %
1	17.084	MM R	0.6976	3751.12817	89.61537	50.8598
2	25.878	BBA	0.9226	3624.29517	58.83786	49.1402

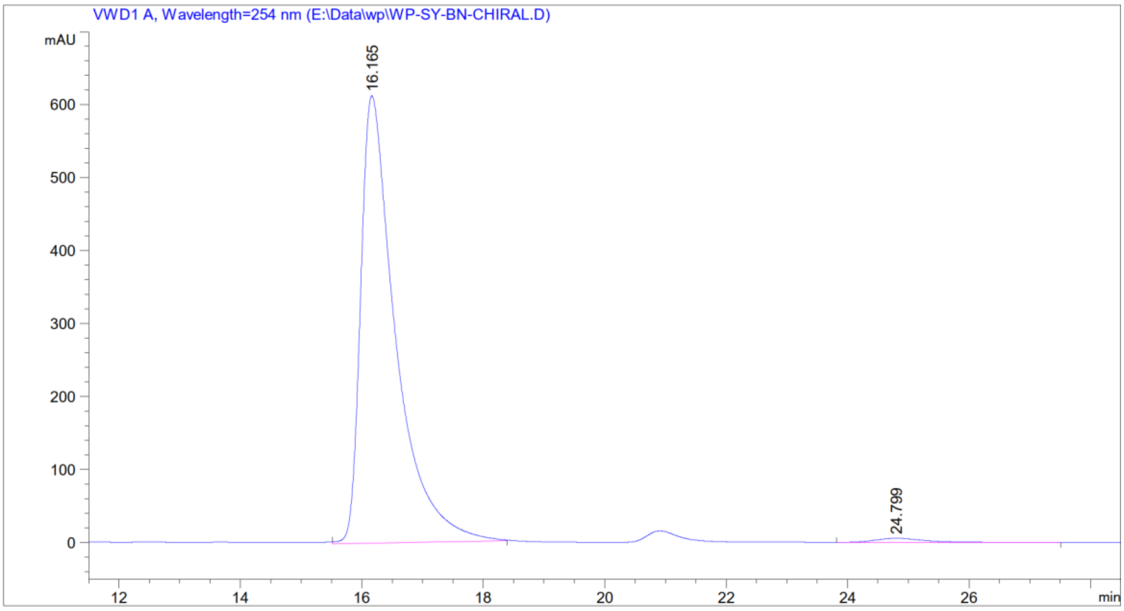

Peak #	RetTime [min]	Type	Width [min]	Area [mAU*s]	Height [mAU]	Area %
1	16.165	MM R	0.6656	2.45015e4	613.50940	98.6795
2	24.799	VB	0.8752	327.87820	5.69894	1.3205

Supplementary Fig. 34. HPLC traces of racemic **2j** (reference) and enantioenriched **2j**. Area integration = 98.7: 1.3 (97.4% ee)

12. CD Spectra of Chiral Cobalt Complexes

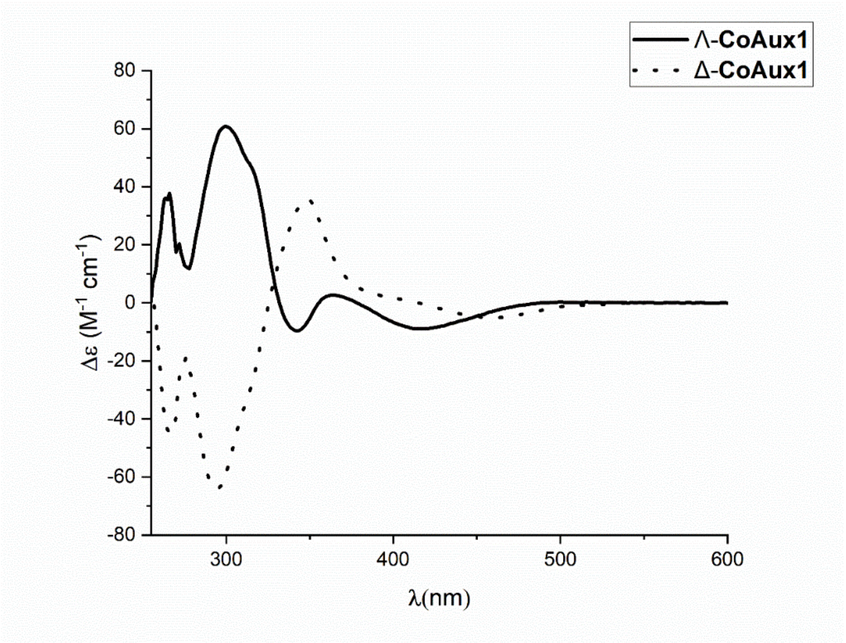

Supplementary Fig. 35. CD spectrum of auxiliary complex Λ-(*S*)-**CoAux1** and Δ-(*S*)-**CoAux1** recorded in CH_2Cl_2 (1.0 mM).

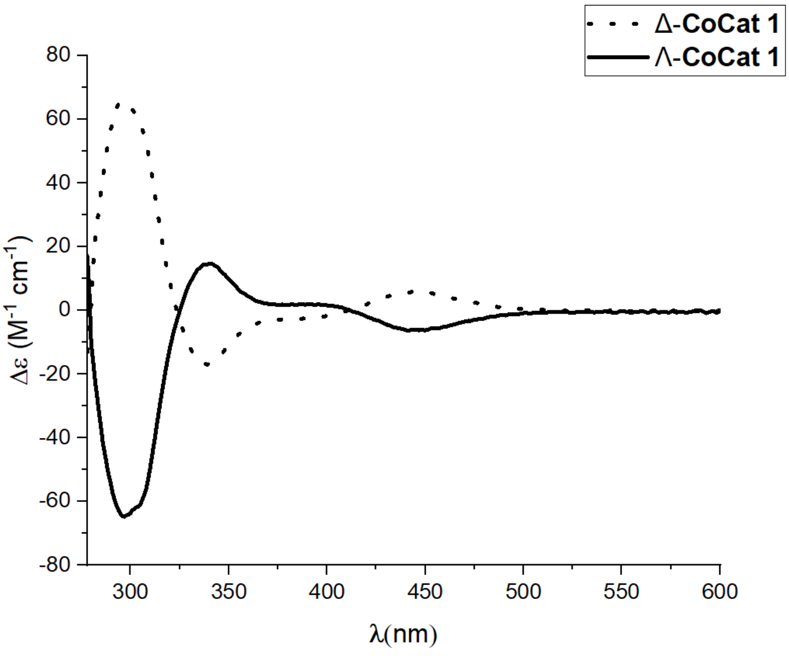

Supplementary Fig. 36. CD spectrum of complex Λ-**CoCat1** and **Δ-CoCat1** recorded in CH_2Cl_2 (1.0 mM).

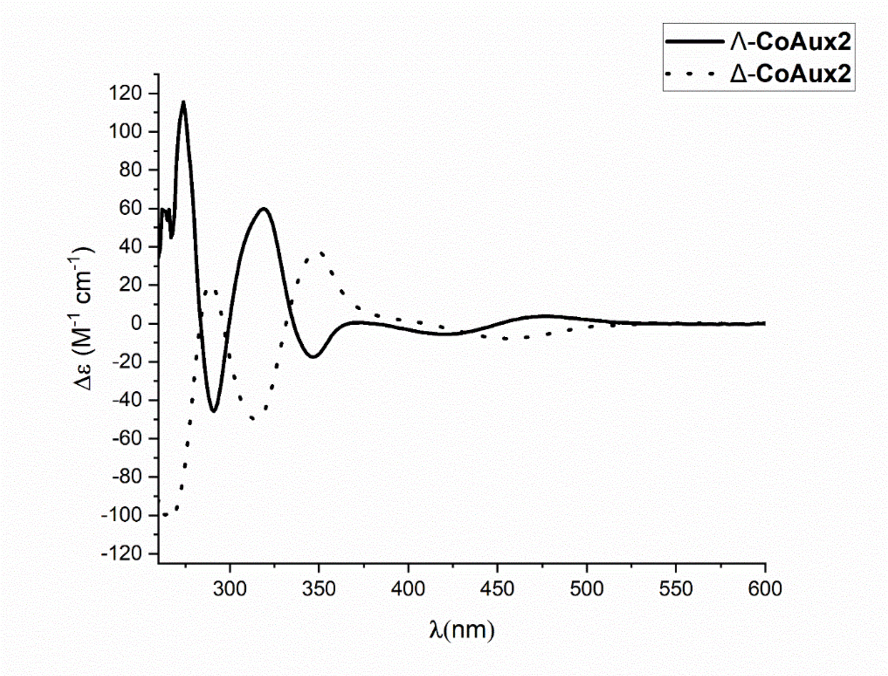

Supplementary Fig. 37. CD spectrum of auxiliary complex Λ-(*S*)-**CoAux2** and Δ-(*S*)-**CoAux2** recorded in CH_2Cl_2 (1.0 mM).

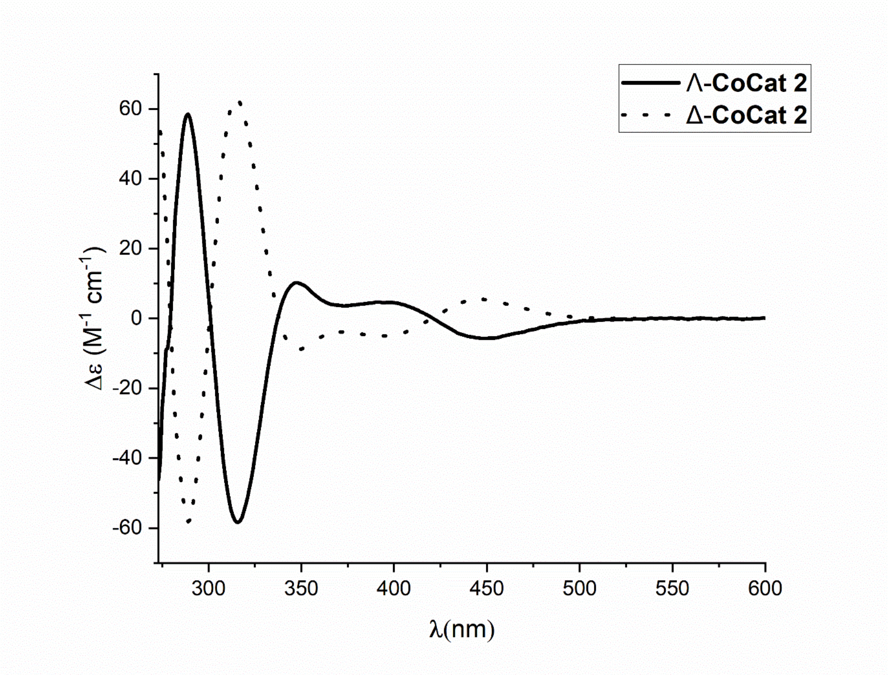

Supplementary Fig. 38. CD spectrum of complex Λ-**CoCat2** and Δ-**CoCat2** recorded in CH_2Cl_2 (1.0 mM).

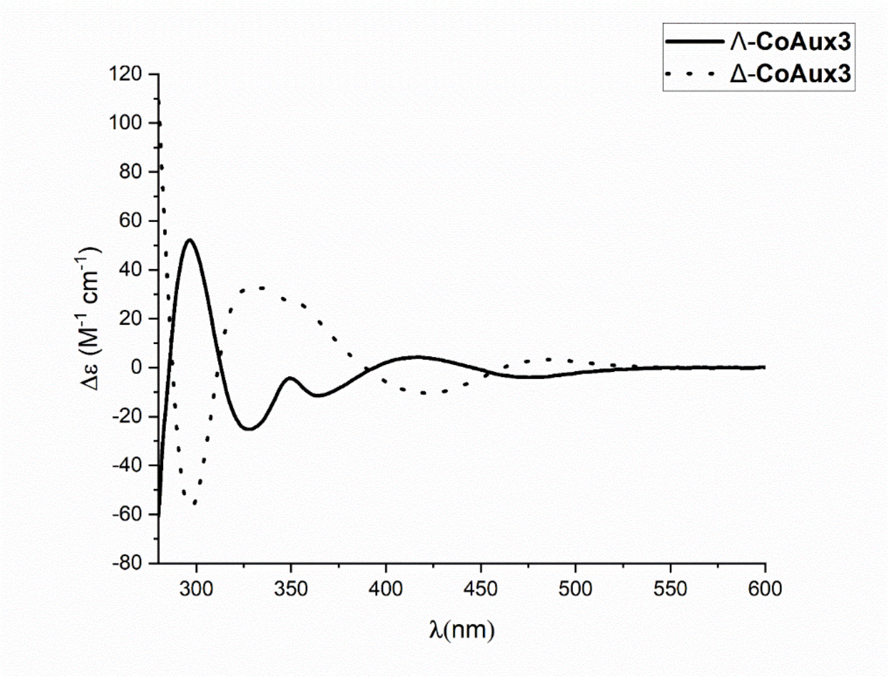

Supplementary Fig. 39. CD spectrum of auxiliary complex Λ-(*S*)-**CoAux3** and Δ-(*S*)-**CoAux3** recorded in CH_2Cl_2 (1.0 mM).

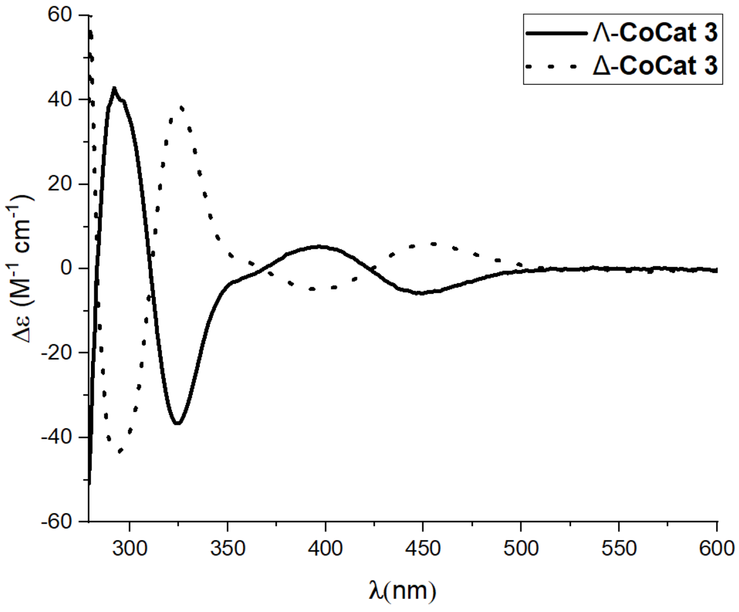

Supplementary Fig. 40. CD spectrum of complex **Λ-CoCat3** and **Δ-CoCat3** recorded in CH_2Cl_2 (1.0 mM).

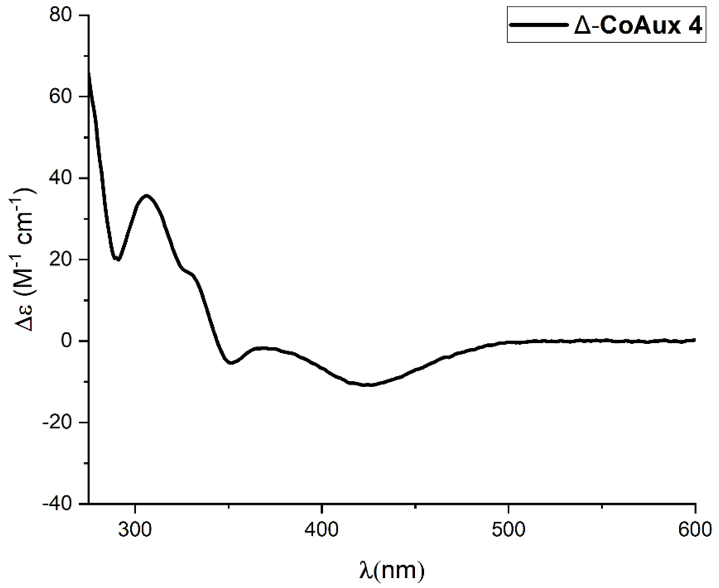

Supplementary Fig. 41. CD spectrum of auxiliary complex Δ-(*S*)-**CoAux4** recorded in CH_2Cl_2 (1.0 mM).

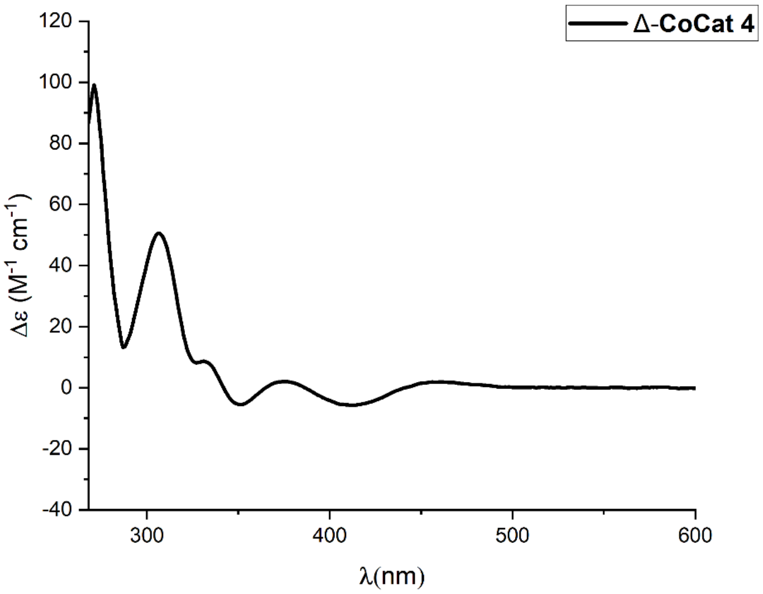

Supplementary Fig. 42. CD spectrum of complex **Δ-CoCat4** recorded in CH_2Cl_2 (1.0 mM).

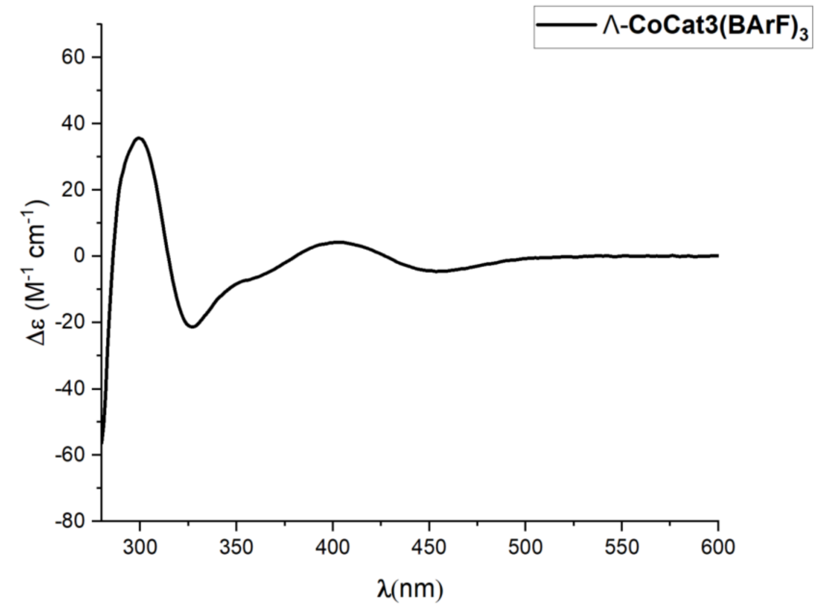

Supplementary Fig. 43. CD spectrum of complex **Λ-[CoCat3]**(BArF)$_3$ recorded in CH_2Cl_2 (1.0 mM).

13. NMR Spectra

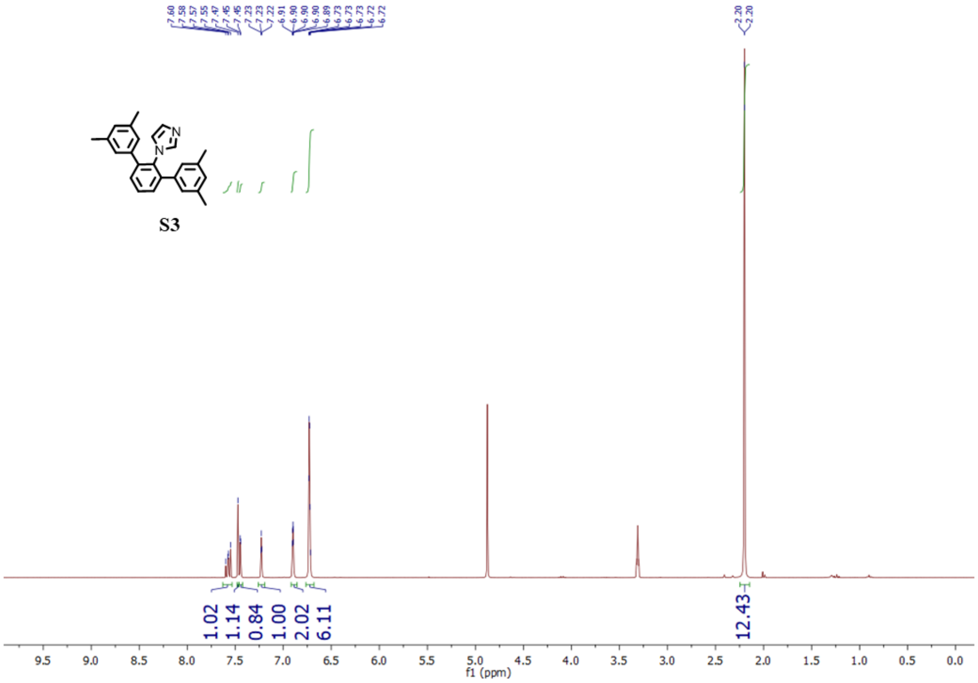

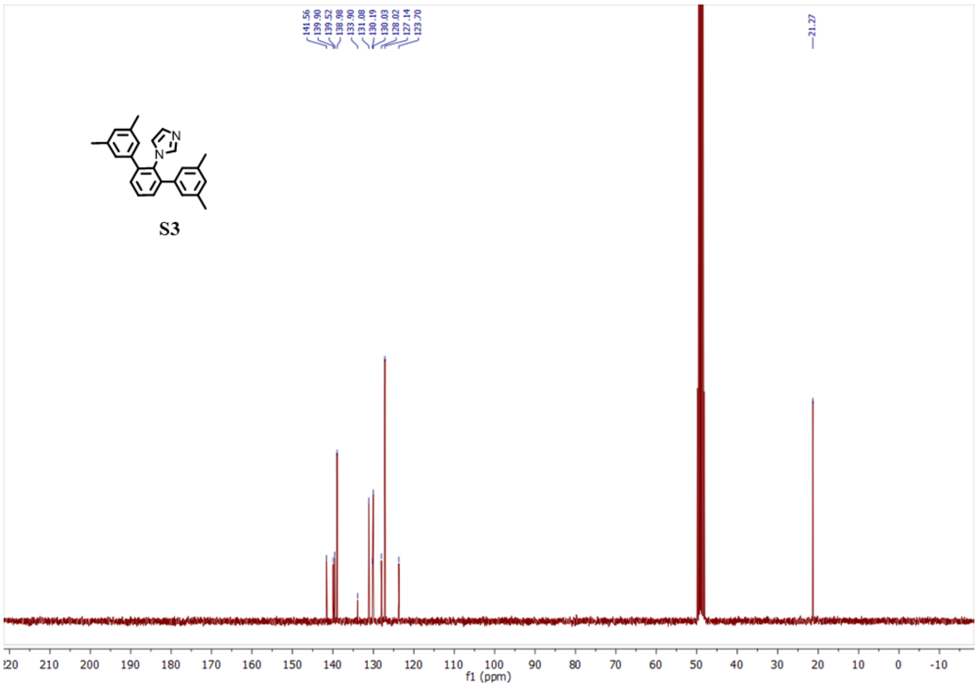

Supplementary Fig. 44. ^{1}H NMR (300 MHz, 298 K) and ^{13}C NMR (75 MHz, 298 K) spectra of **S3** in CD_3OD.

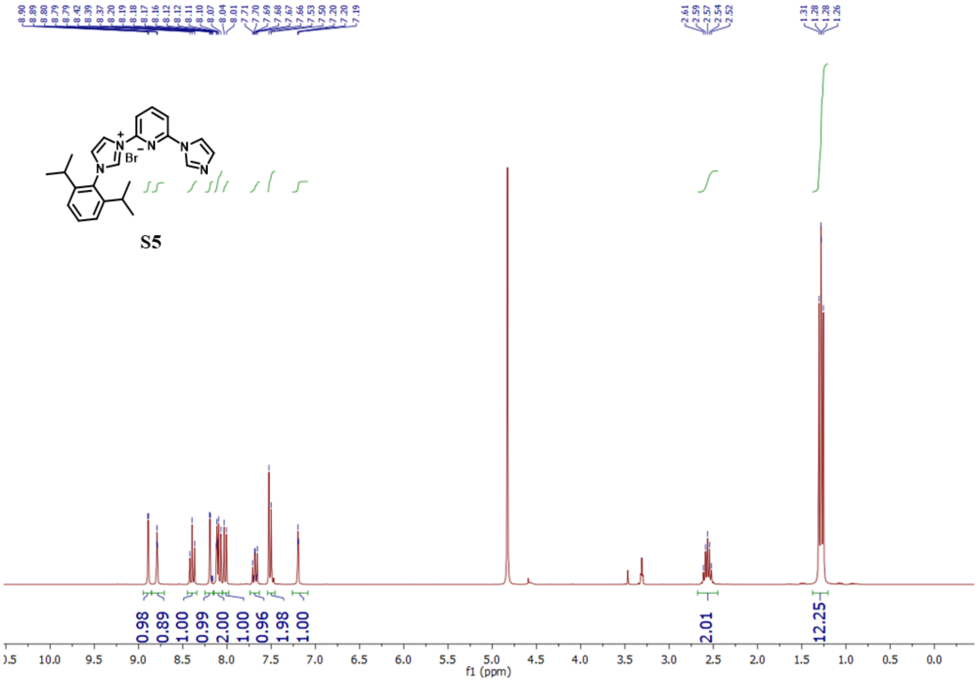

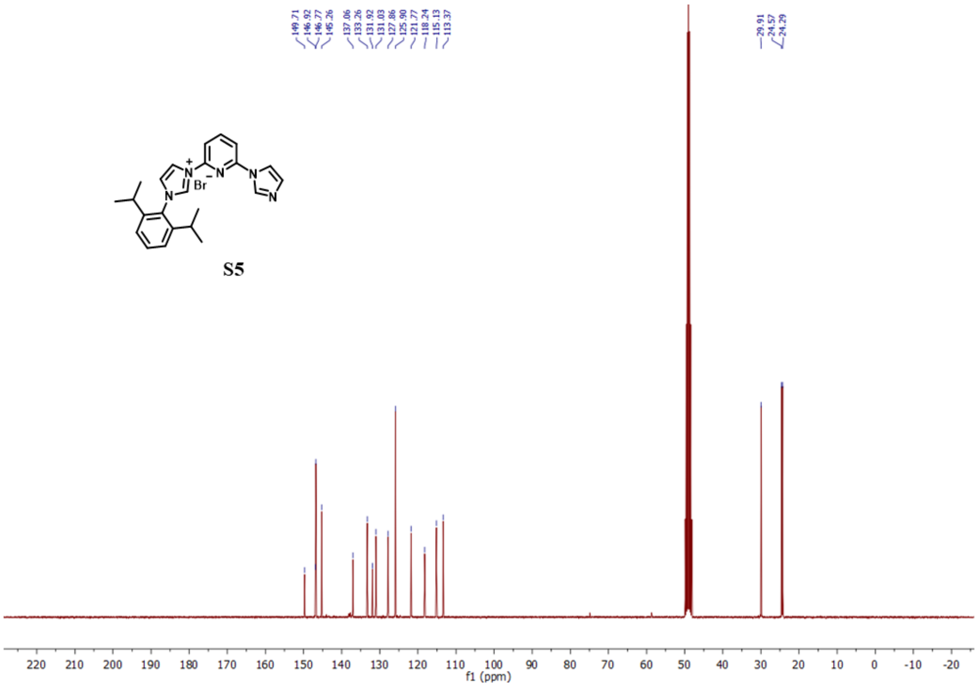

Supplementary Fig. 45. ^{1}H NMR (300 MHz, 298 K) and ^{13}C NMR (75 MHz, 298 K) spectra of **S5** in CD_3OD.

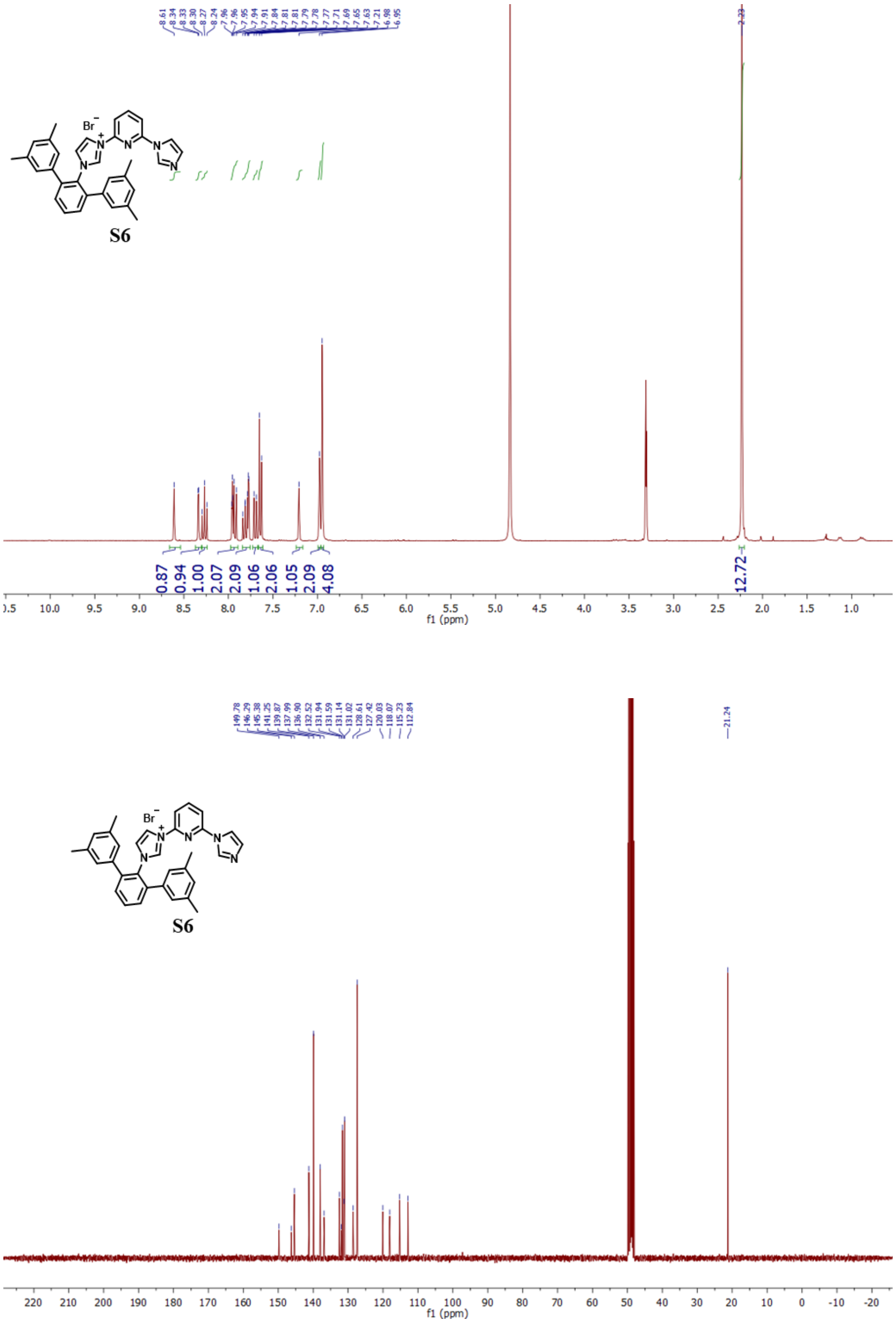

Supplementary Fig. 46. ^{1}H NMR (300 MHz, 298 K) and ^{13}C NMR (75 MHz, 298 K) spectra of **S6** in CD_3OD.

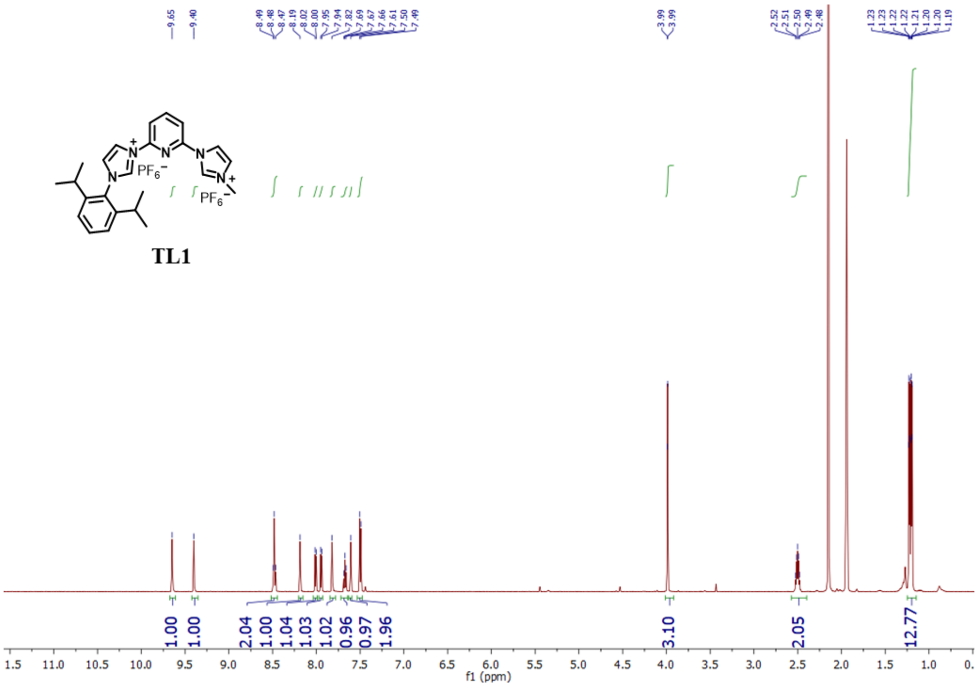
TL1
f1 (ppm)

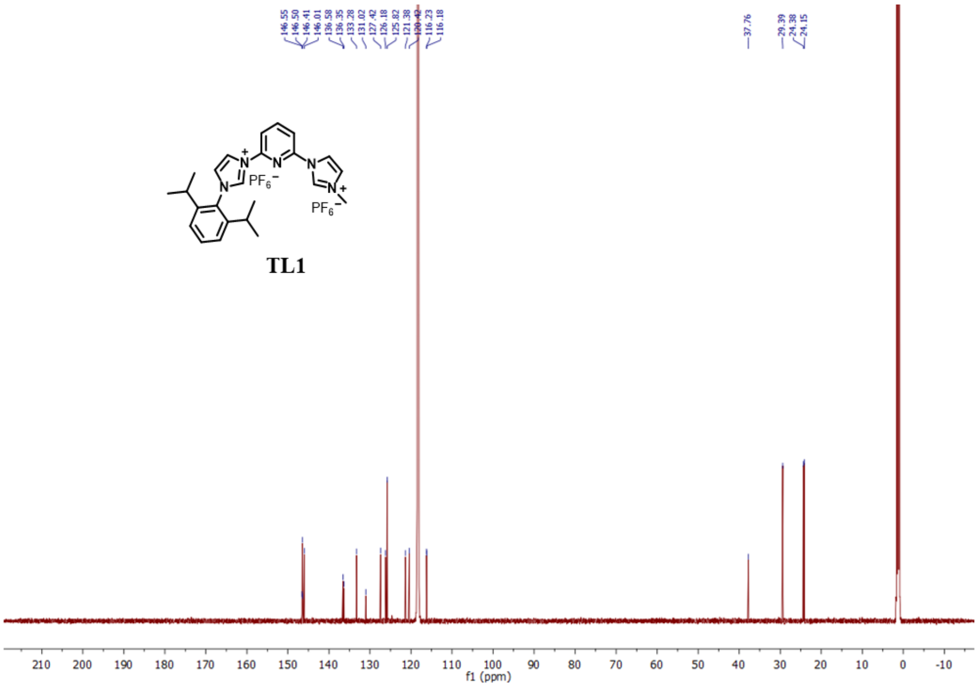
TL1
f1 (ppm)

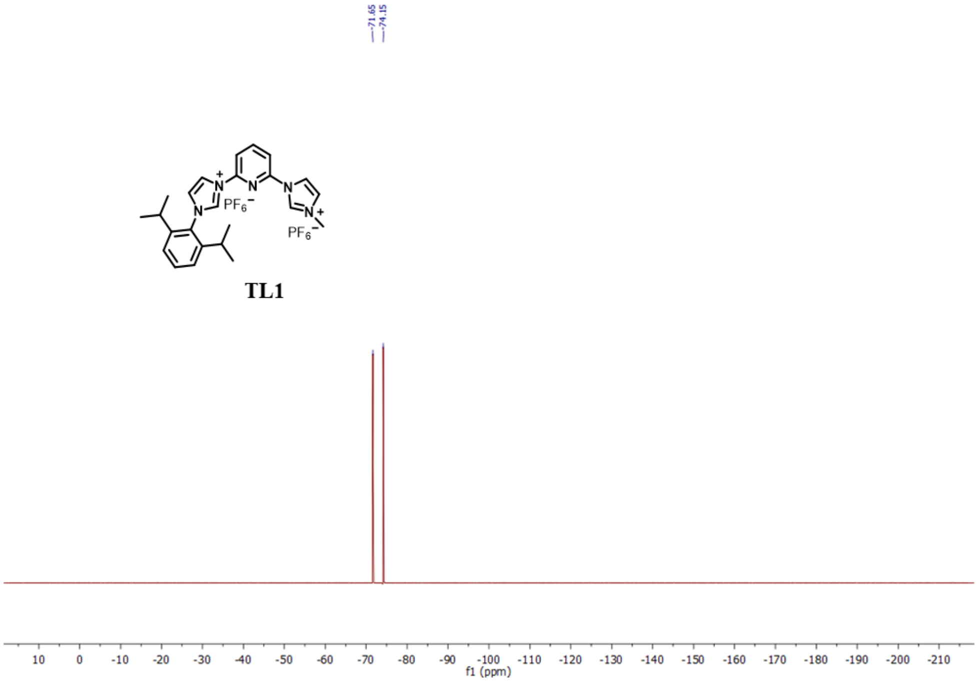

Supplementary Fig. 47. ^{1}H NMR (300 MHz, 298 K), ^{13}C NMR (75 MHz, 298 K) and ^{19}F NMR (282 MHz, 298 K) spectra of **TL1** in CD_3CN.

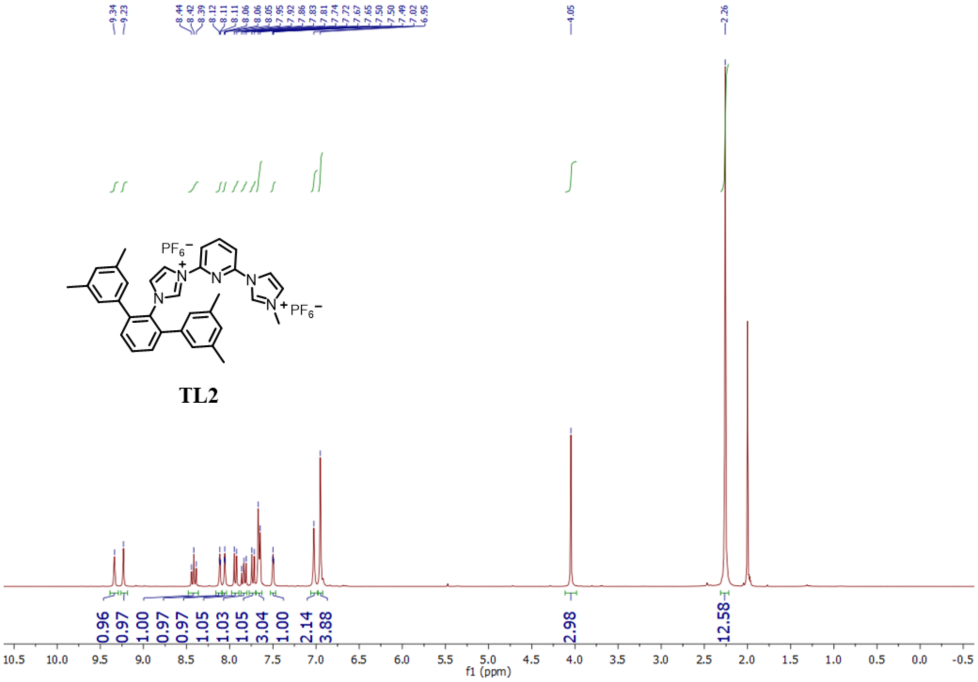

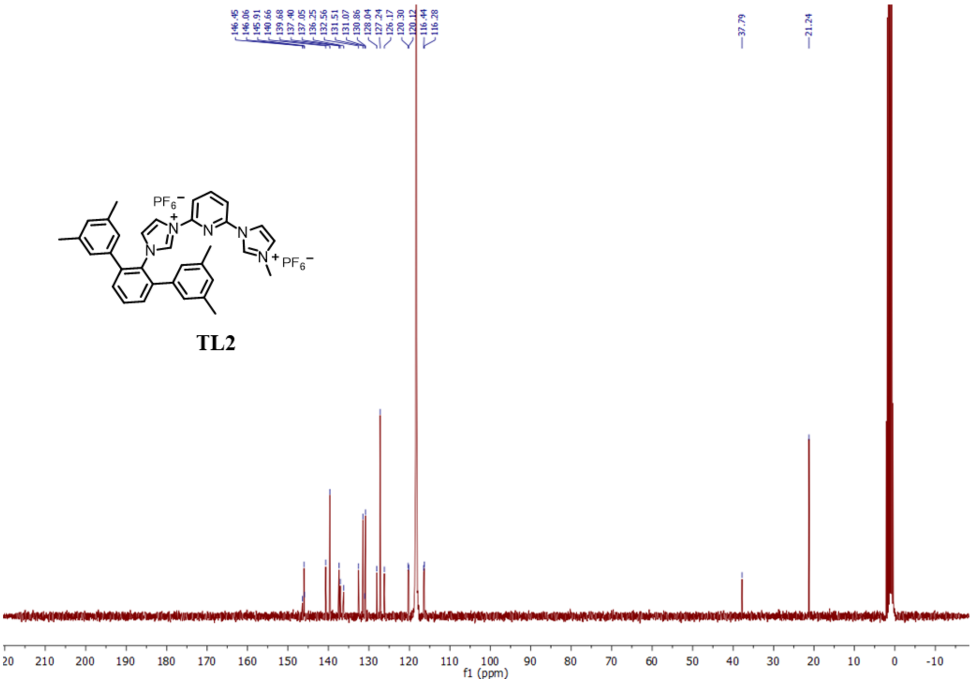

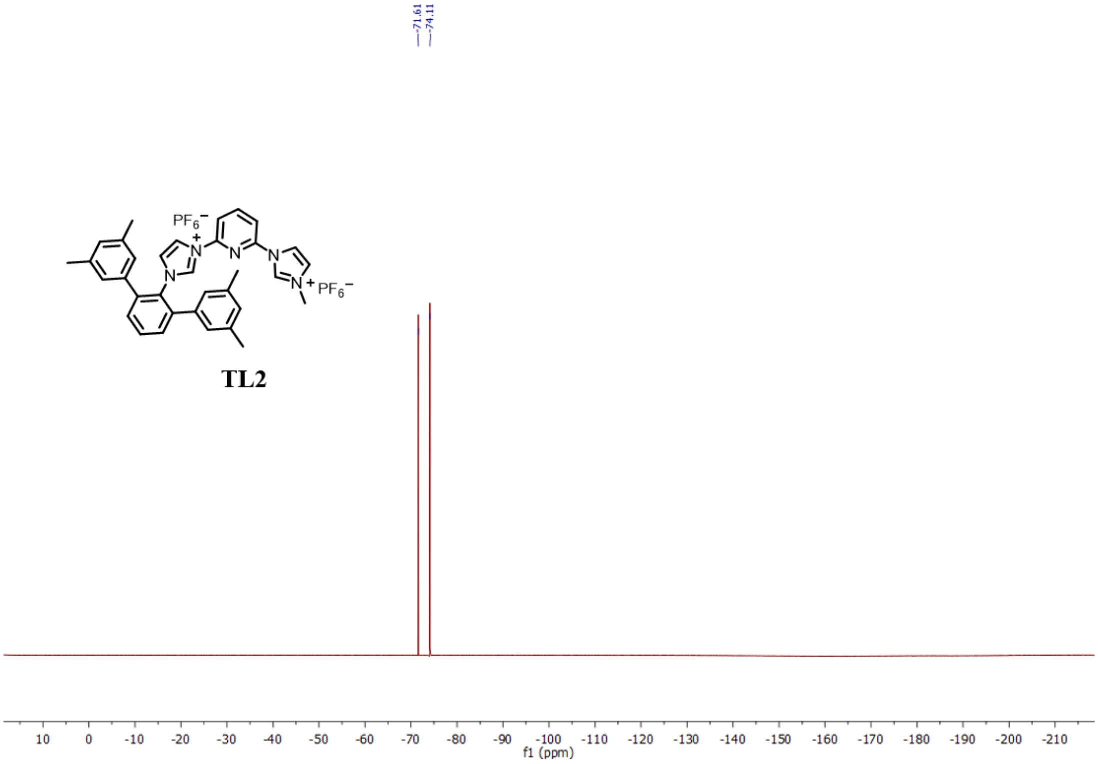

Supplementary Fig. 48. ^{1}H NMR (300 MHz, 298 K), ^{13}C NMR (75 MHz, 298 K) and ^{19}F NMR (282 MHz, 298 K) of **TL2** in CD_3CN.

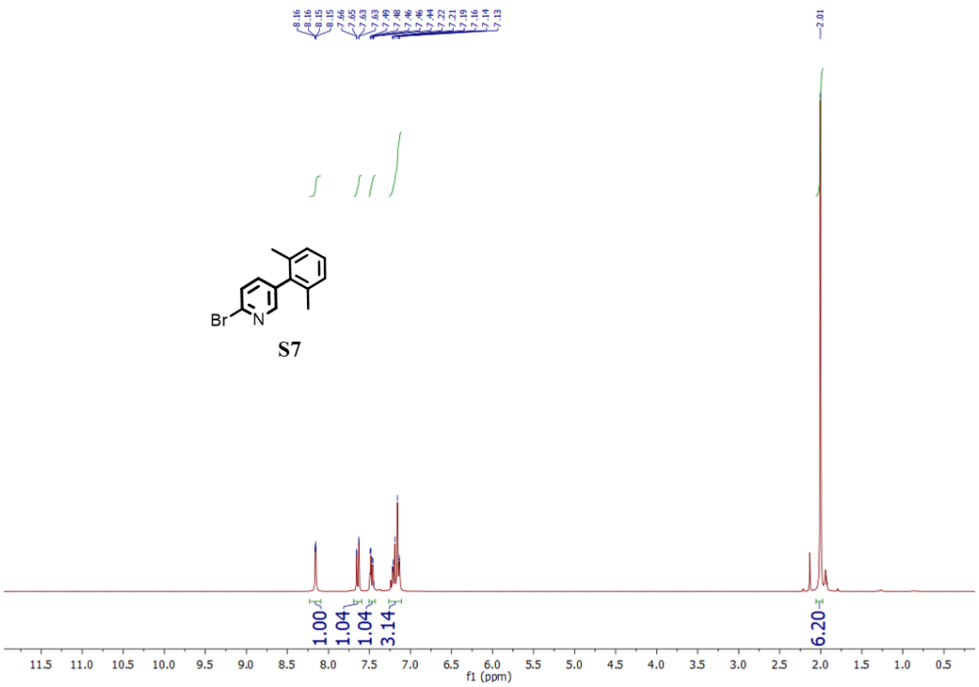

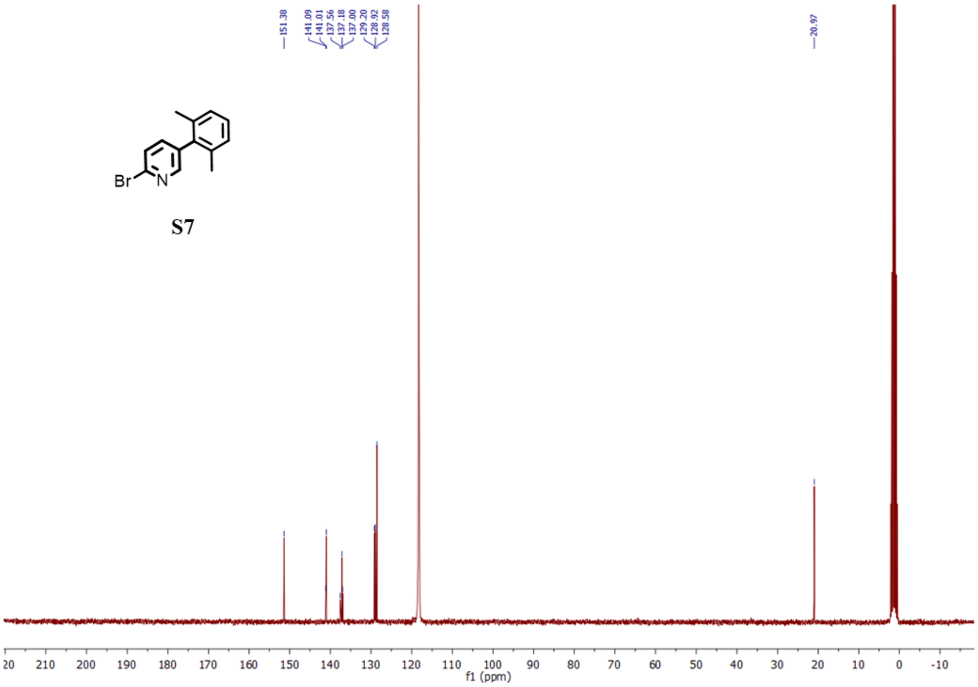

Supplementary Fig. 49. ^{1}H NMR (300 MHz, 298 K) and ^{13}C NMR (75 MHz, 298 K) spectra of **S7** in CD_3CN.

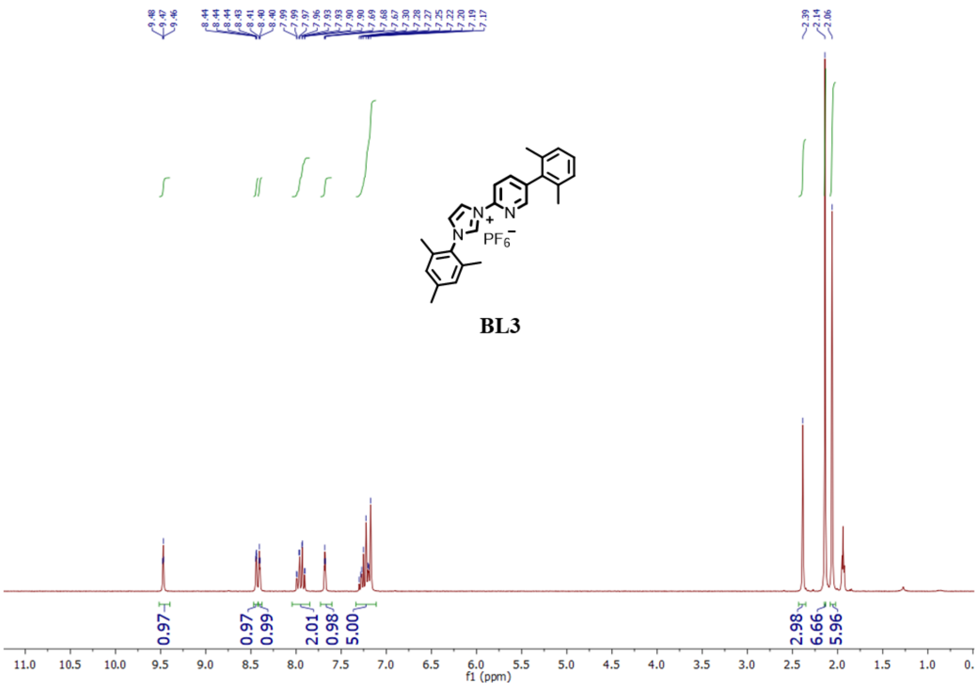

BL3
PF6
0.97
0.97
0.99
2.01
0.98
5.00
2.98
6.66
5.96
f1 (ppm)

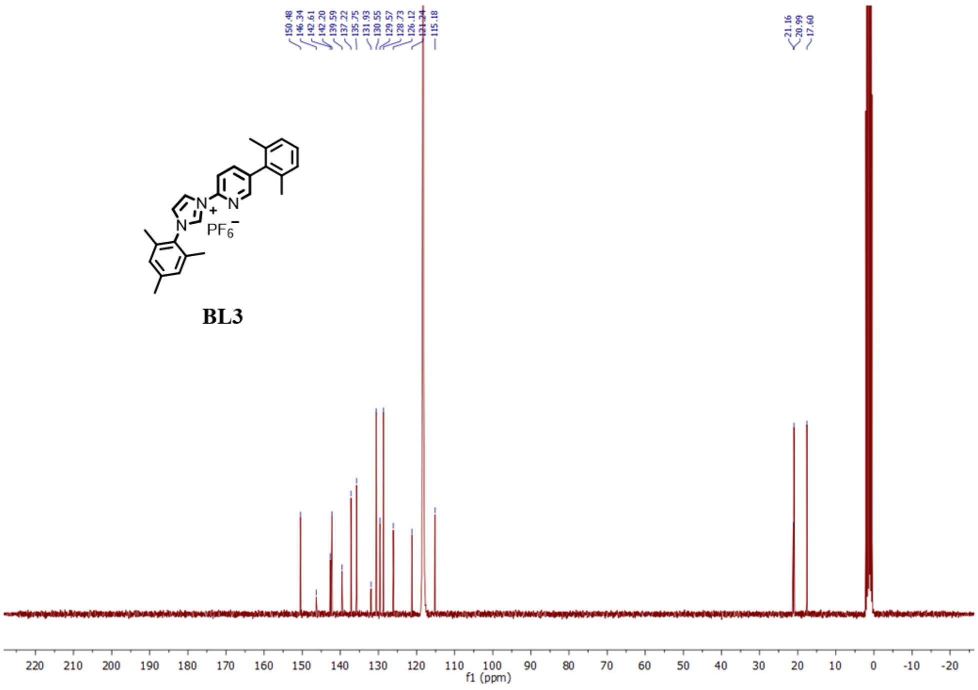

BL3
PF6
f1 (ppm)

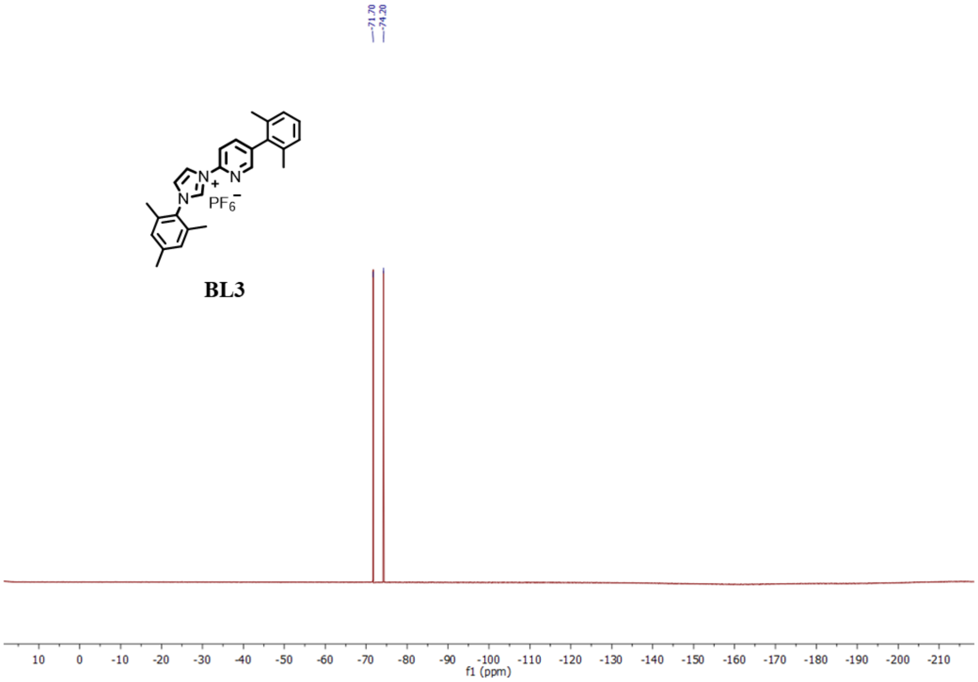

Supplementary Fig. 50. ^{1}H NMR (300 MHz, 298 K), ^{13}C NMR (75 MHz, 298 K) and ^{19}F NMR (282 MHz, 298 K) of **BL3** in CD_3CN.

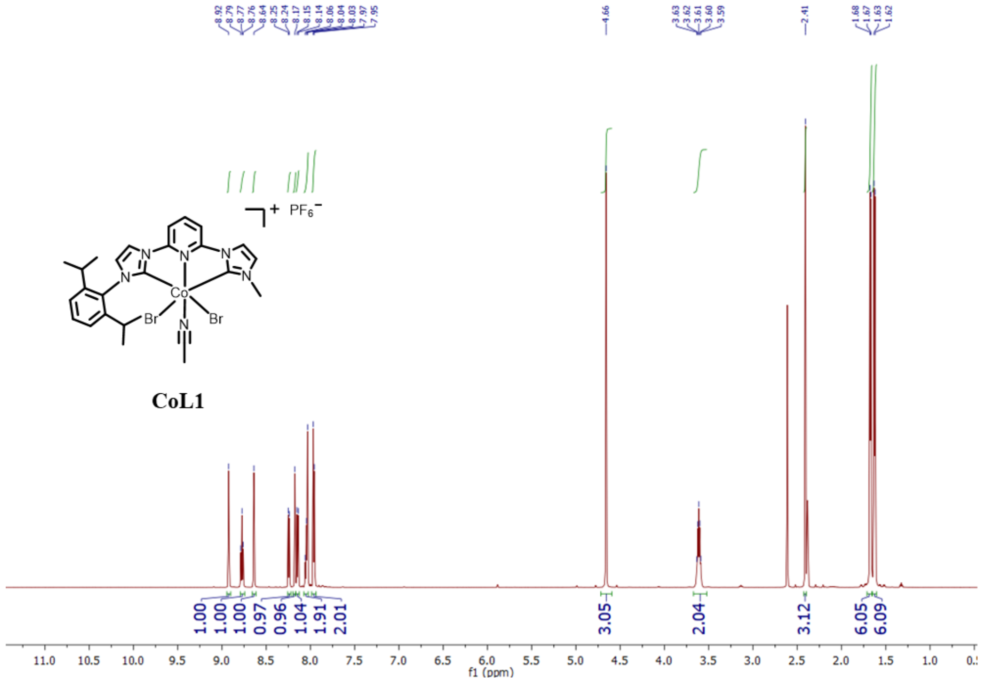

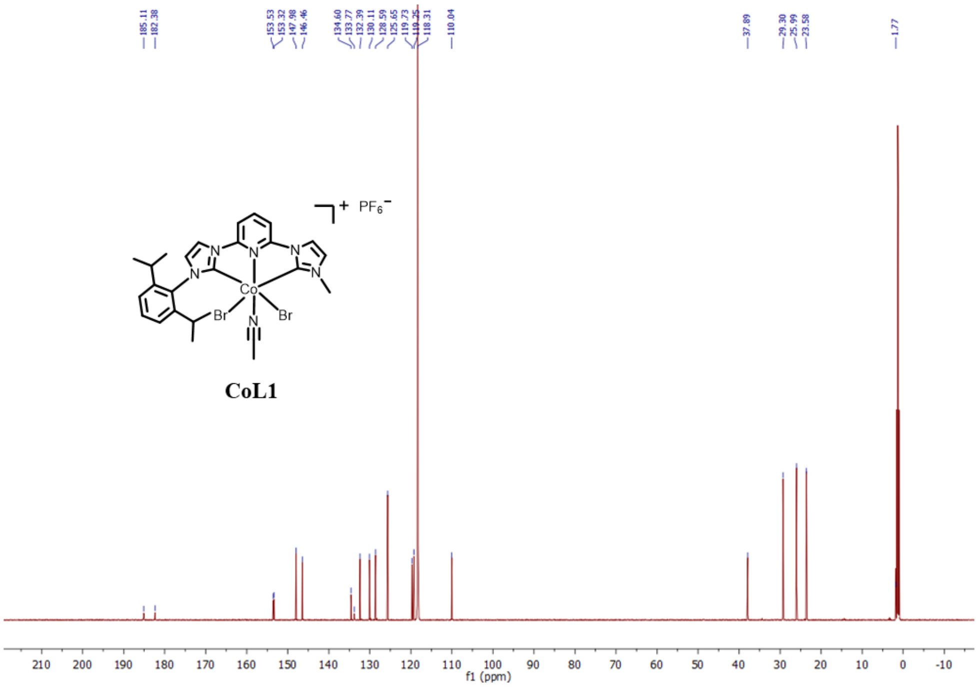

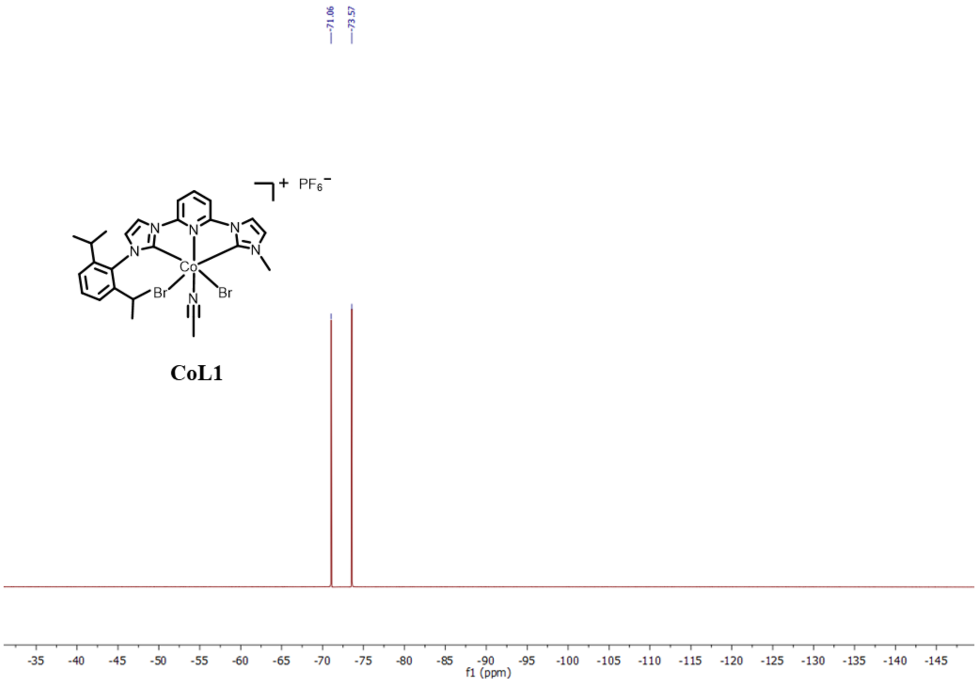

Supplementary Fig. 51. ^{1}H NMR (600 MHz, 298 K), ^{13}C NMR (151 MHz, 298 K) and ^{19}F NMR (282 MHz, 298 K) spectra of **CoL1** in CD_3CN.

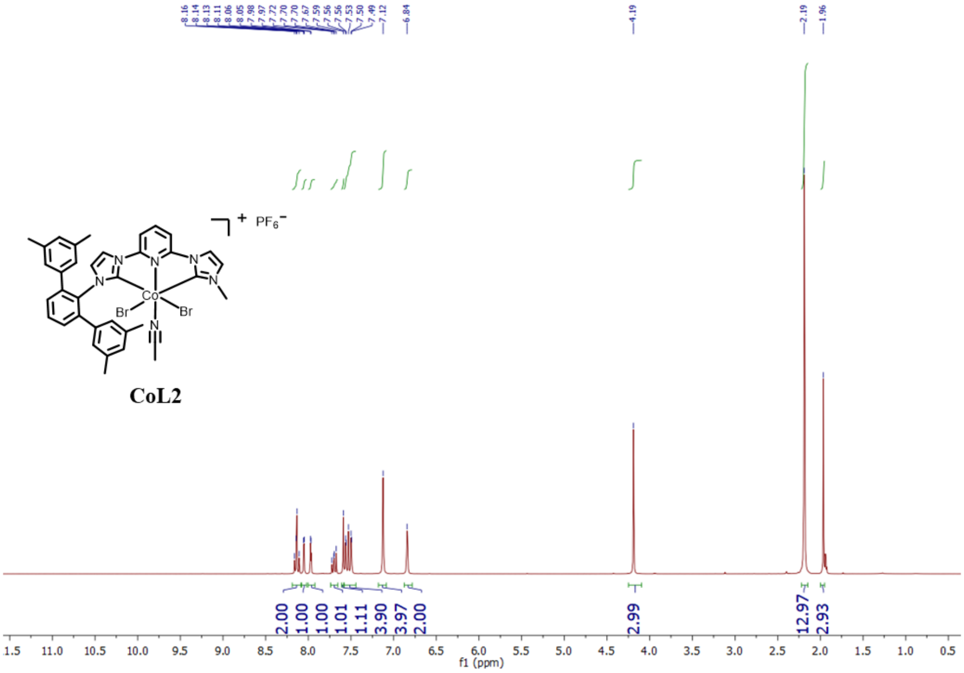

CoL2
f1 (ppm)

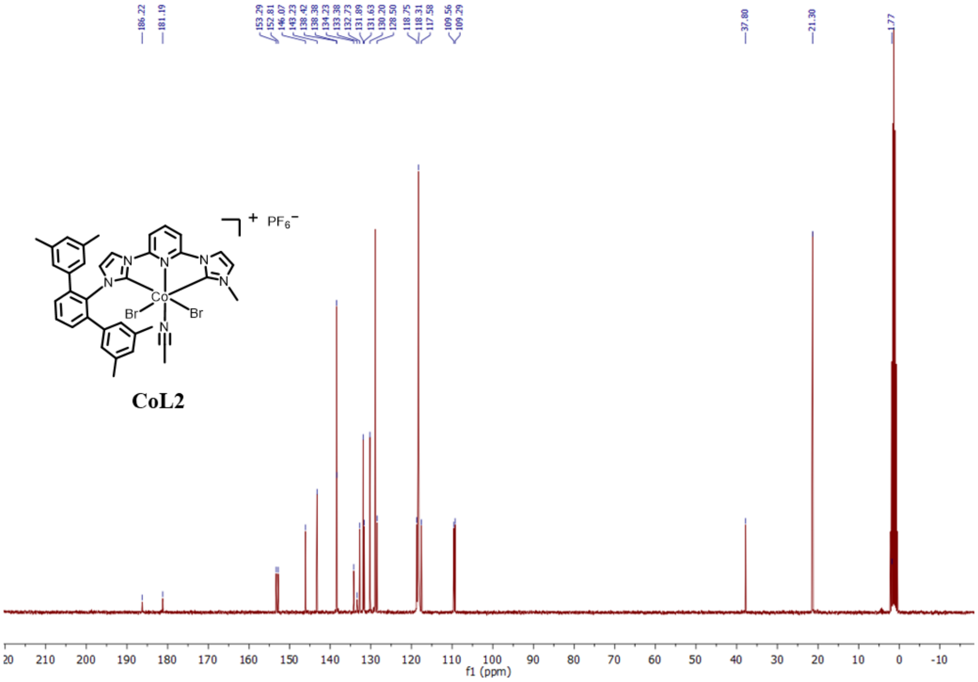

CoL2
f1 (ppm)

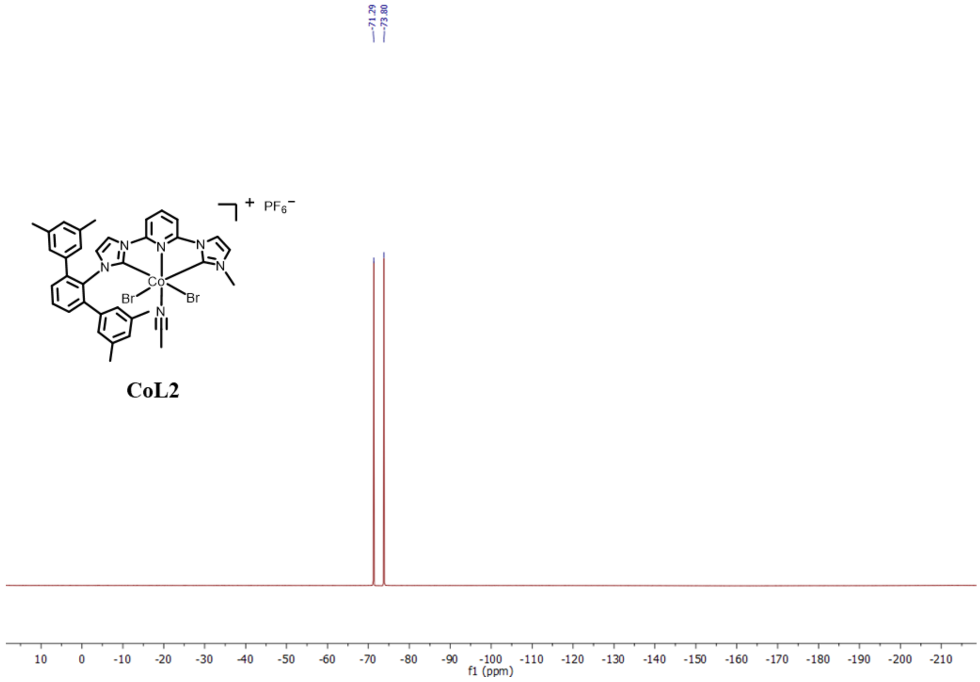

Supplementary Fig. 52. ^{1}H NMR (300 MHz, 298 K), ^{13}C NMR (75 MHz, 298 K) and ^{19}F NMR (282 MHz, 298 K) of **CoL2** in CD_3CN.

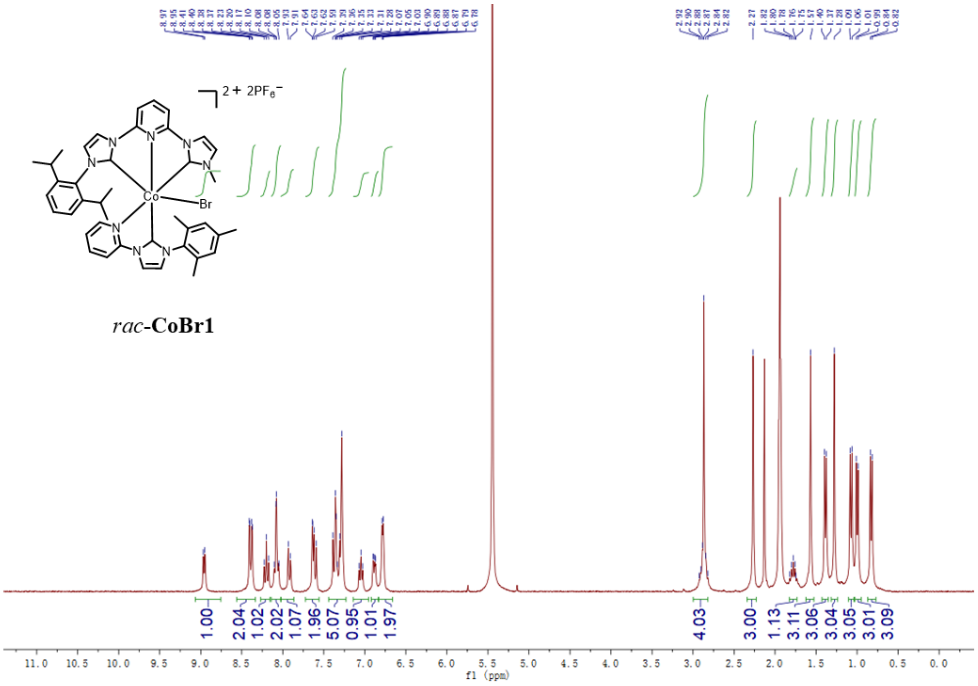

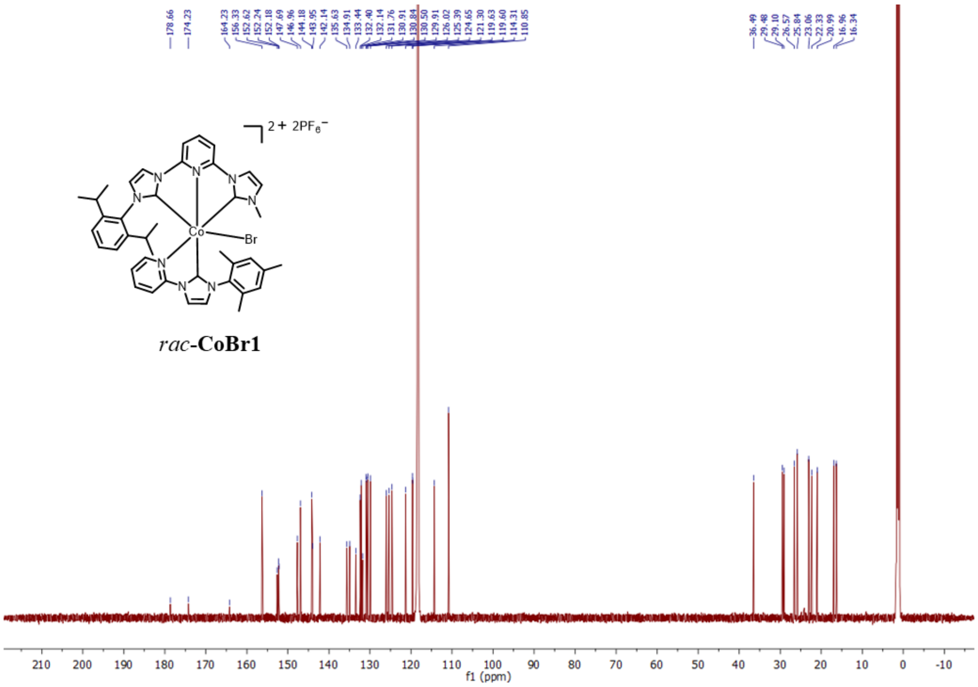

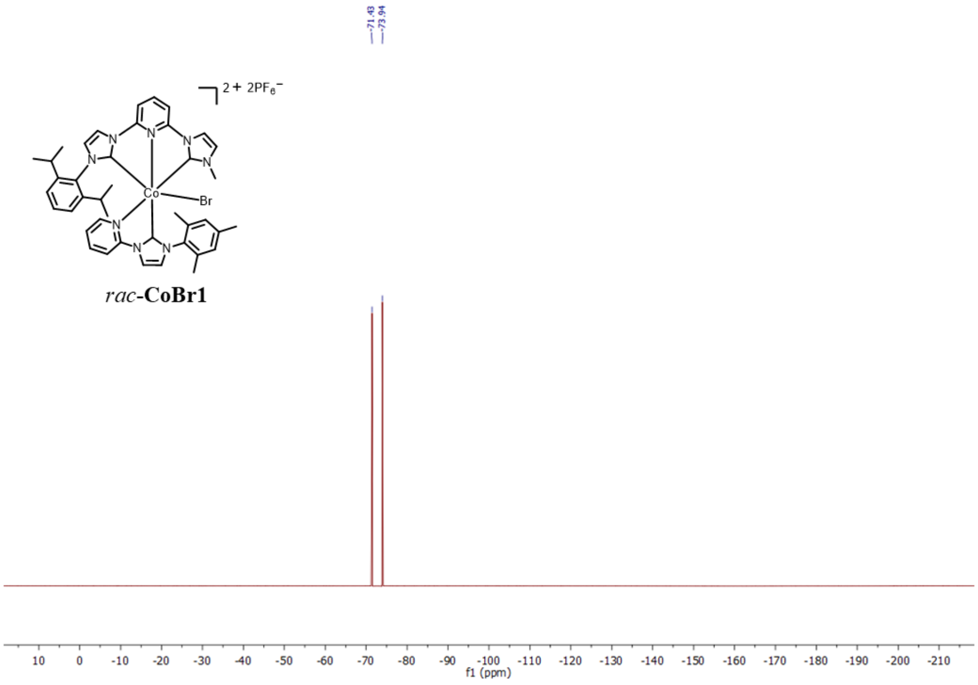

Supplementary Fig. 53 ^{1}H NMR (300 MHz, 298 K), ^{13}C NMR (151 MHz, 298 K) and ^{19}F NMR (282 MHz, 298 K) of *rac*-**CoBr1** in CD_3CN.

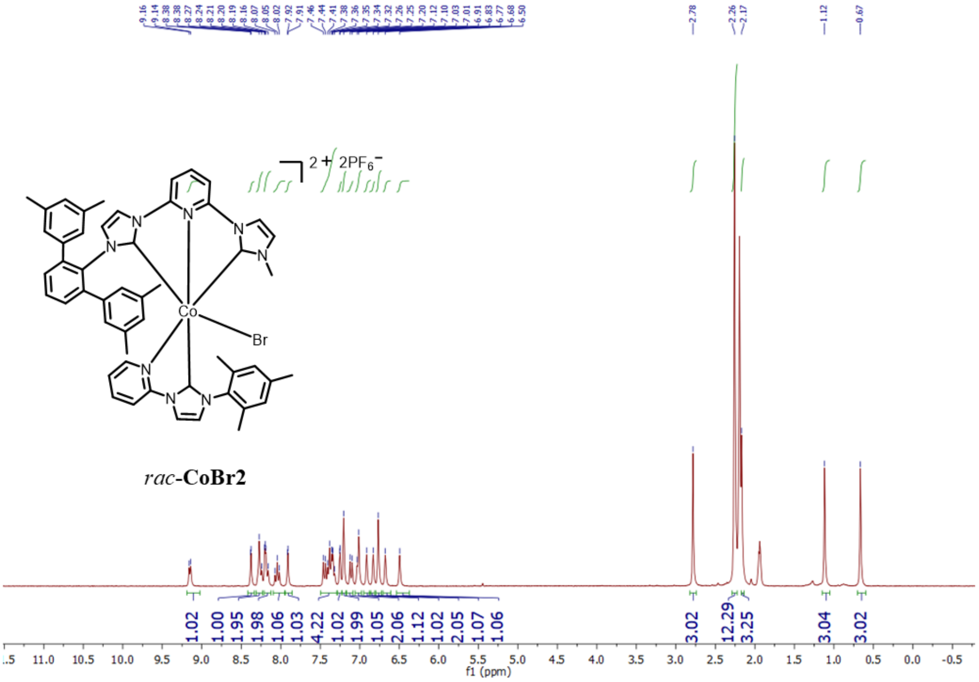
2+ 2PF6−
Co
Br
rac-CoBr2
f1 (ppm)

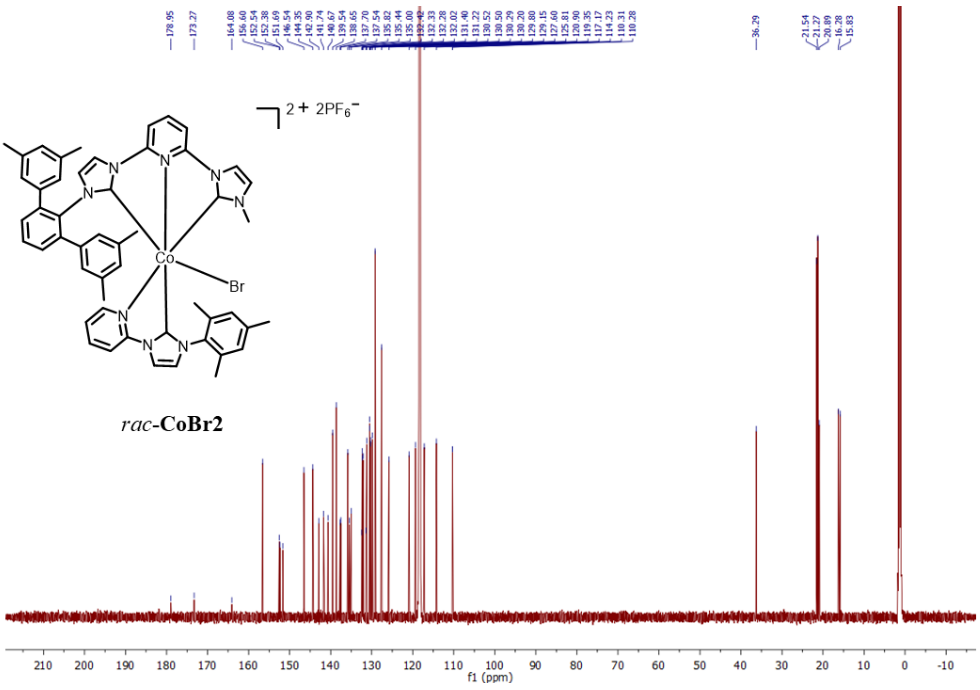
2+ 2PF6−
Co
Br
rac-CoBr2
f1 (ppm)

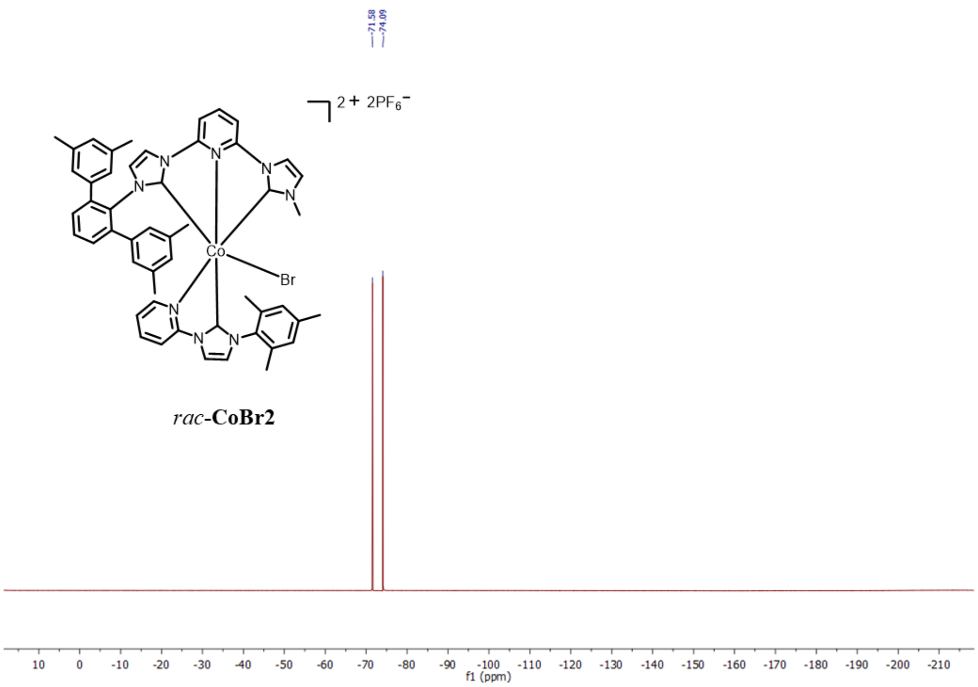

Supplementary Fig. 54. ^{1}H NMR (300 MHz, 298 K), ^{13}C NMR (151 MHz, 298 K) and ^{19}F NMR (282 MHz, 298 K) of *rac*-**CoBr2** in CD_3CN.

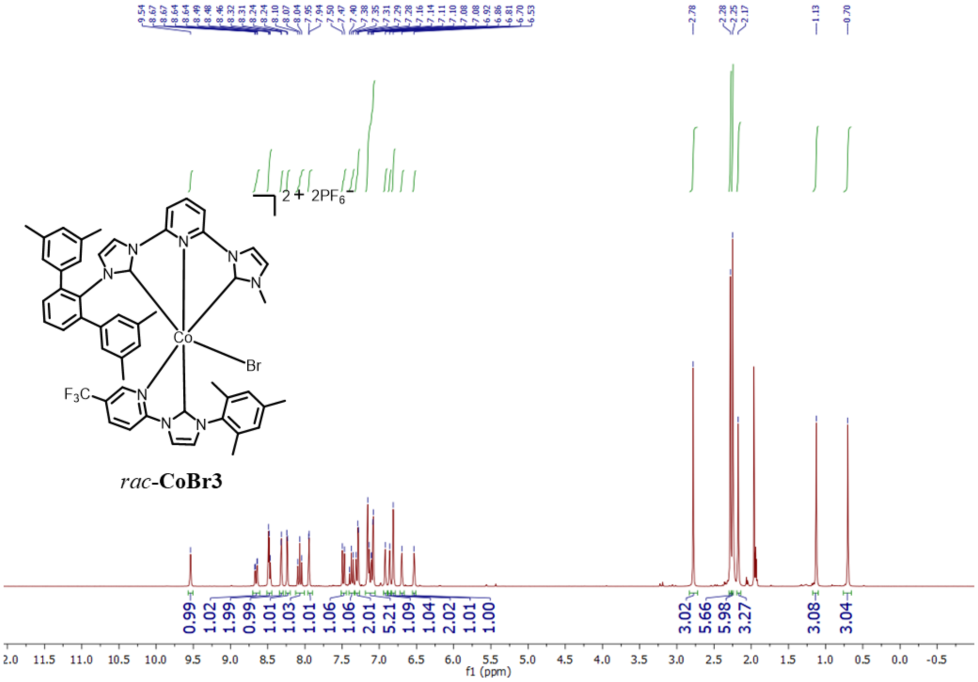

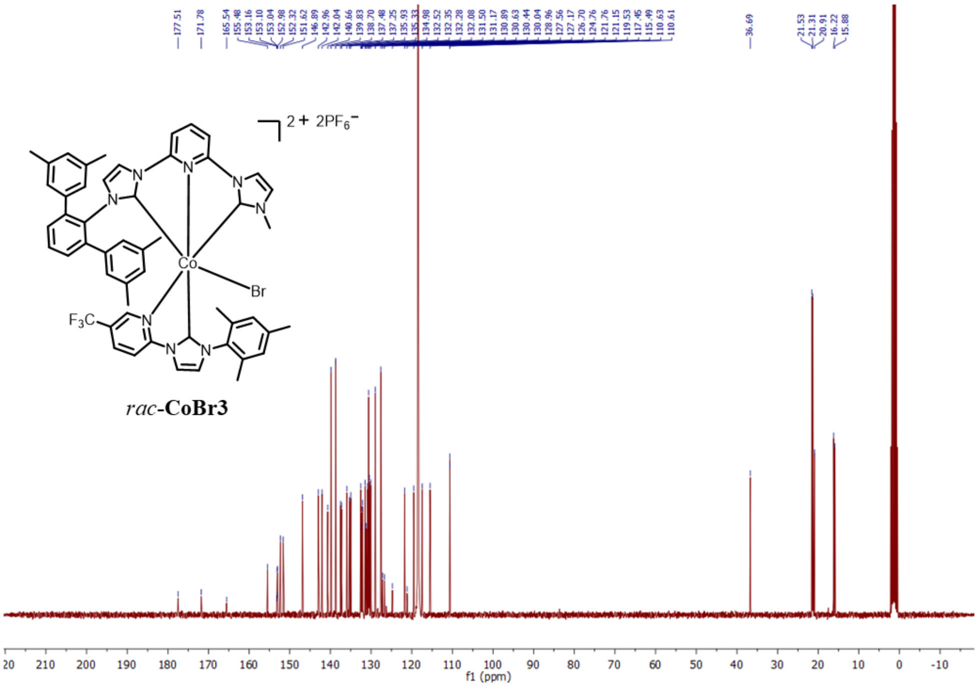

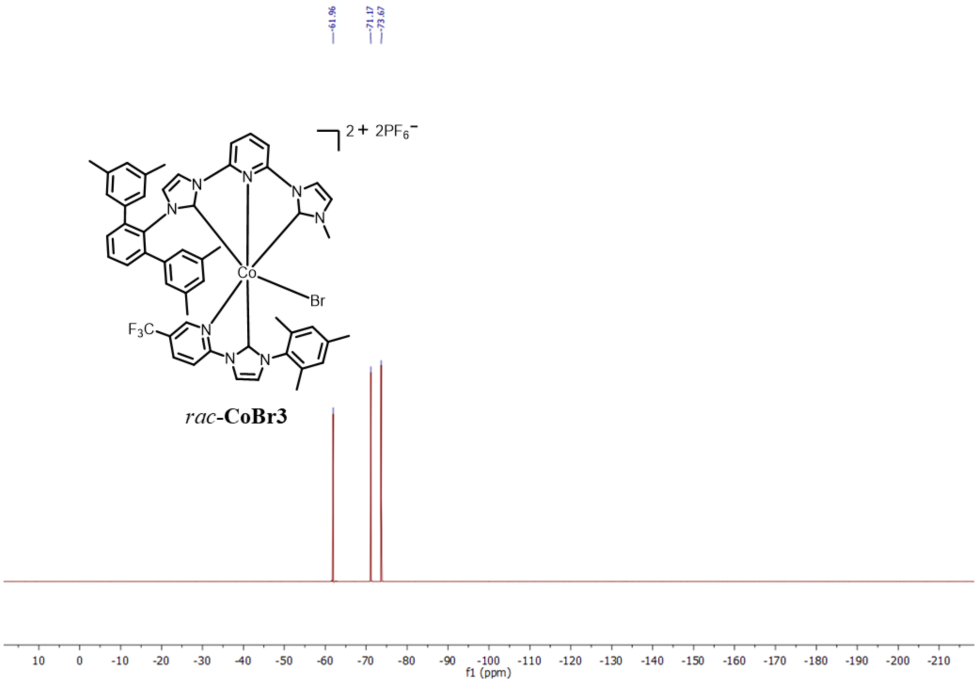

Supplementary Fig. 55. ^{1}H NMR (300 MHz, 298 K), ^{13}C NMR (75 MHz, 298 K) and ^{19}F NMR (282 MHz, 298 K) of *rac*-**CoBr3** in CD_3CN.

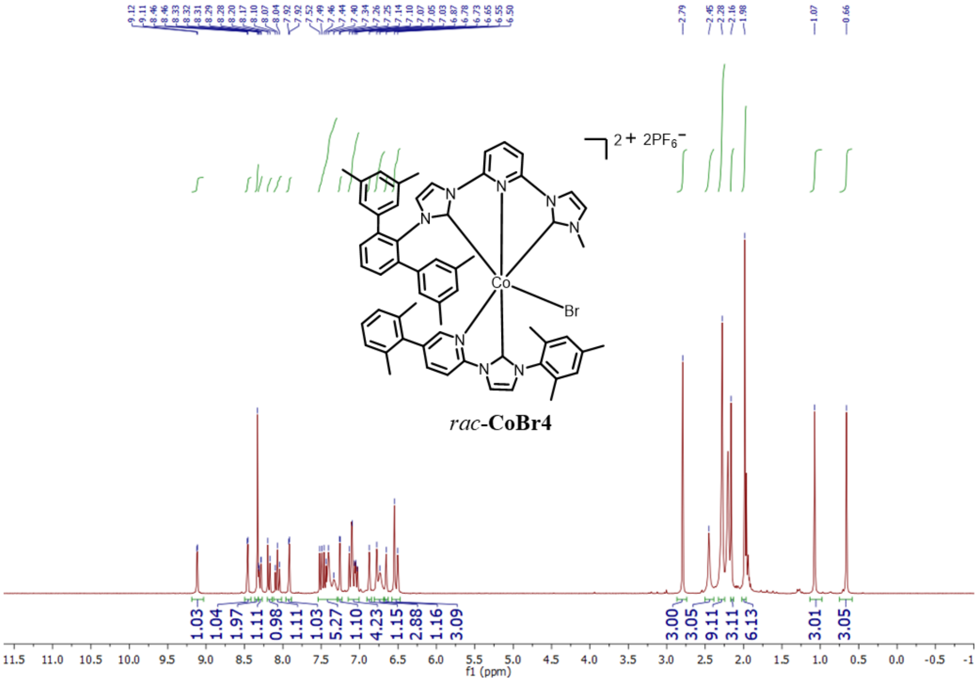
2+ 2PF6−
rac-CoBr4

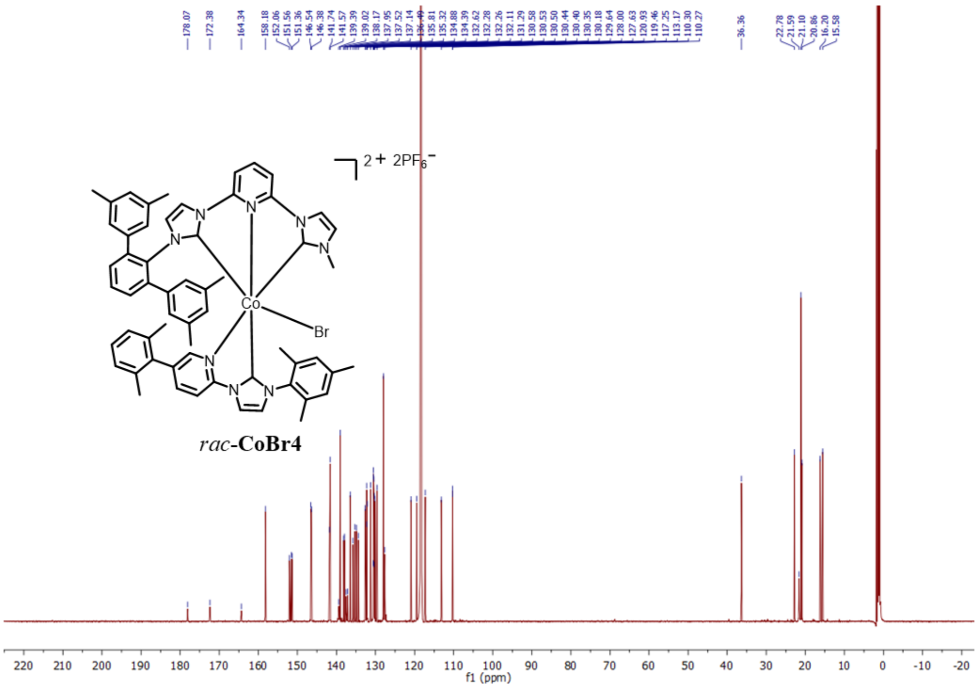
2+ 2PF6−
rac-CoBr4

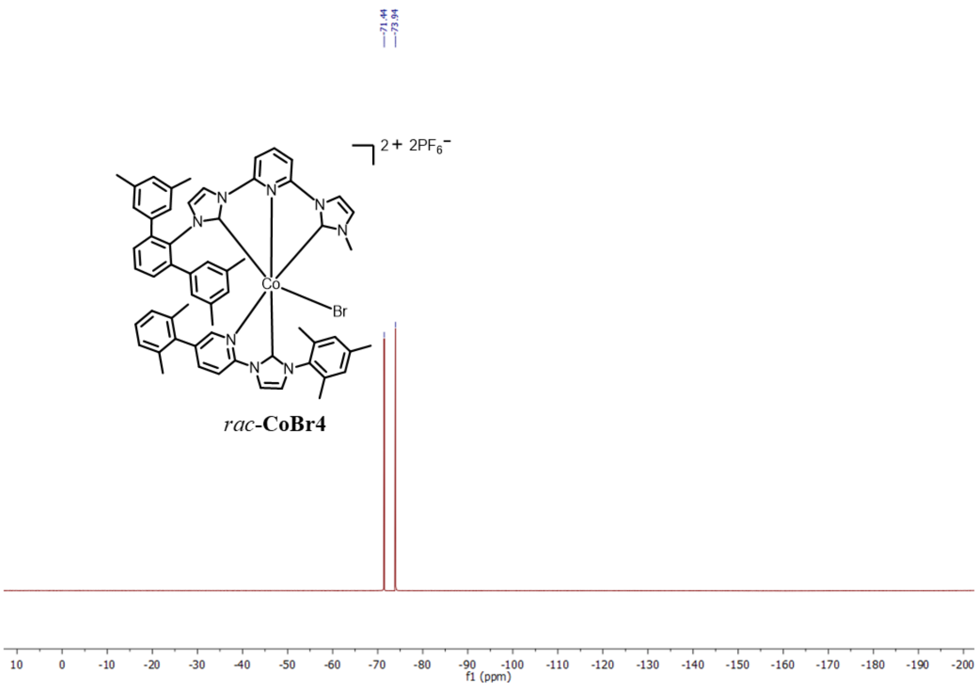

Supplementary Fig. 56. ^{1}H NMR (300 MHz, 298 K), ^{13}C NMR (126 MHz, 298 K) and ^{19}F NMR (282 MHz, 298 K) spectra of *rac*-**CoBr4** in CD_3CN.

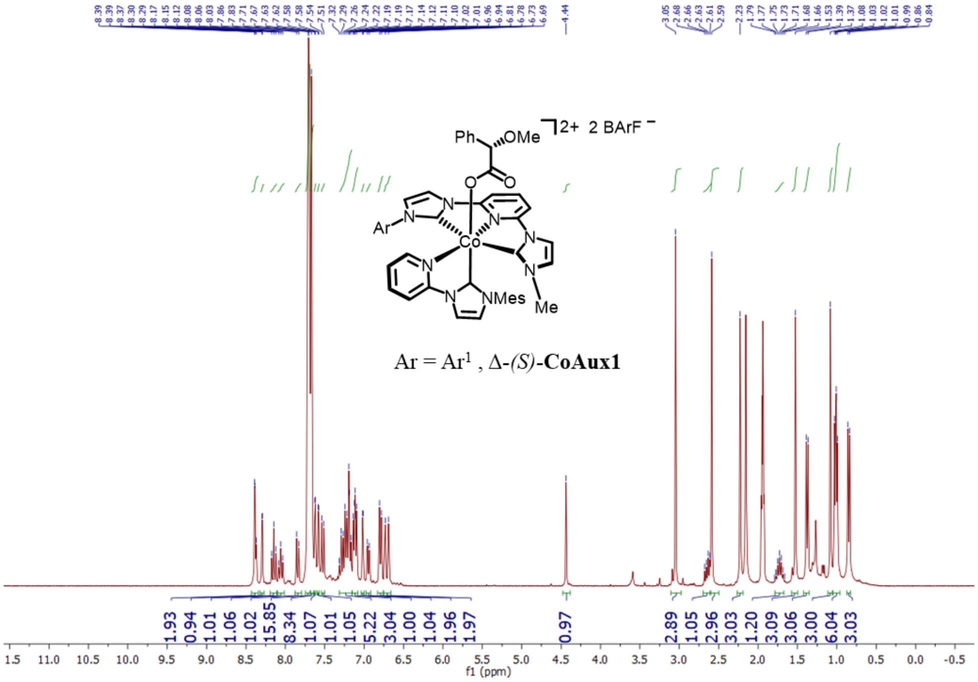

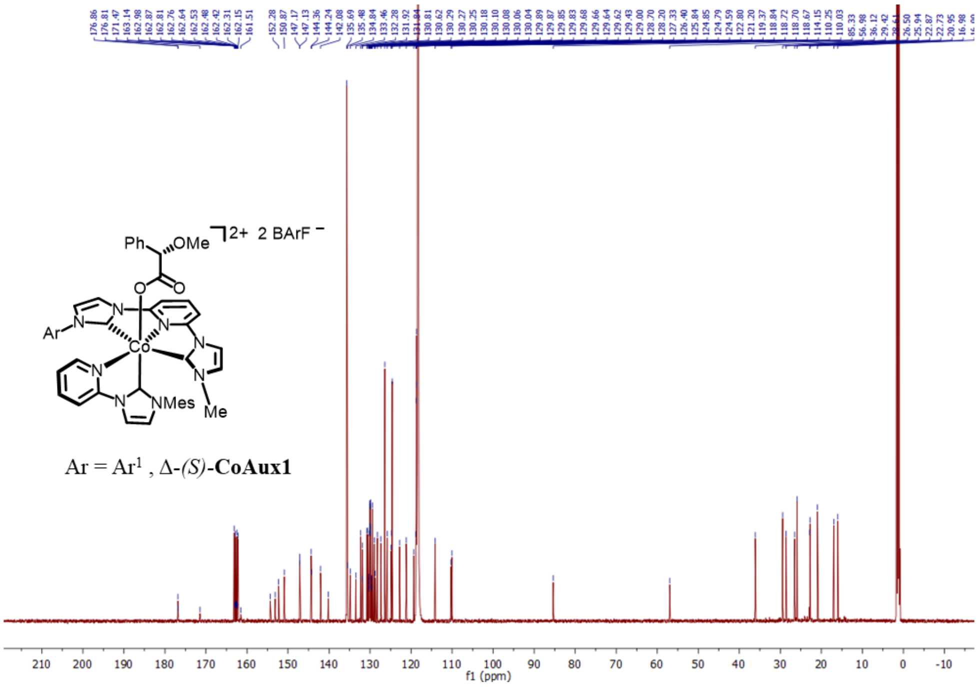

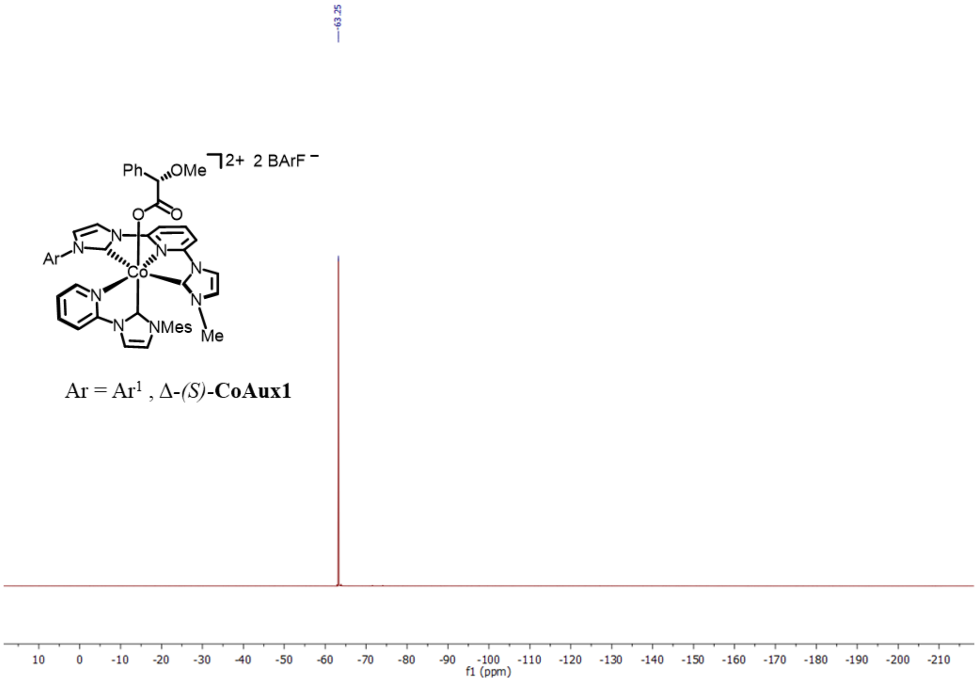

Supplementary Fig. 57. ^{1}H NMR (300 MHz, 298 K), ^{13}C NMR (151 MHz, 298 K) and ^{19}F NMR (282 MHz, 298 K) of Δ-*(S)*-**CoAux1** in CD_3CN.

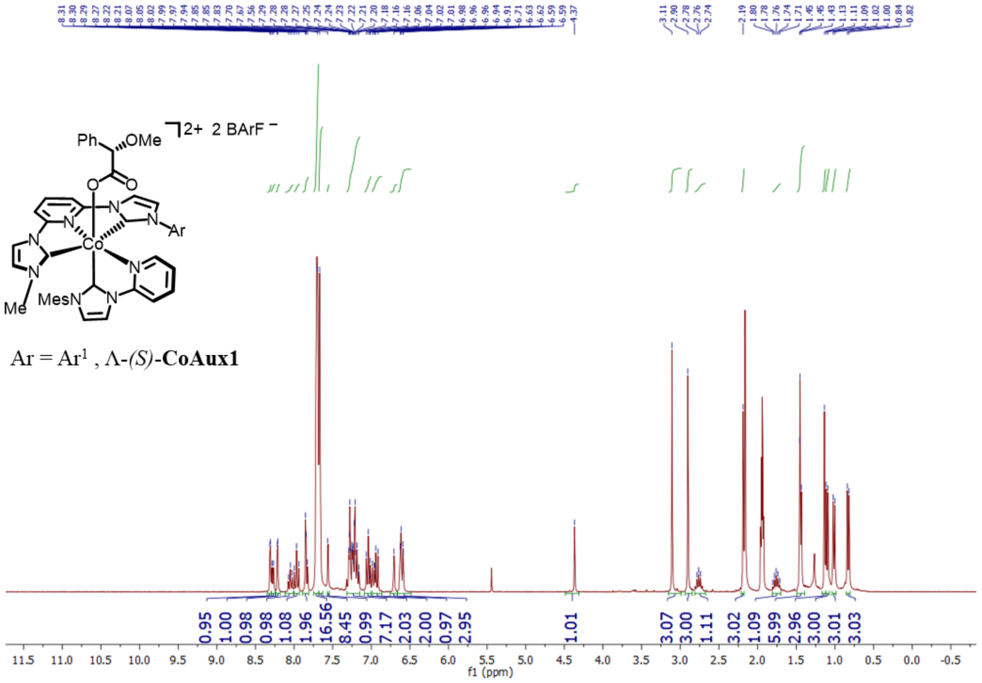
2+ 2 BArF⁻
Ar = Ar1 , Λ-(S)-CoAux1
f1 (ppm)

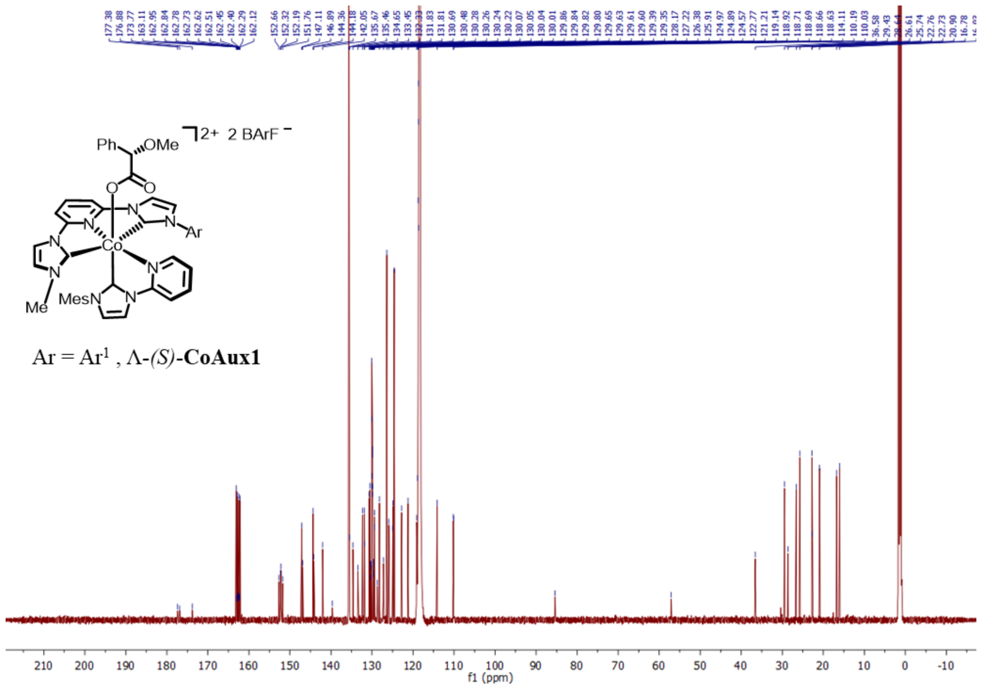
2+ 2 BArF⁻
Ar = Ar1 , Λ-(S)-CoAux1
f1 (ppm)

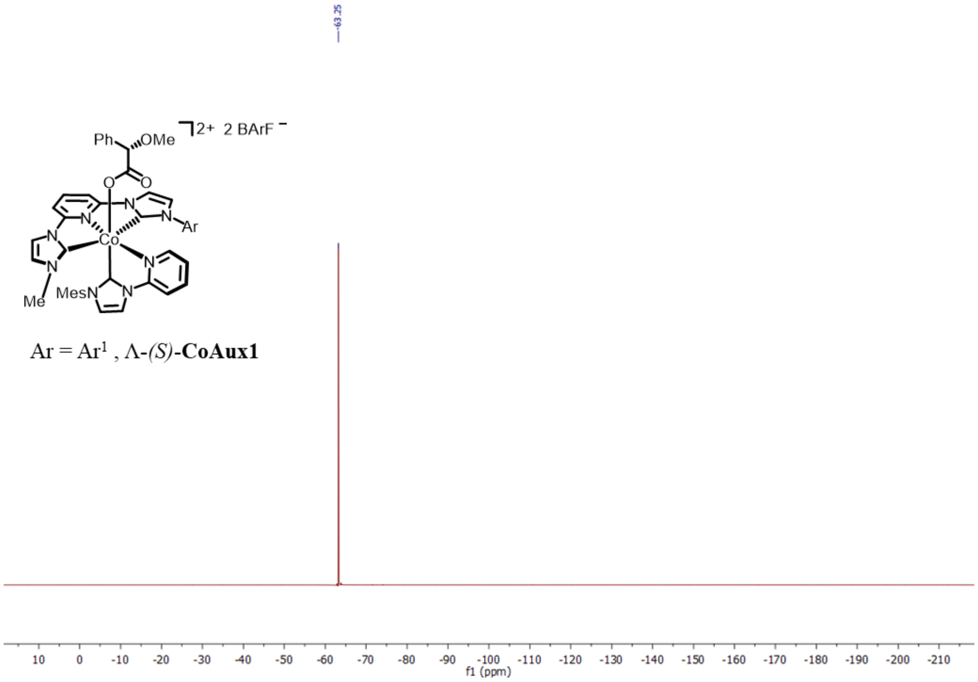

Supplementary Fig. 58. ^{1}H NMR (300 MHz, 298 K), ^{13}C NMR (75 MHz, 298 K) and ^{19}F NMR (282 MHz, 298 K) of Λ-*(S)*-**CoAux1** in CD_3CN.

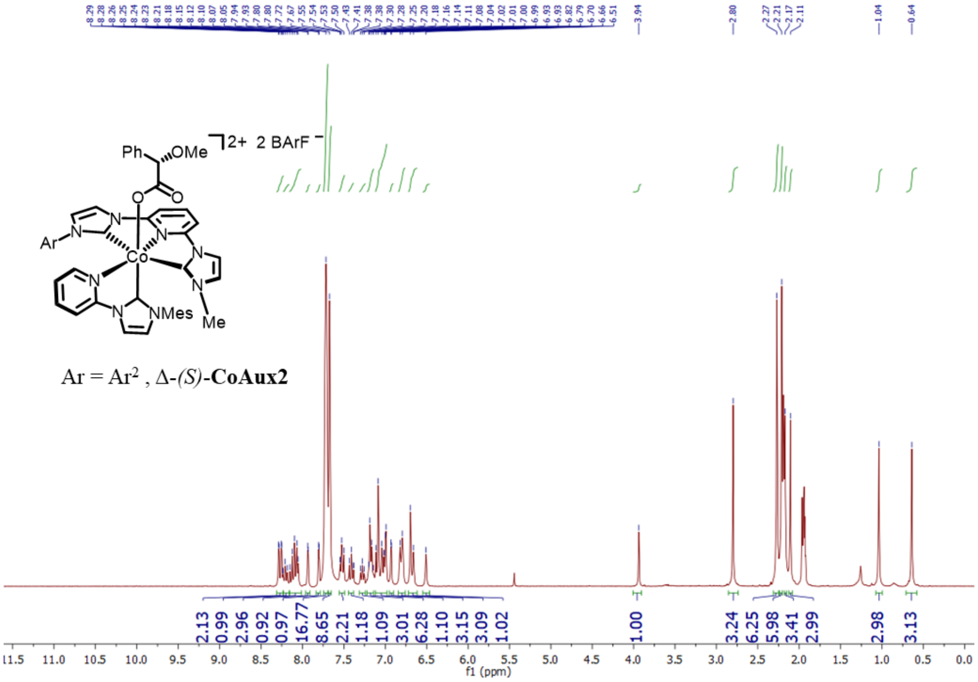

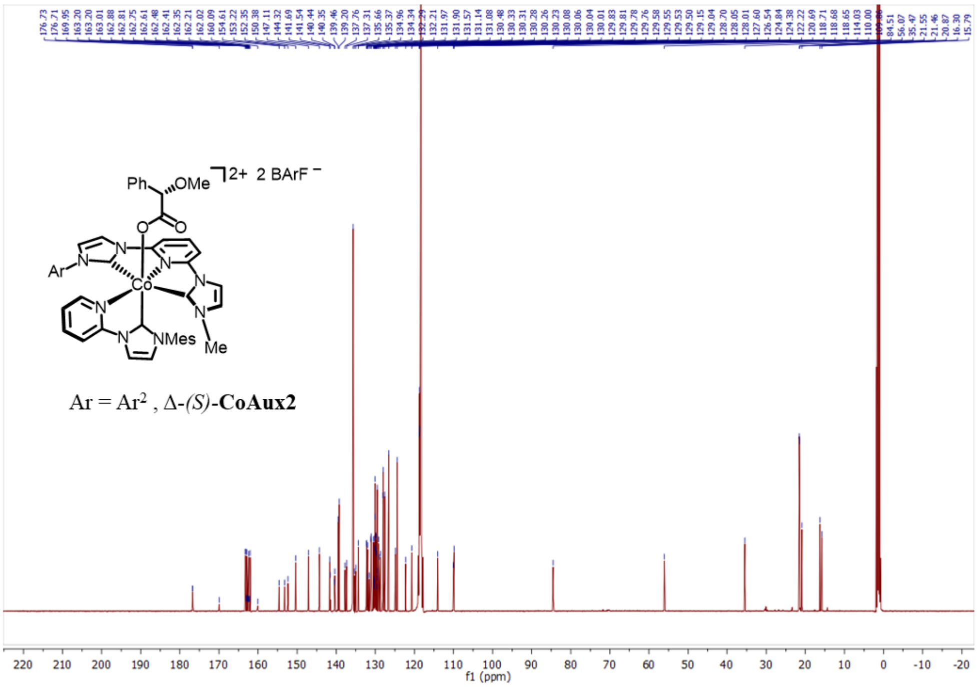

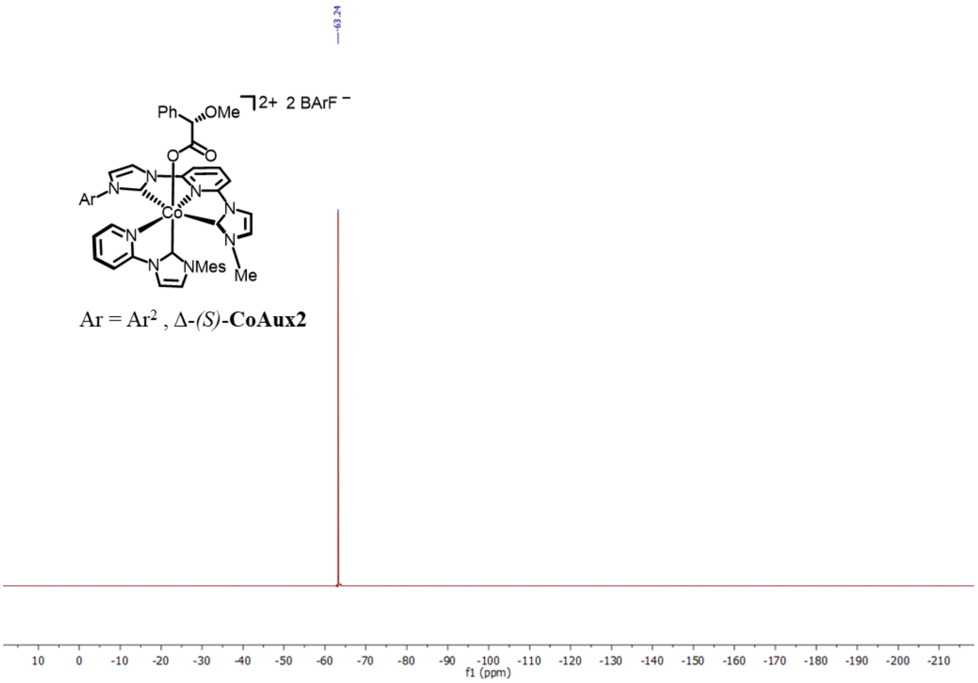

Supplementary Fig. 59. ^{1}H NMR (300 MHz, 298 K), ^{13}C NMR (126 MHz, 298 K) and ^{19}F NMR (282 MHz, 298 K) spectra of Δ-*(S)*-**CoAux2** in CD_3CN.

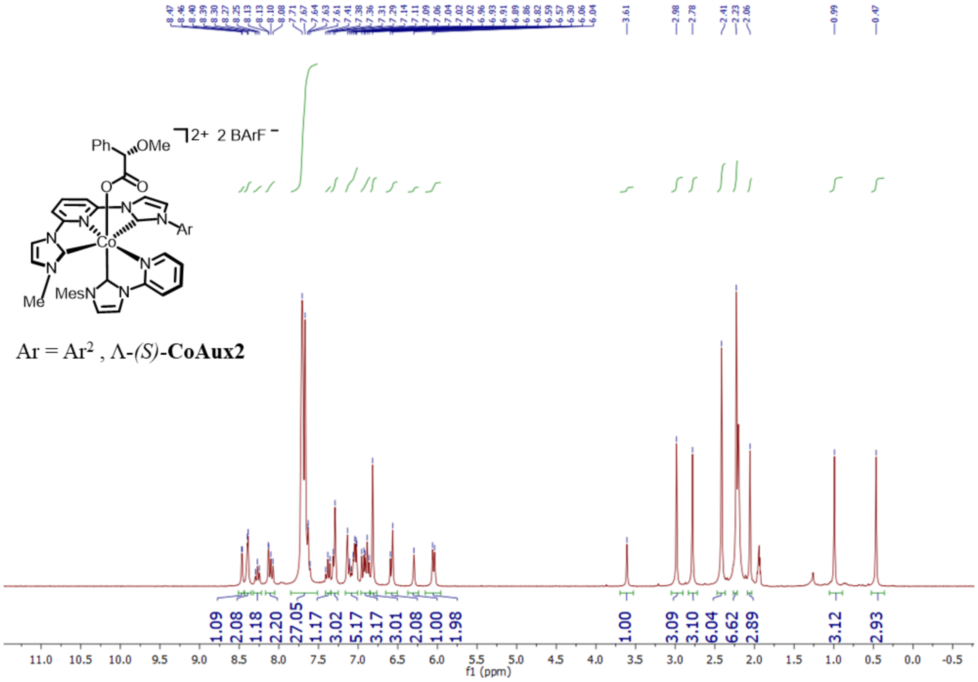
Ph
OMe
2+ 2 BArF −
Co
Ar
Me
MesN
Ar = Ar2 , Λ-(S)-CoAux2
f1 (ppm)

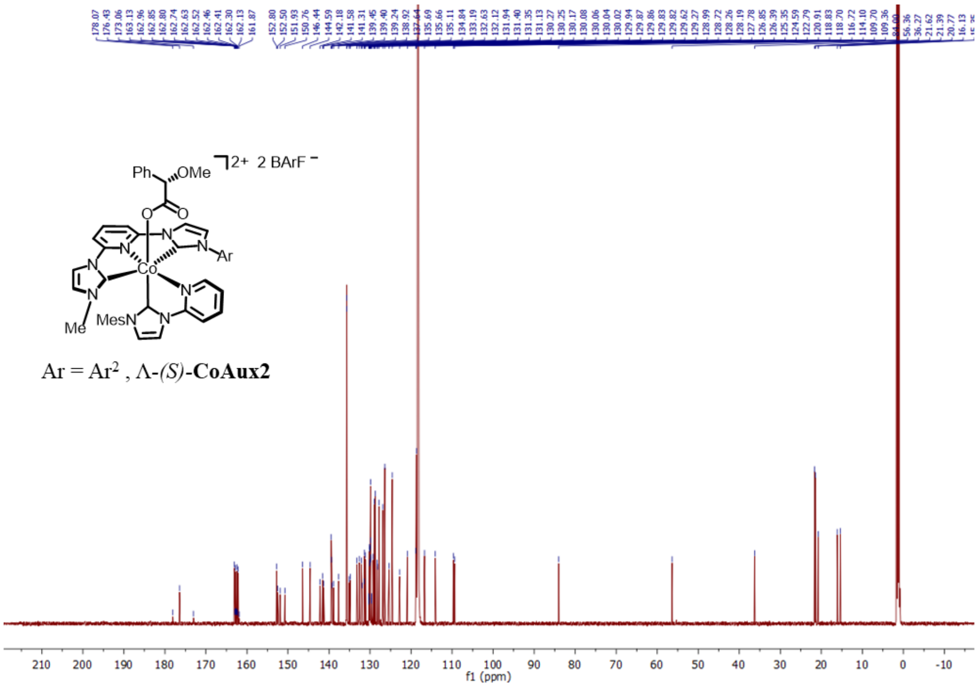
Ph
OMe
2+ 2 BArF −
Co
Ar
Me
MesN
Ar = Ar2 , Λ-(S)-CoAux2
f1 (ppm)

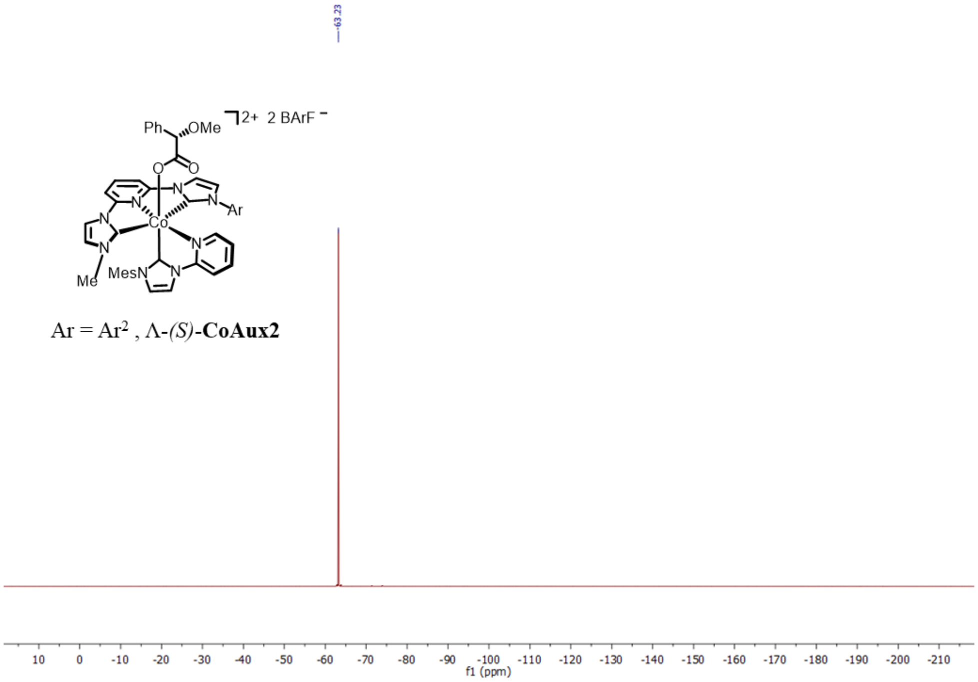

Supplementary Fig. 60. ^{1}H NMR (300 MHz, 298 K), ^{13}C NMR (151 MHz, 298 K) and ^{19}F NMR (282 MHz, 298 K) of Λ-*(S)*-**CoAux2** in CD_3CN.

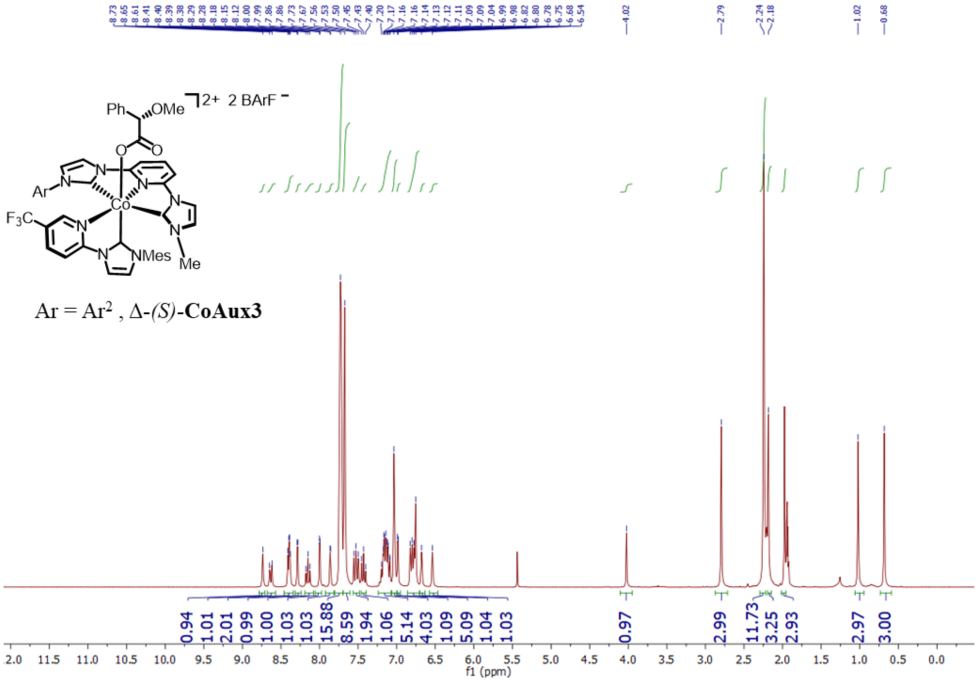

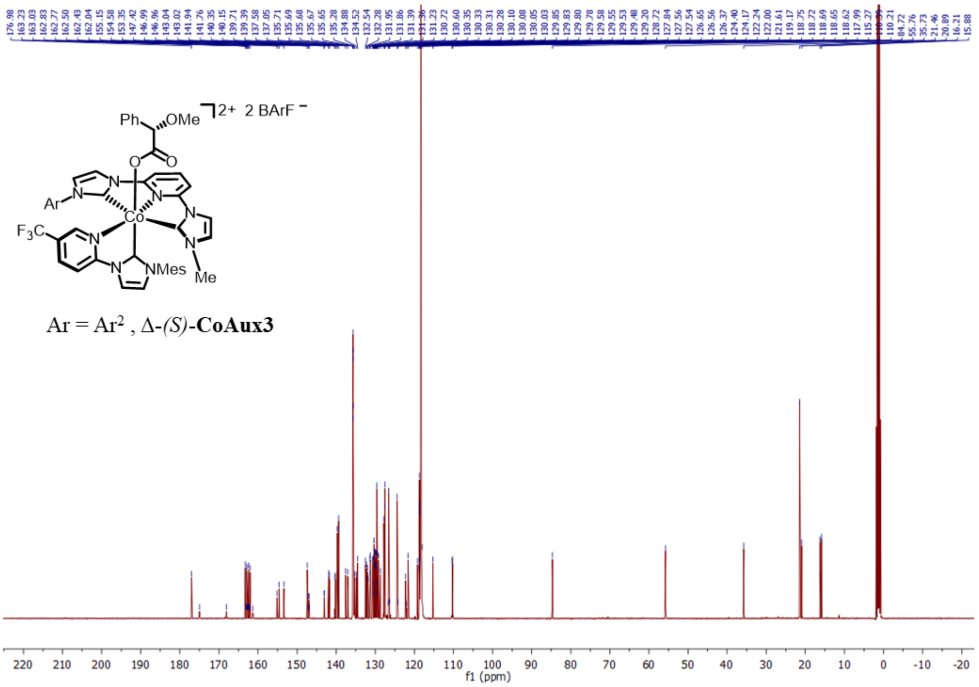

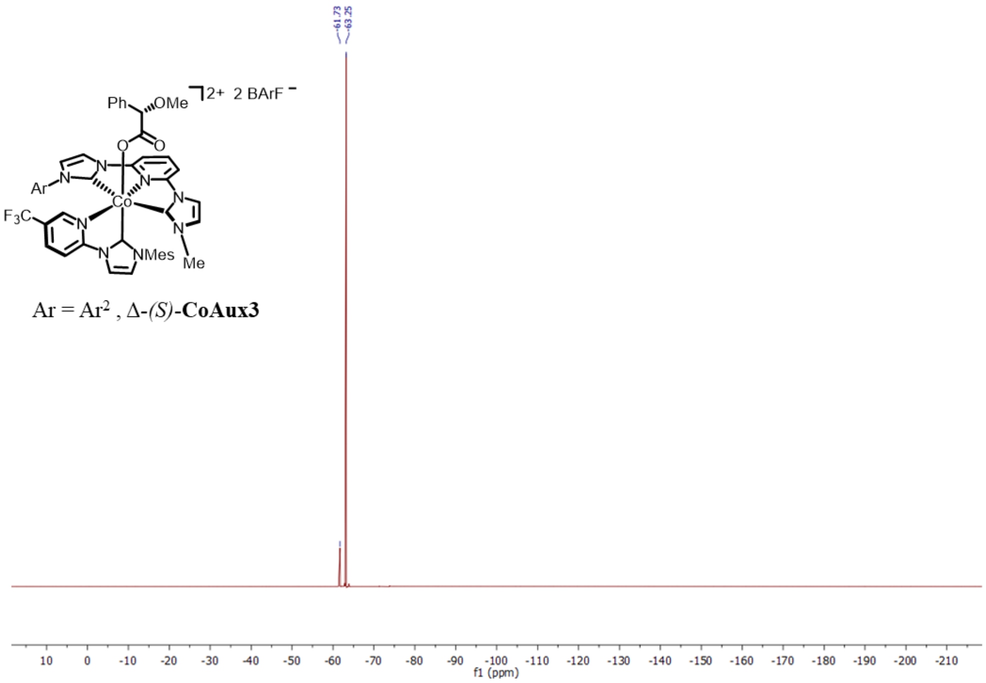

Supplementary Fig. 61. ^{1}H NMR (300 MHz, 298 K), ^{13}C NMR (126 MHz, 298 K) and ^{19}F NMR (282 MHz, 298 K) of Δ-*(S)*-**CoAux3** in CD_3CN.

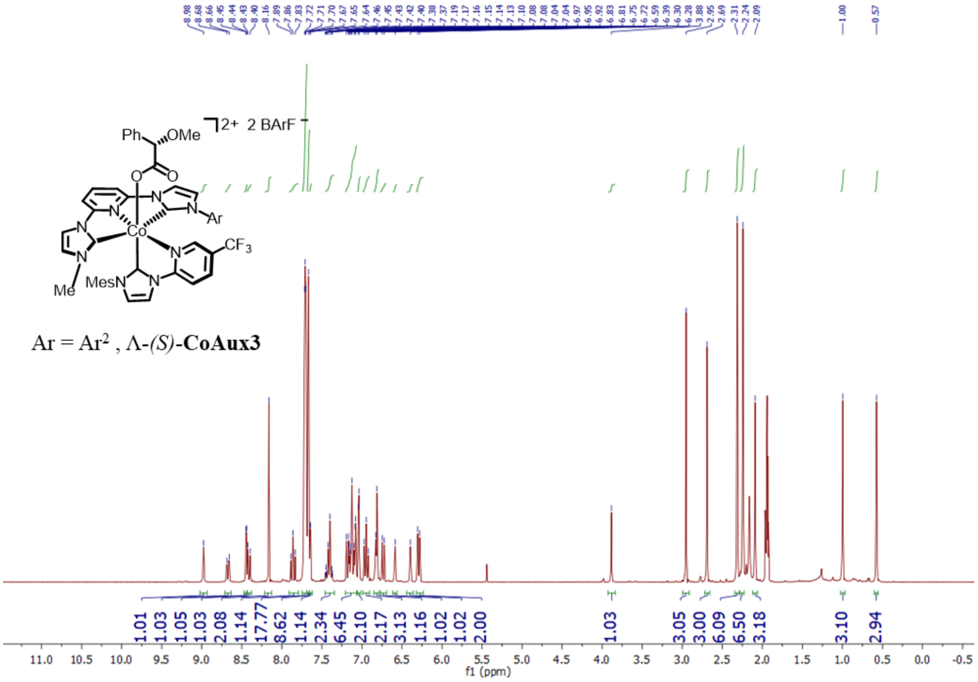
Ph
OMe
2+ 2 BArF −
Co
CF3
Mes
Me
Ar
Ar = Ar², Λ-(S)-CoAux3
f1 (ppm)

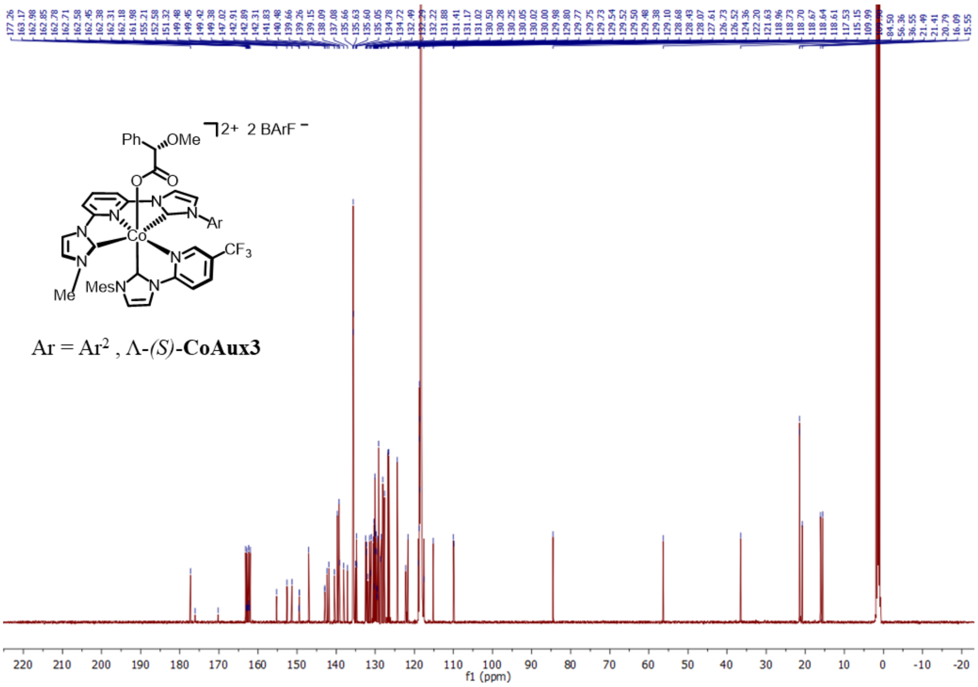
Ph
OMe
2+ 2 BArF −
Co
CF3
Mes
Me
Ar
Ar = Ar², Λ-(S)-CoAux3
f1 (ppm)

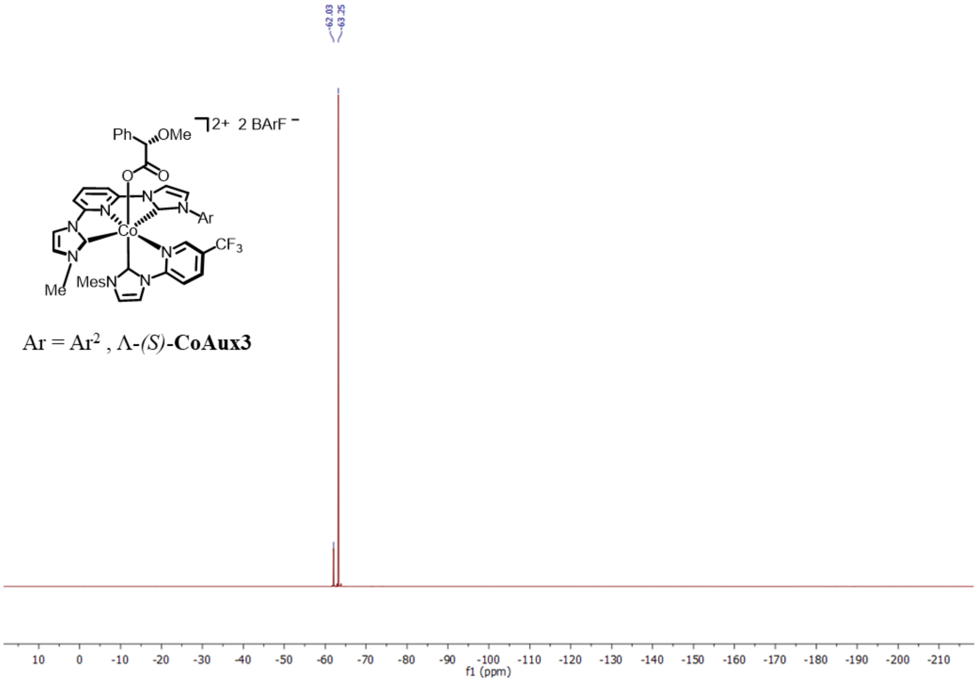

Supplementary Fig. 62. ^{1}H NMR (300 MHz, 298 K), ^{13}C NMR (126 MHz, 298 K) and ^{19}F NMR (282 MHz, 298 K) spectra of Λ-*(S)*-**CoAux3** in CD_3CN.

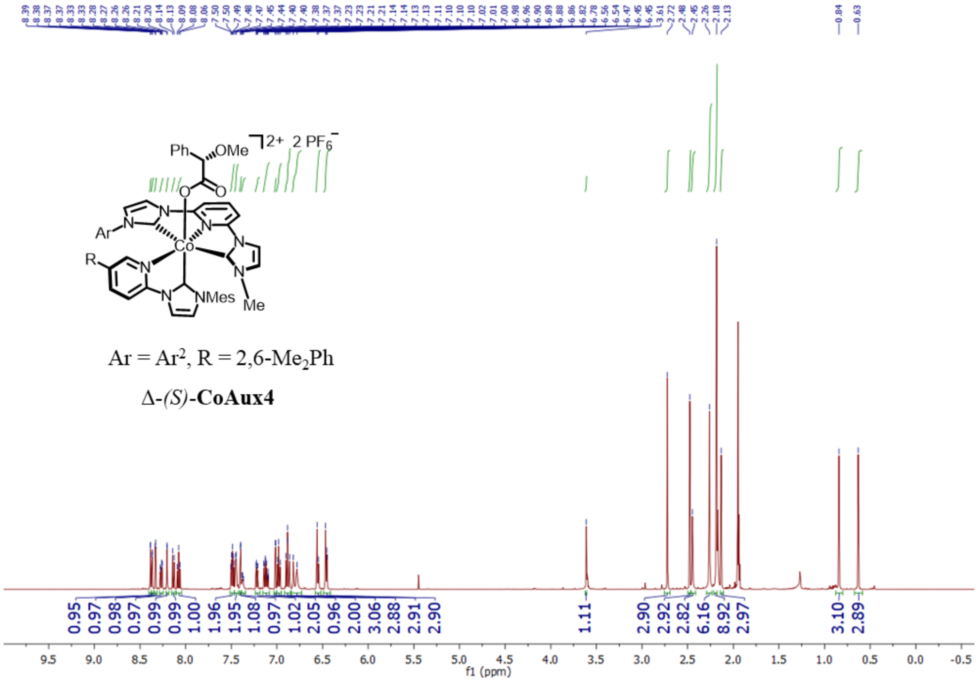

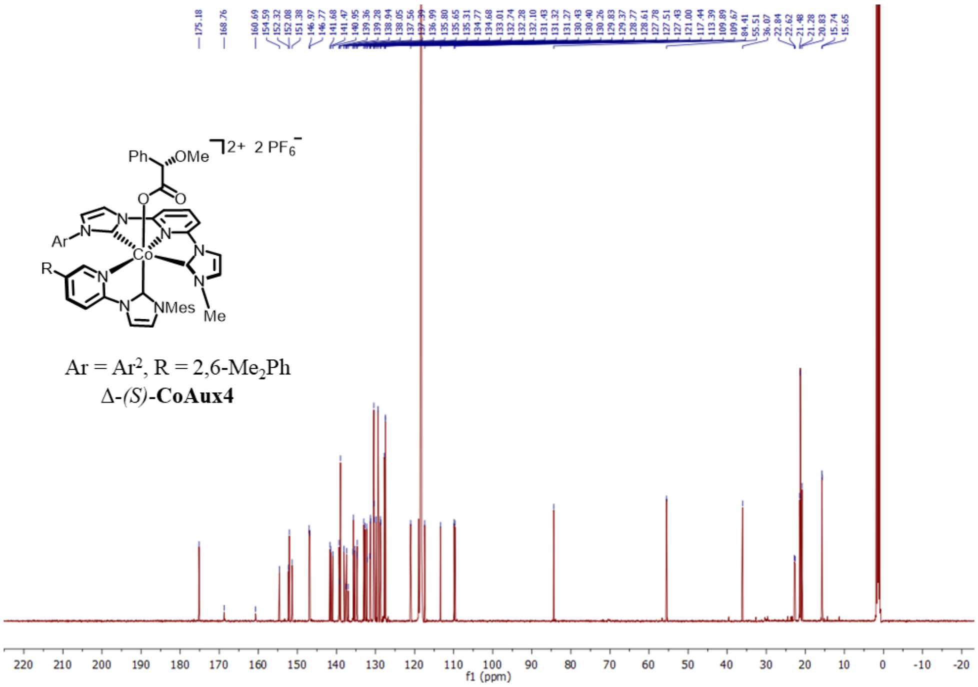

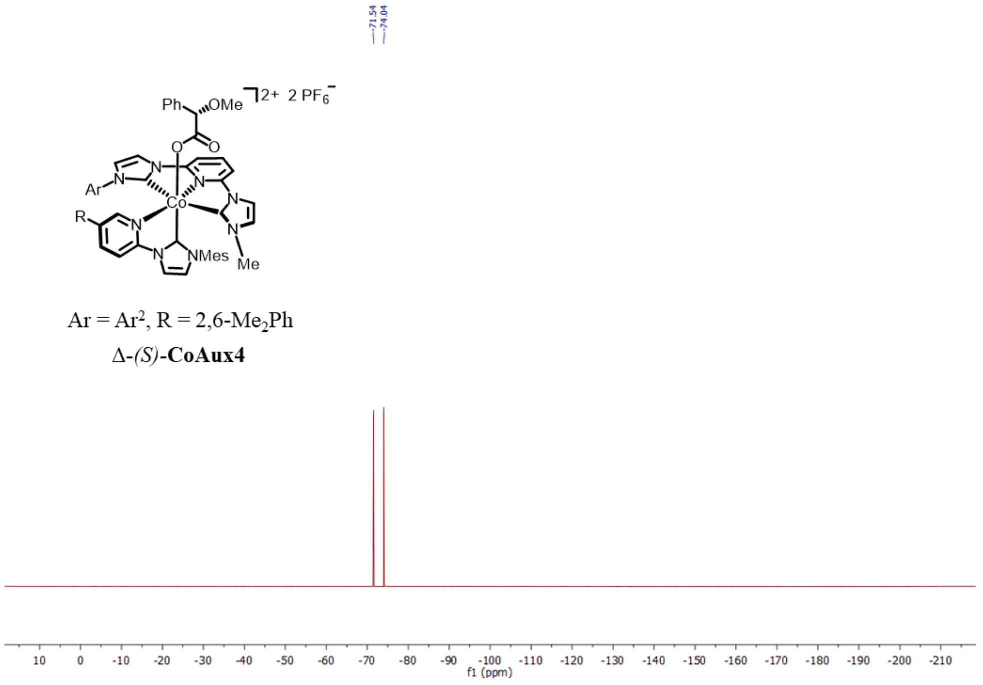

Supplementary Fig. 63. ^{1}H NMR (500 MHz, 298 K), ^{13}C NMR (126 MHz, 298 K) and ^{19}F NMR (282 MHz, 298 K) spectra of Δ-*(S)*-**CoAux4** in CD_3CN.

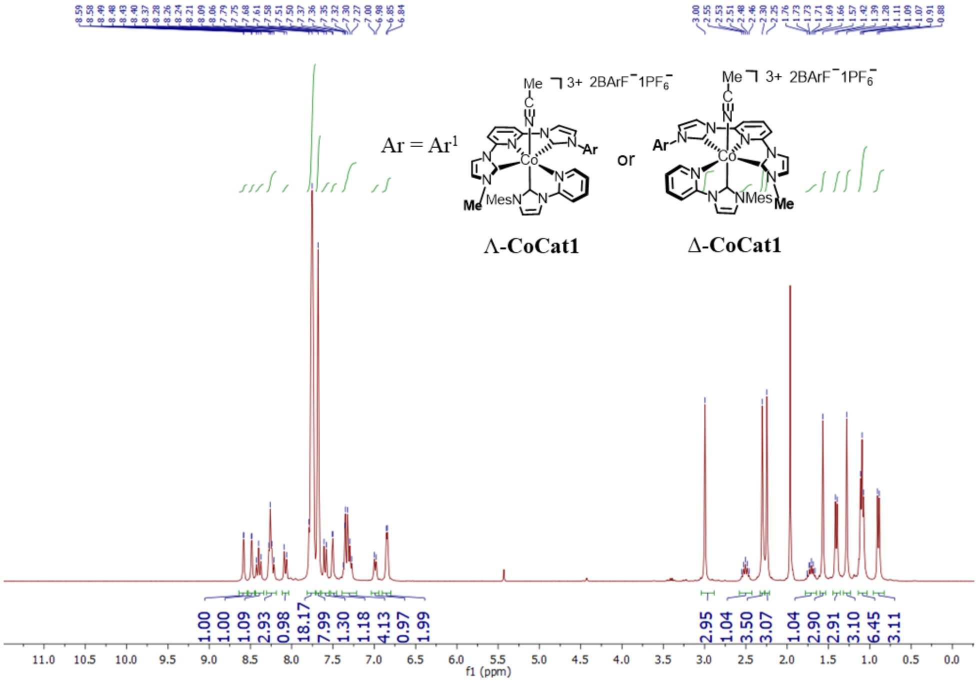
Ar = Ar1
Λ-CoCat1
or
Δ-CoCat1
f1 (ppm)

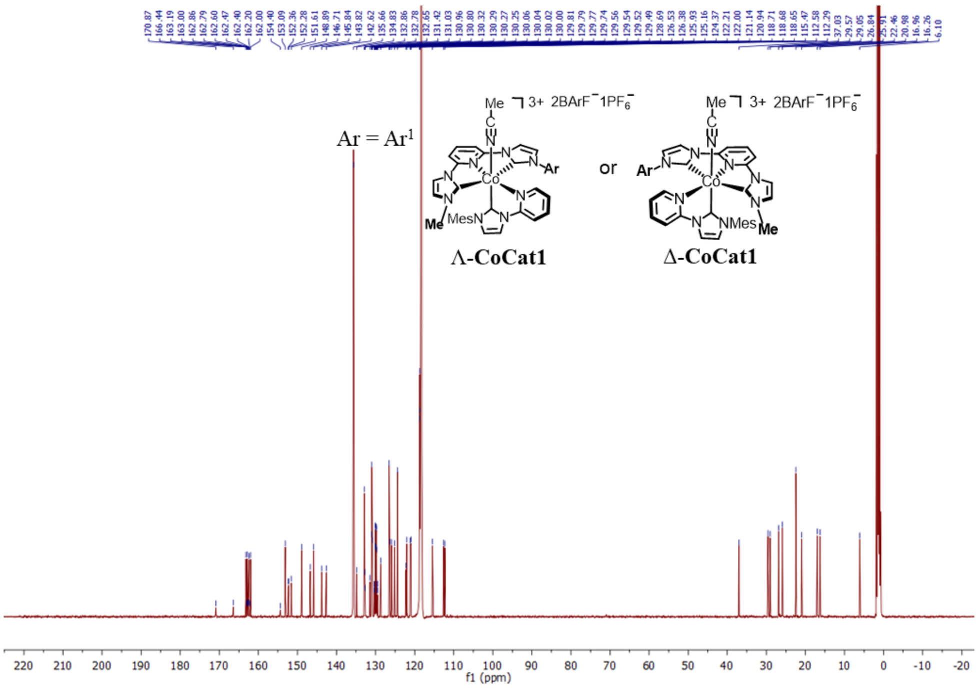
Ar = Ar1
Λ-CoCat1
or
Δ-CoCat1
f1 (ppm)

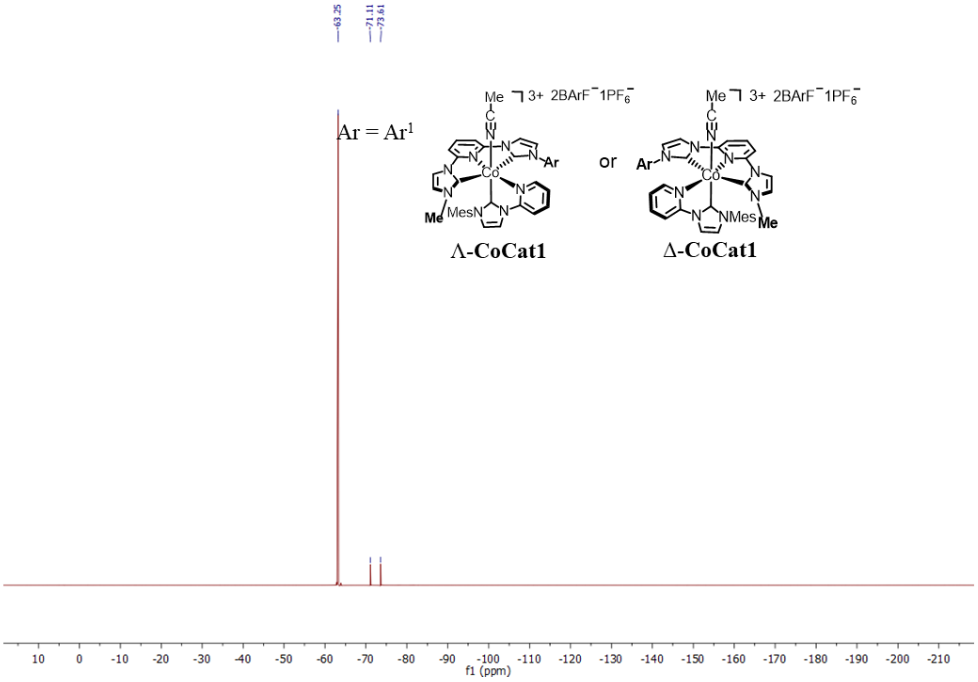

Supplementary Fig. 64. ^{1}H NMR (300 MHz, 298 K), ^{13}C NMR (126 MHz, 298 K) and ^{19}F NMR (282 MHz, 298 K) spectra of Λ-**CoCat1** or Δ-**CoCat1** in CD_3CN.

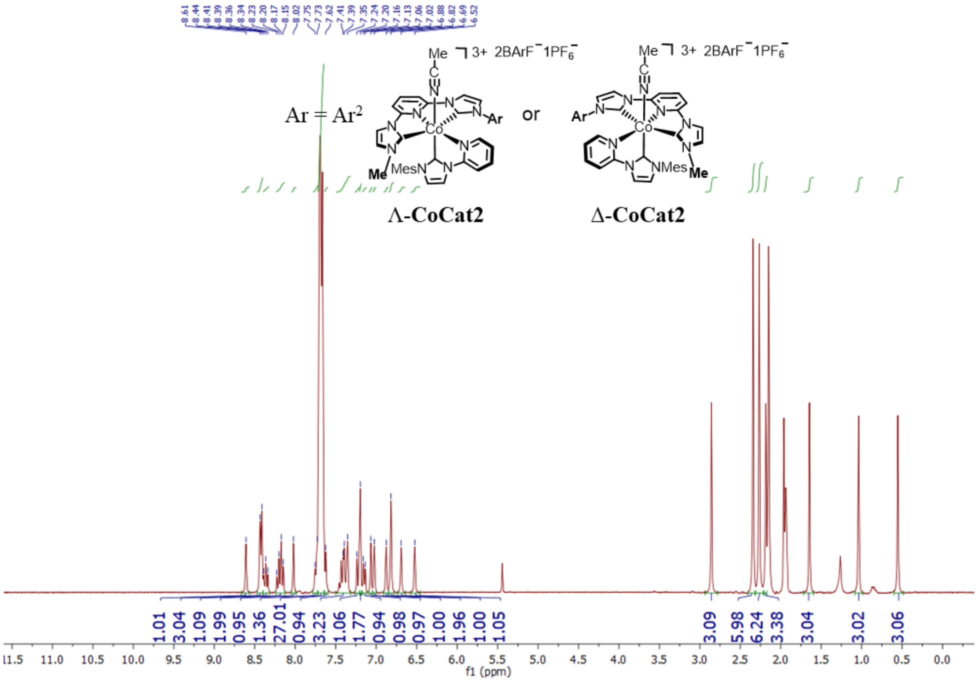

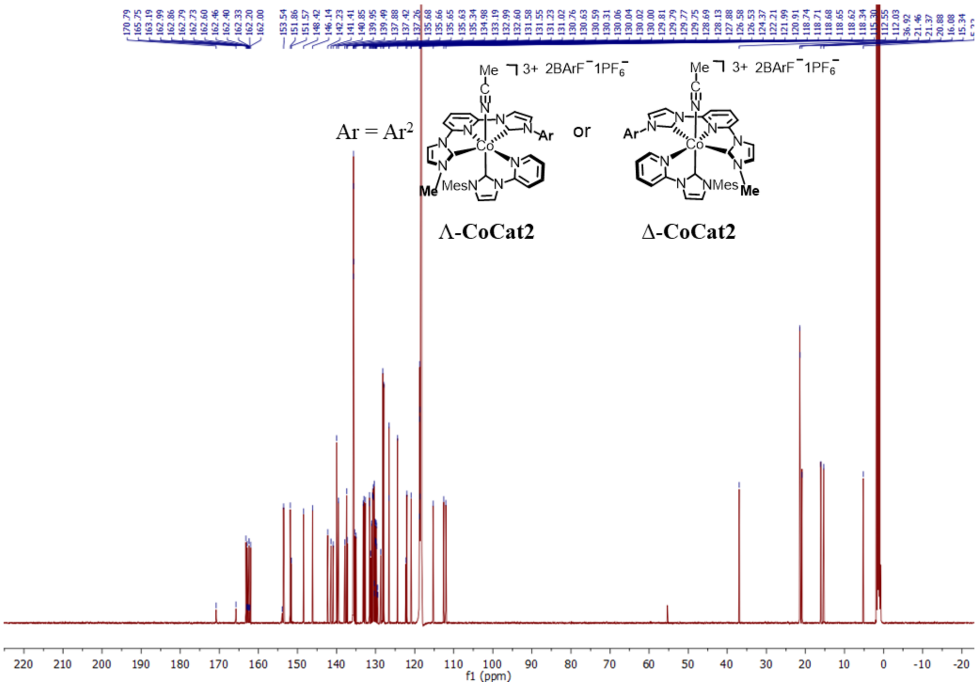

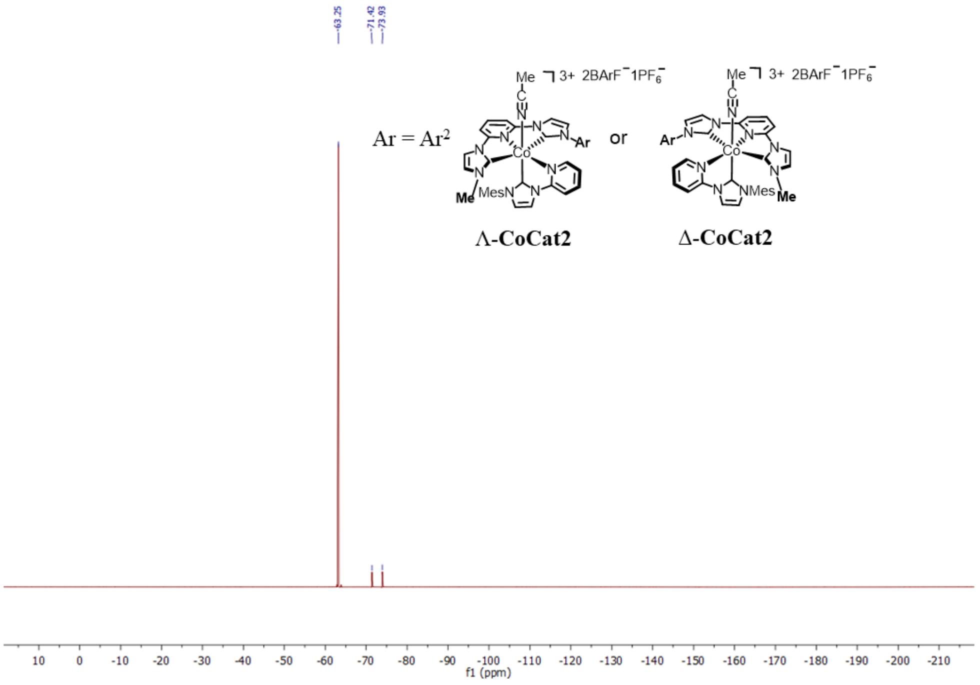

Supplementary Fig. 65. ^{1}H NMR (300 MHz, 298 K), ^{13}C NMR (126 MHz, 298 K) and ^{19}F NMR (282 MHz, 298 K) spectra of Λ-**CoCat2** or Δ-**CoCat2** in CD_3CN.

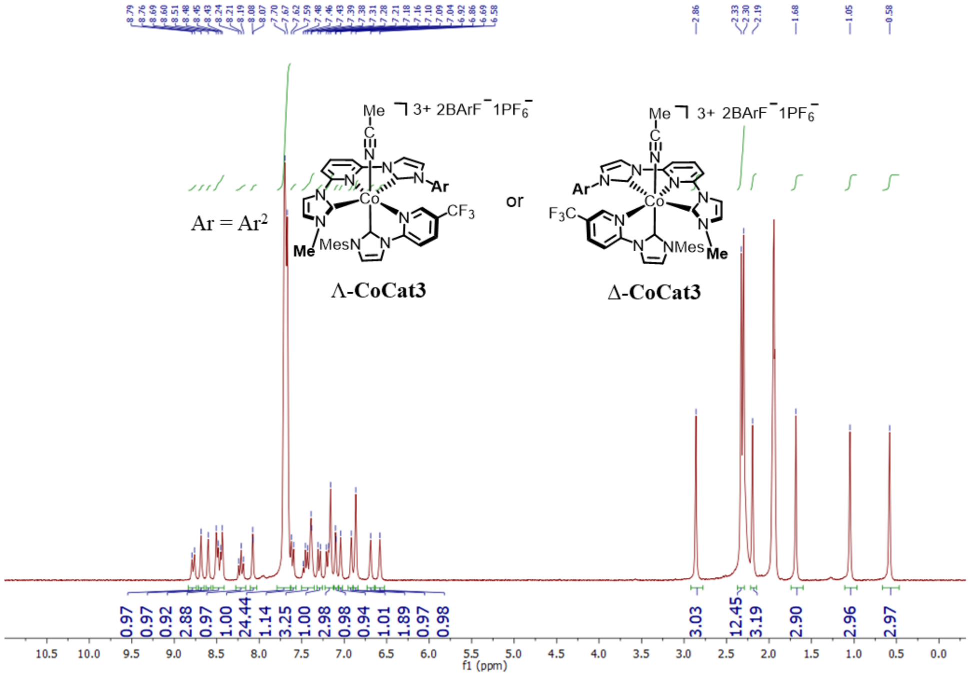
Ar = Ar2
Λ-CoCat3
or
Δ-CoCat3
3+ 2BArF⁻ 1PF6⁻
f1 (ppm)

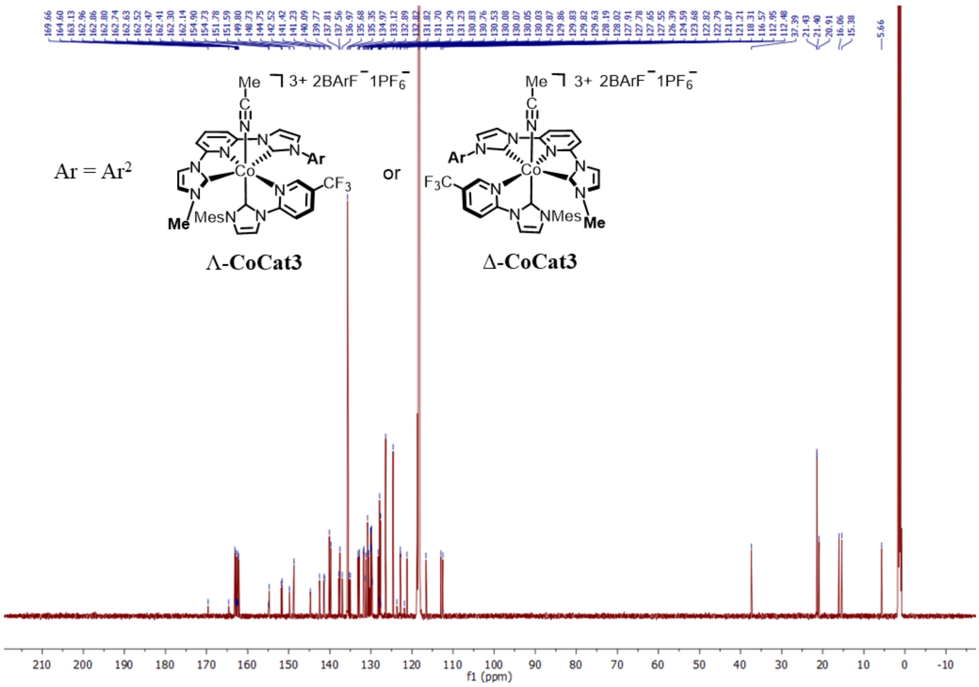
Ar = Ar2
Λ-CoCat3
or
Δ-CoCat3
3+ 2BArF⁻ 1PF6⁻
f1 (ppm)

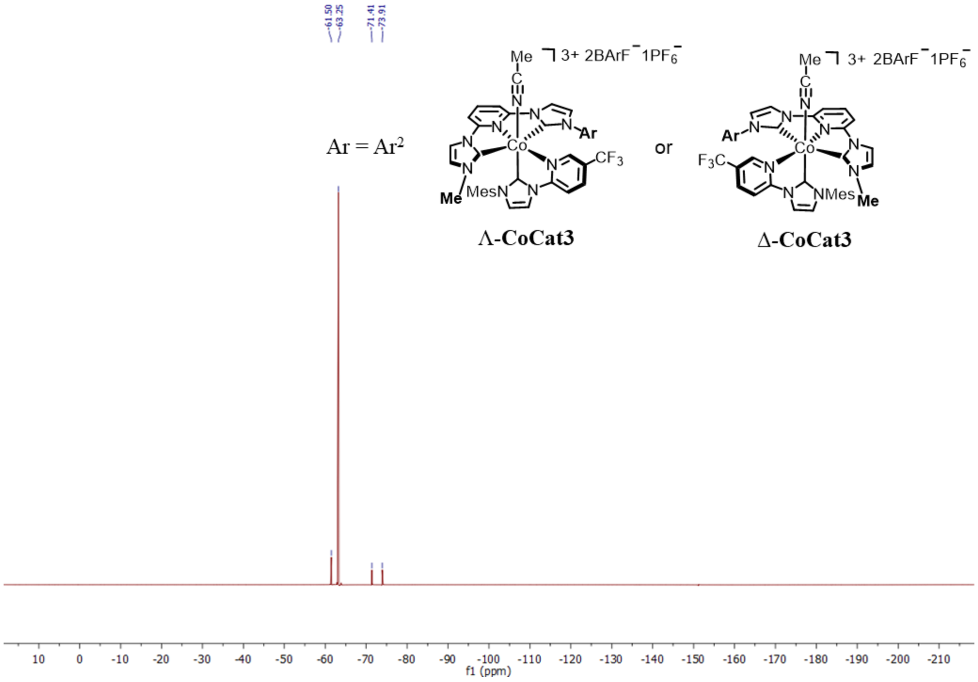

Supplementary Fig. 66. ^{1}H NMR (300 MHz, 298 K), ^{13}C NMR (151 MHz, 298 K) and ^{19}F NMR (282 MHz, 298 K) spectra of Λ-**CoCat3** or Δ-**CoCat3** in CD_3CN.

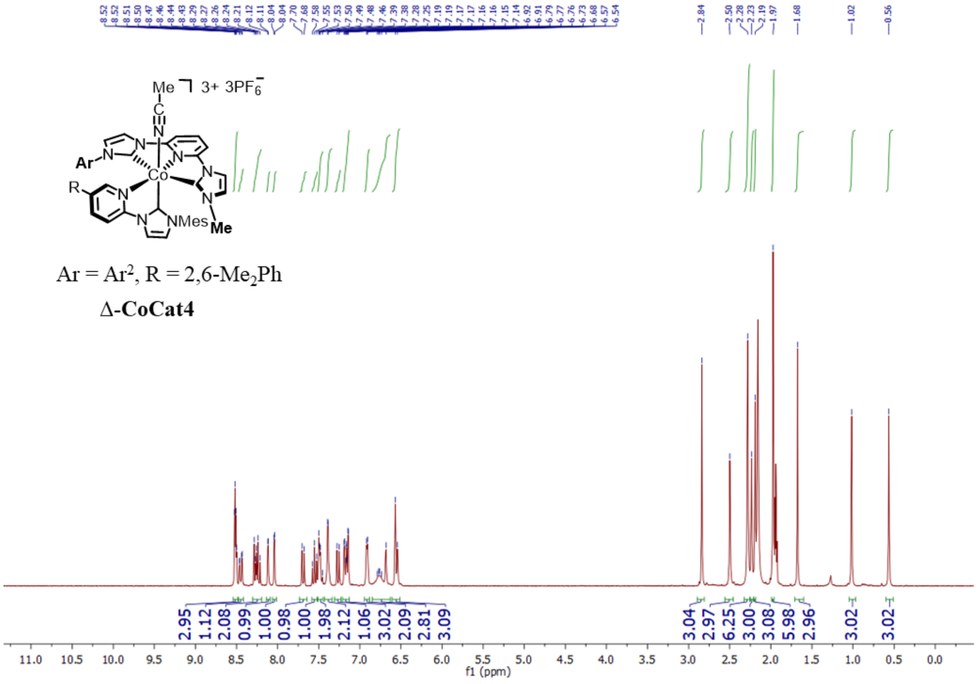

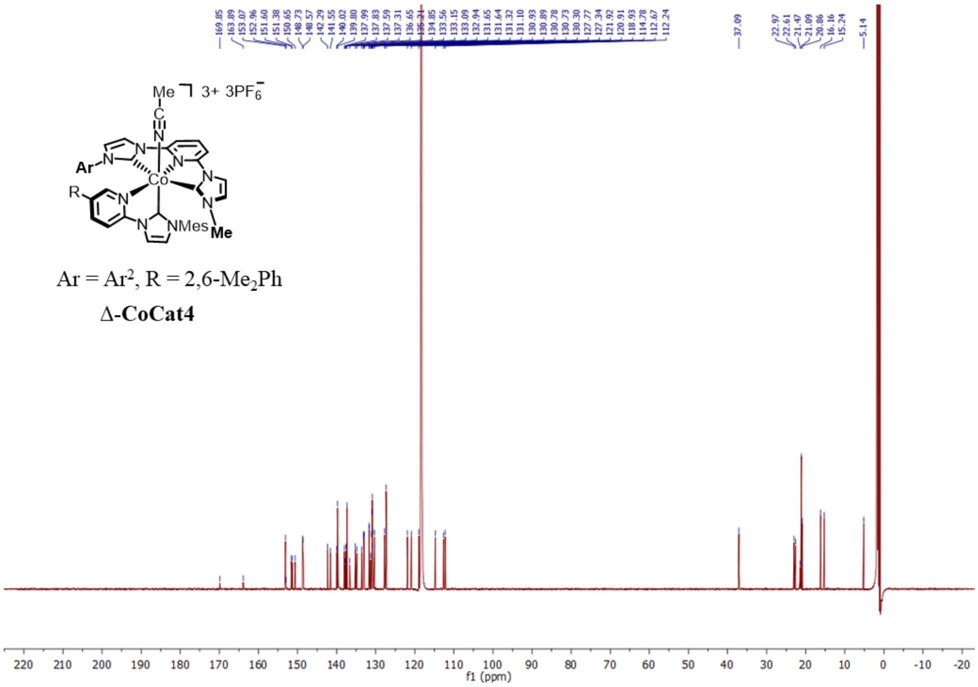

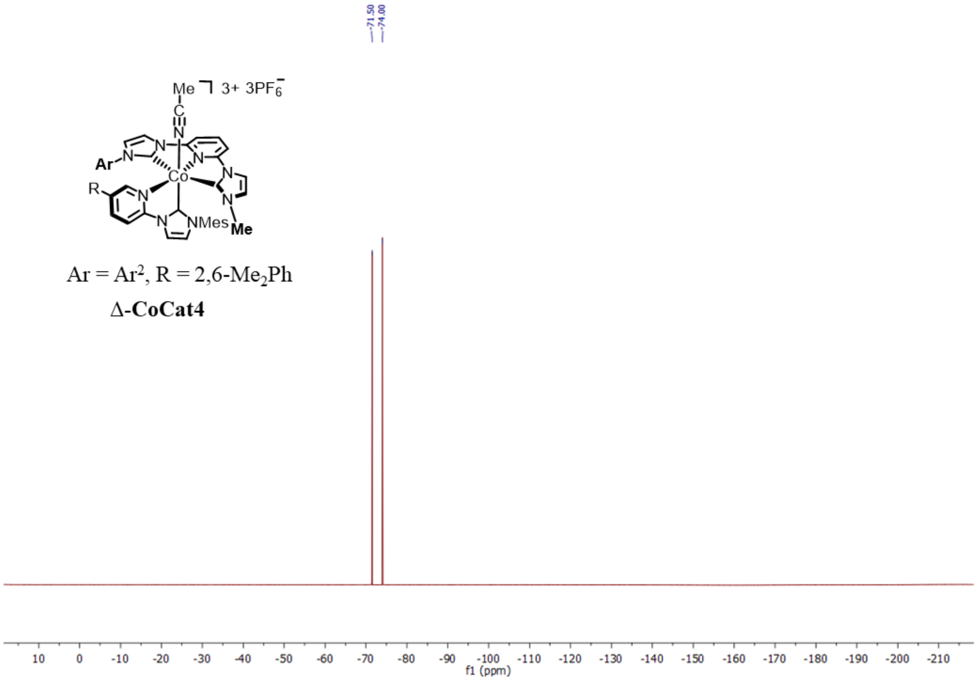

Supplementary Fig. 67. ^{1}H NMR (300 MHz, 298 K), ^{13}C NMR (126 MHz, 298 K) and ^{19}F NMR (282 MHz, 298 K) spectra of Δ-**CoCat4** in CD_3CN.

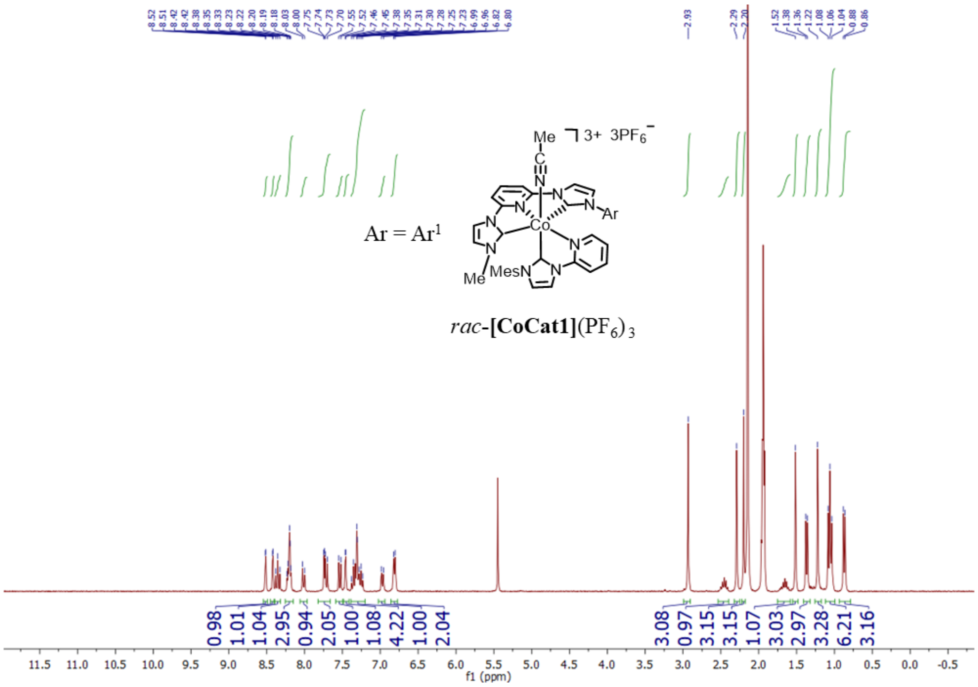
Ar = Ar1
rac-[CoCat1](PF6)3
f1 (ppm)

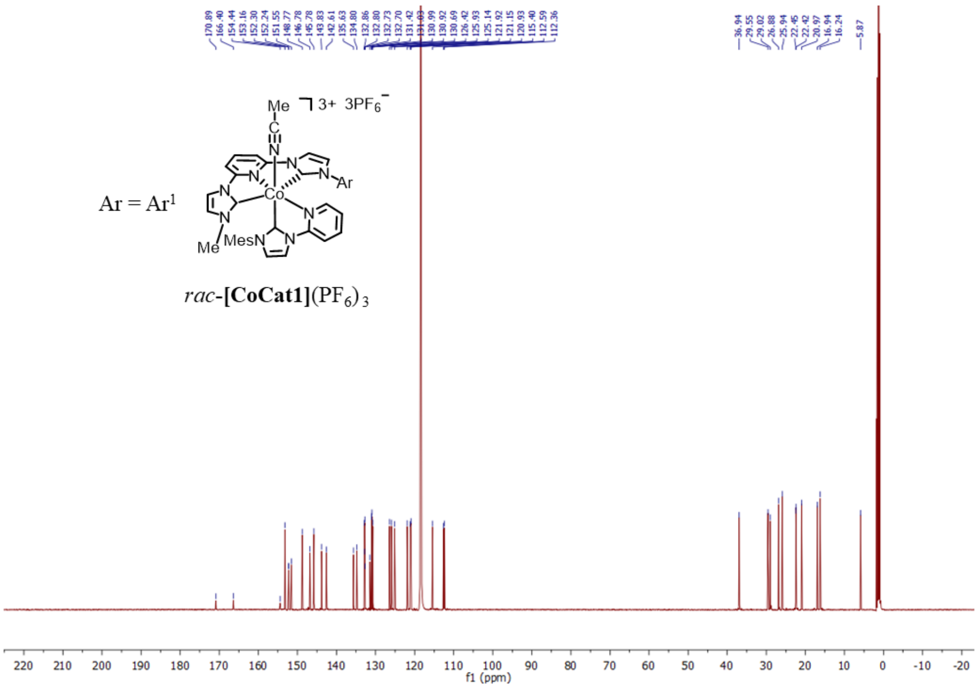
Ar = Ar1
rac-[CoCat1](PF6)3
f1 (ppm)

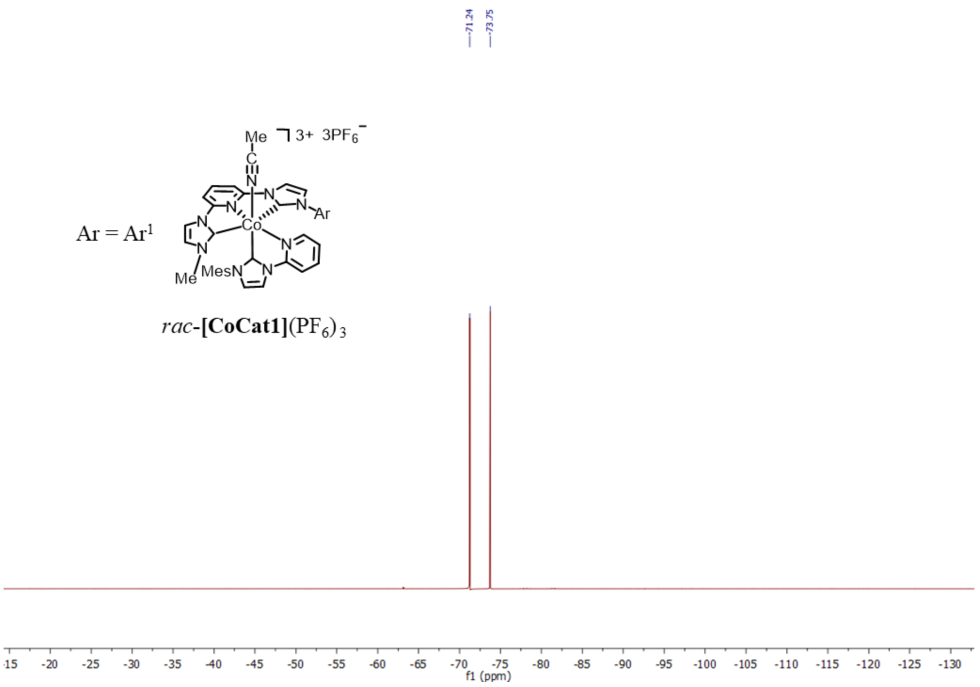

Supplementary Fig. 68. ^{1}H NMR (300 MHz, 298 K), ^{13}C NMR (126 MHz, 298 K) and ^{19}F NMR (282 MHz, 298 K) of *rac*-**[CoCat1]**$(PF_6)_3$ in CD_3CN.

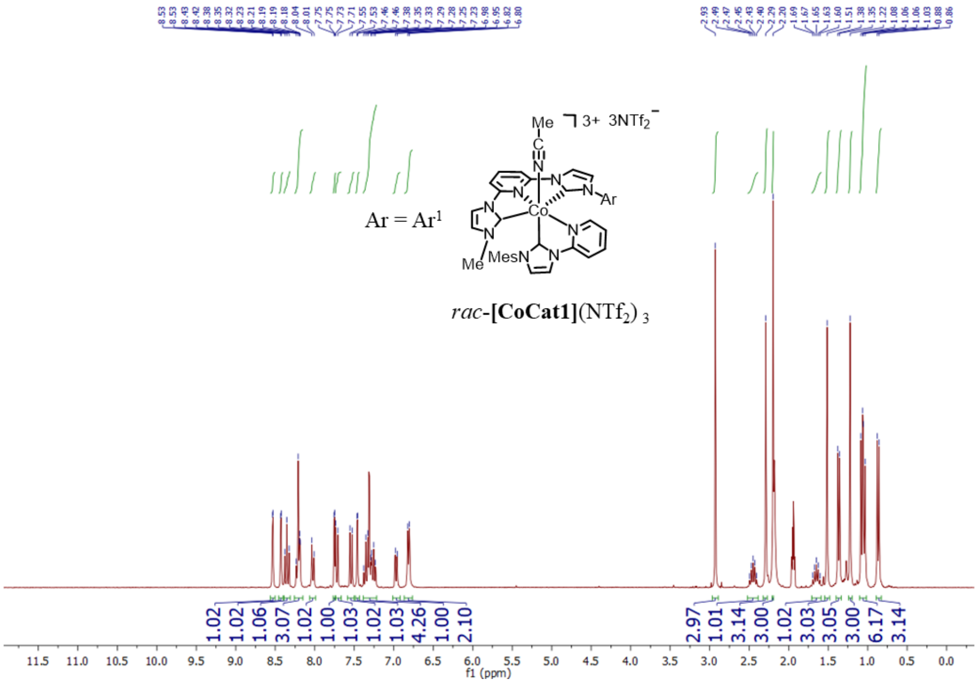

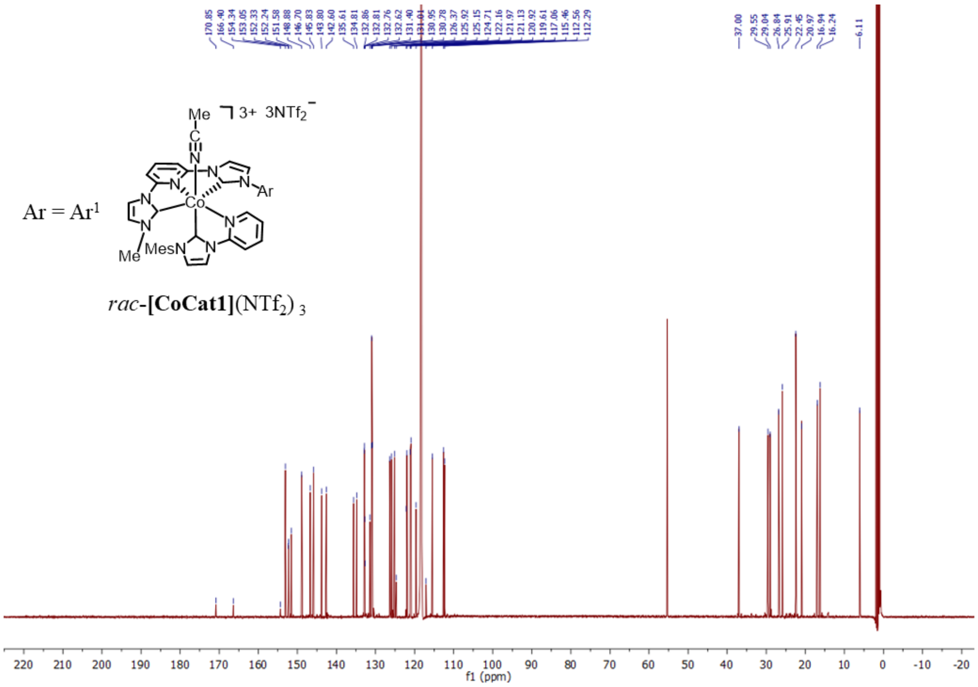

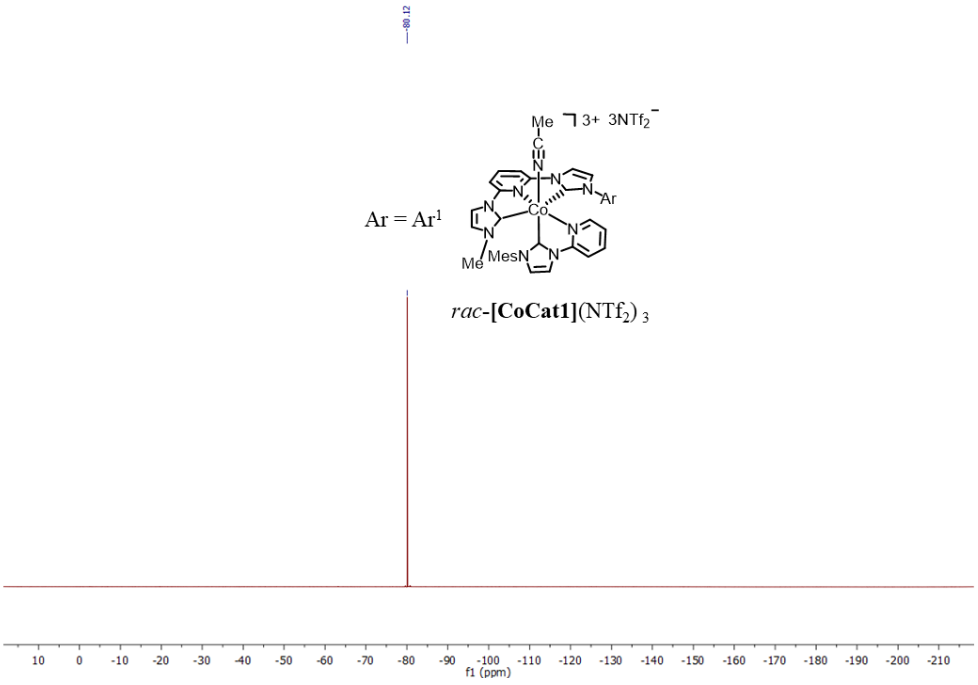

Supplementary Fig. 69. ^{1}H NMR (300 MHz, 298 K), ^{13}C NMR (126 MHz, 298 K) and ^{19}F NMR (282 MHz, 298 K) spectra of *rac*-**[CoCat1]**$(NTf_2)_3$ in CD_3CN.

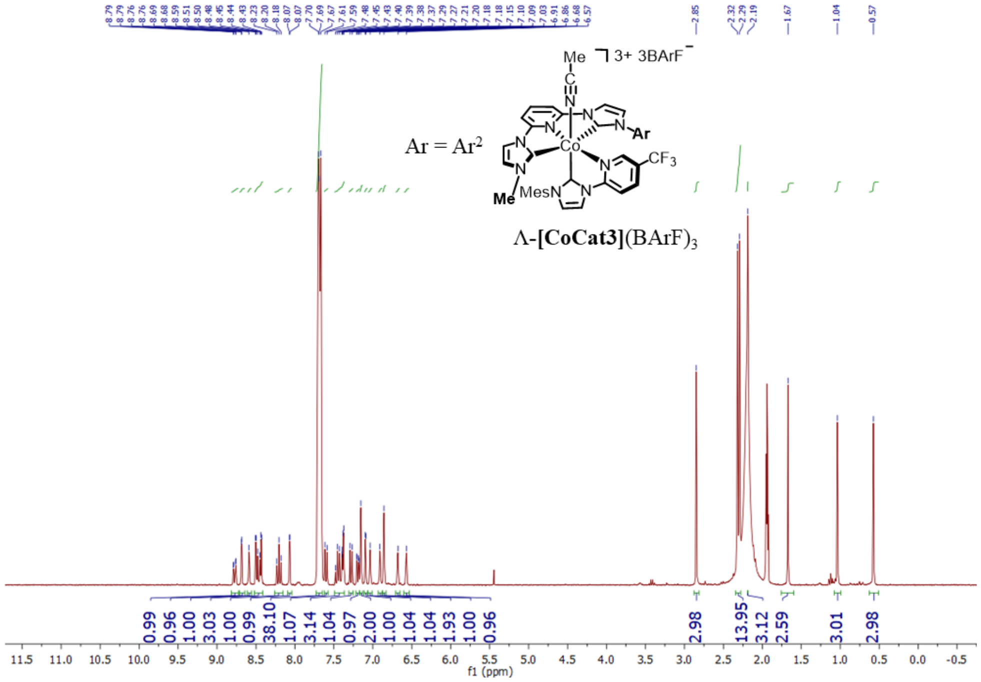

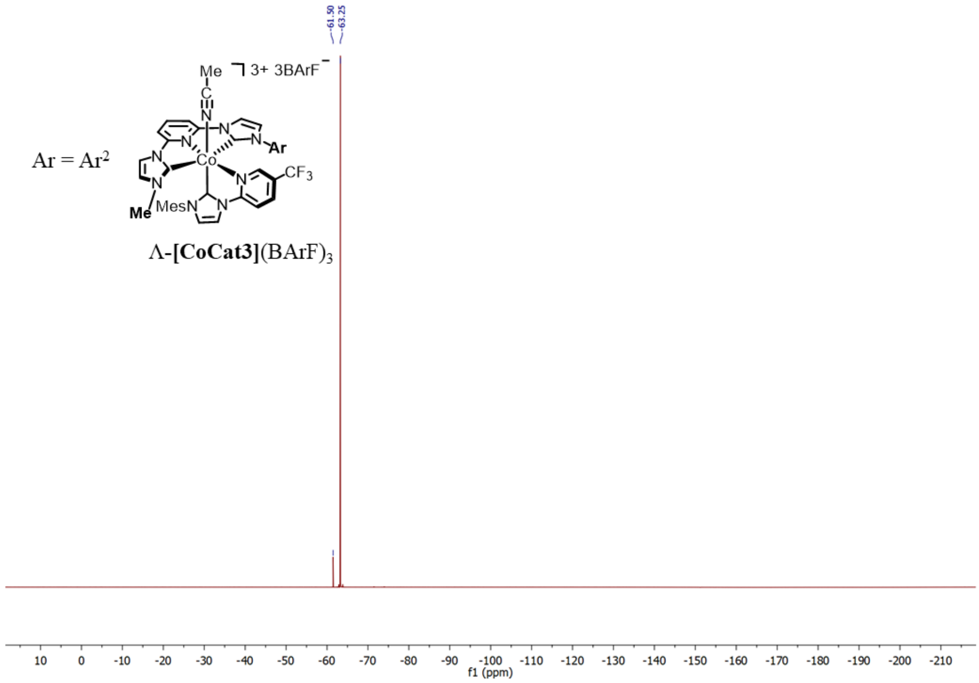

Supplementary Fig. 70. ^{1}H NMR (300 MHz, 298 K) and ^{19}F NMR spectra (126 MHz, 298 K) of Λ-**[CoCat3]**(BArF)$_3$ in CD_3CN.

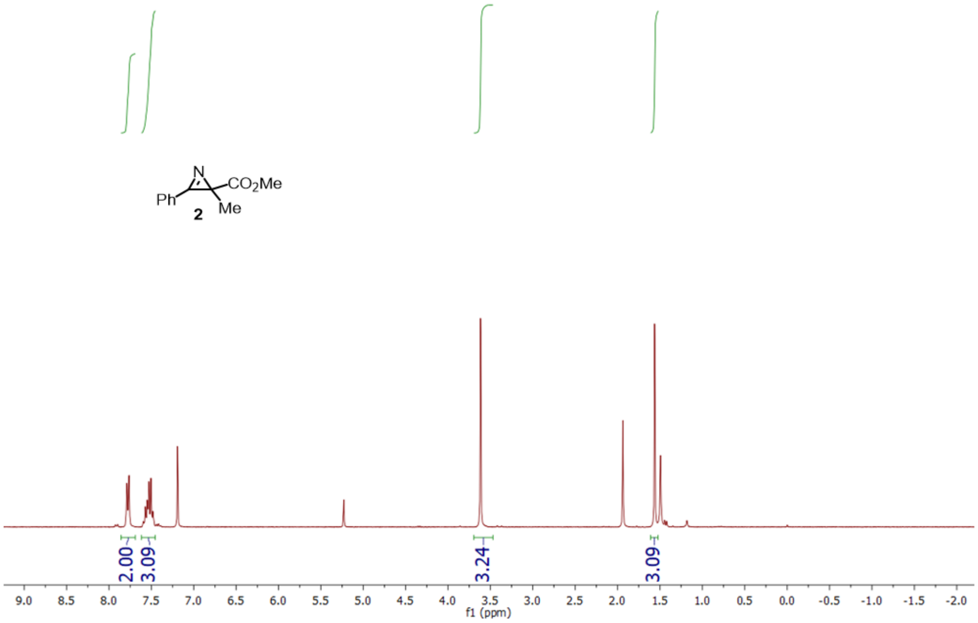

Supplementary Fig. 71. ^{1}H NMR (300 MHz, 298 K) of product **2** in $CDCl_3$.

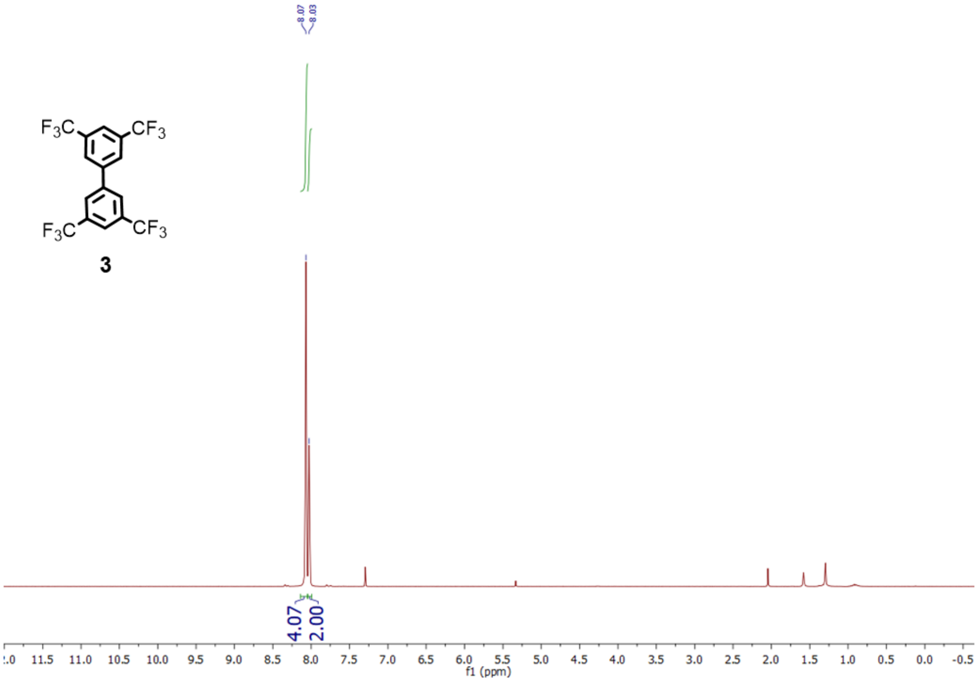

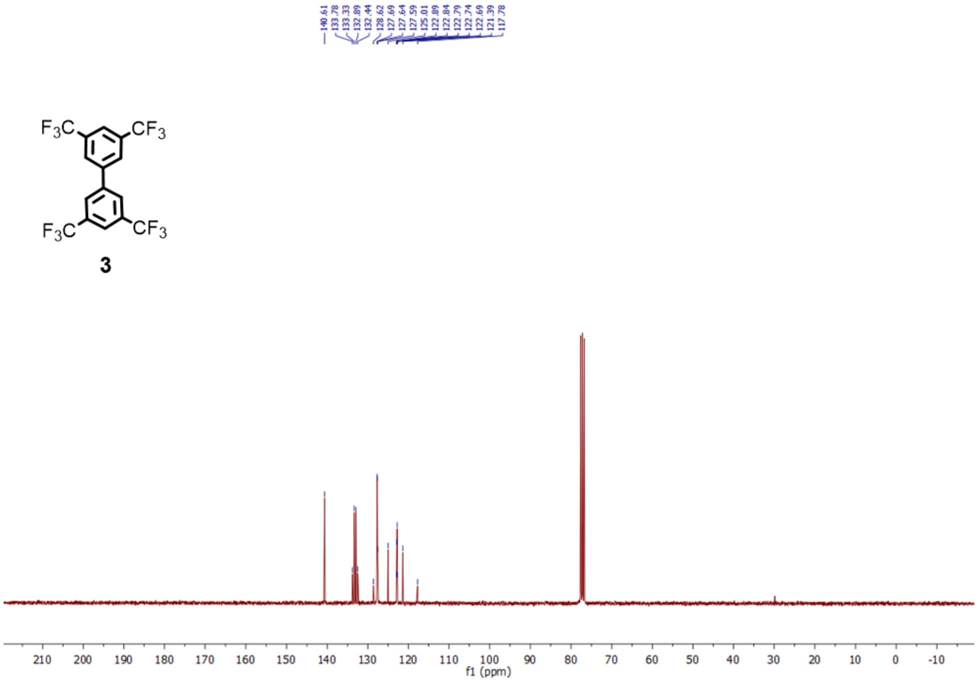

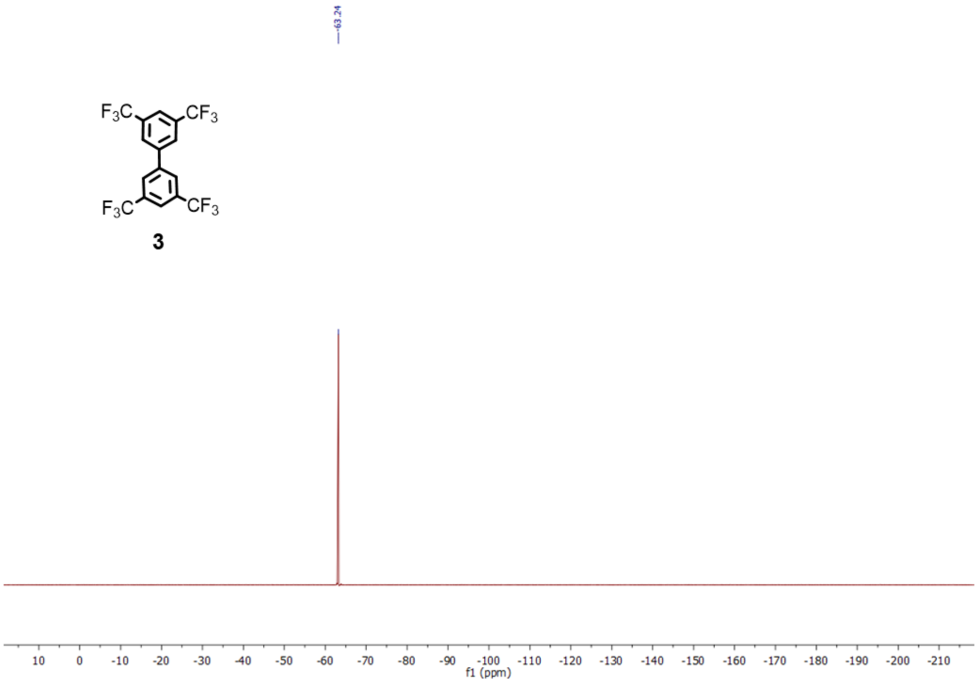

Supplementary Fig. 72. ^{1}H NMR (300 MHz, 298 K), ^{13}C NMR (75 MHz, 298 K) and ^{19}F NMR (282 MHz, 298 K) of product **3** in $CDCl_3$.

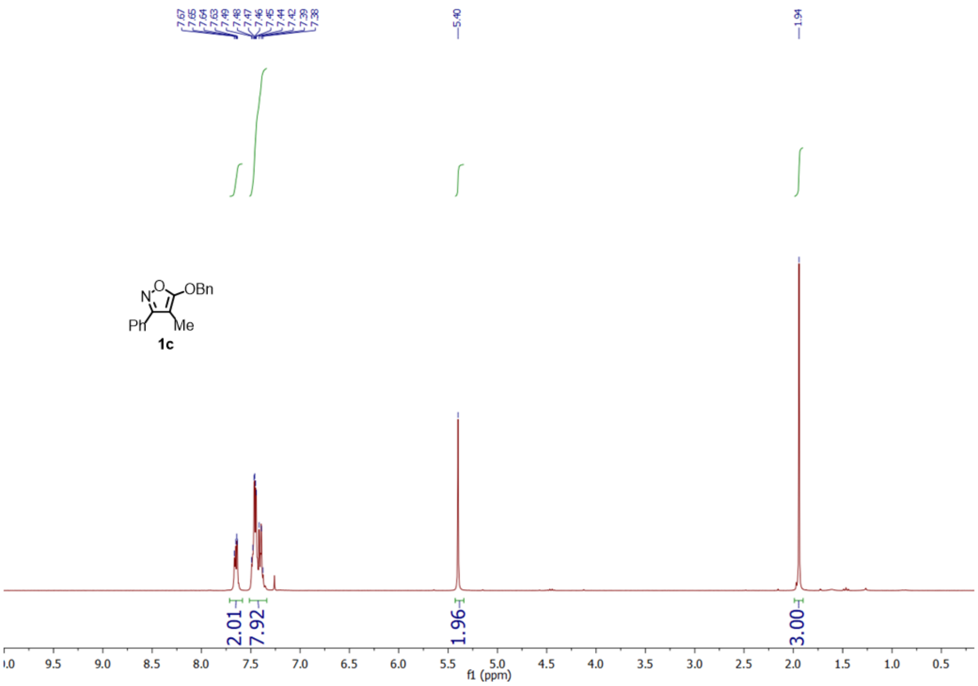

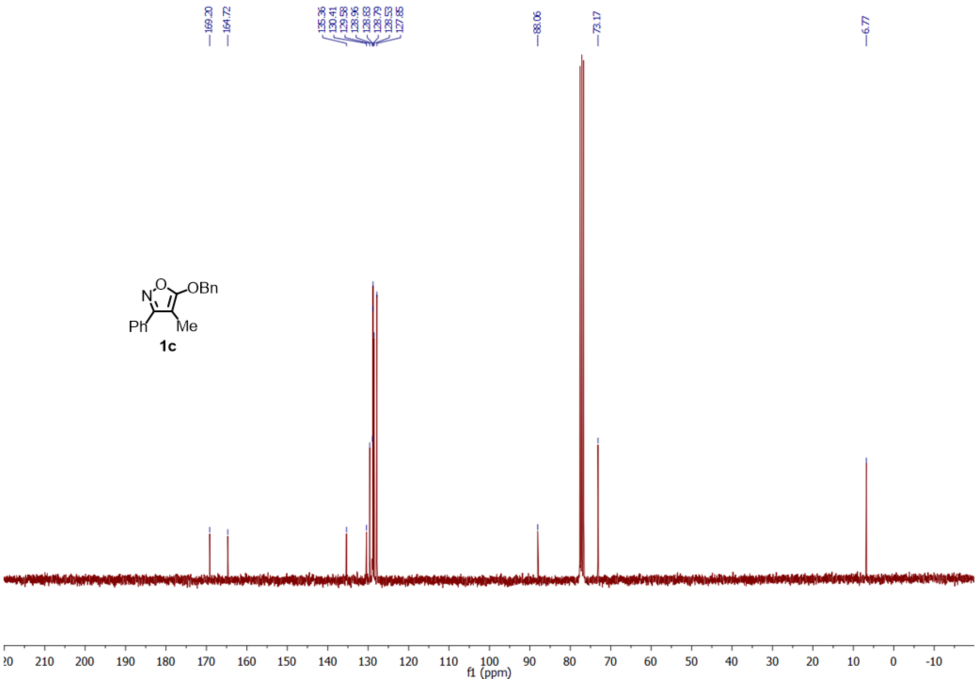

Supplementary Fig. 73. ^{1}H NMR (300 MHz, 298 K) and ^{13}C NMR (75 MHz, 298 K) spectra of substrate **1c** in $CDCl_3$.

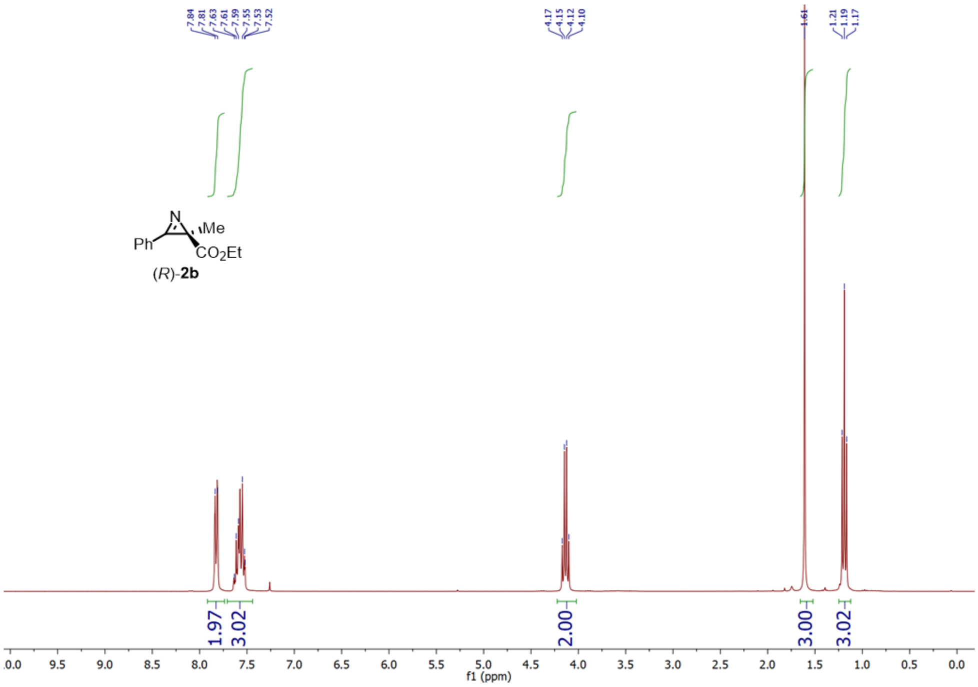

Supplementary Fig. 74. ^{1}H NMR (300 MHz, 298 K) spectrum of product **2b** in $CDCl_3$.

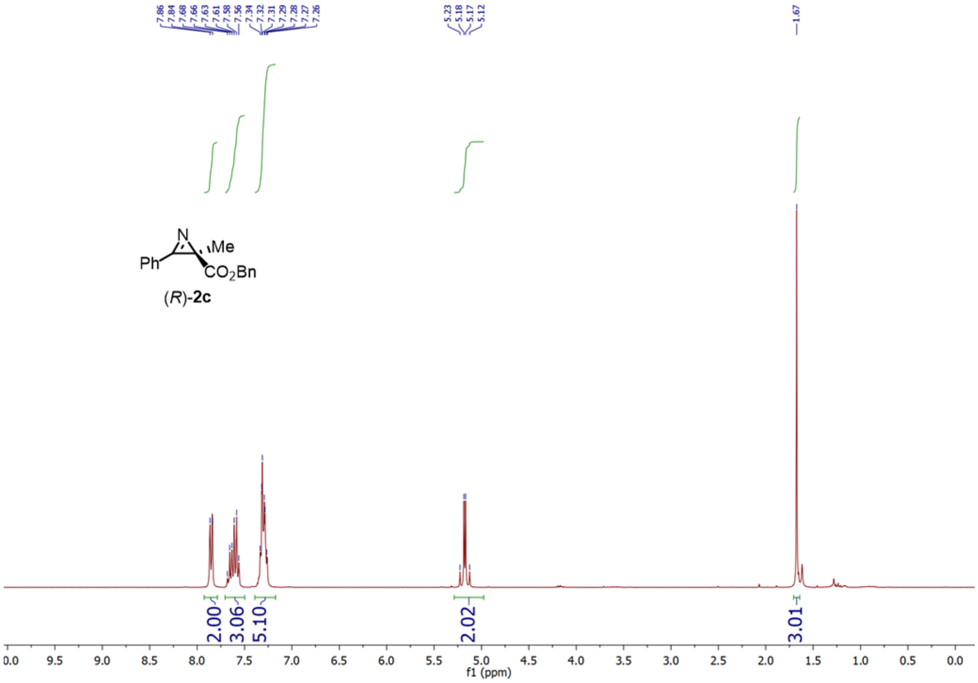

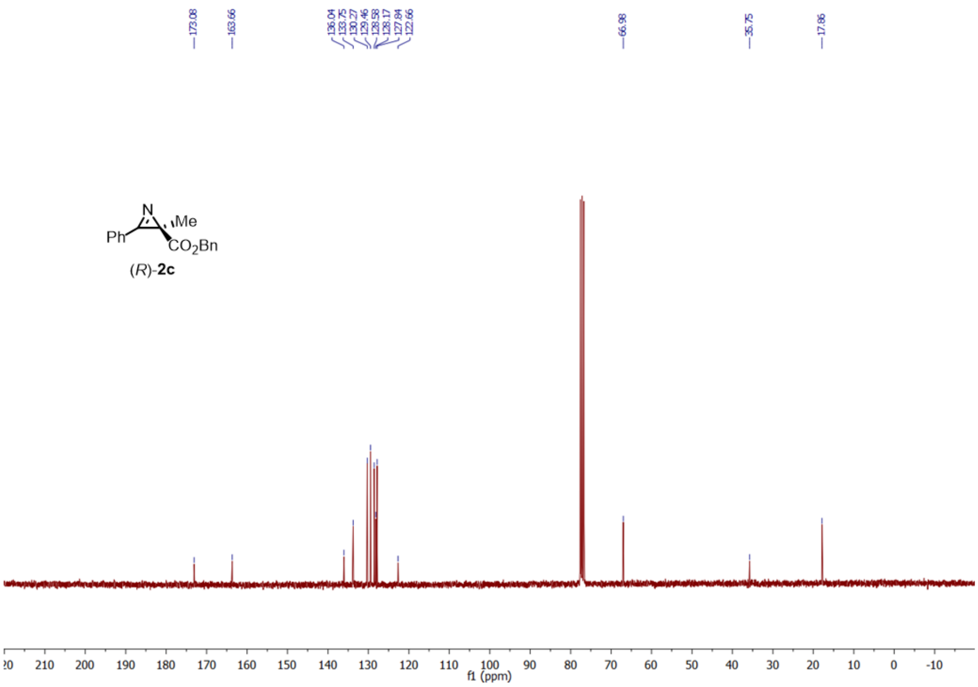

Supplementary Fig. 75. ^{1}H NMR (300 MHz, 298 K) and ^{13}C NMR (75 MHz, 298 K) spectra of product **2c** in $CDCl_3$.

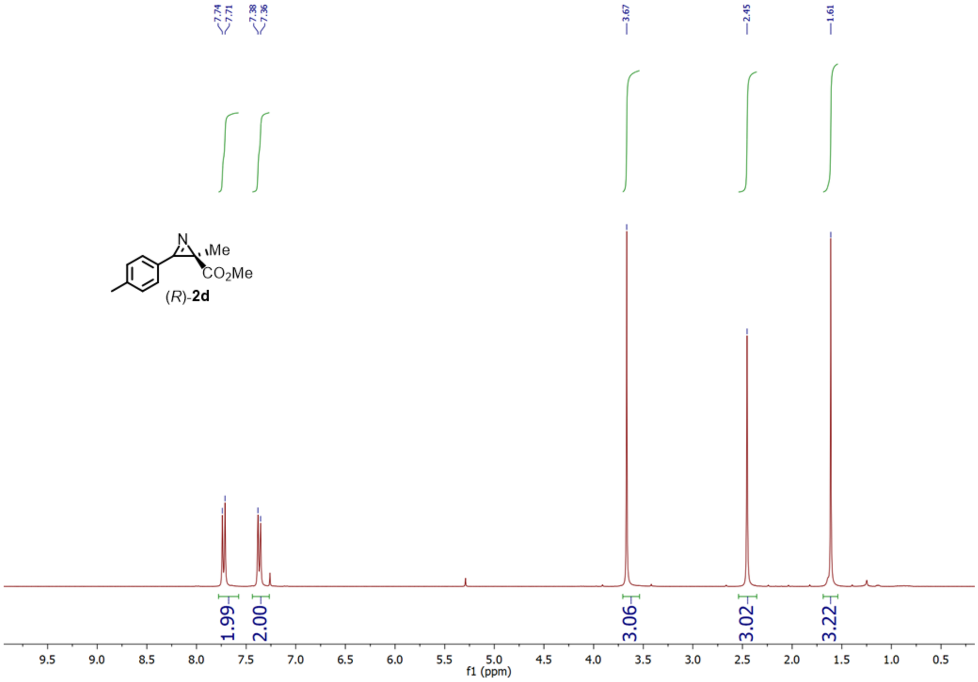

Supplementary Fig. 76. ^{1}H NMR (300 MHz, 298 K) spectrum of product **2d** in $CDCl_3$.

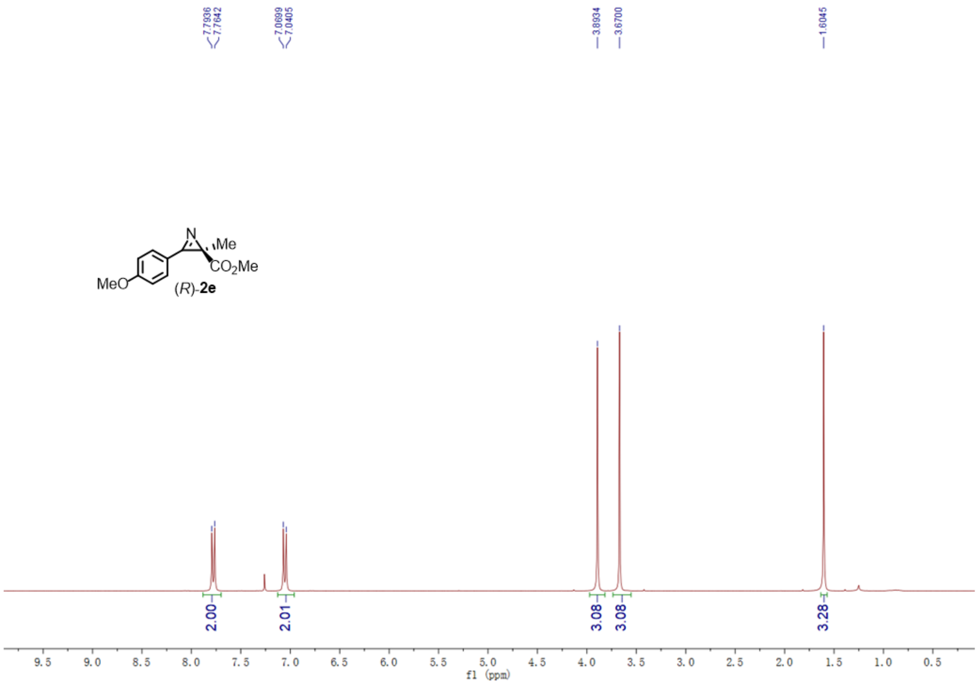

Supplementary Fig. 77. ^{1}H NMR (300 MHz, 298 K) spectrum of product **2e** in $CDCl_3$.

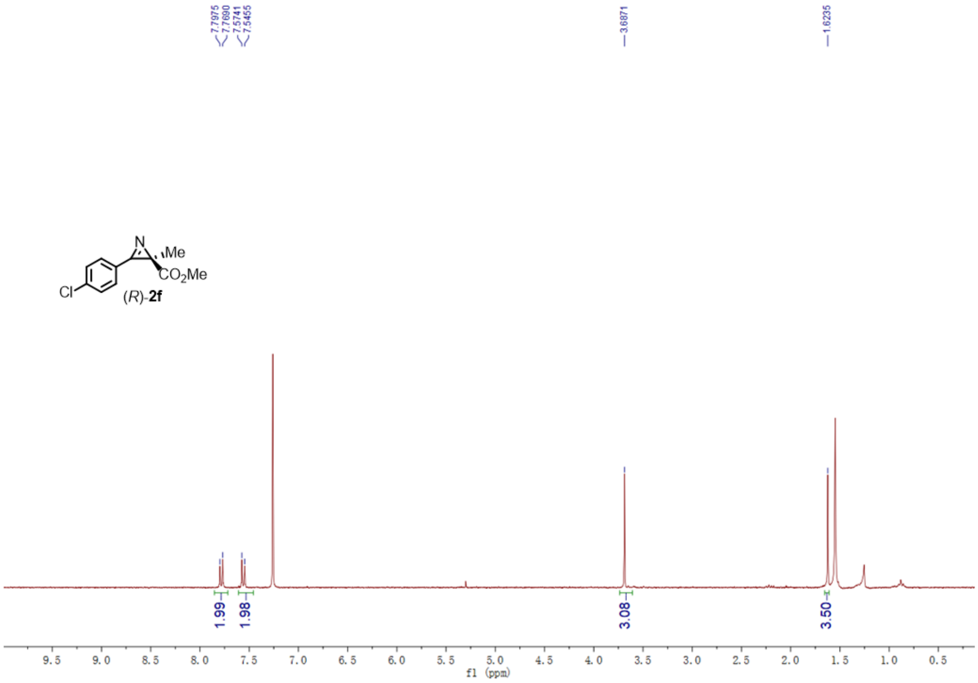

Supplementary Fig. 78. ^{1}H NMR (300 MHz, 298 K) spectrum of product **2f** in $CDCl_3$.

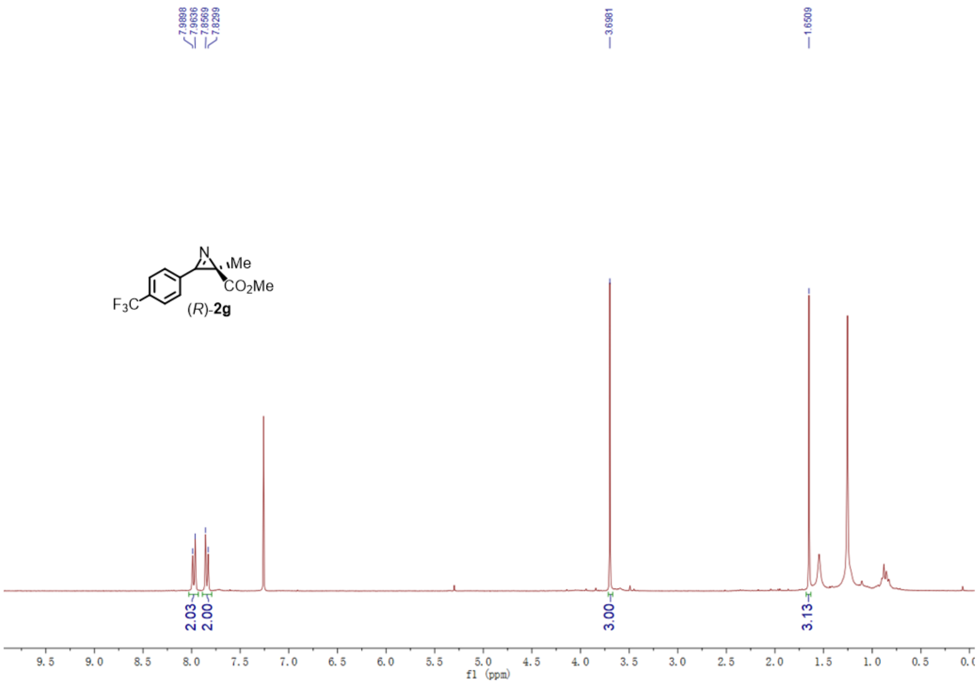

Supplementary Fig. 79. ^{1}H NMR (300 MHz, 298 K) spectrum of product **2g** in $CDCl_3$.

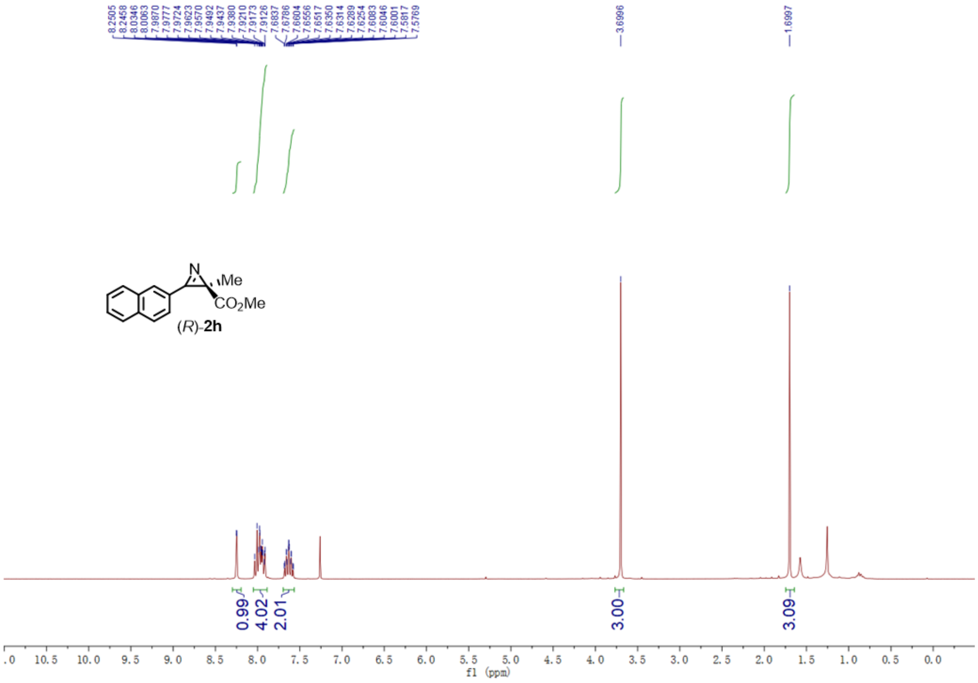

Supplementary Fig. 80. ^{1}H NMR (300 MHz, 298 K) spectrum of product **2h** in $CDCl_3$.

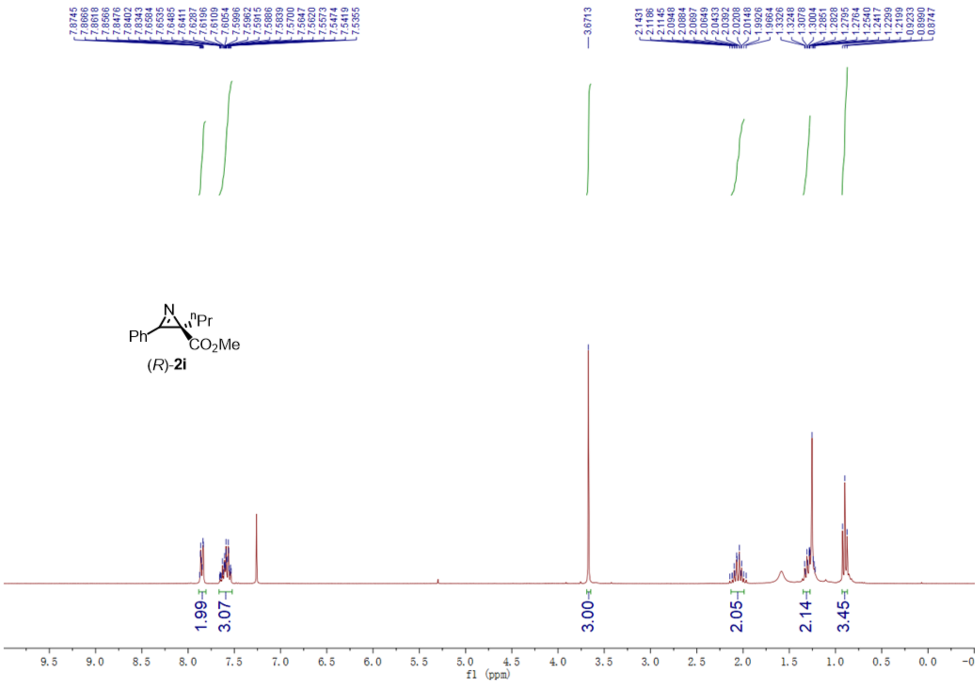

Supplementary Fig. 81. ^{1}H NMR (300 MHz, 298 K) spectrum of product **2i** in $CDCl_3$.

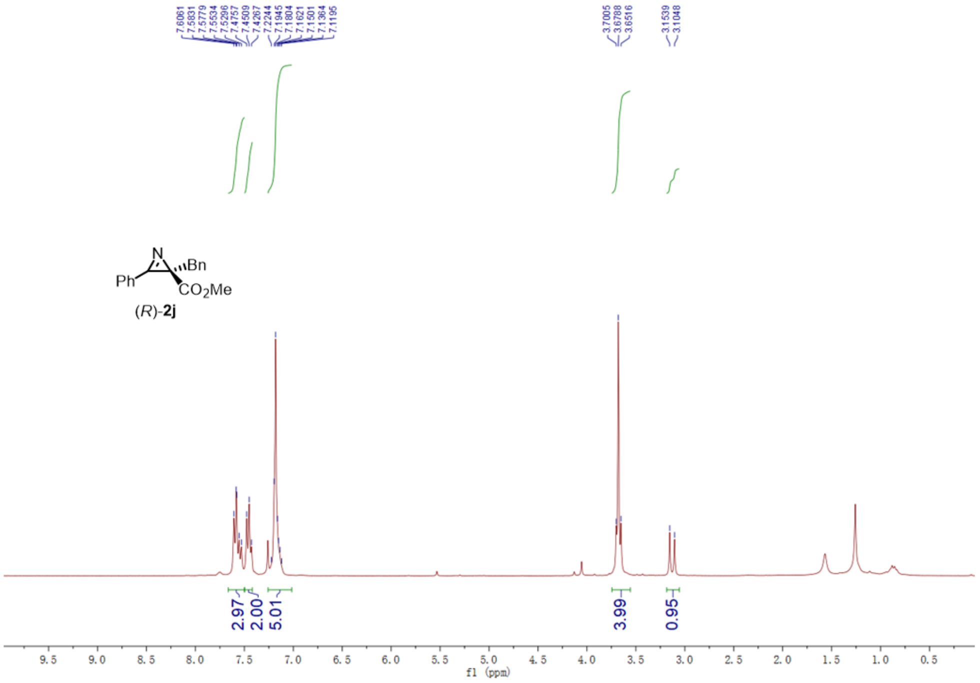

Supplementary Fig. 82. ^{1}H NMR (300 MHz, 298 K) spectrum of product **2j** in $CDCl_3$.

14. References

1. Meinhard, D. *et al.* New Nickel(II) Diimine Complexes and the Control of Polyethylene Microstructure by Catalyst Design. *J. Am. Chem. Soc.* **129**, 9182–9191 (2007).

2. Zhang, H. *et al.* A Modified Procedure for the Synthesis of 1-Arylimidazoles. *Synthesis* **17**, 2661–2666 (2003).

3. Wang, L., Liu, N. & Dai, B. Metal-free site-selective C–N bond-forming reaction of polyhalogenated pyridines and pyrimidines. *RSC Adv.* **5**, 82097–82111 (2015).

4. Hong, Y., Jarrige, L., Harms, K. & Meggers, E. Chiral-at-Iron Catalyst: Expanding the Chemical Space for Asymmetric Earth-Abundant Metal Catalysis. *J. Am. Chem. Soc.* **141**, 4569–4572 (2019).

5. Steinlandt, P. S., Hemming, M., Xie, X., Ivlev, S. I. & Meggers, E. Trading Symmetry for Stereoinduction in Tetradentate, non-C_2-Symmetric Fe(II)-Complexes for Asymmetric Catalysis. *Chem. Eur. J.* **29**, (2023).

6. Nie, X., Ye, C., Ivlev, S. I. & Meggers, E. Nitrene-Mediated C−H Oxygenation: Catalytic Enantioselective Formation of Five-Membered Cyclic Organic Carbonates. *Angew. Chem. Int. Ed.* **61**, e202211971 (2022).

7. Xi, Z., Liu, B., Lu, C. & Chen, W. Cobalt(III) complexes bearing bidentate, tridentate, and tetradentate N-heterocyclic carbenes: synthesis, X-ray structures and catalytic activities. *Dalton Trans.* **35,** 7008–7014 (2009).

8. Liu, X., Pan, S., Wu, J., Wang, Y. & Chen, W. A Planar π-Conjugated Naphthyridine-Based N-Heterocyclic Carbene Ligand and Its Derived Transition-Metal Complexes. *Organometallics* **32**, 209–217 (2012).

9. Steinlandt, P. S., Xie, X., Ivlev, S. & Meggers, E. Stereogenic-at-Iron Catalysts with a Chiral Tripodal Pentadentate Ligand. *ACS Catal.* **11**, 7467–7476 (2021).

10. Budiman, Y. P. *et al.* Palladium-Catalyzed Homocoupling of Highly Fluorinated Arylboronates: Studies of the Influence of Strongly vs Weakly Coordinating Solvents on the Reductive Elimination Process. *J. Am. Chem. Soc.***142**, 6036–6050 (2020).

11. Miranda-Pizarro, J., Navarro, M. & Campos, J. Multiple C–B Bond Cleavage Reactions at $[BAr^F_4]^-$ Anions Mediated by Terphenyl Phosphine Gold Catalysts. *Organometallics* **44**, 340–346 (2024).

12. Garduño, J. A., Glueck, D. S., Hernandez, R. E., Figueroa, J. S. & Rheingold, A. L. Protonolysis of the $[B(Ar_F)_4]^-$ Anion Mediated by Nucleophile/Electrophile/Water Cooperativity in a Platinum–PMe_2OH Complex. *Organometallics* **41**, 1475–1479 (2022).

13. Lai, Y.-Y., Bornand, M. & Chen, P. Homogeneous Model Complexes for Supported Rhenia Metathesis Catalysts. *Organometallics* **31**, 7558–7565 (2012).

14. APEX3 V2019.11-2, Bruker AXS Inc., Madison, Wisconsin, USA, (2019).

15. SADABS, Bruker AXS Inc., Madison, Wisconsin, USA, (2016).

16. Krause, L., Herbst-Irmer, R., Sheldrick, G. M. & Stalke, D. Comparison of silver and molybdenum microfocus X-ray sources for single-crystal structure determination. *J. Appl. Crystallogr*. **48**, 3–10 (2015).

17. Sheldrick,G. M. SHELXT-Integrated Space-Group and Crystal-Structure Determination. *Acta Crystallogr*. **A71**, 3–8 (2015).

18. Sheldrick,G. M. Crystal Structure Refinement with SHELXL. *Acta Crystallogr*. **C71**, 3–8 (2015).

19. Hübschle, C. B., Sheldrick, G. M. & Dittrich, B. *ShelXle*: a Qt graphical user interface for*SHELXL*. *J. Appl. Crystallogr*. **44**, 1281–1284 (2011).

20. Kratzert, D. & Krossing, I. Recent improvements inDSR. *J. Appl. Crystallogr*. **51**, 928–934 (2018).

21. Spek, A. L. *PLATON*SQUEEZE: a tool for the calculation of the disordered solvent contribution to the calculated structure factors. *Acta Crystallographica Sect. C: Struct. Chem*. **71**, 9–18 (2015).

22. Spek, A. L. PLATON - *A Multipurpose Crystallographic Tool*, Utrecht University, Utrecht, The Netherlands, (2019).

23. *X-Area*, STOE & Cie GmbH, Darmstadt, Germany (2018).

24. *LANA - Laue Analyzer*, STOE & Cie GmbH, Darmstadt, Germany (2019).

25. *X-RED32*, STOE & Cie GmbH, Darmstadt, Germany (2018).
